# Expanding the Diversity at the C-4 Position of Pyrido[2,3-*d*]pyrimidin-7(8*H*)-ones to Achieve Biological Activity against ZAP-70

**DOI:** 10.3390/ph14121311

**Published:** 2021-12-15

**Authors:** Victor Masip, Ángel Lirio, Albert Sánchez-López, Ana B. Cuenca, Raimon Puig de la Bellacasa, Pau Abrisqueta, Jordi Teixidó, José I. Borrell, Albert Gibert, Roger Estrada-Tejedor

**Affiliations:** 1 Grup de Química Farmacèutica, IQS School of Engineering, Universitat Ramon Llull, Via Augusta 390, E-08017 Barcelona, Spain; victormasipb@iqs.url.edu (V.M.); angelliriom@iqs.url.edu (Á.L.); albertsanchez1@iqs.url.edu (A.S.-L.); anabelen.cuenca@iqs.url.edu (A.B.C.); raimon.puig@iqs.url.edu (R.P.d.l.B.); jordi.teixido@iqs.url.edu (J.T.); j.i.borrell@iqs.url.edu (J.I.B.); albert.gibert@iqs.url.edu (A.G.); 2 Department of Hematology, Hospital Universitari Vall d’Hebron, Experimental Hematology, Vall d’Hebron Institute of Oncology (VHIO), Passeig de la Vall d’Hebron 119-129, E-08035 Barcelona, Spain; pau.abrisqueta9@gmail.com

**Keywords:** pyrido[2,3-*d*]pyrimidines, cross-coupling, tyrosine kinase inhibitors, ZAP-70

## Abstract

Pyrido[2,3-*d*]pyrimidin-7(8*H*)-ones have attracted widespread interest due to their similarity with nitrogenous bases found in DNA and RNA and their potential applicability as tyrosine kinase inhibitors. Such structures, presenting up to five diversity centers, have allowed the synthesis of a wide range of differently substituted compounds; however, the diversity at the C4 position has mostly been limited to a few substituents. In this paper, a general synthetic methodology for the synthesis of 4-substituted-2-(phenylamino)-5,6-dihydropyrido[2,3-*d*]pyrimidin-7(8*H*)-ones is described. By using cross-coupling reactions, such as Ullmann, Buchwald–Hartwig, Suzuki–Miyaura, or Sonogashira reactions, catalyzed by Cu or Pd, we were able to describe new potential biologically active compounds. The resulting pyrido[2,3-*d*]pyrimidin-7(8*H*)-ones include *N*-alkyl, *N*-aryl, *O*-aryl, *S*-aryl, aryl, and arylethynyl substituents at C4, which have never been explored in connection with the biological activity of such heterocycles as tyrosine kinase inhibitors, in particular as ZAP-70 inhibitors.

## 1. Introduction

Zeta-chain-associated protein kinase 70 kDa (ZAP-70) is a non-receptor tyrosine kinase belonging to the Syk family mainly expressed in T lymphocytes and NK cells. After the activation of the T cell receptor (TCR) by an antigen, ZAP-70 is recruited by the ITAM motifs of CD3Z chains of the TCR complex and activated. Upon phosphorylation, ZAP-70 phosphorylates LAT and SLP-76, which are responsible for the proliferation and survival of T lymphocytes [1,2]. Given this, ZAP-70 plays a pivotal role in the regulation and adaptive immune signaling of T cells, thus it has been identified as a promising therapeutic target for some diseases like peripheral T cell lymphoma (PTCL). Despite its significance in T cell activation, few compounds have been reported as ZAP-70 inhibitors and they have low affinity [3] or are unselective [2]. Irreversible inhibitor RDN009 has been recently described [4] as a good strategy to improve selectivity, albeit their pharmacokinetic properties need to be improved.

**Figure 1:**
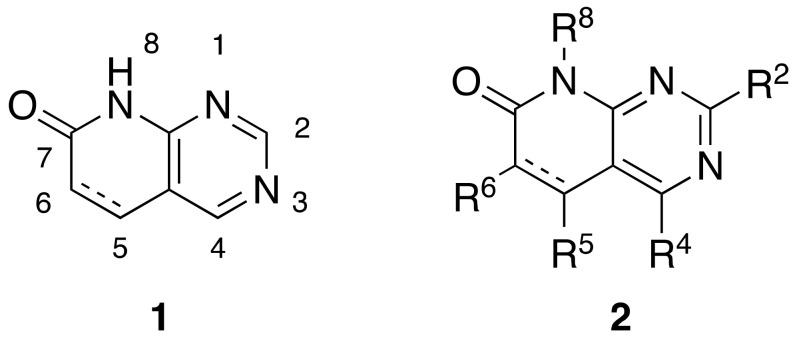
Pyrido[2,3-*d*]pyrimidin-7(8*H*)-ones **1** and diversity centers of such a scaffold (**2**).

Pyrido[2,3-*d*]pyrimidin-7(8*H*)-ones **1** are *ortho*-fused bicyclic heterocycles consisting of pyridone and pyrimidine rings. This kind of structure presents up to five diversity centers (R^2^, R^4^, R^5^, R^6^, and R^8^) and two possible degrees of unsaturation between C5 and C6 (Figure 1). Functionalized pyrido[2,3-*d*]pyrimidines **2** are considered *privileged heterocyclic scaffolds* for drug discovery due to their well-known activity as tyrosine kinase inhibitors (TKI) [5,6]. In this context, our group has previously synthesized multiple compounds with activities in the nanomolar range as BCR inhibitors for B lymphoid malignancies or DDR2 inhibitors for the treatment of lung cancer, among others [7].

**Figure 2:**
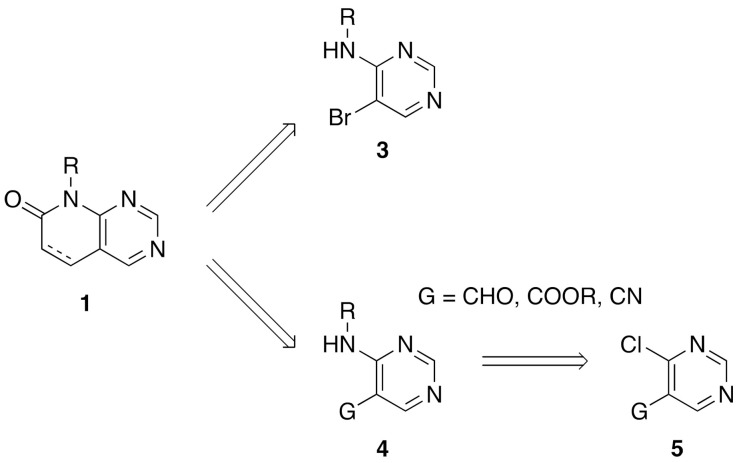
Synthetic approaches for pyrido[2,3-*d*]pyrimidin-7(8*H*)-ones **1** from a preformed pyrimidine ring.

From a synthetic point of view, most of the research groups currently working with such a scaffold construct the molecule either from a preformed pyrimidine or from a preformed pyridone ring. In the first strategy, two approaches have been described to synthesize a pyrido[2,3-*d*]pyrimidin-7(8*H*)-one **1**: starting from an adequately substituted 4-amino-5-bromopyrimidine **3** or starting from an *N*-substituted pyrimidine-4-amine **4**, which contains a carbon functional group G (CHO, COOR, or CN) at position C5 of the pyrimidine ring. This latter compound is usually obtained from the corresponding 4-chloro-substituted pyrimidine **5** (Figure 2) [8].

One of the major drawbacks of these two synthetic approaches is the difficulty of introducing substituents at the C4 position of the final pyrido[2,3-*d*]pyrimidin-7(8*H*)-one **1** either because they could be involved in a possible secondary reaction during the formation of the pyridone ring or because they can even preclude the construction of such a ring due to the steric hindrance that such a substituent could produce. These difficulties have reduced the chemical diversity of substituents present at C4 of the resulting pyridopyrimidine to hydrogen, amino, or short alkyl groups. Only the introduction of a chlorine atom at the starting pyrimidine **4** allows the construction of the corresponding 4-chloro-substituted pyrido[2,3-*d*]pyrimidin-7(8*H*)-one **1** that could allow the further introduction of diversity at such a position [9].

**Figure 3:**
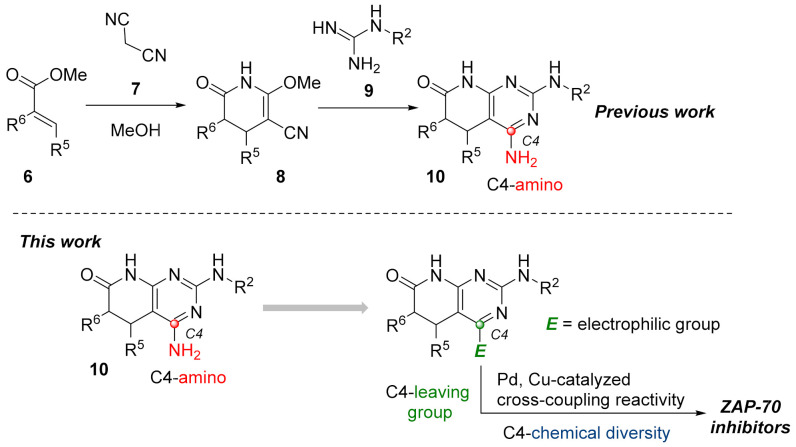
Synthetic approach for pyrido[2,3-*d*]pyrimidin-7(8*H*)-ones (**10**) from a preformed pyridone ring (**left**). The working hypothesis developed in the present work (**right**).

As for the second strategy, the construction of structures **2** from a pyridone ring, there are clearly fewer protocols and references, but our group has large experience in the synthesis of 4-amino-5,6-dihydropyrido[2,3-*d*]pyrimidin-7-(8*H*)-ones (**10**) by cyclization of guanidine systems like **9** with 2-methoxy-6-oxo-1,4,5,6-tetrahydropyridine-3-carbonitriles (**8**), which in turn are obtained by means of a Michael-type reaction between malononitrile (**7**) and an α,β-unsaturated ester of general structure **6** in NaOMe/MeOH media (Figure 3 Left). We also described a protocol to obtain 4-oxo-5,6-dihydropyrido[2,3-*d*]pyrimidin-7-(8*H*)-ones via the corresponding Michael adduct between an α,β-unsaturated ester **6** and methyl cyanoacetate [10].

The accessible chemical diversity at the C4 position of pyrido[2,3-*d*]pyrimidin-7-(8*H*)-ones using the preceding synthetic approaches is certainly limited as we showed in a recent review [11]. Depending on the degree of unsaturation between C5 and C6, the most described C4 substituent differs, being hydrogen (79% of reported structures) and carbon substituents (18%) when having a double bond, and oxygen (mainly as a carbonyl group, 63%) or alkyl groups (26%, mainly methyl) when having a C5-C6 single bond. Most of the molecules containing an amino group in the C4 position have been described by our research group using the strategy included in the left of Figure 3 or similar.

Such limited chemical diversity and the need to explore new tyrosine kinase targets taking advantage of our methodology impelled us to study the introduction of new substituents at C4 to evaluate the potential biological activity of the resulting derivatives on ZAP-70. Hence, and given the great impact that transition metal-catalyzed coupling reactivity have demonstrated in the synthesis of pharmaceuticals [12], we sought the introduction of an electrophilic manifold at the C4 position capable of engaging in either Pd and/or Cu cross-coupling reactions as a key point to rapidly enlarge the number of possible substitutions at such a position (Figure 3 right).

## 2. Results and Discussion

### 2.1. Chemistry

**Figure 4:**
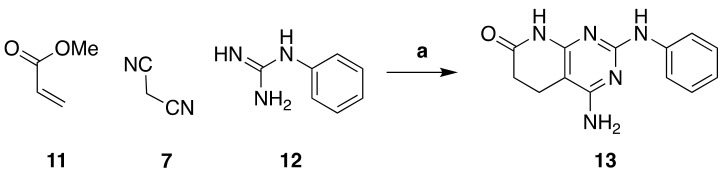
Synthesis of 4-amino-2-(phenylamino)-5,6-dihydropyrido[2,3-*d*]pyrimidin-7(8*H*)-one (**13**). Reaction conditions (a): MW (10 min, 140 °C), dry methanol.

One of our objectives was to obtain a chemical library of pyrido[2,3-*d*]pyrimidin-7(8H)-ones with a wide range of substituents at the C4 position, starting from a common structure. To do so, we synthesized 4-amino-2-(phenylamino)-5,6-dihydropyrido[2,3-*d*]pyrimidin-7(8*H*)-one (**13**) in a one-pot microwave-assisted reaction between methyl acrylate (**11**), malononitrile (**7**)**,** and phenyl guanidine (**12**) (Figure 4), with yields ranging from 65–70%.

**Figure 5:**
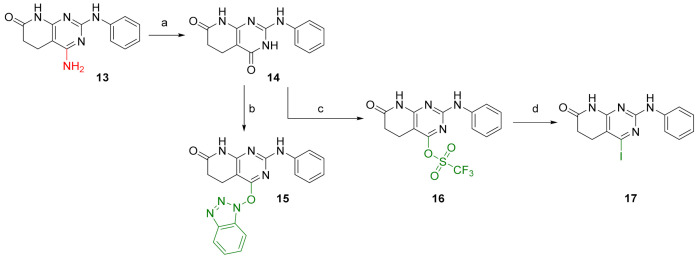
Synthesis of substrates for C4 decoration. Reaction conditions (a): *t*-BuONO, H_2_O:DMF (1:5), MW (10 min, 65 °C). (b): BOP, DBU, ACN, 2 days RT. (c): (CF_3_SO_2_)_2_O, dry pyridine, 30 min RT. (d): dry NaI, CH_3_COCl, dry ACN, MW (5 h, 80 °C).

To enable the presence of an electrophilic leaving group on C4, the diazotization of **13** was performed, followed by trapping with water, to lead the 2-(phenylamino)-5,6-dihydropyrido[2,3-*d*]pyrimidine-4,7(3*H*,8*H*)-dione (**14**) in almost a quantitative yield (Figure 5). We considered that the tautomeric enol form of the C4-oxo group of **14** would be a good entry point. Although compound **14** can also be obtained directly in a one-pot reaction by using methyl cyanoacetate, the overall yield is lower than the two-step reaction via **13**.

Once **14** was obtained, it was possible to introduce either a benzotriazole group (BOP) to afford 4-((1*H*-benzo[*d*][1,2,3]triazol-1-yl)oxy)-2-(phenylamino)-5,6-dihydropyrido[2,3-*d*]pyrimidin-7(8*H*)-one (**15**) or a triflate group (OTf), affording 7-oxo-2-(phenylamino)-5,6,7,8-tetrahydropyrido[2,3-*d*]pyrimidin-4-yl trifluoromethanesulfonate (**16**), with both reactions having yields above 90% (Figure 5). Finally, an iodine atom can also be introduced via nucleophilic substitution of the triflate group with NaI. In this way, 4-iodo-2-(phenylamino)-5,6-dihydropyrido[2,3-*d*]pyrimidin-7(8*H*)-one (**17**) can be obtained in a 95% yield. On the basis of these structures, holding diverse leaving groups, we were able to synthesize, describe, characterize, and test the biological activity of several new compounds.

**Figure 6:**
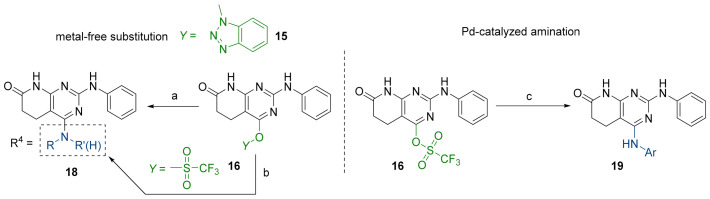
Synthesis of C4 *N*-alkyl (18) and *N*-aryl (19) derivatives from structures 15 and 16. Reaction conditions (a): corresponding amine, ACN, MW (6 h, 140 °C). (b): corresponding amine, ACN, 8–16 h, 100 °C. (c): corresponding aniline, Cs_2_CO_3_, Pd(OAc)_2_, Xphos, dry toluene, O/N, 100 °C.

We were therefore interested in the introduction of the largest possible variety of substituents at the C4 position in order to study the potential biological activity of these compounds. First, we studied the *N*-alkyl and *N*-aryl substituents (Figure 6). There are many reports on how to substitute a benzotriazole group for primary or secondary amines [13]. In this work, we observed that the completion ratio and the yields of the reaction logically differ depending on the nucleophilic capacity of the amine. For this reason, we employed a triflate group not only to carry out a Buchwald–Hartwig cross-coupling reaction with anilines, less nucleophilic than the corresponding aliphatic amines (Figure 6 Right), but to improve both the completion ratio and yields of the substitution reactions with the different amine groups also in a metal-free context (Figure 6 left) [14,15].

Products with *N*-alkyl bonds obtained from structure **15** were synthesized under microwave irradiation conditions while products with the same bond obtained from structure **16** showed both higher yields and purity under conventional heating conditions. This fact may refer to a possible or partial decomposition of the OTf group under such aggressive conditions. The structures of the obtained products and their respective yields are shown in Table 1.

Regarding the synthesis of compounds with *N*-aryl bonds at the C4 position, we used the Buchwald–Hartwig cross-coupling reaction on compound **16**. In particular, the variant of this reaction that uses OTf as a leaving group for the *N*-arylation [16] was demonstrated to be quite efficient in providing yields of the corresponding amines that oscillate between 72 and 97% (Table 2). The full account of our 4-arylamino-2-(phenylamino)-5,6-dihydropyrido[2,3-*d*]pyrimidin-7(8*H*)-ones (**19**) synthesized by direct palladium-catalyzed coupling of aryl triflates and anilines is included in Table 2.

Unfortunately, when we explored *O*-Aryl and *S*-aryl substituents using the same cross-coupling conditions described in the right of Figure 6 that were very effective for the *N*-aryl substituents, we observed the recovery of the starting material **16.**

**Figure 7:**
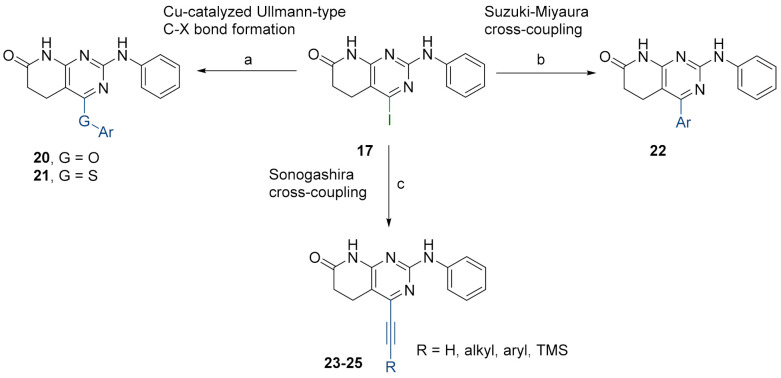
Synthesis of C4-substituted *O*-aryl **20**, *S*-aryl **21**, aryl **22**, alkylethynyl **23**, ethynyl **24**, and arylethinyl **25-**substituted derivatives from structure **17**. Reaction conditions (a): corresponding phenol or thiophenol, K_3_PO_4_, CuI, 2-picolinic acid, dry DMSO, O/N, 80 °C. (b): corresponding boronic acid, Cs_2_CO_3_, Pd(PPh_3_)_4_, deoxygenated mixture of 1,4-dioxane/water (10:1), O/N, 90 °C. (c): Sonogashira-type reactions; see the detailed reaction conditions in Figure 8.

Then, we anticipated that substituting the OTf group by an iodine atom, substrate **17**, could have a beneficial effect on the reactivity of the pyrido[2,3-*d*]pyrimidine and it might help us to achieve the O-arylated or S-arylated products by means of catalytic Ullmann-type reactions. Besides, the presence of the iodine group can also open the door to explore other cross-couplings like Suzuki–Miyaura or Sonogashira reactions at the very same position (Figure 7).

Buchwald et al. [17] described the synthesis of diaryl ethers from aryl iodides or bromides upon the *O*-arylation of phenols under mild conditions using CuI in DMSO and K_3_PO_4_ using picolinic acid as ligand. Such a reaction allows the presence of a wide range of functional groups in the phenol ring, while being also effective with bulky groups.

Table 3 lists the resulting structures obtained with this method.

The best conditions found for the Suzuki arylation of compound **17** to afford 4-aryl-2-(phenylamino)-5,6-dihydropyrido[2,3-*d*]pyrimidin-7(8*H*)-ones (**22**) included the use of Cs_2_CO_3_ as a base, Pd(PPh_3_)_4_ as the catalyst, and a deoxygenated mixture of 1,4-dioxane/water (10:1) as a solvent. The resulting structures are shown in Table 4.

**Figure 8:**
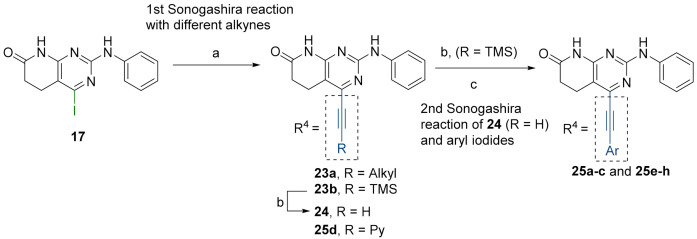
(a): Corresponding alkyne (excess reagent), CuI, PdCl_2_(PPh_3_)_2_, Et_3_N, O/N, 65 °C. (b): 1M TBAF/THF, 3 h, RT. (c): Corresponding iodoaryl (excess reagent), CuI, PdCl_2_(PPh_3_)_2_, Et_3_N, 2 days, 65 °C.

Finally, we explored the possibility of introducing a triple bond at the C4 position because they play an important functional moiety in a wide range of biologically active compounds [18]. With the aim of lending the Sonogashira reaction a higher degree of modularity, we considered that it would be a good idea to gain access to a common terminal alkyne intermediate of type of **24**. In turn, this approach would allow the generation of a wider variety of structures just by carrying out a second Sonogashira coupling with **24** and different aryl iodides, which are generally more accessible and cheaper than ethynylaryl compounds. On this basis, we synthesized the 2-(phenylamino)-4-((trimethylsilyl)ethynyl)-5,6-dihydropyrido[2,3-*d*]pyrimidin-7(8*H*)-one (**23b**) and proceeded to the efficient deprotection of the trimethylsilyl group using a 1 M TBAF/THF solution [19]. Once the 4-ethynyl-2-(phenylamino)-5,6-dihydropyrido[2,3-*d*]pyrimidin-7(8*H*)-one (**24**) was obtained, a second Sonogashira reaction was accomplished, but this time using the pyrido[2,3-*d*]pyrimidine as a limiting reagent and the aryl iodides as an excess reagent (Figure 8).

The structures of the obtained products, the yields of each reaction, and the conditions that were used in each reaction are shown in Table 5.

### 2.2. Biological Activity

As previously stated, our work aimed to obtain relevant information about the potential biological activity of new 4-substituted-2-(phenylamino)-5,6-dihydropyrido[2,3-*d*]pyrimidin-7(8*H*)-ones, which have never been synthesized before. Our group tested the biological activity of several pyrido[2,3-*d*]pyrimidin-7(8*H*)-ones with substituents at C2, C5, and/or C6 with an amino or carbonyl group at the C4 position as TKI. In our experience, most of these compounds show high biological activity against tyrosine kinases involved in solid tumors, by targeting overexpressed proteins in lung [20,21] or pancreatic cancer (unpublished results).

This is a common trend, with other pyrido[2,3-*d*]pyrimidin-7(8*H*)-ones **1** described previously in the literature bearing a hydrogen atom or alkyl groups at position C4. A search carried out in SciFinder [22,23] with structure **1** revealed that there are only 16 references that include the term ZAP-70, with most of them being patents. Checking the 110 structures **1** included in these references, 106 structures present a hydrogen atom at the C4 position and 4 of them contain a methyl group. It is important to emphasize that, apparently, none of these structures show biological activity against ZAP-70.

Convergently, when, as a part of our project on ZAP-70 inhibitors, we sent to Reaction Biology (https://www.reactionbiology.com/) (accessed on 10 November 2021) to determine the kinase inhibition profile, by measuring residual activity values at a concentration of 10 µM of the test compound in duplicate in front of a set of selected kinases, the group of pyrido[2,3-*d*]pyrimidin-7(8*H*)-ones presenting amino or carbonyl groups at C4 showed no significant activity against this biological target.

**Figure 9:**
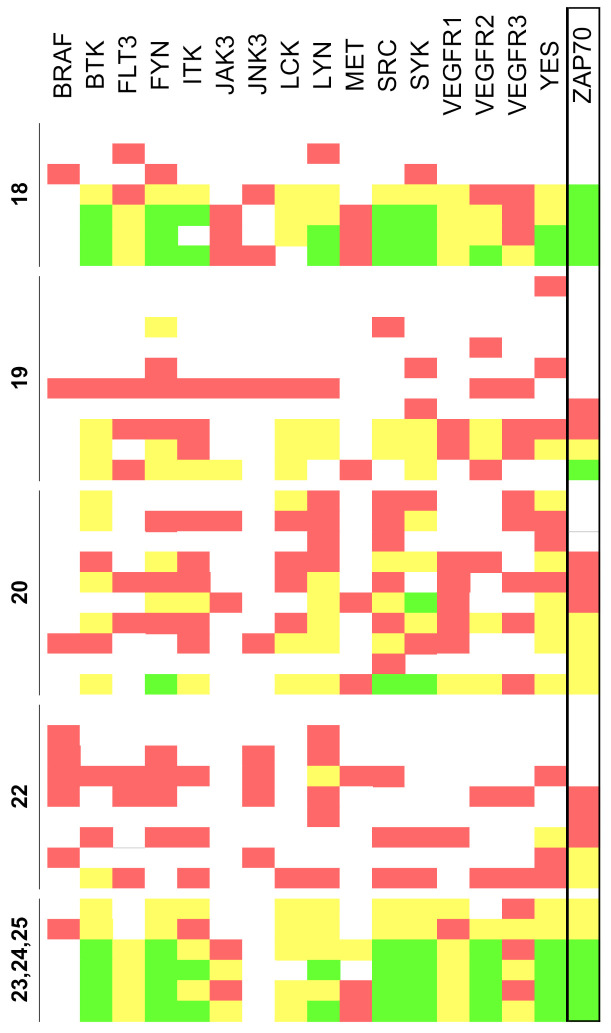
Residual activity percentages at a concentration of 10 µM of the tested compounds (<20% in green, 20–60% yellow, 60–80% red, and >80% white). Results are classified according to the corresponding family of compounds (**18**, **19**, **20**, **22** and **23**, **24**, **25**).

However, the extension of the chemical space achieved by the synthetic methodology described in this study offers the possibility to broaden the biological profile of pyrido[2,3-*d*]pyrimidin-7(8*H*)-ones to hitherto unexplored regions. Thus, two successive iterations modifying the chemical diversity at the C4 position allowed us to obtain compounds with high biological activity against ZAP-70 and SYK, key tyrosine kinases involved in lymphoma. In particular, some of the compounds synthesized exhibited inhibitory activities higher than 90% for ZAP-70 (expressed as residual kinase activity, Figure 9).

**Figure 10:**
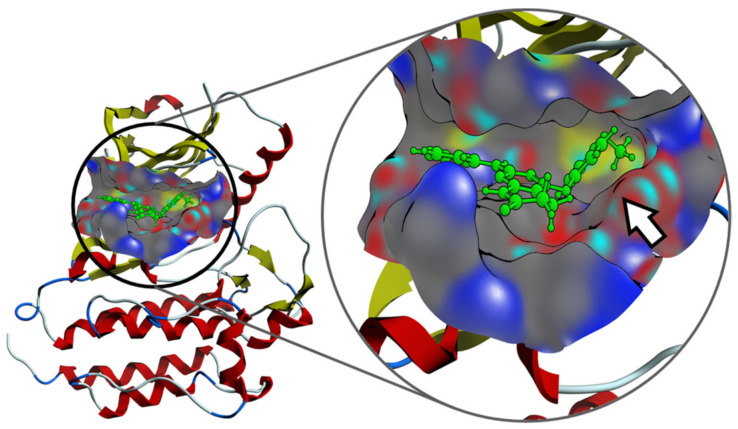
Interaction mechanism between one of the compounds with C4 substitution and the pocket of the ZAP-70 protein, predicted by molecular docking. The presence of the C4 substituent clearly allows the molecule to improve the binding on the active site (white arrow).

Interestingly, the experimental results provide evidence that the introduction of substituents that introduce at the C4 position an sp3 oxygen or nitrogen atom that causes the resulting substituent to be nonlinear, as it occurs in compounds **19** and **20** derivatives, do not contribute to the activity against ZAP-70. To rationalize these findings, molecular docking studies were conducted considering the compounds listed in this study and ZAP-70 protein (see the Supplementary Materials for computational details). Results suggested that active pyrido[2,3-*d*]pyrimidin-7(8*H*)-ones can fit the ATP-binding site of the tyrosine kinase domain of ZAP-70 and the C4 substituent would help to reach the pocket defined by Lys369 (involved in the salt bridge characteristic in the active kinase structure) and Asp479, which belongs to the DFG motif, abutting the activation loop (Figure 10).

## 3. Materials and Methods

### 3.1. General Considerations

All solvents and chemicals were reagent grade. Unless otherwise mentioned, all solvents and chemicals were purchased from commercial suppliers (Sigma Aldrich, Fluorochem, Apollo scientific, Activate scientific, Alfa Aesar, and Enamine) and used without further purification. ^1^H, ^13^C, and ^19^F NMR spectra were recorded on a Varian 400-MR spectrometer (^1^H NMR at 400 MHz, ^13^C NMR at 100.5 MHz, and ^19^F NMR at 376 MHz). Chemical shifts were reported in parts per million (δ) and are referenced to the residual signal of the solvent DMSO-*d_6_* 2.50 ppm or tetramethylsilane (TMS) 0 ppm in ^1^H NMR spectra and to the residual signal of the solvent DMSO-*d_6_* 39.5 ppm in ^13^C NMR. Coupling constants are reported in Hertz (Hz). Standard and peak multiplicities are designed as follows: s, singlet; d, doublet; dd, doublet of doublets; t, triplet; q, quartet; p, quintet; br, broad signal. “*” means interchangeable assignment. IR spectra were recorded in a Thermo Scientific Nicolet iS10 FTIR spectrophotometer with Smart iTr. Wavenumbers (ν) are reported in cm^−1^. MS data (m/z (%), EI, 70 eV) were obtained by using an Agilent Technologies 5975. HRMS data were obtained by using a X500B (SCIEX) QTOF high-resolution mass spectrometer (ESI mode). Elemental microanalyses were obtained on a EuroVector Instruments Euro EA 3000 elemental analyzer. All microwave irradiation experiments were carried out in a dedicated Biotage-Initiator microwave apparatus, operating at a frequency of 2.45 GHz with continuous irradiation power from 0 to 400 W with the utilization of the standard absorbance level of 400 W maximum power. Reactions were carried out in glass tubes, sealed with aluminum/Teflon crimp tops, which can be exposed up to 250 °C and 20 bar internal pressure. Temperature was measured with an IR sensor on the outer surface of the process vial. After the irradiation period, the reaction vessel was cooled rapidly (60–120 s) to ambient temperature by air-jet cooling. Synthesis and spectroscopic data for all compounds are described in the Supplementary Materials.

### 3.2. General Procedure for the Synthesis of Structures ***18***

The intermediate 4-((1*H*-benzo[*d*][1-3]triazol-1-yl)oxy)-2-(phenylamino)-4,5,6,8-tetrahydropyrido[2,3-*d*]pyrimidin-7(8*H*)-one (**15**) (100 mg, 0.267 mmol) was suspended in ACN (20 mL), 3 equivalents of the corresponding amine (0.803 mmol) were added to the suspension, and the mixture was heated at 140 °C under microwave irradiation for 6 h. Then, water was added to the residue and the solid was collected by filtration and washed with water, ethanol, and diethyl ether in order to afford the corresponding spectroscopically pure product.

**4-((3-Morpholinopropyl)amino)-2-(phenylamino)-5,6-dihydropyrido[2,3-*d*]pyrimidin-7(8*H*)-one (18a)**

As above but carried out by using 4-((1*H*-benzo[*d*][1-3]triazol-1-yl)oxy)-2-(phenylamino)-4,5,6,8-tetrahydropyrido[2,3-*d*]pyrimidin-7(8*H*)-one (**15**) (100 mg, 0.267 mmol) and *N*-(3-aminopropyl)morpholine (117 μL, 0.803 mmol). The mixture was heated at 140 °C for 6h under microwave irradiation. In total, 54.1 mg (0.141 mmol, 54%) of spectroscopically pure 4-((3-morpholinopropyl)amino)-2-(phenylamino)-5,6-dihydropyrido[2,3-*d*]pyrimidin-7(8*H*)-one (**18a**) were obtained as an orangish solid. mp: >250 °C. ^1^H-NMR (400 MHz, DMSO-*d*_6_) δ (ppm): 9.96 (s, 1H, N8-H), 8.73 (s, 1H, N9-H), 7.85–7.79 (m, 2H, C11-H), 7.22–7.16 (m, 2H, C12-H), 6.88–6.81 (m, 1H, C13-H), 6.67 (t, *J* = 5.5 Hz, 1H, N14-H), 3.56 (t, *J* = 4.6 Hz, 4H, C20-H), 3.41 (q, *J* = 6.7 Hz, 2H, C15-H), 2.60–2.51 (m, 4H, C5-H, C6-H), 2.40–2.29 (m, 6H, C19-H, C17-H), 1.74 (p, *J* = 7.1 Hz, 2H, C16-H). ^13^C-NMR (100.5 MHz, DMSO-*d*_6_) δ (ppm): 171.1 (C7), 160.1 (C4), 157.9 (C2), 155.5 (C8a), 141.5 (C10), 128.2 (C12), 120.1 (C13), 118.2 (C11), 85.9 (C4a), 66.2 (C20), 56.4 (C17), 53.4 (C19), 39.1 (C15), 30.3 (C6), 26.0 (C16), 17.1 (C5). IR (KBr) ν (cm^−1^): 3429, 3287, 3204, 2917, 2861, 1635, 1601, 1579, 1548, 1444, 1375, 1245, 1119, 752. OEA calculated for C_20_H_26_N_6_O_2_: C: 62.81%, H: 6.85%, N: 21.97%; found: C: 62.46%, H: 6.67%, N: 21.57%. HRMS (APCI-FIA-TOF) (m/z) calculated for C_20_H_26_N_6_O_2_: 382.2117, [M]^+^, found: 383.2187, [M+H]^+^.

### 3.3. General Procedure for the Synthesis of Structures ***19***

Intermediate 7-oxo-2-(phenylamino)-5,6,7,8-tetrahydropyrido[2,3-*d*]pyrimidin-4-yl trifluoromethanesulfonate (**16**) (1 eq), cesium carbonate (1.2 eq), palladium(II)acetate (0.1 eq), XPhos (0.15 eq), and the corresponding aniline (1.1 eq) were introduced under argon atmosphere into a Schlenk tube. After that, anhydrous toluene (1 mL) was added and the mixture was heated overnight at 100 °C. Then, water (30 mL) was added to the residue and the solid was collected by filtration and washed with water, ethanol, and cyclohexane in order to afford the spectroscopically pure product.

**2-(Phenylamino)-4-((3,4,5-trimethoxyphenyl)amino)-5,6-dihydropyrido[2,3-*d*]pyrimidin-7(8*H*)-one (19a)**

As above but carried out by using 7-oxo-2-(phenylamino)-5,6,7,8-tetrahydropyrido[2,3-*d*]pyrimidin-4-yl trifluoromethanesulfonate (**16**) (77.66 mg, 0.20 mmol) and 3,4,5-trimethoxyaniline (40.30 mg, 0.22 mmol). In total, 79.2 mg (0.187 mmol, 94%) of spectroscopically pure 2-(phenylamino)-4-((3,4,5-trimethoxyphenyl)amino)-5,6-dihydropyrido[2,3-*d*]pyrimidin-7(8*H*)-one (**19a**) were obtained as a pale brown solid. mp: >250 °C. ^1^H-NMR (400 MHz, DMSO-*d*_6_) δ (ppm): 10.17 (s, 1H, N8-H), 8.87 (s, 1H, N9-H), 8.29 (s, 1H, N14-H), 7.75–7.72 (m, 2H, C11-H), 7.13–7.09 (m, 2H, C12-H), 6.90 (s, 2H, C16-H), 6.86–6.82 (m, 1H, C13-H), 3.69 (s, 6H, C19-H), 3.65 (s, 3H, C20-H), 2.77 (dd, *J* = 8.3, 7.0 Hz, 2H, C5-H), 2.56 (dd, *J* = 8.2, 7.0 Hz, 2H, C6-H). ^13^C-NMR (100.5 MHz, DMSO-*d*_6_) δ (ppm): 171.2 (C7), 158.1 (C4), 157.5 (C2), 157.0 (C8a), 152.4 (C17), 141.0 (C10), 135.9 (C15), 133.1 (C18), 128.1 (C12), 120.5 (C13), 118.6 (C11), 100.2 (C16), 87.9 (C4a), 60.1 (C20), 55.6 (C19), 30.3 (C6), 17.3 (C5). IR (KBr) ν (cm^−1^): 3350, 3288, 3206, 3138, 2959, 2927, 2851, 1677, 1601, 1580, 1503, 1443, 1239, 1221, 1129, 996, 745. HRMS (APCI-FIA-TOF) (m/z) calculated for C_22_H_23_N_5_O_4_: 421.1750, [M]^+^, found: 422.1820, [M+H]^+^.

### 3.4. General Procedure for the Synthesis of Structures ***20*** and ***21***

Intermediate 4-iodo-2-(phenylamino)-5,6-dihydropyrido[2,3-*d*]pyrimidin-7(8*H*)-one (**17**) (1 eq), tripotassium phosphate (2 eq), copper(I) iodide (0.05 eq), 2-picolinic acid (0.1 eq), and the corresponding phenol or thiophenol (1.2 eq) were introduced under argon atmosphere into a Schlenk tube. After that, anhydrous DMSO (1.4 mL) was added and the mixture was heated overnight at 80 °C. Then, water (30 mL) was added to the residue and the solid was collected by filtration and washed with more water, ethanol, and diethyl ether to afford the spectroscopically pure product.

**4-Phenoxy-2-(phenylamino)-5,6-dihydropyrido[2,3-*d*]pyrimidin-7(8*H*)-one (20a)**

As above but carried out by using 4-iodo-2-(phenylamino)-5,6-dihydropyrido[2,3-*d*]pyrimidin-7(8*H*)-one (**17**) (73.23 mg, 0.20 mmol) and phenol (22.59 mg, 0,24 mmol). The mixture reaction was heated at 80 °C for 48h. In total, 41.7 mg (0.125 mmol, 64%) of spectroscopically pure 4-phenoxy-2-(phenylamino)-5,6-dihydropyrido[2,3-*d*]pyrimidin-7(8*H*)-one (**20a**) were obtained as a greyish solid. mp: >250 °C. ^1^H-NMR (400 MHz, DMSO-*d*_6_) δ (ppm): 10.60 (s, 1H, N8-H), 9.20 (s, 1H, N9-H), 7.53–7.41 (m, 4H, C11-H, C17-H), 7.33–7.24 (m, 1H, C18-H), 7.26–7.17 (m, 2H, C16-H), 7.06–6.98 (m, 2H, C12-H), 6.85–6.76 (m, 1H, C13-H), 2.85 (dd, *J* = 8.2, 7.1 Hz, 2H, C5-H), 2.61 (dd, *J* = 8.2, 7.1 Hz, 2H, C6-H). ^13^C-NMR (100.5 MHz, DMSO-*d*_6_) δ (ppm): 171.5 (C7), 165.9 (C4*), 159.6 (C8a*), 157.4 (C2), 153.0 (C15), 140.4 (C10), 129.5 (C17), 128.0 (C12), 125.0 (C18), 121.9 (C16), 120.8 (C13), 118.2 (C11), 89.9 (C4a), 30.2 (C6), 16.4 (C5). IR (ATR) ν (cm^−1^): 3284, 3203, 3138, 2968, 1683, 1618, 1577, 1549, 1441, 1401, 1349, 1236, 1199, 750, 688. HRMS (APCI-FIA-TOF) (m/z) calculated for C_19_H_16_N_4_O_2_: 332.1273, [M]^+^, found: 333.1345, [M+H]^+^.

### 3.5. General Procedure for the Synthesis of Structures ***22***

Intermediate 4-iodo-2-(phenylamino)-5,6-dihydropyrido[2,3-*d*]pyrimidin-7(8*H*)-one (**17**) (1 eq), cesium carbonate (2.5 eq), tetrakis(triphenylphosphine)palladium(0) (2% molar), and the corresponding boronic acid (1.4 eq) were introduced under argon atmosphere into a Schlenk tube. After that, a deoxygenated mixture of 1,4-dioxane/water (10:1) (1.5 mL) was added and the resultant reaction mixture was heated overnight at 90 °C. Then, water was added (20 mL) to the residue and the solid appeared was collected by filtration and washed with more water, ethanol, and diethyl ether to afford the spectroscopically pure product.

**4-Phenyl-2-(phenylamino)-5,6-dihydropyrido[2,3-*d*]pyrimidin-7(8*H*)-one (22a)**

As above but carried out by using 4-iodo-2-(phenylamino)-5,6-dihydropyrido[2,3-*d*]pyrimidin-7(8*H*)-one (**17**) (73.23 mg, 0.20 mmol) and phenylboronic acid (34.14 mg, 0.28 mmol). 37.4 mg (60%, 0.118 mmol) of spectroscopically pure 4-phenyl-2-(phenylamino)-5,6-dihydropyrido[2,3-*d*]pyrimidin-7(8*H*)-one (**22a**) were obtained as a dark grey solid. mp: >250 °C. ^1^H-NMR (400 MHz, DMSO-*d*_6_) δ (ppm): 10.72 (s, 1H, N8-H), 9.41 (s, 1H, N9-H), 7.90–7.81 (m, 2H, C11-H), 7.65–7.59 (m, 2H, C15-H), 7.54–7.46 (m, 3H, C16-H, C17-H), 7.26–7.20 (m, 2H, C12-H), 6.93–6.87 (m, 1H, C13-H), 2.85 (dd, *J* = 8.4, 6.6 Hz, 2H, C5-H), 2.53–2.50 (m, 2H, C6-H). ^13^C-NMR (100.5 MHz, DMSO-*d*_6_) δ (ppm): 171.6 (C7), 163.2 (C4), 158.9 (C8a), 158.1 (C2), 140.9 (C10), 137.7 (C14), 129.0 (C17), 128.6 (C15), 128.3 (C12), 128.1 (C16), 120.8 (C13), 118.5 (C11), 103.4 (C4a), 30.7 (C6), 20.5 (C5). IR (ATR) ν (cm^−1^): 3281, 3198, 3141, 3047, 2904, 1686, 1592, 1544, 1435, 1343, 1303, 1217, 815, 749, 689. HRMS (APCI-FIA-TOF) (m/z) calculated for C_19_H_16_N_4_O: 316.1324, [M]^+^, found: 317.1392, [M+H]^+^.

### 3.6. General Procedure for the Synthesis of Structures ***23***

Intermediate 4-iodo-2-(phenylamino)-5,6-dihydropyrido[2,3-*d*]pyrimidin-7(8*H*)-one (**17**) (1 eq), bis(triphenylphosphine)palladium dichloride (1.5% molar), copper(I) iodide (3% molar), and the corresponding alkyne (1.5 eq) were introduced under argon atmosphere into a Schlenk tube. Then, triethylamine (1.5 mL) was added and the Schlenk tube was sealed. The resulting reaction mixture was heated at 65 °C overnight under vigorous stirring. The solvent was evaporated in vacuo and the resulting mixture was suspended in water (50 mL) and the spectroscopically pure product was collected by filtration after being washed with more water, ethanol, and diethyl ether.

**2-(Phenylamino)-4-((trimethylsilyl)ethynyl)-5,6-dihydropyrido[2,3-*d*]pyrimidin-7(8*H*)-one (23b)**

As above but carried out by using 4-iodo-2-(phenylamino)-5,6-dihydropyrido[2,3-*d*]pyrimidin-7(8*H*)-one (**17**) (300.0 mg, 0.82 mmol) and ethynyltrimethylsilane (170 μL, 1.23 mmol). In total, 218.3 mg (0.649 mmol, 79%) of spectroscopically pure 2-(phenylamino)-4-((trimethylsilyl)ethynyl)-5,6-dihydropyrido[2,3-*d*]pyrimidin-7(8*H*)-one (**23b**) were obtained as a pale green solid. mp: >250 °C. ^1^H-NMR (400 MHz, DMSO-*d*_6_) δ (ppm): 10.80 (s, 1H, N8-H), 9.51 (s, 1H, N9-H), 7.83–7.71 (m, 2H, C11-H), 7.34–7.16 (m, 2H, C12-H), 7.01–6.87 (m, 1H, C13-H), 2.86 (dd, *J* = 8.3, 6.9 Hz, 2H, C5-H), 2.59 (dd, *J* = 8.3, 6.9 Hz, 2H, C6-H), 0.26 (s, 9H, C17-H). ^13^C-NMR (100.5 MHz, DMSO-*d*_6_) δ (ppm): 171.4 (C7), 158.5 (C8a), 158.2 (C2), 146.3 (C4), 140.5 (C10), 128.4 (C12), 121.1 (C13), 118.5 (C11), 107.9 (C4a), 100.7 (C15), 100.5 (C14), 30.1 (C6), 19.9 (C5), -0.5 (C17). IR (ATR) ν (cm^−1^): 3283, 3144, 2957, 1685, 1592, 1567, 1547, 1500, 1437, 1335, 1210, 1063, 1020, 839, 744. HRMS (APCI-FIA-TOF) (m/z) calculated for C_18_H_20_N_4_OSi: 336.1406, [M]^+^, found: 337.1475, [M+H]^+^.

**Kinase inhibition profile.** The kinase inhibition profile of compounds was evaluated at Reaction Biology (https://www.reactionbiology.com/) (accessed on 10 November 2021) by measuring residual activity values at a concentration of 10 µM of the test compound in singlicate in front of the several kinases including Btk, Lyn, Syk aa1-635, and Zap70 (see the Supplementary Materials for the complete list of targets) using the following protocol: The compounds were dissolved to 1 × 10^−3^ M stock solutions in 100% DMSO. Subsequently, 100 μL of each stock solution were transferred into wells A3-F12 of a microtiter plate (“master plate”). Wells A1-F2 were filled with 100 μL of 100% DMSO as controls. In total, 5 × 10 μL of the master plate were aliquoted into 5 copy plates, which were stored at −20 °C until use. For the testing of each group of up to 8 kinases, one copy plate was used. In the process, 90 μL of H_2_O were added to each well of a copy plate. To minimize precipitation, the H_2_O was added to each well only a few minutes before the transfer of the compound solutions into the assay plates. The plate was shaken thoroughly, resulting in a “compound dilution plate” with a compound concentration of 1 × 10^−4^ M/10 % DMSO. This plate was used for the transfer of 5 μL of compound solution into the assay plates. The final volume of the assay was 50 μL. All compounds were tested at 1 × 10^−5^ M in singlicate. The final DMSO concentration in the reaction cocktails was 1% in all cases. The compound dilution plates were disposed of at the end of each working day.

A radiometric protein kinase assay (^33^PanQinase^®^ Activity Assay) was used for measuring the kinase activity of the corresponding protein kinases. All kinase assays were performed in 96-well FlashPlatesTM from Perkin Elmer (Boston, MA, USA) in a 50 μL reaction volume. The reaction cocktail was pipetted in 4 steps in the following order: 10 μL of non-radioactive ATP solution (in H_2_O); 25 μL of assay buffer/ [γ-33P]-ATP mixture; 5 μL of test sample in 10% DMSO; and 10 μL of enzyme/substrate mixture. The assay for all protein kinases contained 70 mM HEPES-NaOH pH 7.5, 3 mM MgCl_2_, 3 mM MnCl_2_, 3 μM Na-orthovanadate, 1.2 mM DTT, ATP (variable amounts, corresponding to the apparent ATP-Km of the respective kinase), [γ-^33^P]-ATP (approximately 8 × 10^5^ cpm per well), protein kinase (variable amounts), and substrate (variable amounts). The protein kinase reaction cocktails were incubated at 30 °C for 60 min. The reaction was stopped with 50 μL of 2 % (*v*/*v*) H_3_PO_4_, plates were aspirated, and washed two times with 200 μL of 0.9 % (*w*/*v*) NaCl. All assays were performed with a BeckmanCoulter Biomek 2000/SL robotic system. Incorporation of ^33^Pi (counting of “cpm”) was determined with a microplate scintillation counter (Microbeta, Wallac). All protein kinase assays were performed with a BeckmanCoulter Core robotic system.

For each kinase, the median value of the cpm of six wells of column 1 of each assay plate was defined as the “low control” (*n* = 6). This value reflects unspecific binding of radioactivity to the plate in the absence of a protein kinase but in the presence of the substrate. Additionally, for each kinase, the median value of the cpm of six wells of column 2 of each assay plate was taken as the “high control”, i.e., full activity in the absence of any inhibitor (*n* = 6). The difference between the high and low control of each enzyme was taken as 100% activity. As part of the data evaluation the low control of each kinase was subtracted from the high control value as well as from their corresponding “compound values”. The residual activity (in %) for each compound well was calculated by using the following formula:

Res. Activity (%) = 100 × [(signal of compound − low control)/(high control − low control)]

As a parameter for assay quality, the Z´-factor^30^ for the low and high controls of each assay plate (*n* = 8) was used. Reaction Biology´s criterion for repetition of an assay plate is a Z´-factor below 0.4.^31^ Z´-factors did not drop below 0.51, indicating an excellent assay quality.

## 4. Conclusions

This study nicely illustrates the impact that transition-metal-catalyzed coupling reactivity has in the chemical diversification at the C4 position of the 4-substituted pyrido[2,3-*d*]pyrimidin-7(8*H*)-one skeleton. As far as biological activity is concerned, we demonstrate that an adequate 4-substituted pyrido[2,3-*d*]pyrimidin-7(8*H*)-one can drastically increase the biological activity of such a scaffold against some tyrosine kinases that could not be targeted before, including ZAP-70.

## Figures and Tables

**Table 1:** C4-substituted *N-*alkyl pyrido[2,3-*d*]pyrimidines **18a-h**.

Compound	R^4^	Yield (%)	Compound	R^4^	Yield (%)
**18a**	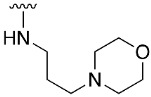	54 ^a^	**18e**	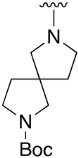	83 ^b^
**18b**	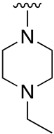	55 ^a^	**18f**	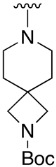	79 ^b^
**18c**	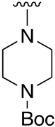	67 ^b^	**18g**	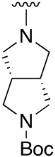	65 ^b^
**18d**	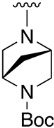	63 ^b^	**18h**	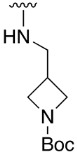	71 ^b^

^a^ Carried out using 4-((1*H*-benzo[*d*][1,2,3]triazol-1-yl)oxy)-2-(phenylamino)-5,6-dihydropyrido[2,3-*d*]pyrimidin-7(8*H*)-one (**15**) as substrate. ^b^ Carried out using 7-oxo-2-(phenylamino)-5,6,7,8-tetrahydropyrido[2,3-*d*]pyrimidin-4-yl trifluoromethanesulfonate (**16**) as substrate.

**Table 2:** C4-substituted *N*-aryl pyrido[2,3-*d*]pyrimidines **19a-k.**

Compound	-NH-Ar	Yield (%)	Compound	-NH-Ar	Yield (%)
**19a**	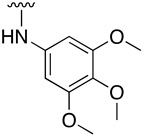	94 ^a^	**19g**	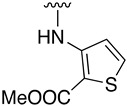	97 ^a^
**19b**	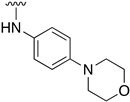	85 ^a^	**19h**	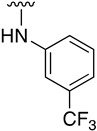	81 ^a^
**19c**	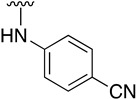	87 ^a^	**19i**	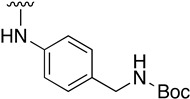	95 ^a^
**19d**	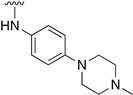	72 ^a^	**19j**	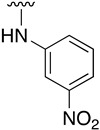	94 ^a^
**19e**	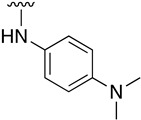	84 ^a^	**19k**	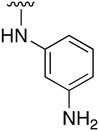	91 ^b^
**19f**	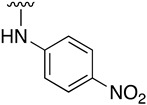	88 ^a^			

^a^ Palladium-catalyzed cross-coupling reaction between **16** and the corresponding aniline. ^b^ Reduction of **19k** using SnCl_2_, HCl_(c)_ in AcOEt.

**Table 3:** C4-substittuted *O*-aryl (**20a-j**) and *S-*aryl (**21a-b)** pyrido[2,3-*d*]pyrimidines.

Compound	-G-Ar	Yield (%)	Compound	-G-Ar	Yield (%)
**20a**	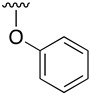	64 ^a^	**20g**	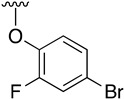	75 ^a^
**20b**	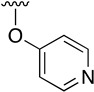	68 ^a^	**20h**	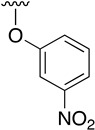	75 ^a^
**20c**	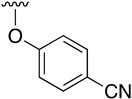	78 ^a^	**20i**	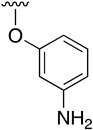	91 ^b^
**20d**	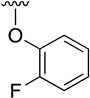	90 ^a^	**20j**	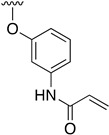	78 ^c^
**20e**	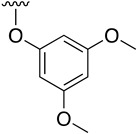	68 ^a^	**21a**	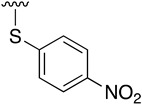	92 ^a^
**20f**	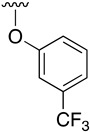	60 ^a^	**21b**	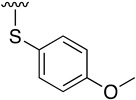	44 ^a^

^a^ Copper-catalyzed cross-coupling between **17** and the corresponding phenol or thiophenol. ^b^ Reduction of **20h** using SnCl_2_, HCl_(c)_ in AcOEt. ^c^ Amidation reaction between **20i** and acryloyl chloride in a mixture of THF:NaHCO_3_ (1:1).

**Table 4:** C4-substituted aryl-substituted derivatives **22a-j** accessible using a Suzuki reaction.

Compound	Ar	Yield (%)	Compound	Ar	Yield (%)
**22a**	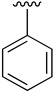	60	**22f**	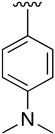	70
**22b**	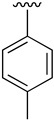	89	**22g**	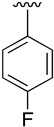	72
**22c**	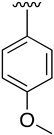	78	**22h**	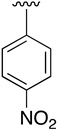	76
**22d**	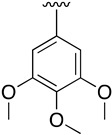	63	**22i**	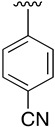	78
**22e**	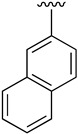	76	**22j**	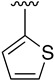	70

**Table 5:** C4-substituted alkynyl derivatives **23a-b**, **24**, and **25a-h** obtained using a Sonogashira cross-coupling reaction.

Compound	R^4^	Yield (%)	Compound	R^4^	Yield (%)
**23a**	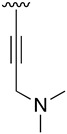	88 ^a^	**25d**	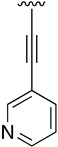	87 ^a^
**23b**	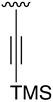	79 ^a^	**25e**	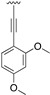	89 ^c^
**24**	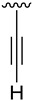	97 ^b^	**25f**	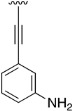	96 ^c^
**25a**	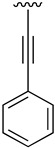	95 ^c^	**25g**	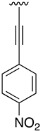	95 ^c^
**25b**	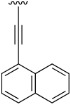	52 ^c^	**25h**	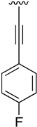	82 ^c^
**25c**	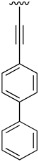	84 ^c^			

^a^ Sonogashira cross-coupling reaction using the alkyne as an excess reagent. ^b^ Deprotection of the trimethylsilyl group using a 1 M TBAF/THF solution. ^c^ Sonogashira cross-coupling between compound **24** and aryl iodides.

## Data Availability

Data is contained within the article and supplementary material.

## Supplementary Materials

The following are available online at https://www.mdpi.com/article/10.3390/ph14121311/s1: Molecular modeling details, synthesis and spectroscopic data for all compounds.
