# Supplementary material for: Expanding the Diversity at the C-4 Position of Pyrido[2,3-d]pyrimidin-7(8H)-ones to Achieve Biological Activity against ZAP-70
Source: Pharmaceuticals (Basel). 2021 Dec 15;14(12):1311. doi: 10.3390/ph14121311 (PMC8703703; doi:10.3390/ph14121311)
Supplement: Supplementary file 1 [file pharmaceuticals-14-01311-s001.zip › pharmaceuticals-1480828-supplementary.pdf]

SUPPLEMENTARY INFORMATION

Expanding the diversity at C-4 position of pyrido[2,3-*d*]pyrimidin-7(8*H*)-ones to achieve biological activity against ZAP-70

Victor Masip [1], Angel Lirio [1], Albert Sánchez-López [1], Ana B. Cuenca [1], Raimon Puig de la Bellacasa [1], Pau Abrisqueta [2], Jordi Teixidó [1], José I. Borrell [1], Albert Gibert [1] and Roger Estrada-Tejedor [1,*]

[1]*Grup de Química Farmacèutica, Institut Químic de Sarrià, Universitat Ramon Llull, Via Augusta, 390, E-08017 Barcelona, Spain. E-mail: roger.estrada@iqs.url.edu*

[2] *Department of Hematology, Hospital Universitari Vall d´Hebron. Experimental Hematology, Vall d'Hebron Institute of Oncology (VHIO), Passeig de la Vall d'Hebron 119-129, E-08035 Barcelona, Spain.*

Table of Contents

	Page
Experimental Procedures	2
NMR Spectra	34
Molecular modelling	91

Experimental Procedures

General considerations

All solvents and chemicals were reagent grade. Unless otherwise mentioned, all solvents and chemicals were purchased from commercial vendors (Sigma Aldrich, Fluorochem, Apollo scientific, Activate scientific, Alfa Aesar and Enamine) and used without further purification. ^{1}H, ^{13}C and ^{19}F NMR spectra were recorded on a Varian 400-MR spectrometer (^{1}H NMR at 400 MHz, ^{13}C NMR at 100.5 MHz and ^{19}F NMR at 376 MHz). Chemical shifts were reported in parts per million (δ) and are referenced to the residual signal of the solvent DMSO-d_6 2.50 ppm or tetramethylsilane (TMS) 0 ppm in ^{1}H NMR spectra and to the residual signal of the solvent DMSO-d_6 39.5 ppm in ^{13}C NMR). Coupling constants are reported in Hertz (Hz). Standard and peak multiplicities are designed as follows: s, singlet; d, doublet; dd, doublet of doublets; t, triplet; q, quartet; p, quintet; br, broad signal. "*" means interchangeable assignment.

IR spectra were recorded in a Thermo Scientific Nicolet iS10 FTIR spectrophotometer with Smart iTr. Wavenumbers (ν) are reported in cm^{-1}.

MS data (m/z (%), EI, 70 eV) were obtained by using an Agilent Technologies 5975. HRMS data were obtained by using a X500B (SCIEX) QTOF high-resolution mass spectrometer (ESI mode).

Elemental microanalyses were obtained on a EuroVector Instruments Euro EA 3000 elemental analyzer.

All microwave irradiation experiments were carried out in a dedicated Biotage-Initiator microwave apparatus, operating at a frequency of 2.45 GHz with continuous irradiation power from 0 to 400 W with utilization of the standard absorbance level of 400 W maximum power. Reactions were carried out in glass tubes, sealed with aluminium/teflon crimp tops, which can be exposed up to 250 °C and 20 bar internal pressure. Temperature was measured with an IR sensor on the outer surface of the process vial. After the irradiation period, the reaction vessel was cooled rapidly (60–120 s) to ambient temperature by air jet cooling.

1.1. Synthesis of starting materials

1.1.1. Synthesis and characterization of 4-amino-2-(phenylamino)-5,6-dihydropyrido[2,3-*d*]pyrimidin-7(8*H*)-one (13)

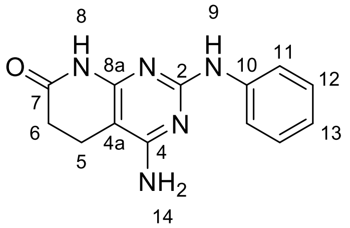

A mixture of *N*-phenylguanidine carbonate (having a $C_7H_9N_3\cdot(H_2CO_3)_{0.69}$ stoichiometry) (1296 mg, 9.8 mmol of *N*-phenylguanidine), methyl methacrylate (0.85 mL, 10.12 mmol) and malononitrile (720 mg, 10.8 mmol) and methanol (20 mL) is sealed in a 20 mL microwave vial and heated at 140°C under microwave irradiation for 10 min. Compound **13** is obtained as a white solid that can be isolated by filtration, washed with water, ethanol and diethyl ether to afford 653 mg (2.56 mmol, 69 %) of spectroscopically pure product. m.p. >250°C. ^{1}H-NMR (400 MHz, DMSO-d_6) δ (ppm): 10.02 (s, 1H, N8-H), 8.68 (s, 1H, N9-H), 7.93 – 7.66 (m, 2H, C11-H), 7.26 – 7.07 (m, 2H, C12-H), 6.85 – 6.80 (m, 1H, C13-H), 6.35 (s, 2H, N14-H), 2.60 – 2.46 (m, 4H, C5-H, C6-H). ^{13}C-NMR (100.6 MHz, DMSO-d_6) δ (ppm): 171.7 (C7), 161.4 (C4), 158.1 (C2), 156.3 (C8a), 141.5 (C10), 128.2 (C12), 120.2 (C13), 118.3 (C11), 85.8 (C4a), 30.5 (C6), 17.2 (C5). IR (KBr) ν (cm^{-1}): 3467, 3198, 1679, 1641, 1593, 1575, 1543, 1438, 1375, 1226, 781, 750, 701.

1.1.2. Synthesis and characterization of 2-(phenylamino)-5,6-dihydropyrido[2,3-*d*]pyrimidine-4,7(3*H*,8*H*)-dione (14)

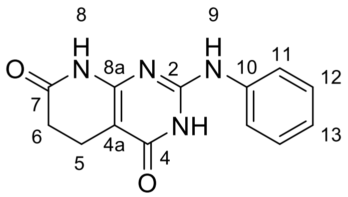

A mixture of 4-amino-2-(phenylamino)-5,6-dihydropyrido[2,3-*d*]pyrimidin-7(8*H*)-one (**13**) (400 mg, 1.56 mmol), *t*-butyl nitrite (1.0 mL, 8.6 mmol), water (2.0 mL) and DMF (8.0 mL) is sealed in a 20mL microwave vial and heated at 65°C for 10 minutes under microwave irradiation. The resulting slurry is diluted with water (50 mL) and the white solid can be isolated by filtration and washed with water, ethanol and diethyl ether to afford 364 mg (1.29 mmol, 90%) of spectroscopically pure **14**. m.p. >250°C. ^{1}H-NMR (400 MHz, DMSO-d_6) δ (ppm): 10.40 (s, 1H, N8-H), 10.19 (s, 1H, N3-H), 8.73 (s, 1H, N9-H), 7.75 – 7.61 (m, 2H, C11-H), 7.38 – 7.22 (m, 2H, C12-H), 7.10 – 6.94 (m, 1H, C13-H), 2.55 – 2.41 (m, 4H, C5-H, C6-H). ^{13}C-NMR (100.6 MHz, DMSO-d_6) δ (ppm): 171.5 (C7), 161.4 (C4), 155.7 (C8a), 150.8 (C2), 138.7 (C10), 128.8 (C12), 122.5 (C13), 119.3 (C11), 90.6 (C4a), 30.3 (C6), 16.4 (C5). IR (KBr) ν (cm^{-1}): 3402, 2824, 1665, 1615, 1558, 1509, 1470, 1437, 1378, 1335, 1319, 1209, 869, 759.

1.1.3. Synthesis and characterization of 4-((1*H*-benzo[*d*][1,2,3]triazol-1-yl)oxy)-2-(phenylamino)-5,6-dihydropyrido[2,3-*d*]pyrimidin-7(8*H*)-one (15)

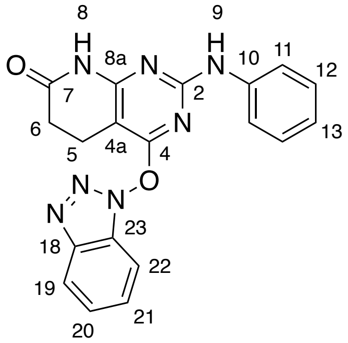

2-(phenylamino)-5,6-dihydropyrido[2,3-*d*]pyrimidine-4,7(3*H*,8*H*)-dione (**14**) (512 mg, 2.0 mmol) was suspended in ACN (15 mL). After 5 minutes of stirring, BOP (1.47 g, 3.32 mmol) and DBU (0.8 mL, 5.32 mmol) were added to the solution. The mixture was stirred at room temperature for two days. Then, water (200 mL) was added to the reaction mixture. The yellowish solid was collected by filtration and washed with water, ethyl acetate and diethyl ether to afford 709.1 mg (1.90 mmol, 95%) of spectroscopically pure 4-((1*H*-benzo[*d*][1,2,3]triazol-1-yl)oxy)-2-(phenylamino)-5,6-dihydropyrido[2,3-*d*]pyrimidin-7(8*H*)-one (**15**). m.p. >250°C. ^{1}H-NMR (400 MHz, DMSO-d_6) δ (ppm): 10.92 (s, 1H, N8-H), 9.39 (s, 1H, N9-H), 8.27 – 8.19 (m, 1H, C19-H), 7.85 – 7.78 (m, 1H, C22-H), 7.71 – 7.62 (m, 1H, C21-H), 7.60 – 7.51 (m, 1H, C20-H), 6.98 – 6.86 (m, 2H, C11-H), 6.87 – 6.76 (m, 2H, C12-H), 6.78 – 6.68 (m, 1H, C13-H), 3.04 (dd, *J* = 8.2, 7.2 Hz, 2H, C5-H), 2.72 (dd, *J* = 8.2, 7.2 Hz, 2H, C6-H). ^{13}C-NMR (100.6 MHz, DMSO-d_6) δ (ppm): 171.4 (C7), 165.0 (C4*), 160.7 (C8a*), 157.0 (C2), 142.9 (C18), 139.3 (C10), 129.1 (C21), 128.6 (C23), 127.8 (C12), 125.2 (C20), 121.4 (C13), 119.8 (C19), 118.4 (C11), 109.4 (C22), 88.1 (C4a), 29.9 (C6), 15.9 (C5). IR (KBr) ν (cm^{-1}): 3278, 3205, 3122, 2934, 1682, 1626, 1582, 1499, 1447, 1370, 1347, 1239, 1094, 1013, 844, 752.

1.1.4. Synthesis and characterization of 7-oxo-2-(phenylamino)-5,6,7,8-tetrahydropyrido[2,3-*d*]pyrimidin-4-yl trifluoromethanesulfonate (16)

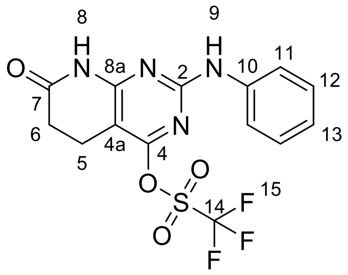

A mixture of 2-(phenylamino)-5,6-dihydropyrido[2,3-*d*]pyrimidine-4,7(3*H*,8*H*)-dione (**14**) (500 mg, 1.95 mmol) and pyridine (8mL) was stirred in a 25 mL round-bottom flask for 10 minutes under Argon atmosphere. Then trifluoromethanesulfonic anhydride (0.65 mL, 3.90 mmol) was added. The mixture was stirred at room temperature for 30 minutes. After that, the mixture was concentrated and the brown solid was washed with water (50 mL) in order to eliminate the pyridinium salt. The resulting precipitate was filtered and washed with water and abundant ethanol to afford 692.5 mg (1.78 mmol, 91%) of the grayish pure product **16**. m.p. 250°C. ^{1}H-NMR (400 MHz, DMSO-d_6) δ (ppm): 11.16 (s, 1H, N8-H), 9.82 (s, 1H, N9-H), 7.81 – 7.67 (m, 2H, C11-H), 7.38 – 7.20 (m, 2H, C12-H), 7.11 – 6.87 (m, 1H, C13-H), 2.80 – 2.75 (m, 2H, C5-H), 2.64 – 2.59 (m, 2H, C6-H). ^{13}C-NMR (100.6 MHz, DMSO-d_6) δ (ppm): 171.2 (C7), 162.0 (C4), 158.8 (C8a), 157.2 (C2), 139.3 (C10), 128.4 (C12), 122.3 (C13), 119.4 (C11), 116.2 (C14), 93.9 (C4a), 29.5 (C6), 16.6 (C5). ^{19}F NMR (376 MHz, DMSO-d_6) δ (ppm): -73.73 (F15). IR (KBr) ν (cm^{-1}): 3291, 3215, 3159, 2986, 1699, 1653, 1628, 1581, 1559, 1502, 1462, 1502, 1462, 1408, 1346, 1217, 1140, 875, 841, 751.

1.1.5. Synthesis and characterization of 4-iodo-2-(phenylamino)-5,6-dihydropyrido[2,3-*d*]pyrimidin-7(8*H*)-one (17)

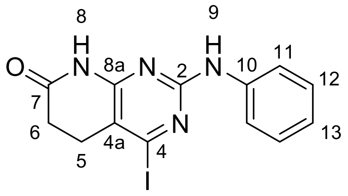

A mixture of 7-oxo-2-(phenylamino)-5,6,7,8-tetrahydropyrido[2,3-*d*]pyrimidin-4-yl trifluoromethanesulfonate (**16**) (400 mg, 1.03 mmol), dry sodium iodide (1540 mg, 10.13 mmol), acetyl chloride (110 µL, 1.55 mmol) and dry acetonitrile (12 mL) was stirred for 10 minutes at room temperature in a sealed 20 mL microwave vial. The resulting dispersion was heated for 5 hours at 80°C under microwave irradiation. Then, the mixture was concentrated in a rotary evaporator distillation system. After that, the brownish residue was washed with water and it was filtered. The product was washed with more water, ethanol and diethyl ether in order to afford 364.8 mg (0.99 mmol, 96%) of spectroscopically pure 4-iodo-2-(phenylamino)-5,6-dihydropyrido[2,3-*d*]pyrimidin-7(8*H*)- one (**17**) as a pale yellow solid. m.p. >250°C. ^{1}H-NMR (400 MHz, DMSO-d_6) δ (ppm): 10.77 (s, 1H, N8-H), 9.65 (s, 1H, N9-H), 7.87 – 7.73 (m, 2H, C11-H), 7.32 – 7.18 (m, 2H, C12-H), 7.00 – 6.88 (m, 1H, C13-H), 2.79 – 2.72 (m, 2H, C5-H), 2.63 – 2.56 (m, 2H, C6-H). ^{13}C-NMR (100.6 MHz, DMSO-d_6) δ (ppm): 171.7 (C7), 157.5 (C8a), 156.5 (C2), 140.6 (C10), 133.5 (C4), 128.9 (C12), 121.9 (C13), 119.2 (C11), 110.9 (C4a), 30.7 (C6), 26.7 (C5). IR (KBr) ν (cm^{-1}): 3275, 3202, 3146, 3046, 2973, 1685, 1593, 1566, 1530, 1499, 1440, 1332, 1299, 1249, 1228, 1210, 901.

1.2. General procedure for C4 amino substituted products 18

Methodology A: The intermediate 4-((1*H*-benzo[*d*][1,2,3]triazol-1-yl)oxy)-2-(phenylamino)-5,6-dihydropyrido[2,3-*d*]pyrimidin-7(8*H*)-one (**15**) (100 mg, 0.267 mmol) was suspended in ACN (20 ml), 3 equivalents of the corresponding amine (0.803 mmol) were added to the suspension and the mixture was heated at 140°C under microwave irradiation for 6 hours. Then, water was added to the residue and the solid was collected by filtration and washed with water, ethanol and EtOEt in order to afford the corresponding spectroscopically pure product.

Methodology B: The intermediate 7-oxo-2-(phenylamino)-5,6,7,8-tetrahydropyrido[2,3-*d*]pyrimidin-4-yl trifluoromethanesulfonate (**16**) (100 mg, 0.257 mmol) was suspended in ACN (10 ml), 1.2 equivalents of the corresponding amine (0.803 mmol) were added to the suspension and the mixture was heated at 100°C for 8 – 16 hours. Then, water was added to the residue and the solid was collected by filtration and washed with water, ethanol and EtOEt in order to afford the corresponding spectroscopically pure product.

1.2.1. Synthesis and characterization of 4-((3-morpholinopropyl)amino)-2-(phenylamino)-5,6-dihydropyrido[2,3-*d*]pyrimidin-7(8*H*)-one (18a)

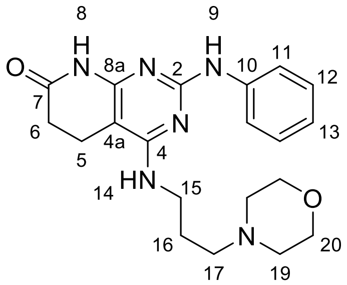

As above but carried out by using 4-((1*H*-benzo[*d*][1,2,3]triazol-1-yl)oxy)-2-(phenylamino)-5,6-dihydropyrido[2,3-*d*]pyrimidin-7(8*H*)-one (**15**) (100 mg, 0.267 mmol) and *N*-(3-aminopropyl)morpholine (117 μL, 0.803 mmol). The mixture was heated at 140°C for 6h under microwave irradiation. 54.1 mg (0.141 mmol, 54%) of spectroscopically pure 4-((3-morpholinopropyl)amino)-2-(phenylamino)-5,6-dihydropyrido[2,3-*d*]pyrimidin-7(8*H*)-one (**18a**) were obtained as an orangish solid. m.p. >250°C. ^{1}H-NMR (400 MHz, DMSO-d_6) δ (ppm): 9.96 (s, 1H, N8-H), 8.73 (s, 1H, N9-H), 7.85 – 7.79 (m, 2H, C11-H), 7.22 – 7.16 (m, 2H, C12-H), 6.88 – 6.81 (m, 1H, C13-H), 6.67 (t, *J* = 5.5 Hz, 1H, N14-H), 3.56 (t, *J* = 4.6 Hz, 4H, C20-H), 3.41 (q, *J* = 6.7 Hz, 2H, C15-H), 2.60 – 2.51 (m, 4H, C5-H, C6-H), 2.40 – 2.29 (m, 6H, C19-H, C17-H), 1.74 (p, *J* = 7.1 Hz, 2H, C16-H). ^{13}C-NMR (100.6 MHz, DMSO-d_6) δ (ppm): 171.1 (C7), 160.1 (C4), 157.9 (C2), 155.5 (C8a), 141.5 (C10), 128.2 (C12), 120.1 (C13), 118.2 (C11), 85.9 (C4a), 66.2 (C20), 56.4 (C17), 53.4 (C19), 39.1 (C15), 30.3 (C6), 26.0 (C16), 17.1 (C5). IR (KBr) ν (cm^{-1}): 3429, 3287, 3204, 2917, 2861, 1635, 1601, 1579, 1548, 1444, 1375, 1245, 1119, 752. OEA calculated for $C_{20}H_{26}N_6O_2$: C: 62.81%, H: 6.85%, N: 21.97%; found: C: 62.46%, H: 6.67%, N: 21.57%. HRMS (APCI-FIA-TOF) (m/z) calculated for $C_{20}H_{26}N_6O_2$: 382.2117, [M]$^+$, found: 383.2187, [M+H]$^+$.

1.2.2. Synthesis and characterization of 4-(4-ethylpiperazin-1-yl)-2-(phenylamino)-5,6-dihydropyrido[2,3-*d*]pyrimidin-7(8*H*)-one (**18b**)

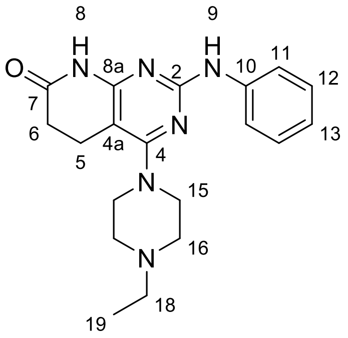

As above but carried out by using 4-((1*H*-benzo[*d*][1,2,3]triazol-1-yl)oxy)-2-(phenylamino)-5,6-dihydropyrido[2,3-*d*]pyrimidin-7(8*H*)-one (**15**) (100 mg, 0.267 mmol) and 1-ethylpiperazine (98.1 mg, 0.803 mmol). The mixture was heated at 140°C for 6h under microwave irradiation. 51.2 mg (0.145 mmol, 54%) of spectroscopically pure 4-(4-ethylpiperazin-1-yl)-2-(phenylamino)-5,6-dihydropyrido[2,3-*d*]pyrimidin-7(8*H*)-one (**18b**) were obtained as a pale yellow solid. m.p. >250°C. ^{1}H-NMR (400 MHz, DMSO-d_6) δ (ppm): 10.25 (s, 1H, N8-H), 8.98 (s, 1H, N9-H), 7.83 – 7.77 (m, 2H, C11-H), 7.25 – 7.18 (m, 2H, C12-H), 6.91 – 6.82 (m, 1H, C13-H), 3.28 (t, *J* = 5.8 Hz, 4H, C15-H), 2.67 (dd, *J* = 8.2, 6.6 Hz, 2H, C5-H), 2.50 – 2.43 (m, 6H, C6-H, C16-H), 2.38 (q, *J* = 7.0 Hz, 2H, C18-H), 1.03 (t, *J* = 7.2 Hz, 3H, C19-H). ^{13}C-NMR (100.6 MHz, DMSO-d_6) δ (ppm): 171.8 (C7), 164.9 (C4), 158.7 (C8a), 157.7 (C2), 141.5 (C10), 128.7 (C12), 120.9 (C13), 118.8 (C11), 93.0 (C4a), 52.7 (C16), 52.1 (C18), 48.3 (C15), 31.3 (C6), 20.5 (C5), 12.3 (C19). IR (KBr) ν (cm^{-1}): 3428, 3287, 3203, 3140, 2968, 2928, 2812, 1691, 1595, 1566, 1440, 1361, 1239, 1017, 751. OEA calculated for $C_{19}H_{24}N_6O$: C: 64.75%, H: 6.86%, N: 23.85%; found: C: 64.97%, H: 6.84%, N: 23.63%. HRMS (APCI-FIA-TOF) (m/z) calculated for $C_{19}H_{24}N_6O$: 352.2012, [M]$^+$, found: 353.2070, [M+H]$^+$.

1.2.3. Synthesis and characterization of *tert*-butyl 4-(7-oxo-2-(phenylamino)-5,6,7,8-tetrahydropyrido[2,3-*d*]pyrimidin-4-yl)piperazine-1-carboxylate (**18c**)

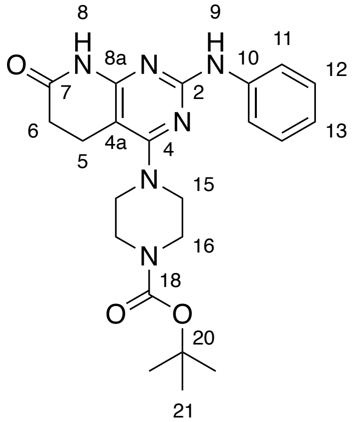

As above but carried out by using 4-((1*H*-benzo[*d*][1,2,3]triazol-1-yl)oxy)-2-(phenylamino)-5,8-dihydropyrido[2,3-*d*]pyrimidin-7(8*H*)-one (**15**) (200 mg, 0.535 mmol) and *tert*-butyl piperazine-1-carboxylate (290 μL, 1.610 mmol). The mixture was heated at 140°C for 8h under microwave irradiation. 153.4 mg (0.361 mmol, 67%) of spectroscopically pure *tert*-butyl 4-(7-oxo-2-(phenylamino)-5,6,7,8-tetrahydropyrido[2,3-*d*]pyrimidin-4-yl)piperazine-1-carboxylate (**18c**) were obtained as a brown solid. m.p. >250°C. ^{1}H-NMR (400 MHz, DMSO-d_6) δ (ppm): 10.29 (s, 1H, N8-H), 9.01 (s, 1H, N9-H), 7.83 – 7.74 (m, 2H, C11-H), 7.28 – 7.18 (m, 2H, C12-H), 6.94 – 6.83 (m, 1H, C13-H), 3.45 (t, *J* = 5.2 Hz, 4H, C16-H), 3.25 (t, *J* = 5.2 Hz, 4H, C15-H), 2.68 (dd, *J* = 8.4, 6.8 Hz, 2H, C5-H), 2.44 (dd, *J* = 8.4, 6.8 Hz, 2H, C6-H), 1.42 (s, 9H, C21-H). ^{13}C-NMR (100.6 MHz, DMSO-d_6) δ (ppm): 171.4 (C7), 164.5 (C18), 158.5 (C4), 157.3 (C8a), 153.9 (C2), 141.1 (C10), 128.3 (C12), 120.6 (C13), 118.5 (C11), 92.8 (C4a), 79.0 (C20), 57.6 (C16), 47.7 (C15), 30.8 (C6), 28.0 (C21), 19.8 (C5). IR (ATR) ν (cm^{-1}): 3281, 3131, 2974, 2924, 1690, 1594, 1564, 1541, 1440, 1415, 1360, 1240, 1163, 1125, 996, 891, 862, 753. HRMS (APCI-FIA-TOF) (m/z) calculated for $C_{22}H_{28}N_6O_3$: 424.2223, [M]$^+$, found: 425.2290, [M+H]$^+$.

1.2.4. Synthesis and characterization of *tert*-butyl (1*S*,4*S*)-5-(7-oxo-2-phenylamino)-5,6,7,8-tetrahydropyrido[2,3-*d*]pyrimidin-4-yl)-2,5-diazabicyclo[2.2.1]heptane-2-carboxylate (18d)

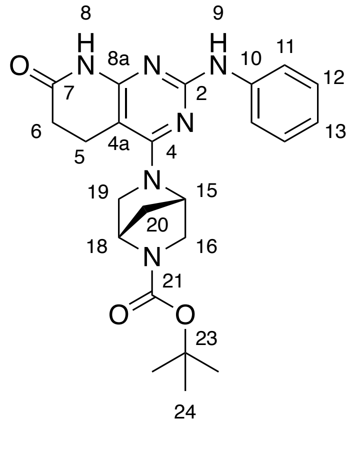

As above but carried out by using 7-oxo-2-(phenylamino)-5,6,7,8-tetrahydropyrido[2,3-*d*]pyrimidin-4-yl trifluoromethanesulfonate (**16**) (150 mg, 0.386 mmol) and *tert*-butyl (1*S*,4*S*)-2,5-diazabicyclo[2.2.1]heptane-2-carboxylate (229.8 mg, 1.16 mmol). The mixture was heated for 16h. 106.2 mg (0.242 mmol, 63%) of spectroscopically pure *tert*-butyl (1*S*,4*S*)-5-(7-oxo-2-phenylamino)-5,6,7,8-tetrahydropyrido[2,3-*d*]pyrimidin-4-yl)-2,5-diazabicyclo[2.2.1]heptane-2-carboxylate (**18d**) were obtained as a white solid. m.p. >250°C. ^{1}H-NMR (400 MHz, DMSO-d_6) δ (ppm): 10.11 (s, 1H, N8-H), 8.85 (s, 1H, N9-H), 7.79 – 7.72 (m, 2H, C11-H), 7.26 – 7.16 (m, 2H, C12-H), 6.90 – 6.82 (m, 1H, C13-H), 4.80 (d, 1H, C15-H*), 4.42 (d, 1H, C18-H*), 3.82 (d, 1H, C19-H**), 3.52 – 3.34 (m, 3H, C19-H, C16-H**), 2.86 (dt, *J* = 15.4, 8.5 Hz, 1H, C5-H), 2.72 (dt, *J* = 15.6, 6.5 Hz, 1H, C5-H), 2.44 (dd, *J* = 8.5, 6.5 Hz, 2H, C6-H), 1.90 – 1.82 (m, 2H, C20-H), 1.37 (d, 9H, C24-H). ^{13}C-NMR (100.6 MHz, DMSO-d_6) δ (ppm): 171.2 (C7), 160.6 (C21*), 160.6 (C4*), 157.9 (C8a*), 156.9 (C2*), 141.2 (C10), 128.3 (C12), 120.4 (C13), 118.3 (C11), 88.9 (C4a), 78.8 (C23), 78.7 (C23), 58.9 (C15**), 58.3 (C15**), 57.6 (C19***), 57.3 (C19***), 57.2 (C18**), 56.1 (C18**), 52.6 (C16***), 52.4 (C16***), 36.0 (C20), 35.5 (C20), 30.9 (C6), 28.1 (C24), 28.1 (C24), 19.3 (C5). IR (ATR) ν (cm^{-1}): 3290, 3213, 2976, 1680, 1592, 1566, 1534, 1442, 1407, 1377, 1317, 1230, 1151, 1099, 847, 753. HRMS (APCI-FIA-TOF) (m/z) calculated for $C_{23}H_{28}N_6O_3$: 436.2223, [M]$^+$, found: 437.2283, [M+H]$^+$.

1.2.5. Synthesis and characterization of *tert*-butyl 7-(7-oxo-2-phenylamino)-5,6,7,8-tetrahydropyrido[2,3-*d*]pyrimidin-4-yl)-2,7-diazaspiro[4.4]nonane-2-carboxylate (18e)

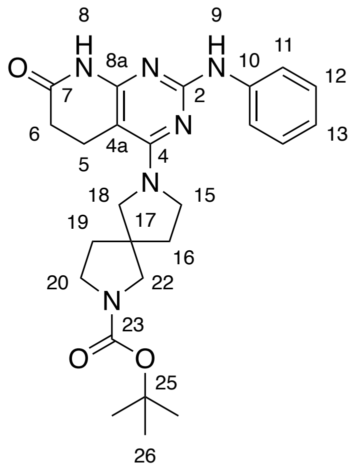

As above but carried out by using 7-oxo-2-(phenylamino)-5,6,7,8-tetrahydropyrido[2,3-*d*]pyrimidin-4-yl trifluoromethanesulfonate (**16**) (200 mg, 0.515 mmol) and *tert-butyl* 2,7-diazaspiro[4.4]nonane-2-carboxylate (349.7 mg, 1.55 mmol). The mixture was heated for 12h. 349.7 mg (0.428 mmol, 83%) of pure *tert*-butyl 7-(7-oxo-2-phenylamino)-5,6,7,8-tetrahydropyrido[2,3-*d*]pyrimidin-4-yl)-2,7-diazaspiro[4.4]nonane-2-carboxylate (**18e**) were obtained as an orangish solid. m.p. >250°C. ^{1}H-NMR (400 MHz, DMSO-d_6) δ (ppm): 10.04 (s, 1H, N8-H), 8.78 (s, 1H, N9-H), 7.85 – 7.73 (m, 2H, C11-H), 7.25 – 7.12 (m, 2H, C12-H), 6.86 – 6.81 (m, 1H, C13-H), 3.76 – 3.69 (m, 2H), 3.62 – 3.52 (m, 2H), 3.39 – 3.33 (m, 2H), 3.26 – 3.17 (m, 2H), 2.93 (dd, *J* = 8.5, 6.6 Hz, 2H, C5-H), 2.43 (dd, *J* = 8.5, 6.7

Hz, 2H, C6-H), 1.93 – 1.79 (m, 4H), 1.40 (d, *J* = 3.1 Hz, 9H, C26-H). ^{13}C-NMR (100.6 MHz, DMSO-*d*$_6$) δ (ppm): 171.3 (C7), 160.5 (C23*), 157.7 (C4*), 157.0 (C8a*), 153.6 (C2*), 141.4 (C10), 128.2 (C12), 120.2 (C13), 118.3 (C11), 88.3 (C4a), 78.4 (C25), 57.8, 54.4, 48.22, 44.9, 33.9, 33.3, 31.1 (C6), 28.2 (C26), 19.6 (C5). IR (ATR) ν (cm^{-1}): 3286, 3120, 2972, 2868, 1685, 1593, 1562, 1430, 1385, 1352, 1232, 1158, 1113, 842, 750. HRMS (APCI-FIA-TOF) (m/z) calculated for $C_{25}H_{32}N_6O_3$: 464.2536, [M]$^+$, found: 465.2600, [M+H]$^+$.

1.2.6. Synthesis and characterization of *tert*-butyl 7-(7-oxo-2-phenylamino)-5,6,7,8-tetrahydropyrido[2,3-*d*]pyrimidin-4-yl)-2,7-diazaspiro[3.5]nonane-2-carboxylate (18f)

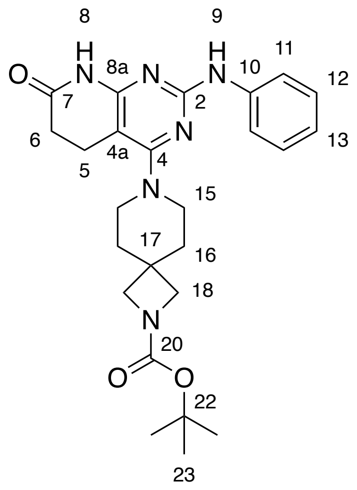

As above but carried out by using 7-oxo-2-(phenylamino)-5,6,7,8-tetrahydropyrido[2,3-*d*]pyrimidin-4-yl trifluoromethanesulfonate (**16**) (100 mg, 0.257 mmol) and *tert*-butyl 2,7-diazaspiro[3.5]nonane-2-carboxylate (174.9 mg, 0.772 mmol). The mixture was heated for 12h. 94.2 mg (0.202 mmol, 79%) of spectroscopically pure 7-(7-oxo-2-phenylamino)-5,6,7,8-tetrahydropyrido[2,3-*d*]pyrimidin-4-yl)-2,7-diazaspiro[3.5]nonane-2-carboxylate (**18f**) were obtained as a brown solid. m.p. >250°C. ^{1}H-NMR (400 MHz, DMSO-*d*$_6$) δ (ppm): 10.22 (s, 1H, N8-H), 8.96 (s, 1H, N9-H), 7.85 – 7.73 (m, 2H, C11-H), 7.26 – 7.16 (m, 2H, C12-H), 6.93 – 6.81 (m, 1H, C13-H), 3.60 (s, 4H, C18-H), 3.22 (t, *J* = 5.2 Hz, 4H, C15-H), 2.66 (dd, *J* = 8.0, 6.7 Hz, 2H, C-5), 2.45 (dd, *J* = 8.0, 6.7 Hz, 2H, C-6), 1.78 (t, *J* = 5.2 Hz, 4H, C16-H), 1.38 (s, 9H, C23-H). ^{13}C-NMR (100.6 MHz, DMSO-*d*$_6$) δ (ppm): 171.4 (C7), 164.7 (C20), 158.3 (C4), 157.3 (C8a), 155.6 (C2), 141.1 (C10), 128.3 (C12), 120.5 (C13), 118.4 (C11), 92.5 (C4a), 78.4 (C22), 45.1 (C15), 34.8 (C16), 33.3 (C17), 30.9 (C6), 28.1 (C23), 20.1 (C5). IR (ATR) ν (cm^{-1}): 3289, 3135, 2933, 2871, 1692, 1593, 1565, 1541, 1437, 1360, 1237, 1152, 1082, 845, 751. HRMS (APCI-FIA-TOF) (m/z) calculated for $C_{25}H_{32}N_6O_3$: 464.2536, [M]$^+$, found: 465.2597, [M+H]$^+$.

1.2.7. Synthesis and characterization of *tert*-butyl (3a*R*,6a*S*)-5-(7-oxo-2-(phenylamino)-5,6,7,8-tetrahydropyrido[2,3-*d*]pyrimidin-4-yl)hexahydropyrrolo[3,4-*c*]pyrrole-2(1*H*)-carboxylate (18g)

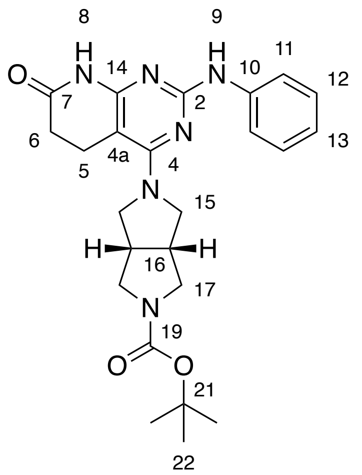

As above but carried out by using 7-oxo-2-(phenylamino)-5,6,7,8-tetrahydropyrido[2,3-*d*]pyrimidin-4-yl trifluoromethanesulfonate (**16**) (150 mg, 0.386 mmol) and *tert*-butyl (3a*R*,6a*S*)-hexahydropyrrolo[3,4-*c*]pyrrole-2(1*H*)-carboxylate (246.1 mg, 1.16 mmol). The mixture was heated for 16h. 113.2 mg (0.251 mmol, 65%) of spectroscopically pure *tert*-butyl (3a*R*,6a*S*)-5-(7-oxo-2-(phenylamino)-5,6,7,8-tetrahydropyrido[2,3-*d*]pyrimidin-4-yl)hexahydropyrrolo[3,4-*c*]pyrrole-2(1*H*)-carboxylate (**18g**) were obtained as a pale brown solid. m.p. >250°C. ^{1}H-NMR (400 MHz, DMSO-d_6) δ (ppm): 10.06 (s, 1H, N8-H), 8.79 (s, 1H, N9-H), 7.82 – 7.71 (m, 2H, C11-H), 7.25 – 7.15 (m, 2H, C12-H), 6.87 – 6.82 (m, 1H, C13-H), 3.89 – 3.78 (m, 2H, C15-H), 3.57 – 3.47 (m, 4H, C15-H, C17-H), 3.20 – 3.12 (m, 2H, C17-H), 2.96 – 2.86 (m, 4H, C5-H, C16-H), 2.43 (dd, *J* = 8.2, 6.7 Hz, 2H, C6-H), 1.39 (s, 9H, C22-H). ^{13}C-NMR (100.6 MHz, DMSO-d_6) δ (ppm): 171.3 (C7), 160.7 (C4), 157.7 (C8a), 156.9 (C19), 153.6 (C2), 141.3 (C10), 128.2 (C12), 120.3 (C13), 118.3 (C11), 88.6 (C4a), 78.4 (C21), 52.8 (C15), 49.6 (C17), 49.5 (C17), 41.3 (C16), 31.1 (C6), 28.1 (C22), 19.7 (C5). IR (ATR) ν (cm^{-1}): 3419, 3286, 3207, 3141, 2972, 2875, 1690, 1594, 1563, 1536, 1431, 1401, 1364, 1233, 1168, 1126, 752. HRMS (APCI-FIA-TOF) (m/z) calculated for $C_{24}H_{30}N_6O_3$: 450.2379, [M]$^+$, found: 451.2452, [M+H]$^+$.

1.2.8. Synthesis and characterization of *tert*-butyl 3-(((7-oxo-2-(phenylamino)-5,6,7,8-tetrahydropyrido[2,3-*d*]pyrimidin-4-yl)amino)methyl)azetidine-1-carboxylate (18h)

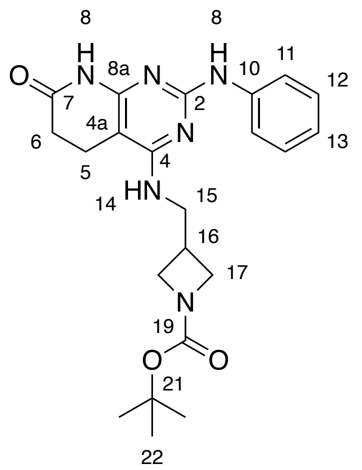

As above but carried out by using 7-oxo-2-(phenylamino)-5,6,7,8-tetrahydropyrido[2,3-*d*]pyrimidin-4-yl trifluoromethanesulfonate (**16**) (130 mg, 0.334 mmol) and *tert*-butyl 3-(aminomethyl)azetidine-1-carboxylate (187.0 mg, 1.00 mmol). The mixture was heated for 8h. 101.4 mg (0.238 mmol, 71%) of spectroscopically pure 3-(((7-oxo-2-(phenylamino)-5,6,7,8-tetrahydropyrido[2,3-*d*]pyrimidin-4-yl)amino)methyl)azetidine-1-carboxylate (**18h**) were obtained as a white solid. m.p. >250°C. ^{1}H-NMR (400 MHz, DMSO-d_6) δ (ppm): 10.03 (s, 1H, N8-H), 8.80 (s, 1H, N9-H), 7.82 – 7.75 (m, 2H, C11-H), 7.22 – 7.15 (m, 2H, C12-H), 6.86 – 6.77 (m, 2H, C13-H, N14-H),

3.91 – 3.83 (m, 2H, C17-H), 3.66 – 3.54 (m, 4H, C15-H, C17-H), 2.85 – 2.78 (m, 1H, C16-H), 2.59 – 2.50 (m, 4H, C5-H, C6-H), 1.36 (s, 9H, C22-H). ^{13}C-NMR (100.6 MHz, DMSO-*d*$_6$) δ (ppm): 171.2 (C7), 160.1 (C4), 157.9 (C19), 155.8 (C8a), 155.6 (C2), 141.4 (C10), 128.2 (C12), 120.2 (C13), 118.3 (C11), 86.0 (C4a), 78.3 (C21), 43.6 (C15), 30.3 (C6), 28.0 (C22, C16), 16.9 (C5). IR (ATR) ν (cm^{-1}): 3398, 3285, 3207, 3153, 2893, 1678, 1619, 1580, 1547, 1480, 1407, 1365, 1244, 1227, 1164, 1132, 844, 785, 751. HRMS (APCI-FIA-TOF) (m/z) calculated for $C_{22}H_{28}N_6O_3$: 424.2223, [M]$^+$, found: 425.2287, [M+H]$^+$.

1.3. General procedure for C4 arylamino substituted products 19

Intermediate 7-oxo-2-(phenylamino)-5,6,7,8-tetrahydropyrido[2,3-*d*]pyrimidin-4-yl trifluoromethanesulfonate (**16**) (1 eq), cesium carbonate (1.2 eq), palladium(II)acetate (0.1 eq), XPhos (0.15 eq) and the corresponding aniline (1.1 eq) were introduced under Argon atmosphere into a Schlenk tube. After that anhydrous toluene (1 mL) was added and the mixture was heated overnight at 100°C. Then, water was added to the residue and the solid was collected by filtration and washed with water, ethanol and cyclohexane in order to afford the spectroscopically pure product.

1.3.1. Synthesis and characterization of 2-(phenylamino)-4-((3,4,5-trimethoxyphenyl)amino)-5,6-dihydropyrido[2,3-*d*]pyrimidin-7(8*H*)-one (19a)

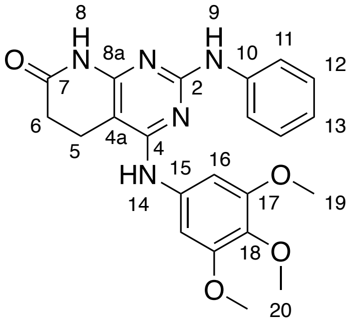

As above but carried out by using 7-oxo-2-(phenylamino)-5,6,7,8-tetrahydropyrido[2,3-*d*]pyrimidin-4-yl trifluoromethanesulfonate (**16**) (77.66 mg, 0.20 mmol) and 3,4,5-trimethoxyaniline (40.30 mg,0.22 mmol). 79.2 mg (0.187 mmol, 94%) of spectroscopically pure 2-(phenylamino)-4-((3,4,5-trimethoxyphenyl)amino)-5,6-dihydropyrido[2,3-*d*]pyrimidin-7(8*H*)-one (**19a**) were obtained as a pale brown solid. m.p. >250°C. ^{1}H-NMR (400 MHz, DMSO-*d*$_6$) δ (ppm): 10.17 (s, 1H, N8-H), 8.87 (s, 1H, N9-H), 8.29 (s, 1H, N14-H), 7.75 – 7.72 (m, 2H, C11-H), 7.13 – 7.09 (m, 2H, C12-H), 6.90 (s, 2H, C16-H), 6.86 – 6.82 (m, 1H, C13-H), 3.69 (s, 6H, C19-H), 3.65 (s, 3H, C20-H), 2.77 (dd, *J* = 8.3, 7.0 Hz, 2H, C5-H), 2.56 (dd, *J* = 8.2, 7.0 Hz, 2H, C6-H). ^{13}C-NMR (100.6 MHz, DMSO-*d*$_6$) δ (ppm): 171.2 (C7), 158.1 (C4), 157.5 (C2), 157.0 (C8a), 152.4 (C17), 141.0 (C10), 135.9 (C15), 133.1 (C18), 128.1 (C12), 120.5 (C13), 118.6 (C11), 100.2 (C16), 87.9 (C4a), 60.1 (C20), 55.6 (C19), 30.3 (C6), 17.3 (C5). IR (KBr) ν (cm^{-1}): 3350, 3288, 3206, 3138, 2959, 2927, 2851, 1677, 1601, 1580, 1503, 1443, 1239, 1221, 1129, 996, 745. HRMS (APCI-FIA-TOF) (m/z) calculated for $C_{22}H_{23}N_5O_4$: 421.1750, [M]$^+$, found: 422.1820, [M+H]$^+$.

1.3.2. Synthesis and characterization of 4-((4-morpholinophenyl)amino)-2-(phenylamino)-5,6-dihydropyrido[2,3-*d*]pyrimidin-7(8*H*)-one (**19b**)

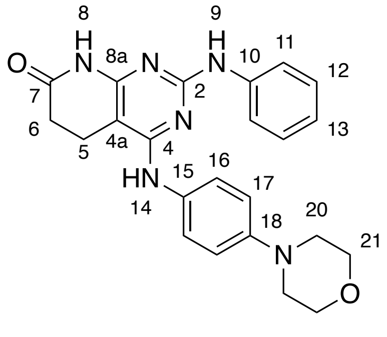

As above but carried out by using 7-oxo-2-(phenylamino)-5,6,7,8-tetrahydropyrido[2,3-*d*]pyrimidin-4-yl trifluoromethanesulfonate (**16**) (77.66 mg, 0.20 mmol) and 4-morpholinoaniline (39.21 mg,0.22 mmol). 71.2 mg (0.170 mmol, 85%) of spectroscopically pure 4-((4-morpholinophenyl)amino)-2-(phenylamino)-5,6-dihydropyrido[2,3-*d*]pyrimidin-7(8*H*)-one (**19b**) were obtained as a grey solid. m.p. >250°C. ^{1}H-NMR (400 MHz, DMSO-d_6) δ (ppm): 10.11 (s, 1H, N8-H), 8.78 (s, 1H, N9-H), 8.20 (s, 1H, N14-H), 7.74 – 7.69 (m, 2H, C11-H), 7.47 – 7.43 (m, 2H, C16-H), 7.15 – 7.09 (m, 2H, C12-H), 6.94 – 6.89 (m, 2H, C17-H), 6.86 – 6.80 (m, 1H, C13-H), 3.78 – 3.72 (m, 4H, C21-H), 3.11 – 3.06 (m, 4H, C20-H), 2.76 (dd, *J* = 8.3, 7.0 Hz, 2H, C5-H), 2.55 (dd, *J* = 8.3, 7.0 Hz, 2H, C6-H). ^{13}C-NMR (100.6 MHz, DMSO-d_6) δ (ppm): 171.2 (C7), 158.4 (C4), 157.6 (C2), 156.6 (C8a), 147.1 (C18), 141.1 (C10), 132.1 (C15), 128.1 (C12), 123.8 (C16), 120.3 (C13), 118.5 (C11), 115.1 (C17), 87.2 (C4a), 66.1 (C21), 49.1 (C20), 30.3 (C6), 17.3 (C5). IR (KBr) ν (cm^{-1}): 3439, 3280, 3201, 3125, 2960, 2853, 1688, 1597, 1574, 1514, 1442, 1370, 1245, 1210, 1122, 926, 826, 761. HRMS (APCI-FIA-TOF) (m/z) calculated for $C_{23}H_{24}N_6O_2$: 416.1961, [M]$^+$, found: 417.2039, [M+H]$^+$.

1.3.3. Synthesis and characterization of 4-((7-oxo-2-(phenylamino)-5,6,7,8-tetrahydropyrido[2,3-*d*]pyrimidin-4-yl)amino)benzonitrile (**19c**)

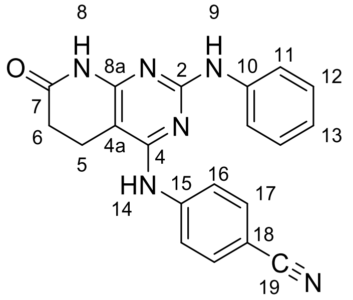

As above but carried out by using 7-oxo-2-(phenylamino)-5,6,7,8-tetrahydropyrido[2,3-*d*]pyrimidin-4-yl trifluoromethanesulfonate (**16**) (77.66 mg, 0.20 mmol) and 4-aminobenzonitrile (26.0 mg,0.22 mmol). 64.3 mg (0.180 mmol, 87%) of spectroscopically pure 4-((7-oxo-2-(phenylamino)-5,6,7,8-tetrahydropyrido[2,3-*d*]pyrimidin-4-yl)amino)benzonitrile (**19c**) were obtained as a grey solid. m.p. >250°C. ^{1}H-NMR (400 MHz, DMSO-d_6) δ (ppm): 10.37 (s, 1H, N8-H), 9.08 (s, 1H, N9-H), 8.75 (s, 1H, N14-H), 7.98 – 7.94 (m, 2H, C16-H), 7.76 – 7.72 (m, 2H, C11-H), 7.71 – 7.67 (m, 2H, C17-H), 7.25 – 7.20 (m, 2H, C12-H), 6.94 – 6.89 (m, 1H, C13-H), 2.84 (dd, *J* = 8.3, 7.0 Hz, 2H, C5-H), 2.57 (dd, *J* = 8.3, 7.0 Hz, 2H, C6-H). ^{13}C-NMR (100.6 MHz, DMSO-d_6) δ (ppm): 171.2 (C7), 157.7 (C8a), 157.5 (C2), 157.0 (C4), 144.9 (C15), 140.7 (C10), 132.6 (C17), 128.3 (C12), 121.0 (C13), 120.3 (C16), 119.5 (C19), 119.1 (C11), 102.9 (C18), 89.5 (C4a), 30.2 (C6), 17.4 (C5). IR (KBr) ν (cm^{-1}): 3386, 3265, 3201, 3125, 2955, 2218, 1687, 1602, 1587, 1571, 1503, 1478, 1455, 1415, 1250, 1221, 1175, 843, 751, 545. HRMS (APCI-FIA-TOF) (m/z) calculated for $C_{20}H_{16}N_6O$: 356.1386, [M]$^+$, found: 357.1460, [M+H]$^+$.

1.3.4. Synthesis and characterization of 4-((4-(4-methylpiperazin-1-yl)phenyl)amino)-2-(phenylamino)-5,6-dihydropyrido[2,3-*d*]pyrimidin-7(8*H*)-one (19d)

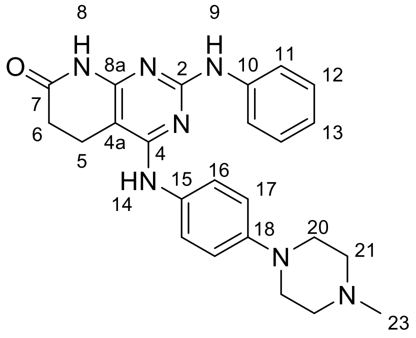

As above but carried out by using 7-oxo-2-(phenylamino)-5,6,7,8-tetrahydropyrido[2,3-*d*]pyrimidin-4-yl trifluoromethanesulfonate **(16)** (77.66 mg, 0.20 mmol) and 4-(4-methylpiperazin-1-yl)aniline (42.1 mg,0.22 mmol). 61.8 mg (0.144 mmol, 72%) of spectroscopically pure 4-((4-(4-methylpiperazin-1-yl)phenyl)amino)-2-(phenylamino)-5,6-dihydropyrido[2,3-*d*]pyrimidin-7(8*H*)-one (**19d**) were obtained as a brownish solid. m.p. >250°C. ^{1}H-NMR (400 MHz, DMSO-d_6) δ (ppm): 10.11 (s, 1H, N8-H), 8.78 (s, 1H, N9-H), 8.18 (s, 1H, N14-H), 7.75 – 7.68 (m, 2H, C11-H), 7.45 – 7.39 (m, 2H, C16-H), 7.15 – 7.08 (m, 2H, C12-H), 6.94 – 6.88 (m, 2H, C17-H), 6.87 – 6.78 (m, 1H, C13-H), 3.11 (t, J = 5.0 Hz, 4H, C20-H), 2.75 (dd, J = 8.3, 7.1 Hz, 2H, C5-H), 2.55 (dd, J = 8.3, 7.1 Hz, 2H, C6-H), 2.50 – 2.44 (m, 4H, C21-H), 2.24 (s, 3H, C23-H). ^{13}C-NMR (100.6 MHz, DMSO-d_6) δ (ppm): 171.2 (C7), 158.4 (C4), 157.6 (C2), 156.5 (C8a), 147.1 (C18), 141.1 (C10), 131.7 (C15), 128.0 (C12), 123.8 (C16), 120.3 (C13), 118.5 (C11), 115.4 (C17), 87.1 (C4a), 54.6 (C21), 48.7 (C20), 45.7 (C23), 30.3 (C6), 17.2 (C5). IR (KBr) ν (cm^{-1}): 3441, 3283, 3204, 3126, 2931, 2847, 1685, 1599, 1575, 1514, 1444, 1246, 1216, 825, 754. HRMS (APCI-FIA-TOF) (m/z) calculated for $C_{24}H_{27}N_7O$: 429.2277, [M]$^+$, found: 430.2338, [M+H]$^+$.

1.3.5. Synthesis and characterization of 4-((4-(dimethylamino)phenyl)amino)-2-(phenylamino)-5,6-dihydropyrido[2,3-*d*]pyrimidin-7(8*H*)-one (19e)

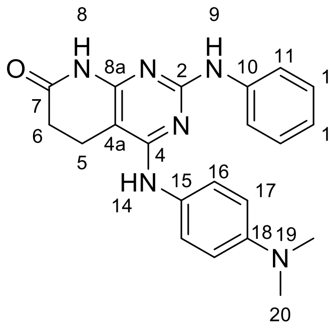

As above but carried out by using 7-oxo-2-(phenylamino)-5,6,7,8-tetrahydropyrido[2,3-*d*]pyrimidin-4-yl trifluoromethanesulfonate (**16**) (77.66 mg, 0.20 mmol) and *N*,*N*-dimethyl-*p*-phenylenediamine (29.96 mg, 0.22 mmol). 63.6 mg (0.169 mmol, 84%) of spectroscopically pure 4-((4-(dimethylamino)phenyl)amino)-2-(phenylamino)-5,6-dihydropyrido[2,3-*d*]pyrimidin-7(8*H*)-one (**19e**) were obtained as a grey solid. m.p. >250°C. ^{1}H-NMR (400 MHz, DMSO-d_6) δ (ppm): 10.07 (s, 1H, N8-H), 8.74 (s, 1H, N9-H), 8.14 (s, 1H, N14-H), 7.74 – 7.69 (m, 2H, C11-H), 7.39 – 7.34 (m, 2H, C16-H), 7.13 – 7.07 (m, 2H, C12-H), 6.84 – 6.79 (m, 1H, C13-H), 6.76 – 6.70 (m, 2H, C17-H), 2.89 (s, 6H, C20-H), 2.75 (dd, *J* = 8.3, 7.1 Hz, 2H, C5-H), 2.55 (dd, *J* = 8.3, 7.1 Hz, 2H, C6-H). ^{13}C-NMR (100.6 MHz, DMSO-d_6) δ (ppm): 171.7 (C7), 159.1 (C4), 158.1 (C2), 156.8 (C8a), 147.6 (C18), 141.6 (C10), 129.9 (C15), 128.5 (C12), 124.9 (C16), 120.6 (C13), 118.9 (C11), 112.9 (C17), 87.3 (C4a), 41.1 (C20), 30.8 (C6), 17.7 (C5). IR (KBr) ν (cm^{-1}): 3398, 3284, 3201, 3135, 2925, 1680, 1634, 1598, 1575, 1518, 1441, 1375, 1246, 820, 750. HRMS (APCI-FIA-TOF) (m/z) calculated for $C_{21}H_{22}N_6O$: 374.1855, [M]$^+$, found: 375.1930, [M+H]$^+$.

1.3.6. Synthesis and characterization of 4-((4-nitrophenyl)amino)-2-(phenylamino)-5,6-dihydropyrido[2,3-*d*]pyrimidin-7(8*H*)-one (19f)

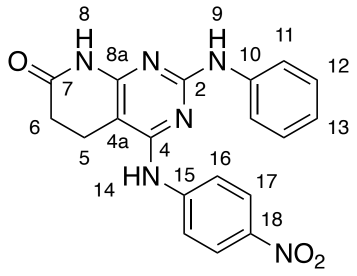

As above but carried out by using 7-oxo-2-(phenylamino)-5,6,7,8-tetrahydropyrido[2,3-*d*]pyrimidin-4-yl trifluoromethanesulfonate (**16**) (77.66 mg, 0.20 mmol) and 4-nitroaniline (30.39 mg, 0.22 mmol). 66.3 mg (0.176 mmol, 88%) of spectroscopically pure 4-((4-nitrophenyl)amino)-2-(phenylamino)-5,6-dihydropyrido[2,3-*d*]pyrimidin-7(8*H*)-one (**19f**) were obtained as a brownish solid. m.p. >250°C. ^{1}H-NMR (400 MHz, DMSO-d_6) δ (ppm): 10.43 (s, 1H, N8-H), 9.12 (s, 1H, N9-H), 9.00 (s, 1H, N14-H), 8.18 – 8.11 (m, 2H, C17-H), 8.06 – 7.99 (m, 2H, C16-H), 7.78 – 7.71 (m, 2H, C11-H), 7.28 – 7.19 (m, 2H, C12-H), 6.95 – 6.90 (m, 1H, C13-H), 2.86 (dd, *J* = 8.3, 6.9 Hz, 2H, C5-H), 2.58 (dd, *J* = 8.3, 6.9 Hz, 2H, C6-H). ^{13}C-NMR (100.6 MHz, DMSO-d_6) δ (ppm): 171.2 (C7), 158.0 (C8a), 157.5 (C2), 156.8 (C4), 147.2 (C15), 140.7 (C10), 140.6 (C18), 128.3 (C12), 124.4 (C17), 121.1 (C13), 119.5 (C16), 119.2 (C11), 90.1 (C4a), 30.2 (C6), 17.4 (C5). IR (KBr) ν (cm^{-1}): 3425, 3275, 3198, 3125, 2921, 1695, 1600, 1578, 1505, 1348, 1324, 1247, 1218, 1114, 855, 762, 748. HRMS (APCI-FIA-TOF) (m/z) calculated for $C_{19}H_{16}N_6O_3$: 376.1284, $[M]^+$, found: 377.1362, $[M+H]^+$.

1.3.7. Synthesis and characterization of methyl 3-((7-oxo-2-(phenylamino)-5,6,7,8-tetrahydropyrido[2,3-*d*]pyrimidin-4-yl)amino)thiophene-2-carboxylate (19g)

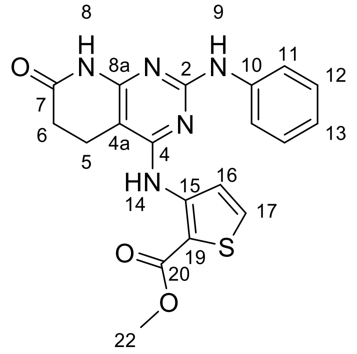

As above but carried out by using 7-oxo-2-(phenylamino)-5,6,7,8-tetrahydropyrido[2,3-*d*]pyrimidin-4-yl trifluoromethanesulfonate (**16**) (77.66 mg, 0.20 mmol) and methyl 3-amino-2-thiophenecarboxylate (34.58 mg, 0.22 mmol). 77.1 mg (0.194 mmol, 97%) of spectroscopically pure 3-((7-oxo-2-(phenylamino)-5,6,7,8-tetrahydropyrido[2,3-*d*]pyrimidin-4-yl)amino)thiophene-2-carboxylate (**19g**) were obtained as a light grey solid. m.p. >250°C. ^{1}H-NMR (400 MHz, DMSO-d_6) δ (ppm): 10.48 (s, 1H, N8-H), 9.89 (s, 1H, N14-H), 9.23 (s, 1H, N9-H), 8.56 (d, *J* = 5.5 Hz, 1H, C16-H), 7.92 (d, *J* = 5.5 Hz, 1H, C17-H), 7.80 – 7.75 (m, 2H, C11-H), 7.29 – 7.23 (m, 2H, C12-H), 6.98 – 6.91 (m, 1H, C13-H), 3.86 (s, 3H, C22-H), 2.80 – 2.71 (m, 2H, C5-H), 2.70 – 2.60 (m, 2H, C6-H). ^{13}C-NMR (100.6 MHz, DMSO-d_6) δ (ppm): 170.9 (C7), 164.8 (C20), 157.8 (C2*), 157.4 (C4*), 155.4 (C8a*), 146.7 (C15), 140.7 (C10), 132.9 (C17), 128.4 (C12), 122.8 (C16), 121.1 (C13), 119.1 (C11), 106.3 (C19), 88.2 (C4a), 52.1 (C22), 29.9 (C6), 17.1 (C5). IR (KBr) ν (cm^{-1}): 3433, 3255, 3194, 3103, 2927, 1681, 1607, 1582, 1540, 1385, 1240, 1100, 781. HRMS (APCI-FIA-TOF) (m/z) calculated for $C_{19}H_{17}N_5O_3S$: 395.1052, $[M]^+$, found: 396.1136, $[M+H]^+$.

1.3.8. Synthesis and characterization of 2-(phenylamino)-4-((3-(trifluoromethyl) phenyl)amino)-5,6-dihydropyrido[2,3-*d*]pyrimidin-7(8*H*)-one (19h)

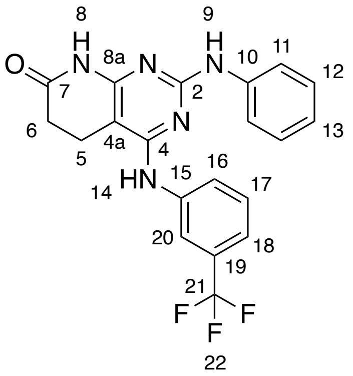

As above but carried out by using 7-oxo-2-(phenylamino)-5,6,7,8-tetrahydropyrido[2,3-*d*]pyrimidin-4-yl trifluoromethanesulfonate (**16**) (77.66 mg, 0.20 mmol) and 3-(Trifluoromethyl)aniline (38 μL, 0.22 mmol). 65.4 mg (0.163 mmol, 81%) of spectroscopically pure 2-(phenylamino)-4-((3-(trifluoromethyl) phenyl)amino)-5,6-dihydropyrido[2,3-*d*]pyrimidin-7(8*H*)-one (**19h**) were obtained as a grey solid. m.p. >250°C. ^{1}H-NMR (400 MHz, DMSO-*d*$_6$) δ (ppm): 10.28 (s, 1H, N8-H), 8.97 (s, 1H, N9-H), 8.62 (s, 1H, N14-H), 8.20 – 8.11 (m, 1H, C16-H), 7.90 – 7.84 (m, 1H, C20-H), 7.72 – 7.66 (m, 2H, C11-H), 7.57 – 7.47 (m, 1H, C17-H), 7.37 – 7.30 (m, 1H, C18-H), 7.21 – 7.12 (m, 2H, C12-H), 6.89 – 6.82 (m, 1H, C13-H), 2.82 (dd, J = 8.3, 7.0 Hz, 2H, C5-H), 2.58 (dd, J = 8.3, 7.0 Hz, 2H, C6-H). ^{13}C-NMR (100.6 MHz, DMSO-*d*$_6$) δ (ppm): 171.2 (C7), 157.6 (C2*), 157.5 (C4*), 157.4 (C8a), 141.1 (C15), 140.8 (C10), 129.4 (C17), 129.1 (q, *J* = 31.6 Hz, C19), 128.1 (C12), 125.0 (C16), 124.2 (q, 273.0 Hz, C21), 120.7 (C13), 118.8 (C11), 118.3 (q, *J* = 3.5 Hz, C18), 117.2 (q, *J* = 3.5 Hz, C20), 88.6 (C4a), 30.2 (C6), 17.3 (C5). IR (KBr) ν (cm^{-1}): 3436, 3278, 3021, 3131, 2921, 1697, 1621, 1581, 1380, 1337, 1243, 1112, 760, 694. HRMS (APCI-FIA-TOF) (m/z) calculated for $C_{20}H_{16}F_3N_5O$: 399.1307, [M]$^+$, found: 400.1383, [M+H]$^+$.

1.3.9. Synthesis and characterization of tert-butyl (4-((7-oxo-2-(phenylamino)-5,6,7,8-tetrahydropyrido[2,3-*d*]pyrimidin-4-yl)amino)benzyl)carbamate (19i)

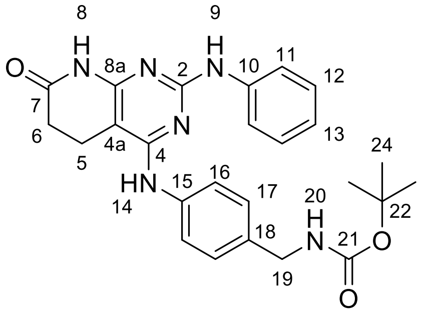

As above but carried out by using 7-oxo-2-(phenylamino)-5,6,7,8-tetrahydropyrido[2,3-*d*]pyrimidin-4-yl trifluoromethanesulfonate (**16**) (77.66 mg, 0.20 mmol) and 4-[(*N*-Boc)aminomethyl]aniline (53.35 mg, 0.24 mmol). 87.2 mg (0.189 mmol, 95%) of spectroscopically pure (4-((7-oxo-2-(phenylamino)-5,6,7,8-tetrahydropyrido[2,3-*d*]pyrimidin-4-yl)amino)benzyl)carbamate (**19i**) were obtained as a brownish solid. m.p. >250°C. ^{1}H-NMR (400 MHz, DMSO-*d*$_6$) δ (ppm): 10.19 (s, 1H, N8-H), 8.87 (s, 1H, N9-H), 8.32 (s, 1H, N14-H), 7.77 – 7.70 (m, 2H, C11-H), 7.61 – 7.53 (m, 2H, C16-H), 7.38 (t, *J* = 6.2 Hz, 1H, N20-H), 7.21 – 7.09 (m, 4H, C17-H, C12-H), 6.88 – 6.78 (m, 1H, C13-H), 4.10 (d, *J* = 6.2 Hz, 2H, C19-H), 2.79 (dd, *J* = 8.3, 7.0 Hz, 2H, C5-H), 2.56 (dd, *J* = 8.3, 7.0 Hz, 2H, C6-H), 1.41 (s, 9H, C24-H). ^{13}C-NMR (100.6 MHz, DMSO-*d*$_6$) δ (ppm): 171.3 (C7), 158.0 (C4), 157.6 (C2), 156.9 (C8a), 155.8 (C21), 141.0 (C10), 138.6 (C15), 134.3 (C18), 128.1 (C12), 126.8 (C17), 122.0 (C16), 120.4 (C13), 118.7 (C11), 87.8 (C4a), 77.7 (C23), 43.0 (C19), 30.3 (C6), 28.3 (C24), 17.3 (C5). IR (ATR) ν (cm^{-1}): 3366, 3274, 3125, 2932, 1681, 1573, 1509, 1420, 1385, 1250, 1169, 1116, 1015, 772. HRMS (APCI-FIA-TOF) (m/z) calculated for $C_{25}H_{28}N_6O_3$: 460.2223, [M]$^+$, found: 461.2284, [M+H]$^+$.

1.3.10. Synthesis and characterization of 4-((3-nitrophenyl)amino)-2-(phenylamino)-5,6-dihydropyrido[2,3-*d*]pyrimidin-7(8*H*)-one (19j)

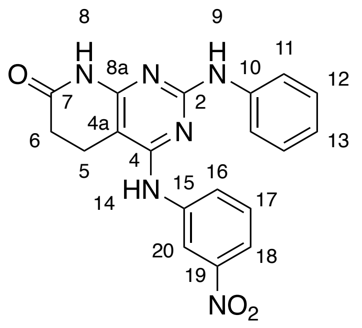

As above but carried out by using 7-oxo-2-(phenylamino)-5,6,7,8-tetrahydropyrido[2,3-*d*]pyrimidin-4-yl trifluoromethanesulfonate (**16**) (77.66 mg, 0.20 mmol) and 3-nitroaniline (30.39 mg, 0.22 mmol). 70.6 mg (0.187 mmol, 94%) of spectroscopically pure 4-((3-nitrophenyl)amino)-2-(phenylamino)-5,6-dihydropyrido[2,3-*d*]pyrimidin-7(8*H*)-one (**19j**) were obtained as a yellowish solid. m.p. >250°C. ^{1}H-NMR (400 MHz, DMSO-d_6) δ (ppm): 10.33 (s, 1H, N8-H), 9.01 (s, 1H, N9-H), 8.82 (s, 1H, N14-H), 8.47 – 8.39 (m, 1H, C20-H), 8.32 – 8.26 (m, 1H, C16-H), 7.86 – 7.83 (m, 1H, C18-H), 7.72 – 7.68 (m, 2H, C11-H), 7.59 – 7.54 (m, 1H, C17-H), 7.17 – 7.12 (m, 2H, C12-H), 6.90 – 6.84 (m, 1H, C13-H), 2.83 (dd, *J* = 8.3, 7.0 Hz, 2H, C5-H), 2.59 (dd, *J* = 8.3, 7.0 Hz, 2H, C6-H). ^{13}C-NMR (100.6 MHz, DMSO-d_6) δ (ppm): 171.3 (C7), 157.6 (C2*), 157.6 (C4*), 157.5 (C8a*), 147.8 (C19), 141.6 (C15), 140.7 (C10), 129.5 (C17), 128.1 (C12), 127.3 (C16), 120.9 (C13), 119.0 (C11), 116.3 (C18), 115.3 (C20), 88.8 (C4a), 30.2 (C6), 17.4 (C5). IR (ATR) ν (cm^{-1}): 3421, 3286, 3138, 2925, 1687, 1622, 1518, 1475, 1455, 1425, 1347, 1294, 1247, 1219, 833, 757, 732. HRMS (APCI-FIA-TOF) (m/z) calculated for $C_{19}H_{16}N_6O_3$: 376.1284, [M]$^+$, found: 377.1354, [M+H]$^+$.

1.3.11. Synthesis and characterization of 4-((3-aminophenyl)amino)-2-(phenylamino)-5,6-dihydropyrido[2,3-*d*]pyrimidin-7(8*H*)-one (19k)

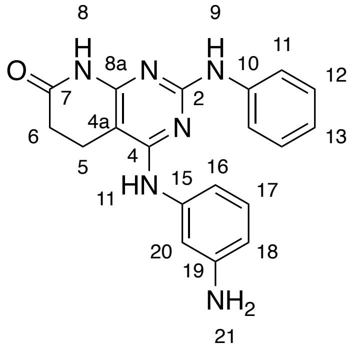

150.1 mg (0.4 mmol, 1 eq) of 4-((3-nitrophenyl)amino)-2-(phenylamino)-5,8-dihydropyrido[2,3-*d*]pyrimidin-7(6*H*)-one (**19j**) were dissolved in 3 mL of ethyl acetate, then 0.3 mL (3.4 mmol, 8.5 eq) of concentrated hydrochloric acid were added dropwise. Finally, 491.9 mg (2.15 mmol, 5.4 eq) of tin(II) chloride dihydrate were added into the round-bottom flask. The resulting mixture was stirred and heated at 60°C for 6h. Upon completion, the solvent was evaporated in vacuo and water (50 mL) was added to the residue and the solid was collected by filtration and washed with water, ethanol and diethyl ether in order to afford 127.8 mg (0.366 mmol, 91%) of spectroscopically pure 4-((3-aminophenyl)amino)-2-(phenylamino)-5,6-dihydropyrido[2,3-*d*]pyrimidin-7(8*H*)-one (**19k**) as a black solid. m.p. >250°C. ^{1}H-NMR (400 MHz, DMSO-d_6) δ (ppm): 10.13 (s, 1H, N8-H), 8.77 (s, 1H, N9-H), 8.09 (s, 1H, N14-H), 7.85 – 7.70 (m, 2H, C11-H), 7.19 – 7.12 (m, 2H, C12-H), 6.97 – 6.91 (m, 1H, C17-H), 6.88 – 6.86 (m, 1H, C20-H), 6.86 – 6.81 (m, 1H, C13-H), 6.80 – 6.77 (m, 1H, C16-H), 6.30 – 6.27 (m, 1H, C18-H), 4.93 (s, 2H, N21-H), 2.76 (dd, *J* = 8.4, 7.0 Hz, 2H, C5-H), 2.54 (dd, *J* = 8.4, 7.0 Hz, 2H, C6-H). ^{13}C-NMR (100.6 MHz, DMSO-d_6) δ (ppm): 171.3 (C7), 158.3 (C4), 157.6 (C2), 156.7 (C8a), 148.6 (C19), 141.1 (C10), 140.6 (C15), 128.4 (C17), 128.2 (C12), 120.3 (C13), 118.6 (C11), 110.5 (C16), 108.9 (C18), 108.2 (C20), 87.8 (C4a), 30.3 (C6), 17.3 (C5). IR (ATR) ν (cm^{-1}): 32813202, 3129, 1680, 1599, 1575, 1545, 1497, 1442, 1376, 1294, 1240, 1215, 1161, 746. HRMS (APCI-FIA-TOF) (m/z) calculated for $C_{19}H_{18}N_6O$: 346.1542, [M]$^+$, found: 347.1609, [M+H]$^+$.

1.4. General procedure for C4 aryloxy substituted products 20

Intermediate 4-iodo-2-(phenylamino)-5,6-dihydropyrido[2,3-*d*]pyrimidin-7(8*H*)-one (**17**) (1 eq), tripotassium phosphate (2 eq), copper(I) iodide (0.05 eq), 2-picolinic acid (0.1 eq) and the corresponding phenol (1.2 eq) were introduced under Argon atmosphere into a Schlenk tube. After that anhydrous DMSO (1.4 mL) was added and the mixture was heated overnight at 80°C. Then, water was added to the residue and the solid was collected by filtration and washed with more water, ethanol, and diethyl ether to afford the spectroscopically pure product.

1.4.1. Synthesis and characterization of 4-phenoxy-2-(phenylamino)-5,6-dihydropyrido[2,3-*d*]pyrimidin-7(8*H*)-one (20a)

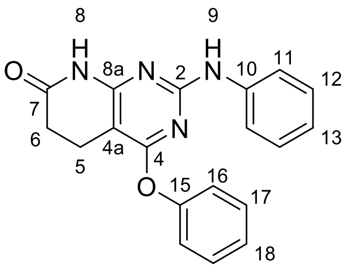

As above but carried out by using 4-iodo-2-(phenylamino)-5,6-dihydropyrido[2,3-*d*]pyrimidin-7(8*H*)-one (**17**) (73.23 mg, 0.20 mmol) and phenol (22.59 mg, 0,24 mmol). The mixture reaction was heated at 80°C during 48h. 41.7 mg (0.125 mmol, 64%) of spectroscopically pure 4-phenoxy-2-(phenylamino)-5,6-dihydropyrido[2,3-*d*]pyrimidin-7(8*H*)-one (**20a**) were obtained as a greyish solid. m.p. >250°C. ^{1}H-NMR (400 MHz, DMSO-*d*$_6$) δ (ppm): 10.60 (s, 1H, N8-H), 9.20 (s, 1H, N9-H), 7.53 – 7.41 (m, 4H, C11-H, C17-H), 7.33 – 7.24 (m, 1H, C18-H), 7.26 – 7.17 (m, 2H, C16-H), 7.06 – 6.98 (m, 2H, C12-H), 6.85 – 6.76 (m, 1H, C13-H), 2.85 (dd, *J* = 8.2, 7.1 Hz, 2H, C5-H), 2.61 (dd, *J* = 8.2, 7.1 Hz, 2H, C6-H). ^{13}C-NMR (100.6 MHz, DMSO-*d*$_6$) δ (ppm): 171.5 (C7), 165.9 (C4*), 159.6 (C8a*), 157.4 (C2), 153.0 (C15), 140.4 (C10), 129.5 (C17), 128.0 (C12), 125.0 (C18), 121.9 (C16), 120.8 (C13), 118.2 (C11), 89.9 (C4a), 30.2 (C6), 16.4 (C5). IR (ATR) ν (cm^{-1}): 3284, 3203, 3138, 2968, 1683, 1618, 1577, 1549, 1441, 1401, 1349, 1236, 1199, 750, 688. HRMS (APCI-FIA-TOF) (m/z) calculated for $C_{19}H_{16}N_4O_2$: 332.1273, [M]$^+$, found: 333.1345, [M+H]$^+$.

1.4.2. Synthesis and characterization of 2-(phenylamino)-4-(pyridin-4-yloxy)-5,6-dihydropyrido[2,3-*d*]pyrimidin-7(8*H*)-one (20b)

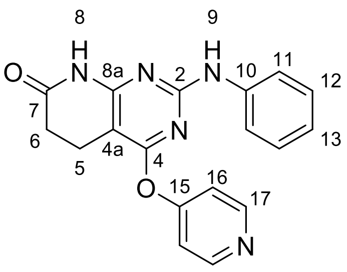

As above but carried out by using 4-iodo-2-(phenylamino)-5,6-dihydropyrido[2,3-*d*]pyrimidin-7(8*H*)-one (**17**) (73.23 mg, 0.20 mmol) and 4-hydroxypyridine (22.82 mg, 0,24 mmol). 44.9 mg (0.134 mmol, 68%) of spectroscopically pure 2-(phenylamino)-4-(pyridin-4-yloxy)-5,6-dihydropyrido[2,3-*d*]pyrimidin-7(8*H*)-one (**20b**) were obtained as a beige solid. m.p. >250°C. ^{1}H-NMR (400 MHz, DMSO-*d*$_6$) δ (ppm): 11.04 (s, 1H, N8-H), 9.72 (s, 1H, N9-H), 8.01 – 7.93 (m, 2H, C17-H), 7.82 – 7.73 (m, 2H, C11-H), 7.31 – 7.22 (m, 2H, C12-H), 7.01 – 6.91 (m, 1H, C13-H), 6.30 – 6.19 (m, 2H, C17-H), 2.78 (dd, *J* = 8.4, 6.6 Hz, 2H, C5-H), 2.57 (dd, *J* = 8.4, 6.6 Hz, 2H, C6-H). ^{13}C-NMR (100.6 MHz, DMSO-*d*$_6$) δ (ppm): 177.8 (C15), 171.4 (C7), 161.1 (C8a), 157.9 (C2), 157.0 (C4), 140.0 (C10), 138.9 (C17), 128.5 (C12), 121.7 (C13), 119.1 (C11), 117.2 (C16), 97.9 (C4a), 30.1 (C6), 18.9 (C5). IR (ATR) ν (cm^{-1}): 3280, 3205, 3140, 2975, 1667, 1619, 1597, 1574, 1543, 1439, 1346, 1302, 1225, 1182, 1153, 845, 756. HRMS (APCI-FIA-TOF) (m/z) calculated for $C_{18}H_{15}N_5O_2$: 333.1226, [M]$^+$, found: 334.1288, [M+H]$^+$.

1.4.3. Synthesis and characterization of 4-((7-oxo-2-(phenylamino)-5,6,7,8-tetrahydropyrido[2,3-*d*]pyrimidin-4-yl)oxy)benzonitrile (**20c**)

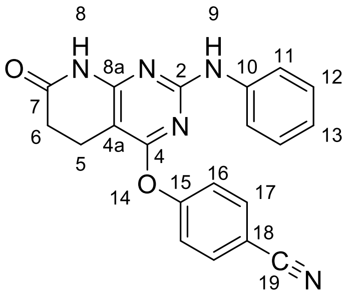

As above but carried out by using 4-iodo-2-(phenylamino)-5,6-dihydropyrido[2,3-*d*]pyrimidin-7(8*H*)-one (**17**) (73.23 mg, 0.20 mmol) and 4-hydroxybenzonitrile (28.59 mg, 0,24 mmol). 55.9 mg (0.156 mmol, 78%) of spectroscopically pure 4-((7-oxo-2-(phenylamino)-5,6,7,8-tetrahydropyrido[2,3-*d*]pyrimidin-4-yl)oxy)benzonitrile (**20c**) were obtained as a brown solid. m.p. >250°C. ^{1}H-NMR (400 MHz, DMSO-d_6) δ (ppm): 10.70 (s, 1H, N8-H), 9.29 (s, 1H, N9-H), 8.01 – 7.92 (m, 2H, C17-H), 7.52 – 7.48 (m, 2H, C11-H), 7.48 – 7.43 (m, 2H, C16-H), 7.11 – 7.03 (m, 2H, C12-H), 6.89 – 6.83 (m, 1H, C13-H), 2.84 (dd, *J* = 8.3, 7.1 Hz, 2H, C5-H), 2.60 (dd, *J* = 8.3, 7.1 Hz, 2H, C6-H). ^{13}C-NMR (100.6 MHz, DMSO-d_6) δ (ppm): 171.5 (C7), 165.2 (C4*), 160.0 (C8a*), 157.3 (C2), 156.9 (C15), 140.2 (C10), 134.0 (C17), 128.1 (C12), 123.1 (C16), 121.2 (C13), 118.6 (C11), 118.5 (C19), 107.6 (C18), 90.5 (C4a), 30.2 (C6), 16.4 (C5). IR (ATR) ν (cm^{-1}): 3281, 3203, 3147, 2966, 2235, 1686, 1618, 1599, 1576, 1550, 1443, 1348, 1309, 1206, 1169, 1099, 841, 754. HRMS (APCI-FIA-TOF) (m/z) calculated for $C_{20}H_{15}N_5O_2$: 357.1226, [M]$^+$, found: 358.1299, [M+H]$^+$.

1.4.4. Synthesis and characterization of 4-(2-fluorophenoxy)-2-(phenylamino)-5,6-dihydropyrido[2,3-*d*]pyrimidin-7(8*H*)-one (**20d**)

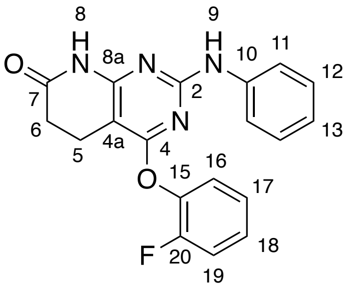

As above but carried out by using 4-iodo-2-(phenylamino)-5,6-dihydropyrido[2,3-*d*]pyrimidin-7(8*H*)-one (**17**) (73.23 mg, 0.20 mmol) and 2-fluorophenol (22 μL, 0,24 mmol). 62.9 mg (0.180 mmol, 90%) of spectroscopically pure 4-(2-fluorophenoxy)-2-(phenylamino)-5,6-dihydropyrido[2,3-*d*]pyrimidin-7(8*H*)-one (**20d**) were obtained as a brownish solid. m.p. >250°C. ^{1}H-NMR (400 MHz, DMSO-d_6) δ (ppm): 10.64 (s, 1H, N8-H), 9.25 (s, 1H, N9-H), 7.45 – 7.33 (m, 5H, C11-H, C19-H, C18-H, C16-H), 7.31 – 7.26 (m, 1H, C17-H), 7.04 – 6.98 (m, 2H, C12-H), 6.83 – 6.78 (m, 1H, C13-H), 2.87 (dd, *J* = 8.3, 7.1 Hz, 2H, C5-H), 2.62 (dd, *J* = 8.3, 7.1 Hz, 2H, C6-H). ^{13}C-NMR (100.6 MHz, DMSO-d_6) δ (ppm): 171.5 (C7), 165.2 (C4*), 159.8 (C8a*), 157.3 (C2), 154.1 (d, *J* = 246.2 Hz, C20), 140.2 (C10), 140.0 (d, *J* = 12.4 Hz, C15), 128.0 (C12), 126.9 (d, *J* = 7.1 Hz, C16**), 125.1 (d, *J* = 3.7 Hz, C17), 124.5 (C18**), 121.0 (C13), 118.4 (C11), 116.7 (d, *J* = 18.3 Hz, C19), 89.3 (C4a), 30.2 (C6), 16.3 (C5). IR (ATR) ν (cm^{-1}): 3285, 3143, 2970, 1683, 1620, 1581, 1552, 1496, 1444, 1350, 1254, 1210, 1107, 751, 693. HRMS (APCI-FIA-TOF) (m/z) calculated for $C_{19}H_{15}FN_4O_2$: 350.1179, [M]$^+$, found: 351.1258, [M+H]$^+$.

1.4.5. Synthesis and characterization of 4-(3,5-dimethoxyphenoxy)-2-(phenylamino)-5,6-dihydropyrido[2,3-*d*]pyrimidin-7(8*H*)-one (20e)

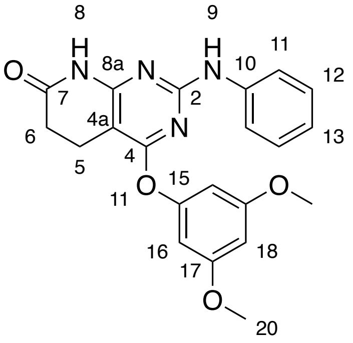

As above but carried out by using 4-iodo-2-(phenylamino)-5,6-dihydropyrido[2,3-*d*]pyrimidin-7(8*H*)-one (**17**) (73.23 mg, 0.20 mmol) and 3,5-dimethoxyphenol (36.99 mg, 0.24 mmol). 53.6 mg (0.136 mmol, 68%) of spectroscopically pure 4-(3,5-dimethoxyphenoxy)-2-(phenylamino)-5,6-dihydropyrido[2,3-*d*]pyrimidin-7(8*H*)-one (**20e**) were obtained as a beige solid. m.p. >250°C. ^{1}H-NMR (400 MHz, DMSO-*d*$_6$) δ (ppm): 10.60 (s, 1H, N8-H), 9.24 (s, 1H, N9-H), 7.60 – 7.56 (m, 2H, C11-H), 7.09 – 7.03 (m, 2H, C12-H), 6.86 – 6.81 (m, 1H, C13-H), 6.44 – 6.40 (m, 3H, C18-H, C16-H), 3.74 (s, 6H, C20-H), 2.82 (dd, *J* = 8.4, 7.3 Hz, 2H, C5-H), 2.59 (dd, *J* = 8.4, 7.3 Hz, 2H, C6-H). ^{13}C-NMR (100.6 MHz, DMSO-*d*$_6$) δ (ppm): 171.5 (C7), 165.8 (C4*), 160.9 (C17), 159.6 (C8a*), 157.4 (C2), 154.7 (C15), 140.4 (C10), 128.0 (C12), 120.9 (C13), 118.5 (C11), 100.5 (C16), 97.3 (C18), 90.0 (C4a), 55.4 (C20), 30.3 (C6), 16.4 (C5). IR (ATR) ν (cm^{-1}): 3280, 3202, 3140, 2963, 1679, 1598, 1575, 1550, 1442, 1400, 1348, 1204, 1142, 1047, 751. HRMS (APCI-FIA-TOF) (m/z) calculated for $C_{21}H_{20}N_4O_4$: 392.1485, [M]$^+$, found: 393.1553, [M+H]$^+$.

1.4.6. Synthesis and characterization of 2-(phenylamino)-4-(3-(trifluoromethyl)phenoxy)-5,6-dihydropyrido[2,3-*d*]pyrimidin-7(8*H*)-one (20f)

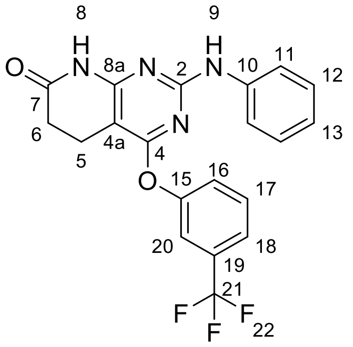

As above but carried out by using 4-iodo-2-(phenylamino)-5,6-dihydropyrido[2,3-*d*]pyrimidin-7(8*H*)-one (**17**) (73.23 mg, 0.20 mmol) and *m*-(Trifluoromethyl)phenol (30 µL, 0,24 mmol). 47.5 mg (0.119 mmol, 60%) of spectroscopically pure 2-(phenylamino)-4-(3-(trifluoromethyl)phenoxy)-5,6-dihydropyrido[2,3-*d*]pyrimidin-7(8*H*)-one (**20f**) were obtained as a beige solid. m.p. >250°C. ^{1}H-NMR (400 MHz, DMSO-*d*$_6$) δ (ppm): 10.65 (s, 1H, N8-H), 9.26 (s, 1H, N9-H), 7.74 – 7.63 (m, 3H, C17-H, C18-H, C20-H), 7.60 – 7.54 (m, 1H, C16-H), 7.48 – 7.39 (m, 2H, C11-H), 7.03 – 6.98 (m, 2H, C12-H), 6.85 – 6.78 (m, 1H, C13-H), 2.87 (dd, *J* = 8.3, 7.1 Hz, 2H, C5-H), 2.61 (dd, *J* = 8.3, 7.1 Hz, 2H, C6-H). ^{13}C-NMR (100.6 MHz, DMSO-*d*$_6$) δ (ppm): 171.5 (C7), 165.6 (C4*), 159.8 (C8a*), 157.3 (C2), 153.3 (C15), 140.2 (C10), 130.9 (C17), 130.3 (q, *J* = 32.1 Hz, C19), 127.9 (C12), 126.4 (C16), 123.8 (q, *J* = 272.6 Hz, C21), 121.8 (q, *J* = 3.01 Hz, C18**), 121.0 (C13), 119.1 (q, *J* = 5.03Hz, C20**), 118.5 (C11), 90.1 (C4a), 30.2 (C6), 16.4 (C5). IR (ATR) ν (cm^{-1}): 3295, 3211, 3150, 2926, 2850, 1689, 1620, 1600, 1579, 1551, 1499, 1444, 1349, 1326, 1235, 1206, 1126, 897, 798, 752, 696. HRMS (APCI-FIA-TOF) (m/z) calculated for $C_{20}H_{15}F_3N_4O_2$: 400.1147, [M]$^+$, found: 401.1222, [M+H]$^+$.

1.4.7. Synthesis and characterization of 4-(4-bromo-2-fluorophenoxy)-2-(phenylamino)-5,6-dihydropyrido[2,3-*d*]pyrimidin-7(8*H*)-one (20g)

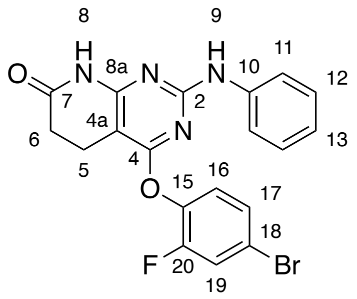

As above but carried out by using 4-iodo-2-(phenylamino)-5,6-dihydropyrido[2,3-*d*]pyrimidin-7(8*H*)-one (**17**) (73.23 mg, 0.20 mmol) and 4-bromo-2-fluorophenol (27 µL, 0,24 mmol). 64.5 mg (0.150 mmol, 75%) of spectroscopically pure 4-(4-bromo-2-fluorophenoxy)-2-(phenylamino)-5,6-dihydropyrido[2,3-*d*]pyrimidin-7(8*H*)-one (**20g**) were obtained as a white solid. m.p. >250°C. ^{1}H-NMR (400 MHz, DMSO-*d*$_6$) δ (ppm): 10.67 (s, 1H, N8-H), 9.29 (s, 1H, N9-H), 7.84 – 7.76 (m, 1H, C19-H), 7.56 – 7.48 (m, 1H, C16-H), 7.46 – 7.34 (m, 3H, C11-H, C17-H), 7.09 – 7.00 (m, 2H, C12-H), 6.89 – 6.80 (m, 1H, C13-H), 2.86 (dd, *J* = 8.2, 7.1 Hz, 2H, C5-H), 2.62 (dd, *J* = 8.2, 7.2 Hz, 2H, C6-H). ^{13}C-NMR (100.6 MHz, DMSO-*d*$_6$) δ (ppm): 171.5 (C7), 164.9 (C4*), 159.9 (C8a*), 157.2 (C2), 154.3 (d, *J* = 251.1 Hz, C20), 140.2 (C10), 139.6 (d, *J* = 12.4 Hz, C15), 128.25 (d, *J* = 3.5 Hz, C16), 128.0 (C12), 126.3 (C17), 121.1 (C13), 120.1 (d, *J* = 21.8 Hz, C19), 118.5 (C11), 117.7 (d, *J* = 8.7 Hz, C18), 89.3 (C4a), 30.1 (C6), 16.3 (C5). IR (ATR) ν (cm^{-1}): 3280, 3205, 3145, 2963, 1683, 1619, 1579, 1551, 1488, 1444, 1350, 1235, 1189, 889, 818, 757, 691. HRMS (APCI-FIA-TOF) (m/z) calculated for $C_{19}H_{14}BrFN_4O_2$: 428.0284, [M]$^+$, found: 429.0358, [M+H]$^+$.

1.4.8. Synthesis and characterization of 4-(3-nitrophenoxy)-2-(phenylamino)-5,6-dihydropyrido[2,3-*d*]pyrimidin-7(8*H*)-one (20h)

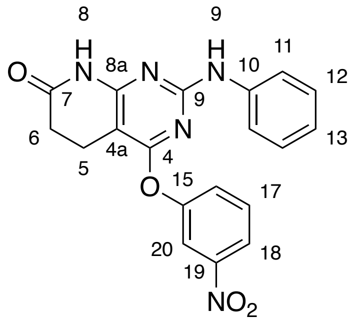

As above but carried out by using 4-iodo-2-(phenylamino)-5,6-dihydropyrido[2,3-*d*]pyrimidin-7(8*H*)-one (**17**) (73.23 mg, 0.20 mmol) and 3-nitrophenol (23 µL mg, 0,24 mmol). 56.7 mg (0.150 mmol, 75%) of spectroscopically pure 4-(3-nitrophenoxy)-2-(phenylamino)-5,6-dihydropyrido[2,3-*d*]pyrimidin-7(8*H*)-one (**20h**) were obtained as a yellowish solid. m.p. >250°C. ^{1}H-NMR (400 MHz, DMSO-*d*$_6$) δ (ppm): 10.69 (s, 1H, N8-H), 9.28 (s, 1H, N9-H), 8.21 – 8.14 (m, 1H, C18-H), 8.15 – 8.10 (m, 1H, C20-H), 7.80 – 7.73 (m, 2H, C16-H, C17-H), 7.49 – 7.43 (m, 2H, C11-H), 7.06 – 6.97 (m, 2H, C12-H), 6.86 – 6.78 (m, 1H, C13-H), 2.87 (dd, *J* = 8.3, 7.1 Hz, 2H, C5-H), 2.62 (dd, *J* = 8.3, 7.1 Hz, 2H, C6-H). ^{13}C-NMR (100.6 MHz, DMSO-*d*$_6$) δ (ppm): 171.5 (C7), 165.3 (C4*), 159.9 (C8a*), 157.2 (C2), 153.3 (C15), 148.4 (C19), 140.1 (C10), 130.8 (C16), 129.1 (C17), 128.0 (C12), 121.2 (C13), 120.0 (C18), 118.7 (C11), 117.4 (C20), 90.2 (C4a), 30.2 (C6), 16.4 (C5). IR (ATR) ν (cm^{-1}): 3280, 3202, 3145, 2963, 1683, 1620, 1578, 1552, 1519, 1445, 1395, 1342, 1236, 1208, 1105, 828, 753, 735. HRMS (APCI-FIA-TOF) (m/z) calculated for $C_{19}H_{15}N_5O_4$: 377.1124, [M]$^+$, found: 378.1197, [M+H]$^+$.

1.4.9. Synthesis and characterization of 4-(3-aminophenoxy)-2-(phenylamino)-5,6-dihydropyrido[2,3-*d*]pyrimidin-7(8*H*)-one (20i)

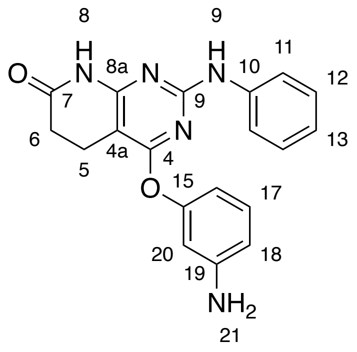

100 mg (0.264 mmol, 1 eq) of 4-(3-nitrophenoxy)-2-(phenylamino)-5,6-dihydropyrido[2,3-*d*]pyrimidin-7(8*H*)-one (**20h**) were dissolved in 2 mL of ethyl acetate, then 0.2 mL (2.24 mmol, 8.5 eq) of hydrochloric acid were added dropwise. Finally, 326.67 mg (1.43 mmol, 5.4 eq) of tin(II) chloride dihydrate were added into the round-bottom flask. The resulting mixture was stirred and heated at 60°C for 6h. Upon completion, the solvent was evaporated in vacuo and water (50 mL) was added to the residue and the solid was collected by filtration and washed with water, ethanol and diethyl ether in order to afford 83.5 mg (0.240 mmol, 91%) of spectroscopically pure 4-(3-aminophenoxy)-2-(phenylamino)-5,6-dihydropyrido[2,3-*d*]pyrimidin-7(8*H*)-one (**20i**) as a orangish solid. m.p. >250°C. ^{1}H-NMR (400 MHz, DMSO-*d*$_6$) δ (ppm): 10.56 (s, 1H, N8-H), 9.20 (s, 1H, N9-H), 7.64 – 7.57 (m, 2H, C11-H), 7.12 – 7.01 (m, 3H, C12-H, C17-H), 6.86 – 6.77 (m, 1H, C13-H), 6.49 – 6.42 (m, 1H, C16-H), 6.38 – 6.32 (m, 1H, C20-H), 6.33 – 6.26 (m, 1H, C18-H), 5.24 (s, 2H, N21-H), 2.80 (dd, *J* = 8.3, 7.1 Hz, 2H, C5-H), 2.59 (dd, *J* = 8.3, 7.1 Hz, 2H, C6-H). ^{13}C-NMR (100.6 MHz, DMSO-*d*$_6$) δ (ppm): 171.5 (C7), 166.1 (C4*), 159.4 (C8a*), 157.5 (C2), 154.1 (C15), 150.1 (C19), 140.5 (C10), 129.5 (C17), 128.1 (C12), 120.8 (C13), 118.5 (C11), 110.6 (C16), 108.6 (C18), 106.9 (C20), 90.0 (C4a), 30.3 (C6), 16.5 (C5). IR (ATR) ν (cm^{-1}): 3373, 3281, 3200, 3149, 2965, 1685, 1618, 1575, 1549, 1488, 1442, 1400, 1349, 1236, 1207, 1148, 996, 951, 752. HRMS (APCI-FIA-TOF) (m/z) calculated for $C_{19}H_{17}N_5O_2$: 347.1382, [M]$^+$, found: 348.1449, [M+H]$^+$.

1.4.10. Synthesis and characterization of *N*-(3-((7-oxo-2-(phenylamino)-5,6,7,8-tetrahydropyrido[2,3-*d*]pyrimidin-4-yl)oxy)phenyl)acrylamide (20j)

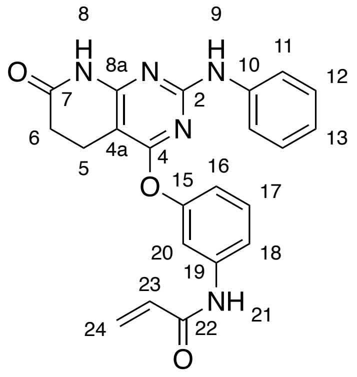

30.0 mg (0.086 mmol, 1 eq) of 4-(3-aminophenoxy)-2-(phenylamino)-5,6-dihydropyrido[2,3-*d*]pyrimidin-7(8*H*)-one (**20i**) were stirred in THF (1 mL) for 10 min. Then a saturated solution of $NaHCO_3$ (1mL) was added and the resulting mixture was stirred for another 10 min at 0°C in an ice bath. Finally 8 μL (0.099 mmol, 1.15 eq) of acryloyl chloride were added dropwise and the resulting mixture was stirred at 0°C for 30 min. The organic solvent was evaporated in vacuo and water (40 mL) was added. The solid appeared was collected by filtration and washed with more water, ethanol and diethyl ether in order to afford 27.1 mg (0.067 mmol, 78%) of spectroscopically pure *N*-(3-((7-oxo-2-(phenylamino)-5,6,7,8-tetrahydropyrido[2,3-*d*]pyrimidin-4-yl)oxy)phenyl) acrylamide (**20j**) as a white solid. m.p. >250°C. ^{1}H-NMR (400 MHz, DMSO-*d*$_6$) δ (ppm): 10.61 (s, 1H, N8-H), 10.29 (s, 1H, N21-H), 9.22 (s, 1H, N9-H), 7.66 – 7.63 (m, 1H, C20-H), 7.55 – 7.48 (m, 3H, C18-H, C11-H), 7.43 – 7.37 (m, 1H, C17-H), 7.06 – 7.00 (m, 2H, C12-H), 6.95 – 6.90 (m, 1H, C16-H), 6.83 – 6.77 (m, 1H, C13-H), 6.42 (dd, *J* = 17.0, 10.1 Hz, 1H, C23-H), 6.25 (dd, *J* = 17.0, 2.0 Hz, 1H, C24-H), 5.76 (dd, *J* = 10.1, 2.0 Hz, 1H, C24-H), 2.85 (dd, *J* = 8.3, 7.1 Hz, 2H, C5-H), 2.61 (dd, *J* = 8.3, 7.1 Hz, 2H, C6-H). ^{13}C-NMR (100.6 MHz, DMSO-*d*$_6$) δ (ppm): 171.5 (C7),

165.8 (C4*), 163.2 (C22), 159.7 (C8a*), 157.4 (C2), 153.2 (C15), 140.4 (C10), 140.1 (C19), 131.6 (C23), 129.6 (C17), 128.0 (C12), 127.2 (C24), 120.9 (C13), 118.6 (C11), 116.9 (C16), 115.8 (C18), 112.8 (C20), 90.0 (C4a), 30.2 (C6), 16.4 (C5). IR (KBr) ν (cm^{-1}): 3423, 3287, 3207, 3148, 2926, 2853, 1691, 1623, 1602, 1580, 1551, 1445, 1401, 1236, 1207, 1153, 790, 750, 688. HRMS (APCI-FIA-TOF) (m/z) calculated for $C_{22}H_{19}N_5O_3$: 401.1488, $[M]^+$, found: 402.1563, $[M+H]^+$.

1.5. General procedure for C4 aryl sulfides substituted products 21

Intermediate 4-iodo-2-(phenylamino)-5,6-dihydropyrido[2,3-*d*]pyrimidin-7(8*H*)-one (**17**) (1 eq), tripotassium phosphate (2 eq), copper(I) iodide (0.05 eq), 2-picolinic acid (0.1 eq) and the corresponding tiophenol (1.2 eq) were introduced under Argon atmosphere into a Schlenk tube. After that anhydrous DMSO (1.4 mL) was added and the mixture was heated overnight at 90°C. Then, water was added to the residue and the solid was collected by filtration and washed with more water, ethanol and diethyl ether. As it was observed that thiophenol tends to form a dimer, the impure product was washed with DCM in order to afford the spectroscopically pure product.

1.5.1. Synthesis and characterization of 4-((4-nitrophenyl)thio)-2-(phenylamino)-5,6-dihydropyrido[2,3-*d*]pyrimidin-7(8*H*)-one (21a)

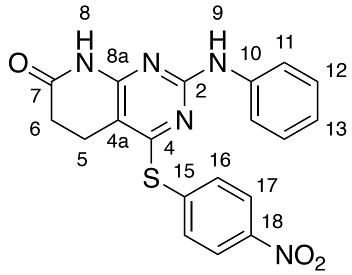

As above but carried out by using 4-iodo-2-(phenylamino)-5,6-dihydropyrido[2,3-*d*]pyrimidin-7(8*H*)-one (**17**) (91.54 mg, 0.25 mmol) and 4-nitrobenzenethiol (46.55 mg, 0,30 mmol). 33.1 mg (0.023 mmol, 92%) of spectroscopically pure 4-((4-nitrophenyl)thio)-2-(phenylamino)-5,6-dihydropyrido[2,3-*d*]pyrimidin-7(8*H*)-one (**21a**) were obtained as an orangish solid. m.p. >250°C. ^{1}H-NMR (400 MHz, DMSO-d_6) δ (ppm): 10.68 (s, 1H, N8-H), 9.23 (s, 1H, N9-H), 8.33 – 8.26 (m, 2H, C17-H), 7.89 – 7.82 (m, 2H, C16-H), 7.32 – 7.25 (m, 2H, C11-H), 6.95 – 6.87 (m, 2H, C12-H), 6.83 – 6.74 (m, 1H, C13-H), 2.81 (dd, *J* = 8.2, 7.0 Hz, 2H, C5-H), 2.63 (dd, *J* = 8.2, 7.0 Hz, 2H, C6-H). ^{13}C-NMR (100.6 MHz, DMSO-d_6) δ (ppm): 171.3 (C7), 163.1 (C2*), 157.5 (C4*), 157.4 (C8a*), 147.6 (C18), 140.1 (C10), 137.9 (C15), 135.9 (C16), 127.8 (C12), 124.0 (C17), 121.1 (C13), 118.5 (C11), 102.7 (C4a), 30.1 (C6), 19.3 (C5). IR (ATR) ν (cm^{-1}): 3274, 3142, 2953, 1679, 1589, 1572, 1561, 1537, 1438, 1423, 1277, 1241, 1028, 845, 821, 768. HRMS (APCI-FIA-TOF) (m/z) calculated for $C_{19}H_{15}N_5O_3S$: 393.0896, $[M]^+$, found: 394.0974, $[M+H]^+$.

1.5.2. Synthesis and characterization of 4-((4-methoxyphenyl)thio)-2-(phenylamino)-5,6-dihydropyrido[2,3-*d*]pyrimidin-7(8*H*)-one (21b)

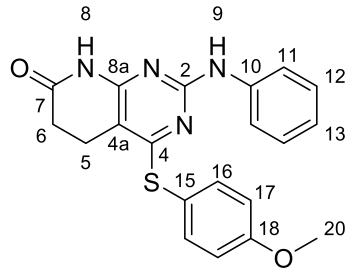

As above but carried out by using 4-iodo-2-(phenylamino)-5,6-dihydropyrido[2,3-*d*]pyrimidin-7(8*H*)-one (**17**) (73.23 mg, 0.20 mmol) and 4-methoxybenzenethiol (30 μL, 0,24 mmol). 33.1 mg (0.087 mmol, 44%) of spectroscopically pure 4-((4-methoxyphenyl)thio)-2-(phenylamino)-5,6-dihydropyrido[2,3-*d*]pyrimidin-7(8*H*)-one (**21b**) were obtained as a greyish solid. m.p. >250°C. ^{1}H-NMR (400 MHz, DMSO-d_6) δ (ppm): 10.50 (s, 1H, N8-H), 9.08 (s, 1H, N9-H), 7.55 – 7.46 (m, 2H, C16-H), 7.26 – 7.19 (m, 2H, C11-H), 7.13 – 7.04 (m, 2H, C17-H), 6.94 – 6.85 (m, 2H, C12-H), 6.81 – 6.72 (m, 1H, C13-H), 3.86 (s, 3H, C20-H), 2.77 (dd, *J* = 8.2, 7.0 Hz, 2H, C5-H), 2.62 (dd, *J* = 8.2, 7.0 Hz, 2H, C6-H). ^{13}C-NMR (100.6 MHz, DMSO-d_6) δ (ppm): 171.3 (C7), 166.0 (C4), 160.5 (C18), 157.2 (C2), 156.6 (C8a), 140.4 (C10), 138.0 (C16), 127.8 (C12), 120.5 (C13), 118.1 (C15), 118.0 (C11), 115.1 (C17), 101.1 (C4a), 55.4 (C20), 30.1 (C6), 18.9 (C5). IR (ATR) ν (cm^{-1}): 3284, 3143, 2933, 1678, 1589, 1560, 1533, 1433, 1246, 1230, 1022, 820, 758. HRMS (APCI-FIA-TOF) (m/z) calculated for $C_{20}H_{18}N_4O_2S$: 378.1150, $[M]^+$, found: 379.1223, $[M+H]^+$.

1.6. General procedure for C4 aryl substituted products 22

Intermediate 4-iodo-2-(phenylamino)-5,6-dihydropyrido[2,3-*d*]pyrimidin-7(8*H*)- one (**17**) (1 eq), cesium carbonate (2.5 eq), tetrakis(triphenylphosphine)palladium(0) (2% molar) and the corresponding boronic acid (1.4 eq) were introduced under Argon atmosphere into a Schlenk tube. After that, a deoxygenated mixture of 1,4-dioxane/water (10:1) (1.5 mL) was added and the resultant reaction mixture was heated overnight at 90°C. Then, water was added (20 mL) to the residue and the solid appeared was collected by filtration and washed with more water, ethanol and diethyl ether to afford the spectroscopically pure product.

1.6.1. Synthesis and characterization of 4-phenyl-2-(phenylamino)-5,6-dihydropyrido[2,3-*d*]pyrimidin-7(8*H*)-one (22a)

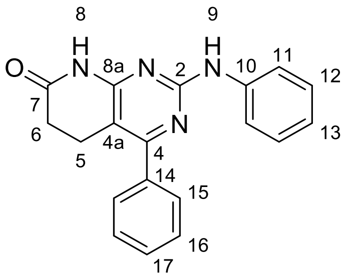

As above but carried out by using 4-iodo-2-(phenylamino)-5,6-dihydropyrido[2,3-*d*]pyrimidin-7(8*H*)-one (**17**) (73.23 mg, 0.20 mmol) and phenylboronic acid (34.14 mg, 0.28 mmol). 37.4 mg (60%, 0.118 mmol) of spectroscopically pure 4-phenyl-2-(phenylamino)-5,6-dihydropyrido[2,3-*d*]pyrimidin-7(8*H*)-one (**22a**) were obtained as a dark grey solid. m.p. >250°C. ^{1}H-NMR (400 MHz, DMSO-d_6) δ (ppm): 10.72 (s, 1H, N8-H), 9.41 (s, 1H, N9-H), 7.90 – 7.81 (m, 2H, C11-H), 7.65 – 7.59 (m, 2H, C15-H), 7.54 – 7.46 (m, 3H, C16-H, C17-H), 7.26 – 7.20 (m, 2H, C12-H), 6.93 – 6.87 (m, 1H, C13-H), 2.85 (dd, *J* = 8.4, 6.6 Hz, 2H, C5-H), 2.53 – 2.50 (m, 2H, C6-H). ^{13}C-NMR (100.6 MHz, DMSO-d_6) δ (ppm): 171.6 (C7), 163.2 (C4), 158.9 (C8a), 158.1 (C2), 140.9 (C10), 137.7 (C14), 129.0 (C17), 128.6 (C15), 128.3 (C12), 128.1 (C16), 120.8 (C13), 118.5 (C11), 103.4 (C4a), 30.7 (C6), 20.5 (C5). IR (ATR) ν (cm^{-1}): 3281, 3198, 3141, 3047, 2904, 1686, 1592, 1544, 1435, 1343, 1303, 1217, 815, 749, 689. HRMS (APCI-FIA-TOF) (m/z) calculated for $C_{19}H_{16}N_4O$: 316.1324, $[M]^+$, found: 317.1392, $[M+H]^+$.

1.6.2. Synthesis and characterization of 2-(phenylamino)-4-(p-tolyl)-5,6-dihydropyrido[2,3-*d*]pyrimidin-7(8*H*)-one (22b)

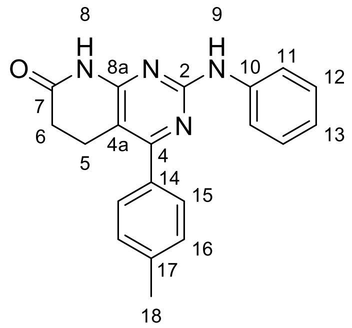

As above but carried out by using 4-iodo-2-(phenylamino)-5,6-dihydropyrido[2,3-*d*]pyrimidin-7(8*H*)-one (**17**) (73.23 mg, 0.20 mmol) and *p*-tolylboronic acid (38.1 mg, 0.28 mmol). 59 mg (0.178 mmol, 89%) of spectroscopically pure 2-(phenylamino)-4-(p-tolyl)-5,6-dihydropyrido[2,3-*d*]pyrimidin-7(8*H*)-one (**22b**) were obtained as a light grey solid. m.p. >250°C. ^{1}H-NMR (400 MHz, DMSO-*d*$_6$) δ (ppm): 10.68 (s, 1H, N8-H), 9.38 (s, 1H, N9-H), 7.90 – 7.82 (m, 2H, C11-H), 7.55 – 7.51 (m, 2H, C15-H), 7.34 – 7.30 (m, 2H, C16-H), 7.26 – 7.19 (m, 2H, C12-H), 6.92 – 6.87 (m, 1H, C13-H), 2.86 (dd, *J* = 8.3, 6.6 Hz, 2H, C5-H), 2.52 – 2.48 (m, 2H, C6-H), 2.38 (s, 3H, C18-H). ^{13}C-NMR (100.6 MHz, DMSO-*d*$_6$) δ (ppm): 171.6 (C7), 163.1 (C4), 158.9 (C8a), 158.0 (C2), 140.9 (C10), 138.6 (C17), 134.9 (C14), 128.7 (C16), 128.6 (C15), 128.3 (C12), 120.8 (C13), 118.5 (C11), 103.3 (C4a), 30.7 (C6), 20.9 (C18), 20.6 (C5). IR (KBr) ν (cm^{-1}): 3282, 3202, 3137, 2953, 1684, 1595, 1560, 1545, 1498, 1438, 1345, 1210, 824, 802, 748. HRMS (APCI-FIA-TOF) (m/z) calculated for $C_{20}H_{18}N_4O$: 330.1481, [M]$^+$, found: 331.1549, [M+H]$^+$.

1.6.3. Synthesis and characterization of 4-(4-methoxyphenyl)-2-(phenylamino)-5,6-dihydropyrido[2,3-*d*]pyrimidin-7(8*H*)-one (22c)

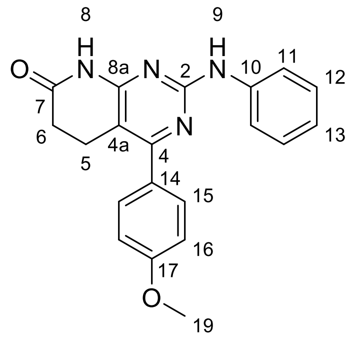

As above but carried out by using 4-iodo-2-(phenylamino)-5,6-dihydropyrido[2,3-*d*]pyrimidin-7(8*H*)-one (**17**) (73.23 mg, 0.20 mmol) and (4-methoxyphenyl)boronic acid (42.54 mg, 0.28 mmol). 54.1 mg (0.156 mmol, 78%) of spectroscopically pure 4-(4-methoxyphenyl)-2-(phenylamino)-5,6-dihydropyrido[2,3-*d*]pyrimidin-7(8*H*)-one (**22c**) were obtained as a grey solid. m.p. >250°C. ^{1}H-NMR (400 MHz, DMSO-*d*$_6$) δ (ppm): 10.66 (s, 1H, N8-H), 9.35 (s, 1H, N9-H), 7.89 – 7.83 (m, 2H, C11-H), 7.65 – 7.56 (m, 2H, C15-H), 7.31 – 7.17 (m, 2H, C12-H), 7.11 – 7.02 (m, 2H, C16-H), 6.95 – 6.85 (m, 1H, C13-H), 3.82 (s, 3H, C19-H), 2.88 (dd, *J* = 8.4, 6.5 Hz, 2H, C5-H), 2.53 – 2.48 (m, 2H, C6-H). ^{13}C-NMR (100.6 MHz, DMSO-*d*$_6$) δ (ppm): 172.0 (C7), 163.2 (C4), 160.3 (C17), 159.3 (C8a), 158.4 (C2), 141.4 (C10), 130.7 (C15), 130.5 (C14), 128.8 (C12), 121.2 (C13), 118.9 (C11), 113.9 (C16), 103.5 (C4a), 55.7 (C19), 31.2 (C6), 21.2 (C5). IR (ATR) ν (cm^{-1}): 3278, 3195, 3138, 2939, 1685, 1592, 1559, 1541, 1498, 1434, 1342, 1300, 1215, 1173, 1019, 835, 798, 755. HRMS (APCI-FIA-TOF) (m/z) calculated for $C_{20}H_{18}N_4O_2$: 346.1430, [M]$^+$, found: 347.1495, [M+H]$^+$.

1.6.4. Synthesis and characterization of 2-(phenylamino)-4-(3,4,5-trimethoxyphenyl)-5,6-dihydropyrido[2,3-*d*]pyrimidin-7(8*H*)-one (**22d**)

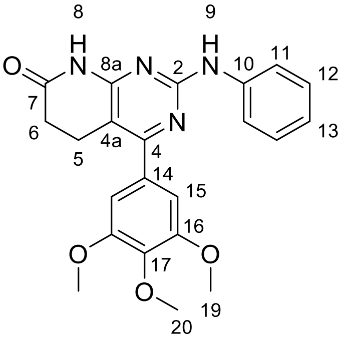

As above but carried out by using 4-iodo-2-(phenylamino)-5,6-dihydropyrido[2,3-*d*]pyrimidin-7(8*H*)-one (**17**) (73.23 mg, 0.20 mmol) and (3,4,5-trimethoxyphenyl)boronic acid (59.36 mg, 0.28 mmol). 51.2 mg (0.125 mmol, 63%) of spectroscopically pure 2-(phenylamino)-4-(3,4,5-trimethoxyphenyl)-5,6-dihydropyrido[2,3-*d*]pyrimidin-7(8*H*)-one (**22d**) were obtained as a beige solid. m.p. >250°C. ^{1}H-NMR (400 MHz, DMSO-*d*$_6$) δ (ppm): 10.70 (s, 1H, N8-H), 9.40 (s, 1H, N9-H), 7.91 – 7.80 (m, 2H, C11-H), 7.28 – 7.20 (m, 2H, C12-H), 6.95 – 6.88 (m, 3H, C15-H, C13-H), 3.83 (s, 6H C19-H), 3.73 (s, 3H, C20-H), 2.92 (dd, *J* = 8.4, 6.6 Hz, 2H, C5-H), 2.54 – 2.50 (m, 2H, C6-H). ^{13}C-NMR (100.6 MHz, DMSO-*d*$_6$) δ (ppm): 171.6 (C7), 162.9 (C4), 159.0 (C8a), 157.9 (C2), 152.5 (C16), 140.9 (C10), 138.1 (C17), 133.1 (C14), 128.3 (C12), 120.8 (C13), 118.6 (C11), 106.2 (C15), 103.4 (C4*a*), 60.1 (C20), 56.0 (C19), 30.7 (C6), 20.6 (C5). IR (ATR) ν (cm^{-1}): 3295, 3141, 2946, 1691, 1586, 1569, 1545, 1500, 1435, 1405, 1349, 1299, 1253, 1204, 1123, 997, 808, 754, 692. HRMS (APCI-FIA-TOF) (m/z) calculated for $C_{22}H_{22}N_4O_4$: 406.1641, [M]$^+$, found: 407.1701, [M+H]$^+$.

1.6.5. Synthesis and characterization of 4-(naphthalen-2-yl)-2-(phenylamino)-5,6-dihydropyrido[2,3-*d*]pyrimidin-7(8*H*)-one (**22e**)

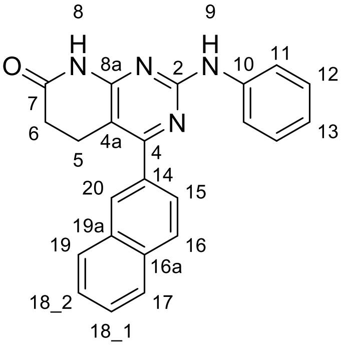

As above but carried out by using 4-iodo-2-(phenylamino)-5,6-dihydropyrido[2,3-*d*]pyrimidin-7(8*H*)-one (**17**) (73.23 mg, 0.20 mmol) and naphthalen-2-ylboronic acid (48.15 mg, 0.28 mmol). 56.2 mg (0.153 mmol, 76%) of spectroscopically pure 4-(naphthalen-2-yl)-2-(phenylamino)-5,6-dihydropyrido[2,3-*d*]pyrimidin-7(8*H*)-one (**22e**) were obtained as a greyish solid. m.p. >250°C. ^{1}H-NMR (400 MHz, DMSO-*d*$_6$) δ (ppm): 10.76 (s, 1H, N8-H), 9.46 (s, 1H, N9-H), 8.18 – 8.17 (m, 1H, C20-H), 8.07 – 7.98 (m, 3H, C16-H, C19-H, C17-H), 7.90 – 7.86 (m, 2H, C11-H), 7.81 – 7.77 (m, 1H, C15-H), 7.64 – 7.56 (m, 2H, C18-1-H, C18-2-H), 7.27 – 7.21 (m, 2H, C12-H), 6.93 – 6.88 (m, 1H, C13-H), 2.95 (dd, *J* = 8.4, 6.6 Hz, 2H, C5-H), 2.55 – 2.51 (m, 2H, C6-H). ^{13}C-NMR (100.6 MHz, DMSO-*d*$_6$) δ (ppm): 171.6 (C7), 163.1 (C4), 159.0 (C8a), 158.1 (C2), 140.9 (C10), 135.2 (C14*), 132.9 (C16a), 132.3 (C19*), 128.4 (C19), 128.4 (C12), 128.1 (C20), 127.7 (C16), 127.6 (C17), 126.9 (C18-1**), 126.5 (C18-2**), 126.2 (C15), 120.8 (C13), 118.5 (C11), 103.6 (C4a), 30.7 (C6), 20.6 (C5). IR (ATR) ν (cm^{-1}): 3279, 3135, 2911, 1688, 1592, 1565, 1543, 1497, 1440, 1346, 1307, 1217, 814, 752, 736. HRMS (APCI-FIA-TOF) (m/z) calculated for $C_{23}H_{18}N_4O$: 366.1481, [M]$^+$, found: 367.1550, [M+H]$^+$.

1.6.6. Synthesis and characterization of 4-(4-(dimethylamino)phenyl)-2-(phenylamino)-5,6-dihydropyrido[2,3-*d*]pyrimidin-7(8*H*)-one (**22f**)

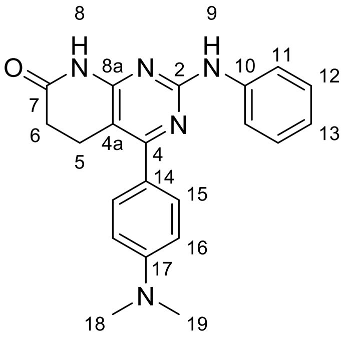

As above but carried out by using 4-iodo-2-(phenylamino)-5,6-dihydropyrido[2,3-*d*]pyrimidin-7(8*H*)-one (**17**) (73.23 mg, 0.20 mmol) and (4-(dimethylamino)phenyl)boronic acid (46.20 mg, 0.28 mmol). 50.1 mg (0.138 mmol, 70%) of spectroscopically pure 4-(4-(dimethylamino)phenyl)-2-(phenylamino)-5,6-dihydropyrido[2,3-*d*]pyrimidin-7(8*H*)-one (**22f**) were obtained as a beige solid. m.p. >250°C. ^{1}H-NMR (400 MHz, DMSO-*d*$_6$) δ (ppm): 10.57 (s, 1H, N8-H), 9.26 (s, 1H, N9-H), 7.90 – 7.82 (m, 2H, C11-H), 7.58 – 7.50 (m, 2H, C15-H), 7.27 – 7.18 (m, 2H, C12-H), 6.93 – 6.84 (m, 1H, C13-H), 6.84 – 6.76 (m, 2H, C16-H), 2.98 (s, 6H, C19-H), 2.92 (dd, *J* = 8.4, 6.6 Hz, 2H, C5-H), 2.52 – 2.46 (m, 2H, C6-H). ^{13}C-NMR (100.6 MHz, DMSO-*d*$_6$) δ (ppm): 171.6 (C7), 163.1 (C4), 158.7 (C8a), 157.9 (C2), 150.8 (C17), 141.1 (C10), 129.9 (C15), 128.3 (C12), 124.9 (C14), 120.6 (C13), 118.4 (C11), 111.1 (C16), 102.5 (C4a), 39.8 (C19), 30.9 (C6), 21.0 (C5). IR (ATR) ν (cm^{-1}): 3292, 3146, 2914, 1677, 1591, 1565, 1526, 1434, 1346, 1303, 1227, 1187, 804, 747. HRMS (APCI-FIA-TOF) (m/z) calculated for $C_{21}H_{21}N_5O$: 359.1746, [M]$^+$, found: 360.1811, [M+H]$^+$.

1.6.7. Synthesis and characterization of 4-(4-fluorophenyl)-2-(phenylamino)-5,6-dihydropyrido[2,3-*d*]pyrimidin-7(8*H*)-one (**22g**)

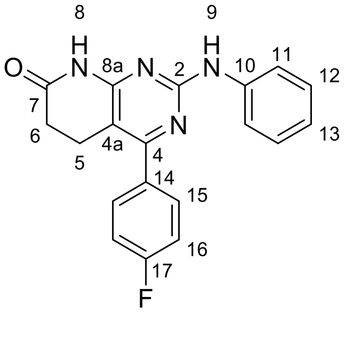

As above but carried out by using 4-iodo-2-(phenylamino)-5,6-dihydropyrido[2,3-*d*]pyrimidin-7(8*H*)-one (**17**) (73.23 mg, 0.20 mmol) and (4-fluorophenyl)boronic acid (39.17 mg, 0.28 mmol). 47.9 mg (0.143 mmol, 72%) of spectroscopically pure 4-(4-fluorophenyl)-2-(phenylamino)-5,6-dihydropyrido[2,3-*d*]pyrimidin-7(8*H*)-one (**22g**) were obtained as a greyish solid. m.p. >250°C. ^{1}H-NMR (400 MHz, DMSO-*d*$_6$) δ (ppm): 10.73 (s, 1H, N8-H), 9.42 (s, 1H, N9-H), 7.89 – 7.79 (m, 2H, C11-H), 7.74 – 7.64 (m, 2H, C15-H), 7.42 – 7.29 (m, 2H, C16-H), 7.28 – 7.18 (m, 2H, C12-H), 6.94 – 6.86 (m, 1H, C13-H), 2.85 (dd, *J* = 8.3, 6.6 Hz, 2H, C5-H), 2.53 – 2.49 (m, 2H, C6-H). ^{13}C-NMR (100.6 MHz, DMSO-*d*$_6$) δ (ppm): 171.6 (C7), 162.4 (d, *J* = 246.3 Hz, C17), 162.1 (C4), 159.0 (C8a), 158.0 (C2), 140.8 (C10), 134.1 (d, *J* = 3.2 Hz, C14), 130.9 (d, *J* = 8.5 Hz, C15), 128.3 (C12), 120.9 (C13), 118.5 (C11), 115.1 (d, *J* = 21.5 Hz, C16), 103.4 (C4a), 30.7 (C6), 20.5 (C5). IR (ATR) ν (cm^{-1}): 3284, 3203, 3135, 2960, 2841, 1679, 1591, 1544, 1497, 1436, 1345, 1301, 1214, 839, 800, 750, 691. HRMS (APCI-FIA-TOF) (m/z) calculated for $C_{19}H_{15}FN_4O$: 334.1230, [M]$^+$, found: 335.1299, [M+H]$^+$.

1.6.8. Synthesis and characterization of 4-(4-nitrophenyl)-2-(phenylamino)-5,6-dihydropyrido[2,3-*d*]pyrimidin-7(8*H*)-one (**22h**)

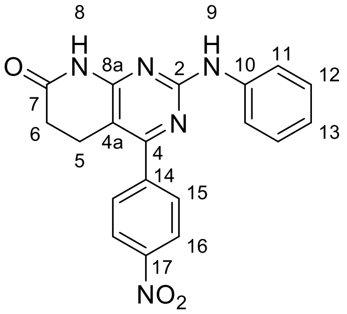

As above but carried out by using 4-iodo-2-(phenylamino)-5,6-dihydropyrido[2,3-*d*]pyrimidin-7(8*H*)-one (**17**) (73.23 mg, 0.20 mmol) and (4-nitrophenyl)boronic acid (47.0 mg, 0.28 mmol). 57.6 mg (0.159 mmol, 76%) of spectroscopically pure 4-(4-nitrophenyl)-2-(phenylamino)-5,6-dihydropyrido[2,3-*d*]pyrimidin-7(8*H*)-one (**22h**) were obtained as a yellowish solid. m.p. >250°C. ^{1}H-NMR (400 MHz, DMSO-d_6) δ (ppm): 10.84 (s, 1H, N8-H), 9.52 (s, 1H, N9-H), 8.41 – 8.31 (m, 2H, C16-H), 7.93 – 7.88 (m, 2H, C15-H), 7.85 – 7.81 (m, 2H, C11-H), 7.31 – 7.18 (m, 2H, C12-H), 6.97 – 6.88 (m, 1H, C13-H), 2.84 (dd, *J* = 8.4, 6.5 Hz, 2H, C5-H), 2.55 – 2.50 (m, 2H, C6-H). ^{13}C-NMR (100.6 MHz, DMSO-d_6) δ (ppm): 171.5 (C7), 161.0 (C4), 159.2 (C8a), 158.1 (C2), 147.6 (C17), 144.0 (C14), 140.6 (C10), 130.0 (C15), 128.4 (C12), 123.4 (C16), 121.1 (C13), 118.6 (C11), 104.0 (C4a), 30.5 (C6), 20.3 (C5). IR (KBr) ν (cm^{-1}): 3291, 3205, 3144, 2962, 1678, 1593, 1566, 1545, 1440, 1348, 1304, 1218, 869, 806, 753. HRMS (APCI-FIA-TOF) (m/z) calculated for $C_{19}H_{15}N_5O_3$: 361.1175, [M]$^+$, found: 362.1251, [M+H]$^+$.

1.6.9. Synthesis and characterization of 4-(7-oxo-2-(phenylamino)-5,6,7,8-tetrahydropyrido[2,3-*d*]pyrimidin-4-yl)benzonitrile (**22i**)

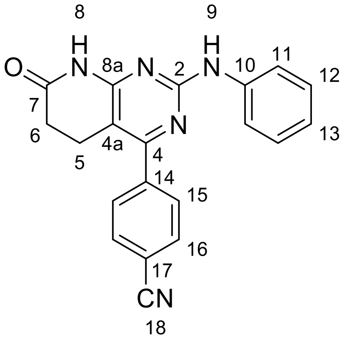

As above but carried out by using 4-iodo-2-(phenylamino)-5,6-dihydropyrido[2,3-*d*]pyrimidin-7(8*H*)-one (**17**) (73.23 mg, 0.20 mmol) and (4-cyanophenyl)boronic acid (41.14 mg, 0.28 mmol). 53.3 mg (0.156 mmol, 78%) of spectroscopically pure 4-(7-oxo-2-(phenylamino)-5,6,7,8-tetrahydropyrido[2,3-*d*]pyrimidin-4-yl)benzonitrile (**22i**) were obtained as a brown solid. m.p. >250°C. ^{1}H-NMR (400 MHz, DMSO-d_6) δ (ppm): 10.81 (s, 1H, N8-H), 9.50 (s, 1H, N9-H), 8.05 – 7.89 (m, 2H, C16-H), 7.98 – 7.75 (m, 4H, C11-H, C15-H), 7.31 – 7.18 (m, 2H, C12-H), 6.94 – 6.88 (m, 1H, C13-H), 2.82 (dd, *J* = 8.4, 6.6 Hz, 2H, C5-H), 2.54 – 2.50 (m, 2H, C6-H). ^{13}C-NMR (100.6 MHz, DMSO-d_6) δ (ppm): 172.0 (C7), 161.8 (C4), 159.6 (C8a), 158.5 (C2), 142.6 (C14), 141.1 (C10), 132.7 (C16), 130.0 (C15), 128.8 (C12), 121.5 (C13), 119.1 (C11), 119.0 (C17), 112.1 (C18), 104.3 (C4a), 31.0 (C6), 20.7 (C5). IR (ATR) ν (cm^{-1}): 3200, 3139, 2226, 1691, 1593, 1543, 1498, 1436, 1343, 1218, 1118, 845, 784, 749. HRMS (APCI-FIA-TOF) (m/z) calculated for $C_{20}H_{15}N_5O$: 341.1277, [M]$^+$, found: 342.1345, [M+H]$^+$.

1.6.10. Synthesis and characterization of 2-(phenylamino)-4-(thiophen-2-yl)-5,6-dihydropyrido[2,3-*d*]pyrimidin-7(8*H*)-one (22j)

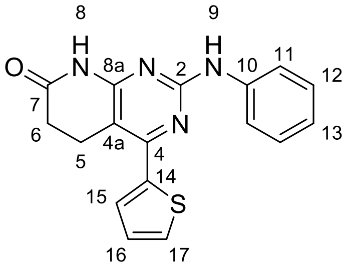

As above but carried out by using 4-iodo-2-(phenylamino)-5,6-dihydropyrido[2,3-*d*]pyrimidin-7(6*H*)-one (**17**) (73.23 mg, 0.20 mmol) and thiophen-2-ylboronic acid (35.82 mg, 0.28 mmol). 45.1 mg (0.140 mmol, 70%) of spectroscopically pure 2-(phenylamino)-4-(thiophen-2-yl)-5,6-dihydropyrido[2,3-*d*]pyrimidin-7(8*H*)-one (**22j**) were obtained as a yellowish solid. m.p. >250°C. ^{1}H-NMR (400 MHz, DMSO-d_6) δ (ppm): 10.68 (s, 1H, N8-H), 9.30 (s, 1H, N9-H), 7.90 – 7.86 (m, 2H, C11-H), 7.80 (dd, *J* = 5.1, 1.1 Hz, 1H, C15-H), 7.62 (dd, *J* = 3.8, 1.1 Hz, 1H, C16-H), 7.29 – 7.22 (m, 3H, C12-H, C17-H), 6.95 – 6.89 (m, 1H, C13-H), 3.10 (dd, *J* = 8.2, 7.1 Hz, 2H, C5-H), 2.60 (dd, *J* = 8.2, 7.1 Hz, 2H, C6-H). ^{13}C-NMR (100.6 MHz, DMSO-d_6) δ (ppm): 171.3 (C7), 159.4 (C4*), 157.4 (C2), 155.2 (C8a*), 142.3 (C14), 140.8 (C10), 129.7 (C15), 129.4 (C16), 128.3 (C12), 128.3 (C17), 121.0 (C13), 118.6 (C11), 101.9 (C4a), 30.3 (C6), 20.7 (C5). IR (ATR) ν (cm^{-1}): 3281, 3144, 2950, 1685, 1594, 1567, 1541, 1442, 1422, 1353, 1333, 1260, 1224, 778, 754, 690. HRMS (APCI-FIA-TOF) (m/z) calculated for $C_{17}H_{14}N_4OS$: 322.0888, $[M]^+$, found: 323.0957, $[M+H]^+$.

1.7. General procedure for C4 alkynyl substituted

Methodology A (Alkyne as excess reagent):

Intermediate 4-iodo-2-(phenylamino)-5,6-dihydropyrido[2,3-*d*]pyrimidin-7(8*H*)-one (**17**) (1 eq), bis(triphenylphosphine)palladium dichloride (1.5% molar), copper(I) iodide (3% molar) and the corresponding alkyne (1.5 eq) were introduced under Argon atmosphere into a Schlenk tube. Then, triethylamine (1.5 mL) was added and the Schlenk tube was sealed. The resulting reaction mixture was heated at 65°C overnight under vigorous stirring. The solvent was evaporated in vacuo and the resulting mixture was suspended in water (50 mL) and the spectroscopically pure product was collected by filtration after being washed with more water, ethanol, and diethyl ether.

Methodology B (Alkyne as limiting reagent):

Compound 4-ethynyl-2-(phenylamino)-5,6-dihydropyrido[2,3-*d*]pyrimidin-7(8*H*)-one (**24**) (1 eq), bis(triphenylphosphine)palladium dichloride (1.5% molar), copper(I) iodide (3% molar), the corresponding iodide-compound (1.3 eq) and triethylamine (2 mL) were introduced under Argon atmosphere into a Schlenk tube. The resulting mixture was stirred and heated at 65°C for 2 days. The solvent was evaporated in vacuo and water was added (50 mL) to the residue and the solid appeared was collected by filtration and washed with more water, ethanol, and diethyl ether to afford the spectroscopically pure product.

1.7.1. Synthesis and characterization of 4-(3-(dimethylamino)prop-1-yn-1-yl)-2-(phenylamino)-5,6-dihydropyrido[2,3-*d*]pyrimidin-7(8*H*)-one (23a)

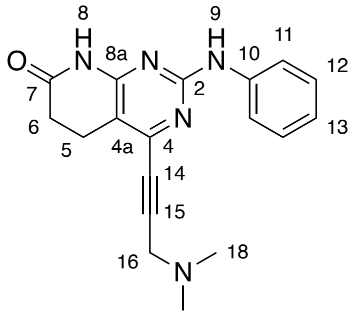

As above but carried out by using 4-iodo-2-(phenylamino)-5,6-dihydropyrido[2,3-*d*]pyrimidin-7(8*H*)-one (**17**) (75.0 mg, 0.205 mmol) and *N*,*N*-dimethylprop-2-yn-1-amine (29 µL, 0.266 mmol). 58.32 mg (0.181 mmol, 88%) of spectroscopically pure 4-(3-(dimethylamino)prop-1-yn-1-yl)-2-(phenylamino)-5,6-dihydropyrido[2,3-*d*]pyrimidin-7(8*H*)-one (**23a**) were obtained as a light grey solid. m.p. >250°C. ^{1}H-NMR (400 MHz, DMSO-d_6) δ (ppm): 10.75 (s, 1H, N8-H), 9.46 (s, 1H, N9-H), 7.80 – 7.76 (m, 2H, C11-H), 7.26 – 7.21 (m, 2H, C12-H), 6.94 – 6.88 (m, 1H, C13-H), 3.57 (s, 2H, C16-H), 2.88 (dd, *J* = 8.3, 6.9 Hz, 2H, C5-H), 2.59 (dd, *J* = 8.3, 6.9 Hz, 2H, C6-H), 2.26 (s, 6H, C18-H). ^{13}C-NMR (100.6 MHz, DMSO-d_6) δ (ppm): 171.4 (C7), 158.4 (C8a), 158.2 (C2), 146.9 (C4), 140.6 (C10), 128.4 (C12), 121.1 (C13), 118.7 (C15), 118.6 (C11), 107.5 (C4a), 81.7 (C14), 47.5 (C16), 43.7 (C18), 30.1 (C6), 20.2 (C5). IR (ATR) ν (cm^{-1}): 3285, 3145, 2945, 2198, 1684, 1592, 1546, 1438, 1339, 1302, 1215, 813, 749, 691. HRMS (APCI-FIA-TOF) (m/z) calculated for $C_{18}H_{19}N_5O$: 321.1590, [M]$^+$, found: 322.1654, [M+H]$^+$.

1.7.2. Synthesis and characterization of 2-(phenylamino)-4-((trimethylsilyl)ethynyl)-5,6-dihydropyrido[2,3-*d*]pyrimidin-7(8*H*)-one (23b)

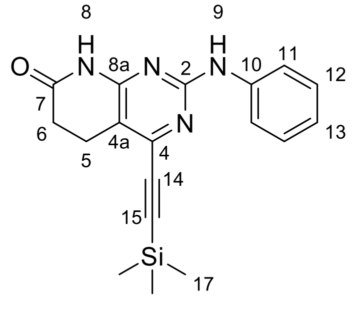

As above but carried out by using 4-iodo-2-(phenylamino)-5,6-dihydropyrido[2,3-*d*]pyrimidin-7(8*H*)-one (**17**) (300.0 mg, 0.82 mmol) and ethynyltrimethylsilane (170 µL, 1.23 mmol). 218.3 mg (0.649 mmol, 79%) of spectroscopically pure 2-(phenylamino)-4-((trimethylsilyl)ethynyl)-5,6-dihydropyrido[2,3-*d*]pyrimidin-7(8*H*)-one (**23b**) were obtained as a pale green solid. m.p. >250°C. ^{1}H-NMR (400 MHz, DMSO-d_6) δ (ppm): 10.80 (s, 1H, N8-H), 9.51 (s, 1H, N9-H), 7.83 – 7.71 (m, 2H, C11-H), 7.34 – 7.16 (m, 2H, C12-H), 7.01 – 6.87 (m, 1H, C13-H), 2.86 (dd, *J* = 8.3, 6.9 Hz, 2H, C5-H), 2.59 (dd, *J* = 8.3, 6.9 Hz, 2H, C6-H), 0.26 (s, 9H, C17-H). ^{13}C-NMR (100.6 MHz, DMSO-d_6) δ (ppm): 171.4 (C7), 158.5 (C8a), 158.2 (C2), 146.3 (C4), 140.5 (C10), 128.4 (C12), 121.1 (C13), 118.5 (C11), 107.9 (C4a), 100.7 (C15), 100.5 (C14), 30.1 (C6), 19.9 (C5), -0.5 (C17). IR (ATR) ν (cm^{-1}): 3283, 3144, 2957, 1685, 1592, 1567, 1547, 1500, 1437, 1335, 1210, 1063, 1020, 839, 744. HRMS (APCI-FIA-TOF) (m/z) calculated for $C_{18}H_{20}N_4Osi$: 336.1406, [M]$^+$, found: 337.1475, [M+H]$^+$.

1.7.3. Synthesis and characterization of 4-ethynyl-2-(phenylamino)-5,6-dihydropyrido[2,3-*d*]pyrimidin-7(8*H*)-one (**24**)

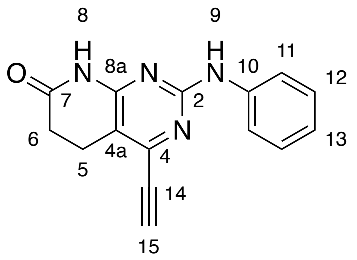

Intermediate 2-(phenylamino)-4-((trimethylsilyl)ethynyl)-5,6-dihydropyrido[2,3-d]pyrimidin-7(8H)-one (**23b**) (200 mg, 0.594 mmol) was 29issolved in a 1M TBAF/THF (10 mL). The resulting mixture was stirred for 3 h at room temperature. Upon completion water was added (200 mL) and the solid appeared was collected by filtration and washed with more water, ethanol (20 mL), and diethyl ether to afford the spectroscopically pure product **24**. 150.4 mg (0.569 mmol, 97%) were obtained as a greyish solid. m.p. >250°C. ^{1}H-NMR (400 MHz, DMSO-d_6) δ (ppm): 10.79 (s, 1H, N8-H), 9.49 (s, 1H, N9-H), 7.83 – 7.70 (m, 2H, C11-H), 7.33 – 7.16 (m, 2H, C12-H), 6.95 – 6.89 (m, 1H, C13-H), 4.72 (s, 1H, C15-H), 2.88 (dd, *J* = 8.3, 6.9 Hz, 2H, C5-H), 2.59 (dd, *J* = 8.3, 6.9 Hz, 2H, C6-H). ^{13}C-NMR (100.6 MHz, DMSO-d_6) δ (ppm): 171.5 (C7), 158.6 (C8a), 158.2 (C2), 146.1 (C4), 140.5 (C10), 128.4 (C12), 121.2 (C13), 118.6 (C11), 108.3 (C4a), 86.3 (C14), 79.7 (C15), 30.2 (C6), 19.9 (C5). IR (KBr) ν (cm^{-1}): 3277, 3202, 3142, 2957, 2112, 1688, 1593, 1545, 1499, 1437, 1336, 1302, 1255, 1206, 816, 752. HRMS (APCI-FIA-TOF) (m/z) calculated for $C_{15}H_{12}N_4O$: 264.1011, [M]$^+$, found: 265.1087, [M+H]$^+$.

1.7.4. Synthesis and characterization of 2-(phenylamino)-4-(phenylethynyl)-5,6-dihydropyrido[2,3-*d*]pyrimidin-7(8*H*)-one (**25a**)

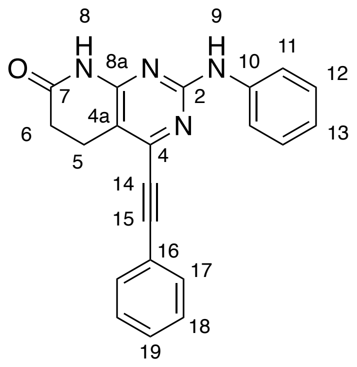

As above but carried out by using 4-ethynyl-2-(phenylamino)-5,6-dihydropyrido[2,3-*d*]pyrimidin-7(8*H*)-one (**24**) (100.0 mg, 0.378 mmol) and iodobenzene (55 μL, 0.491 mmol). 123.0 mg (0.361 mmol, 95%) of spectroscopically pure 2-(phenylamino)-4-(phenylethynyl)-5,6-dihydropyrido[2,3-*d*]pyrimidin-7(8*H*)-one (**25a**) were obtained as a brown solid. m.p. >250°C. ^{1}H-NMR (400 MHz, DMSO-d_6) δ (ppm): 10.82 (s, 1H, N8-H), 9.55 (s, 1H, N9-H), 7.85 – 7.77 (m, 2H, C11-H), 7.68 – 7.61 (m, 2H, C17-H), 7.55 – 7.45 (m, 3H, C19-H, C18-H), 7.30 – 7.21 (m, 2H, C12-H), 6.96 – 6.89 (m, 1H, C13-H), 2.99 (t, *J* = 7.6 Hz, 2H, C5-H), 2.63 (t, *J* = 7,6 Hz, 2H, C6-H). ^{13}C-NMR (100.6 MHz, DMSO-d_6) δ (ppm): 171.5 (C7), 158.5 (C2*), 158.3 (C8a*), 146.7 (C4), 140.6 (C10), 131.9 (C17), 130.1 (C19), 128.9 (C18), 128.4 (C12), 121.1 (C13), 120.7 (C16), 118.6 (C11), 107.8 (C4a), 94.3 (C15), 85.6 (C14), 30.2 (C6), 20.0 (C5). IR (ATR) ν (cm^{-1}): 3282, 3205, 3137, 2912, 2211, 1686, 1593, 1567, 1543, 1500, 1441, 1341, 1301, 1231, 1204, 819, 749. HRMS (APCI-FIA-TOF) (m/z) calculated for $C_{21}H_{16}N_4O$: 340.1324, [M]$^+$, found: 341.1400, [M+H]$^+$.

1.7.5. Synthesis and characterization of 4-(naphthalen-1-ylethynyl)-2-(phenylamino)-5,6-dihydropyrido[2,3-*d*]pyrimidin-7(8*H*)-one (25b)

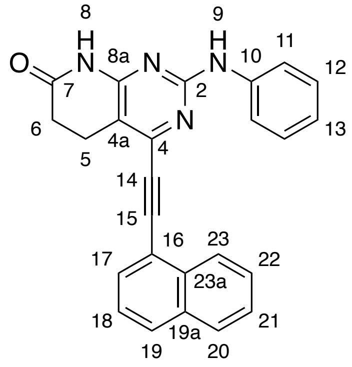

As above but carried out by using 4-ethynyl-2-(phenylamino)-5,6-dihydropyrido[2,3-*d*]pyrimidin-7(8*H*)-one (**24**) (100.0 mg, 0.378 mmol) and 1-iodonaphtalene (74 μL, 0.491 mmol). Once the product was filtered it was necessary to wash the product with chloroform in order to eliminate the leftover 1-iodonaphtalene. 76.2 mg (0.195 mmol, 52%) of spectroscopically pure 4-(naphthalen-1-ylethynyl)-2-(phenylamino)-5,6-dihydropyrido[2,3-*d*]pyrimidin-7(8*H*)-one (**25b**) were obtained as a brown solid. m.p. >250°C. ^{1}H-NMR (400 MHz, DMSO-d_6) δ (ppm): 10.86 (s, 1H, N8-H), 9.61 (s, 1H, N9-H), 8.37 – 8.28 (m, 1H, C23-H), 8.14 – 8.03 (m, 2H, C19-H, C20-H), 7.97 – 7.91 (m, 1H, C17-H), 7.88 – 7.82 (m, 2H, C11-H), 7.79 – 7.72 (m, 1H, C22-H), 7.69 – 7.59 (m, 2H, C21-H, C18-H), 7.31 – 7.23 (m, 2H, C12-H), 6.97 – 6.91 (m, 1H, C13-H), 3.10 (t, *J* = 7.6 Hz, 2H, C5-H), 2.68 (t, *J* = 7.6 Hz, 2H, C6-H). ^{13}C-NMR (100.6 MHz, DMSO-d_6) δ (ppm): 171.4 (C7), 158.6 (C8a*), 158.4 (C2*), 146.8 (C4), 140.6 (C10), 132.8 (C19a), 132.4 (C23a), 131.8 (C17), 130.5 (C19), 128.7 (C20), 128.4 (C12), 127.8 (C22), 127.0 (C21), 125.7 (C18), 124.9 (C23), 121.1 (C13), 118.6 (C11), 118.1 (C16), 107.8 (C4a), 92.1 (C15), 90.6 (C14), 30.2 (C6), 20.2 (C5). IR (ATR) ν (cm^{-1}): 3269, 3135, 3043, 2904, 2195, 1684, 1591, 1563, 1539, 1498, 1440, 1339, 1259, 1233, 1196, 795, 765, 750. HRMS (APCI-FIA-TOF) (m/z) calculated for $C_{25}H_{18}N_4O$: 390.1481, [M]$^+$, found: 391.1558, [M+H]$^+$.

1.7.6. Synthesis and characterization of 4-([1,1'-biphenyl]-4-ylethynyl)-2-(phenylamino)-5,6-dihydropyrido[2,3-*d*]pyrimidin-7(8*H*)-one (25c)

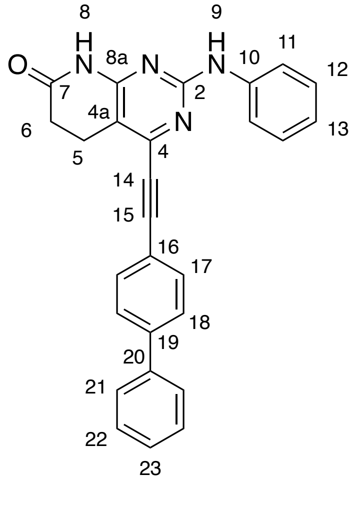

As above but carried out by using 4-ethynyl-2-(phenylamino)-5,6-dihydropyrido[2,3-*d*]pyrimidin-7(8*H*)-one (**24**) (100.0 mg, 0.378 mmol) and 4-iodo-1,1'-biphenyl (137.8 mg, 0.491 mmol). 132.7 mg (0.318 mmol, 84%) of spectroscopically pure 4-([1,1'-biphenyl]-4-ylethynyl)-2-(phenylamino)-5,6-dihydropyrido[2,3-*d*]pyrimidin-7(8*H*)-one (**25c**) were obtained as a brown solid. m.p. >250°C. ^{1}H-NMR (400 MHz, DMSO-d_6) δ (ppm): 10.83 (s, 1H, N8-H), 9.56 (s, 1H, N9-H), 7.83 – 7.78 (m, 4H, C11-H, C18-H), 7.77 – 7.70 (m, 4H, C17-H, C21-H), 7.55 – 7.47 (m, 2H, C22-H), 7.45 – 7.39 (m, 1H, C23-H), 7.29 – 7.23 (m, 2H, C12-H), 6.93 (t, *J* = 7.3 Hz, 1H, C13-H), 3.01 (dd, *J* = 8.3, 6.9 Hz, 2H, C5-H), 2.64 (dd, *J* = 8.3, 6.9 Hz, 2H, C6-H). ^{13}C-NMR (100.6 MHz, DMSO-d_6) δ (ppm): 171.5 (C7), 158.5 (C8a*), 158.3 (C2*), 146.7 (C4), 141.5 (C19), 140.6 (C10), 138.9 (C20), 132.6 (C17), 129.1 (C22), 128.4 (C12), 128.2 (C23), 127.1 (C18), 126.8 (C21), 121.1 (C13), 119.6 (C16), 118.6 (C11), 107.9 (C4a), 94.2 (C15), 86.3 (C14), 30.2 (C6), 20.1 (C5). IR (ATR) ν (cm^{-1}): 3285, 3205,

3145, 2912, 2214, 1684, 1592, 1567, 1543, 1500, 1438, 1341, 1232, 1209, 838, 747. HRMS (APCI-FIA-TOF) (m/z) calculated for $C_{27}H_{20}N_4O$: 416.1637, $[M]^+$, found: 417.1707, $[M+H]^+$.

1.7.7. Synthesis and characterization of 2-(phenylamino)-4-(pyridin-3-ylethynyl)-5,6-dihydropyrido[2,3-*d*]pyrimidin-7(8*H*)-one (**25d**)

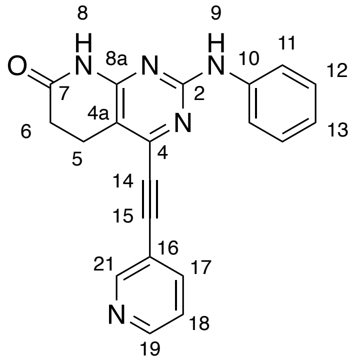

As above but carried out by using 4-iodo-2-(phenylamino)-5,6-dihydropyrido[2,3-*d*]pyrimidin-7(8*H*)- one (**17**) (100.0 mg, 0.273 mmol) and 3-ethynylpyridine (36.7 mg, 0.355 mmol). 81.0 mg (0.237 mmol, 87%) of spectroscopically pure 2-(phenylamino)-4-(pyridin-3-ylethynyl)-5,6-dihydropyrido[2,3-*d*]pyrimidin-7(8*H*)-one (**25d**) were obtained as a orangeish solid. m.p. >250°C. ^{1}H-NMR (400 MHz, DMSO-d_6) δ (ppm): 10.86 (s, 1H, N8-H), 9.58 (s, 1H, N9-H), 8.88 (sa, 1H, C19-H), 8.71 (sa, 1H, C21-H), 8.11 – 8.06 (m, 1H, C17-H), 7.82 – 7.78 (m, 2H, C11-H), 7.54 (sa, 1H, C18-H), 7.29 – 7.22 (m, 2H, C12-H), 6.96 – 6.90 (m, 1H, C13-H), 3.01 (dd, *J* = 8.3, 7.0 Hz, 2H, C5-H), 2.63 (dd, *J* = 8.3, 7.0 Hz, 2H, C6-H). ^{13}C-NMR (100.6 MHz, DMSO-d_6) δ (ppm): 171.5 (C7), 158.6 (C8a*), 158.3 (C2*), 146.2 (C4), 140.5 (C10), 139.1 (C17), 128.4 (C12), 121.2 (C13), 118.7 (C11), 108.2 (C4a), 90.9 (C15), 88.3 (C14), 30.2 (C6), 20.0 (C5). IR (ATR) ν (cm^{-1}): 3282, 3207, 3140, 2982, 2219, 1681, 1592, 1542, 1436, 1341, 1299, 1232, 1208, 799, 746, 691. HRMS (APCI-FIA-TOF) (m/z) calculated for $C_{20}H_{15}N_5O$: 341.1277, $[M]^+$, found: 342.1346, $[M+H]^+$.

1.7.8. Synthesis and characterization of 4-((2,4-dimethoxyphenyl)ethynyl)-2-(phenylamino)-5,6-dihydropyrido[2,3-*d*]pyrimidin-7(8*H*)-one (**25e**)

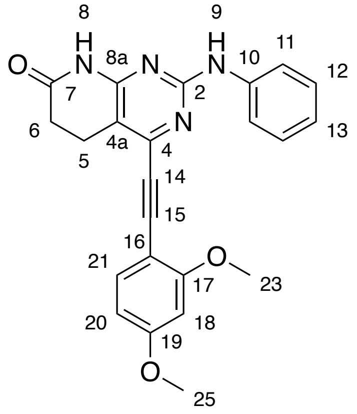

As above but carried out by using 4-ethynyl-2-(phenylamino)-5,6-dihydropyrido[2,3-*d*]pyrimidin-7(8*H*)-one (**24**) (70.0 mg, 0.264 mmol) and 1-iodo-2,4-dimethoxybenzene (104.9 mg, 0.397 mmol). 93.8 mg (0.234 mmol, 89%) of spectroscopically pure 4-((2,4-dimethoxyphenyl)ethynyl)-2-(phenylamino)-5,6-dihydropyrido[2,3-*d*]pyrimidin-7(8*H*)-one (**25e**) were obtained as a brownish solid. m.p. >250°C. ^{1}H-NMR (400 MHz, DMSO-d_6) δ (ppm): 10.75 (s, 1H, N8-H), 9.50 (s, 1H, N9-H), 7.86 – 7.74 (m, 2H, C11-H), 7.49 – 7.45 (m, 1H, C21-H), 7.27 – 7.22 (m, 2H, C12-H), 6.94 – 6.89 (m, 1H, C13-H), 6.69 – 6.67 (m, 1H, C20-H), 6.63 – 6.59 (m, 1H, C16-H), 3.88 (s, 3H, C23-H*), 3.83 (s, 3H, C25-H*), 2.97 (dd, *J* = 8.4, 6.9 Hz, 2H, C5-H), 2.62 (dd, *J* = 8.4, 6.9 Hz, 2H, C6-H). ^{13}C-NMR (100.6 MHz, DMSO-d_6) δ (ppm): 171.5 (C7), 162.3 (C17*), 161.9 (C19*), 158.3 (C8a**), 158.3 (C2**), 147.6 (C4), 140.7 (C10), 134.6 (C21), 128.4 (C12), 121.0 (C13), 118.5 (C11), 107.2 (C4a), 106.1 (C18), 102.0 (C16), 98.5 (C20), 92.5 (C15), 88.6 (C14), 55.9 (C23***), 55.6

(C25***), 30.3 (C6), 19.9 (C5). IR (ATR) ν (cm^{-1}): 3277, 3140, 2974, 2207, 1683, 1593, 1566, 1543, 1499, 1438, 1347, 1304, 1207, 1024, 819, 749. HRMS (APCI-FIA-TOF) (m/z) calculated for $C_{23}H_{20}N_4O_3$: 400.1535, $[M]^+$, found: 401.1601, $[M+H]^+$.

1.7.9. Synthesis and characterization of 4-((3-aminophenyl)ethynyl)-2-(phenylamino)-5,6-dihydropyrido[2,3-*d*]pyrimidin-7(8*H*)-one (25f)

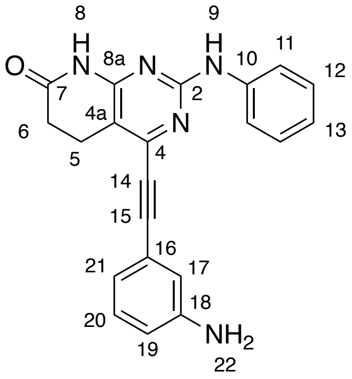

As above but carried out by using 4-ethynyl-2-(phenylamino)-5,6-dihydropyrido[2,3-*d*]pyrimidin-7(8*H*)-one (**24**) (150.0 mg, 0.567 mmol) and 3-iodoaniline (161.6 mg, 0.737 mmol). 194.5 mg (0.547 mmol, 96%) of spectroscopically pure 4-((3-aminophenyl)ethynyl)-2-(phenylamino)-5,6-dihydropyrido[2,3-*d*]pyrimidin-7(8*H*)-one (**25f**) were obtained as a brownish solid. m.p. >250°C. ^{1}H-NMR (400 MHz, DMSO-*d*$_6$) δ (ppm): 10.79 (s, 1H, N8-H), 9.53 (s, 1H, N9-H), 7.83 – 7.76 (m, 2H, C11-H), 7.29 – 7.21 (m, 2H, C12-H), 7.14 – 7.06 (m, 1H, C20-H), 6.96 – 6.88 (m, 1H, C13-H), 6.82 – 6.78 (m, 1H, C17-H), 6.77 – 6.71 (m, 1H, C21-H), 6.71 – 6.64 (m, 1H, C19-H), 5.34 (s, 2H, N22-H), 2.95 (dd, *J* = 8.3, 6.9 Hz, 2H, C5-H), 2.62 (t, *J* = 7.6 Hz, 2H, C6-H). ^{13}C-NMR (100.5 MHz, DMSO-*d*$_6$) δ (ppm): 171.9 (C7), 158.9 (C8a*), 158.8 (C2*), 149.4 (C18), 147.5 (C4), 141.0 (C10), 129.9 (C20), 128.9 (C12), 121.5 (C13), 121.3 (C16), 119.7 (C21), 119.0 (C11), 116.9 (C17), 116.2 (C19), 108.0 (C4a), 96.0 (C15), 84.8 (C14), 30.6 (C6), 20.5 (C5). IR (ATR) ν (cm^{-1}): 3285, 3148, 2971, 2216, 1685, 1592, 1567, 1543, 1500, 1441, 1344, 1301, 1214, 747. HRMS (APCI-FIA-TOF) (m/z) calculated for $C_{21}H_{17}N_5O$: 355.1433, $[M]^+$, found: 356.1495, $[M+H]^+$.

1.7.10. Synthesis and characterization of 4-((4-nitrophenyl)ethynyl)-2-(phenylamino)-5,6-dihydropyrido[2,3-*d*]pyrimidin-7(8*H*)-one (25g)

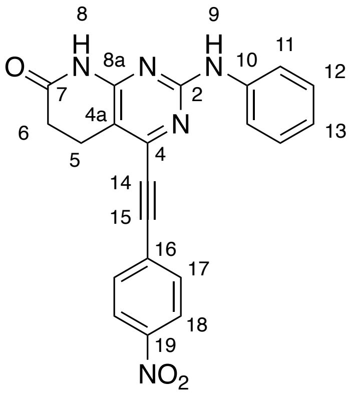

As above but carried out by using 4-ethynyl-2-(phenylamino)-5,6-dihydropyrido[2,3-*d*]pyrimidin-7(8*H*)-one (**24**) (60 mg, 0.227 mmol) and 1-iodo-4-nitrobenzene (84.4 mg, 0.340 mmol). 82.8 mg (0.214 mmol, 95%) of spectroscopically pure 4-((4-nitrophenyl)ethynyl)-2-(phenylamino)-5,6-dihydropyrido[2,3-*d*]pyrimidin-7(8*H*)-one (**25g**) were obtained as a orangish solid. m.p. >250°C. ^{1}H-NMR (400 MHz, DMSO-*d*$_6$) δ (ppm): 10.88 (s, 1H, N8-H), 9.59 (s, 1H, N9-H), 8.34 – 8.27 (m, 2H, C18-H), 7.96 – 7.89 (m, 2H, C17-H), 7.83 – 7.76 (m, 2H, C11-H), 7.30 – 7.22 (m, 2H, C12-H), 6.97 – 6.89 (m, 1H, C13-H), 3.01 (dd, *J* = 8.3, 7.0 Hz, 2H, C5-H), 2.63 (dd, *J* = 8.3, 7.0 Hz, 2H, C6-H). ^{13}C-NMR (100.5 MHz, DMSO-*d*$_6$) δ (ppm): 171.4 (C7), 158.7 (C8a), 158.1 (C2), 147.7 (C19), 145.8 (C4), 140.4 (C10), 133.3 (C17), 128.4 (C12), 127.3 (C16), 124.0 (C18), 121.2 (C13), 118.7 (C11), 108.6 (C4a), 91.7 (C15), 89.5 (C14), 30.1 (C6), 20.0 (C5). IR

(ATR) ν (cm^{-1}): 3274, 3143, 2966, 2216, 1696, 1591, 1546, 1523, 1443, 1341, 1304, 1231, 853, 747. HRMS (APCI-FIA-TOF) (m/z) calculated for $C_{21}H_{15}N_5O_3$: 385.1175, $[M]^+$, found: 386.1248, $[M+H]^+$.

1.7.11. Synthesis and characterization of 4-((4-fluorophenyl)ethynyl)-2-(phenylamino)-5,6-dihydropyrido[2,3-*d*]pyrimidin-7(8*H*)-one (25h)

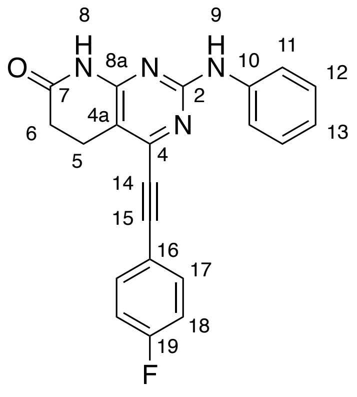

As above but carried out by using 4-ethynyl-2-(phenylamino)-5,6-dihydropyrido[2,3-*d*]pyrimidin-7(8*H*)-one (**24**) (100 mg, 0.378 mmol) and 1-fluoro-4-iodobenzene (60 μL, 0.491 mmol). 111.1 mg (0.310 mmol, 82%) of spectroscopically pure 4-((4-fluorophenyl)ethynyl)-2-(phenylamino)-5,6-dihydropyrido[2,3-*d*]pyrimidin-7(8*H*)-one (**25h**) were obtained as a brownish solid. m.p. >250°C. ^{1}H-NMR (400 MHz, DMSO-*d*$_6$) δ (ppm): 10.82 (s, 1H, N8-H), 9.54 (s, 1H, N9-H), 7.83 – 7.78 (m, 2H, C11-H), 7.75 – 7.70 (m, 2H, C17-H), 7.37 – 7.31 (m, 2H, C18-H), 7.28 – 7.22 (m, 2H, C12-H), 6.96 – 6.88 (m, 1H, C13-H), 2.98 (dd, *J* = 8.3, 6.9 Hz, 2H, C5-H), 2.65 – 2.62 (dd, *J* = 8.3, 6.9 Hz, 2H, C6-H). ^{13}C-NMR (100.5 MHz, DMSO-*d*$_6$) δ (ppm): 171.5 (C7), 162.8 (d, *J* = 249.5 Hz, C19), 158.7 (C8a), 158.3 (C2), 146.6 (C4), 140.5 (C10), 134.5 (d, *J* = 8.9 Hz, C17), 128.4 (C12), 121.1 (C13), 118.6 (C11), 117.2 (d, *J* = 3.2 Hz, C16), 116.3 (d, *J* = 22.4 Hz, C18), 107.9 (C4a), 93.2 (C15), 85.4 (C14), 30.2 (C6), 20.0 (C5). IR (ATR) ν (cm^{-1}): 3266, 3143, 2960, 2208, 1686, 1591, 1541, 1497, 1439, 1341, 1222, 1154, 839, 794, 746. HRMS (APCI-FIA-TOF) (m/z) calculated for $C_{21}H_{15}FN_4O$: 358.1230, $[M]^+$, found: 359.1308, $[M+H]^+$.

NMR Spectra

Figure S1. 4-Amino-2-(phenylamino)-5,6-dihydropyrido[2,3-*d*]pyrimidin-7(8*H*)-one (13)

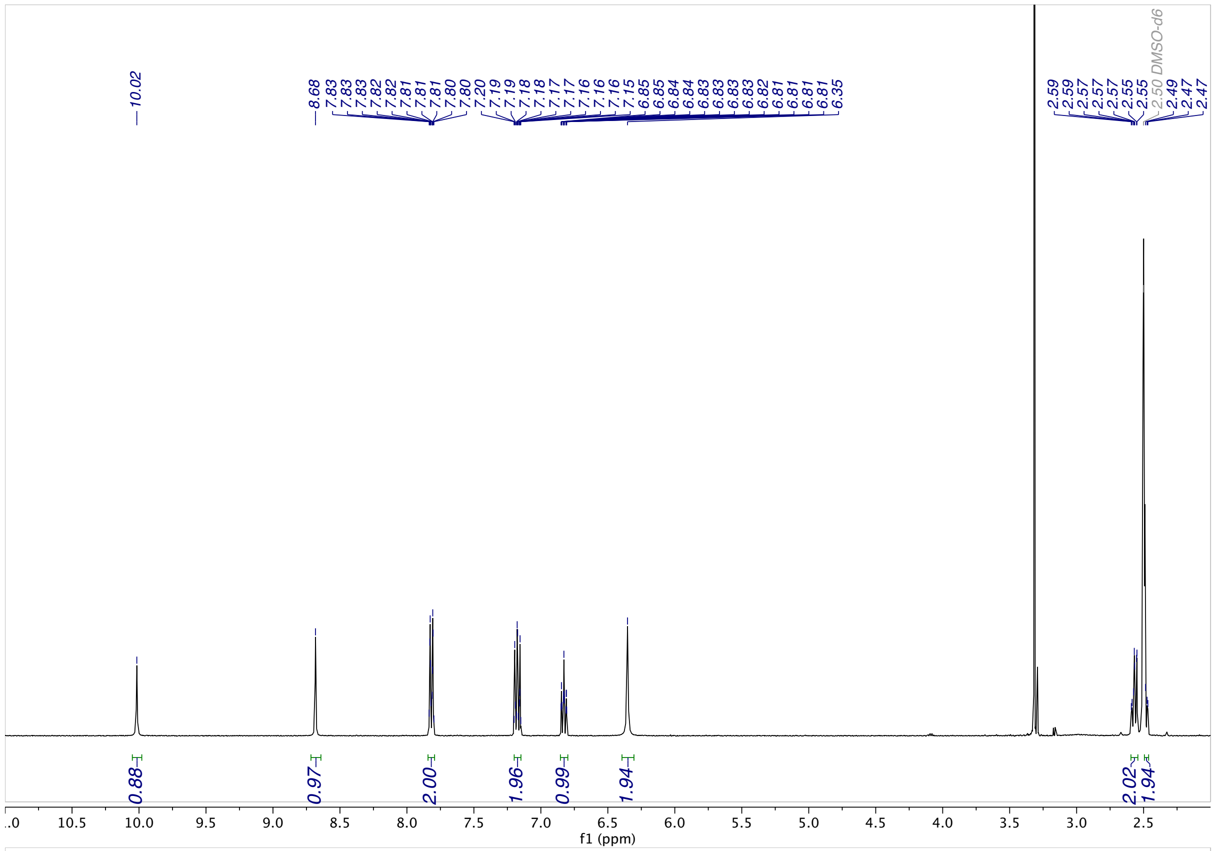

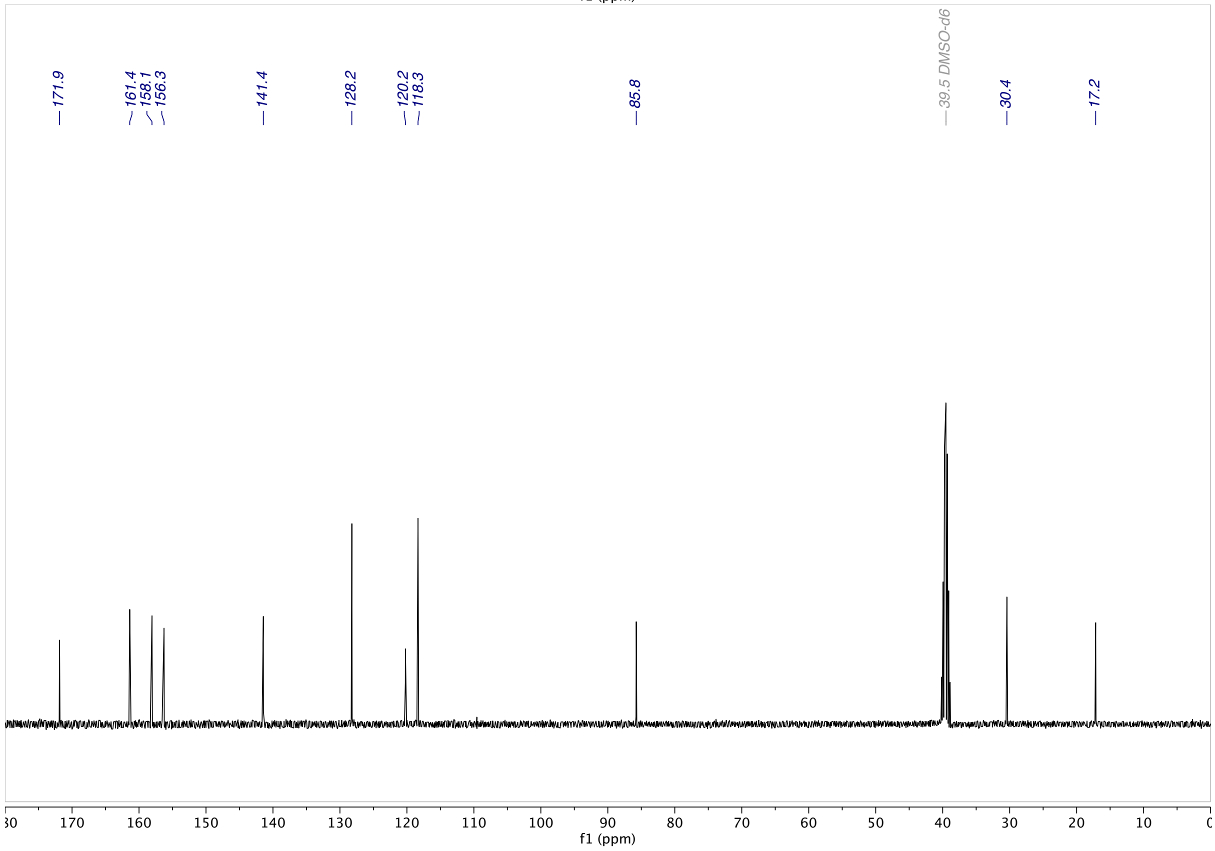

Figure S2. 2-(Phenylamino)-5,6-dihydropyrido[2,3-*d*]pyrimidine-4,7(3*H*,8*H*)-dione (14)

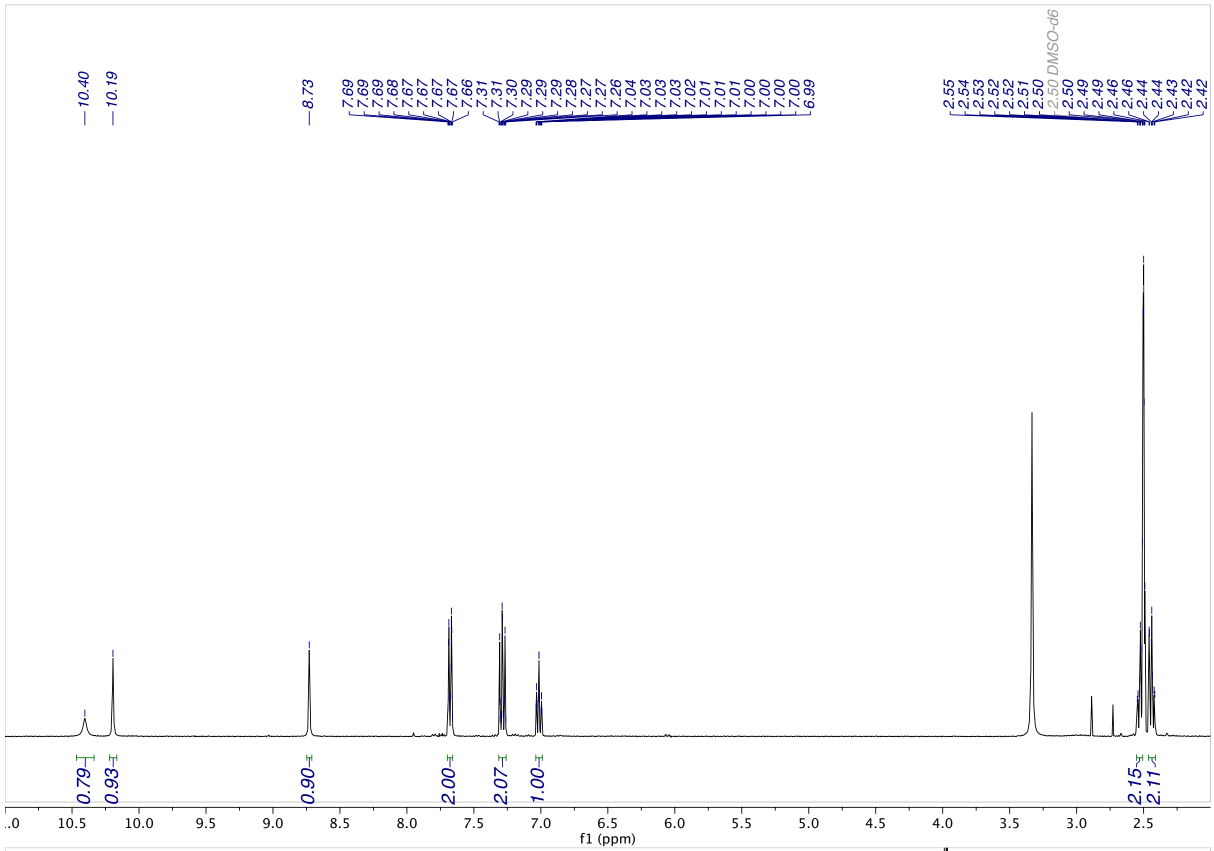

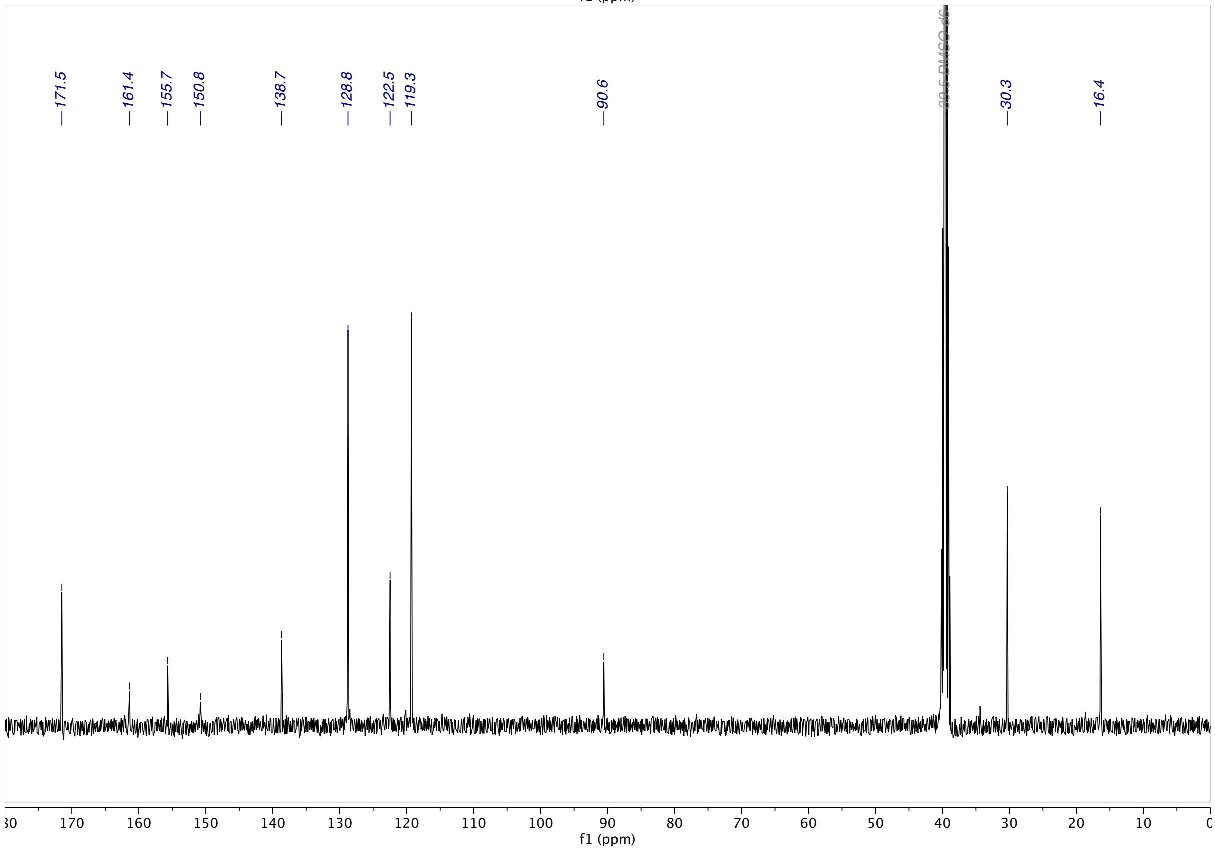

Figure S3. 4-((1*H*-benzo[*d*][1,2,3]triazol-1-yl)oxy)-2-(phenylamino)-5,6-dihydropyrido[2,3-*d*]pyrimidin-7(8*H*)-one (15)

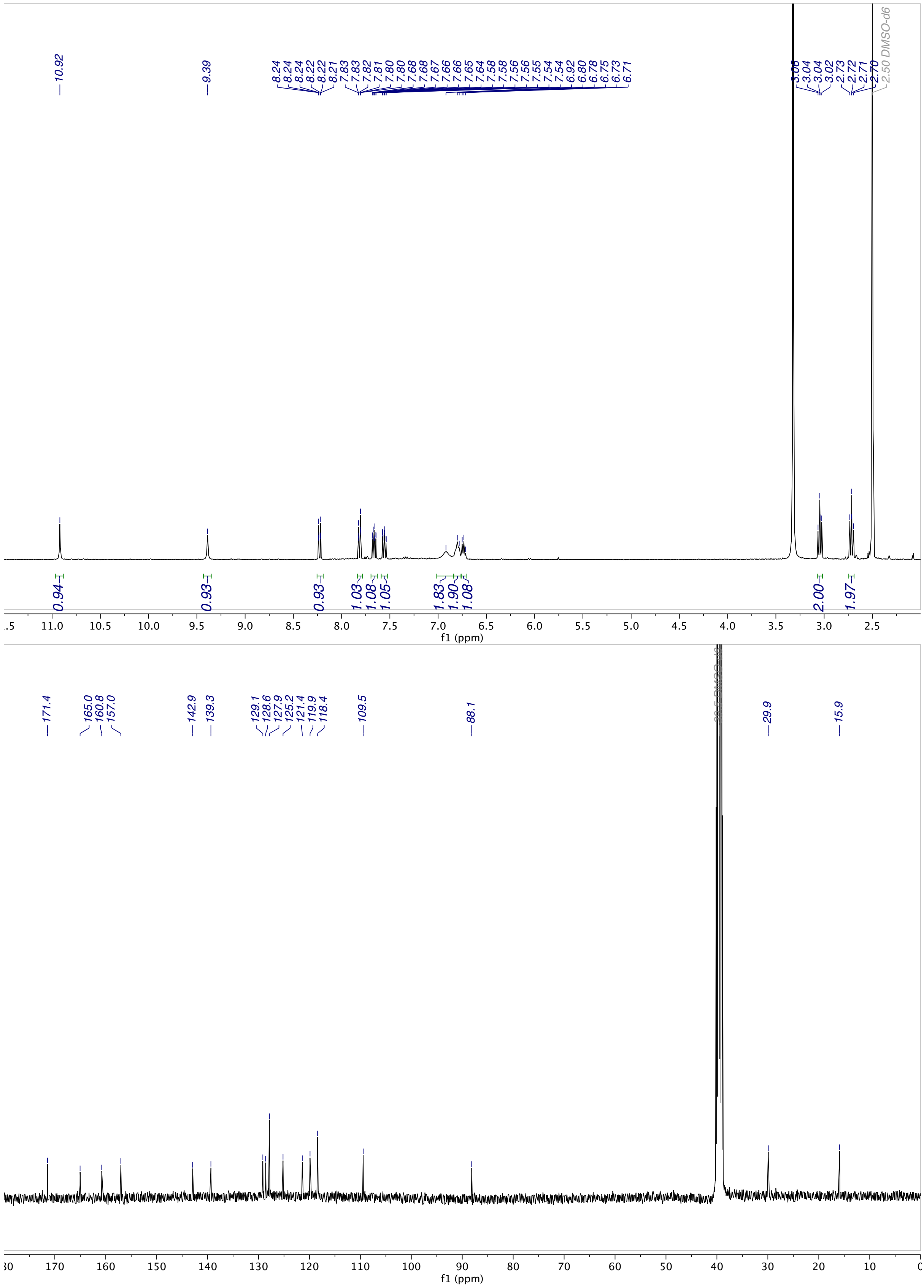

Figure S4. 7-Oxo-2-(phenylamino)-5,6,7,8-tetrahydropyrido[2,3-*d*]pyrimidin-4-yl trifluoromethanesulfonate (16)

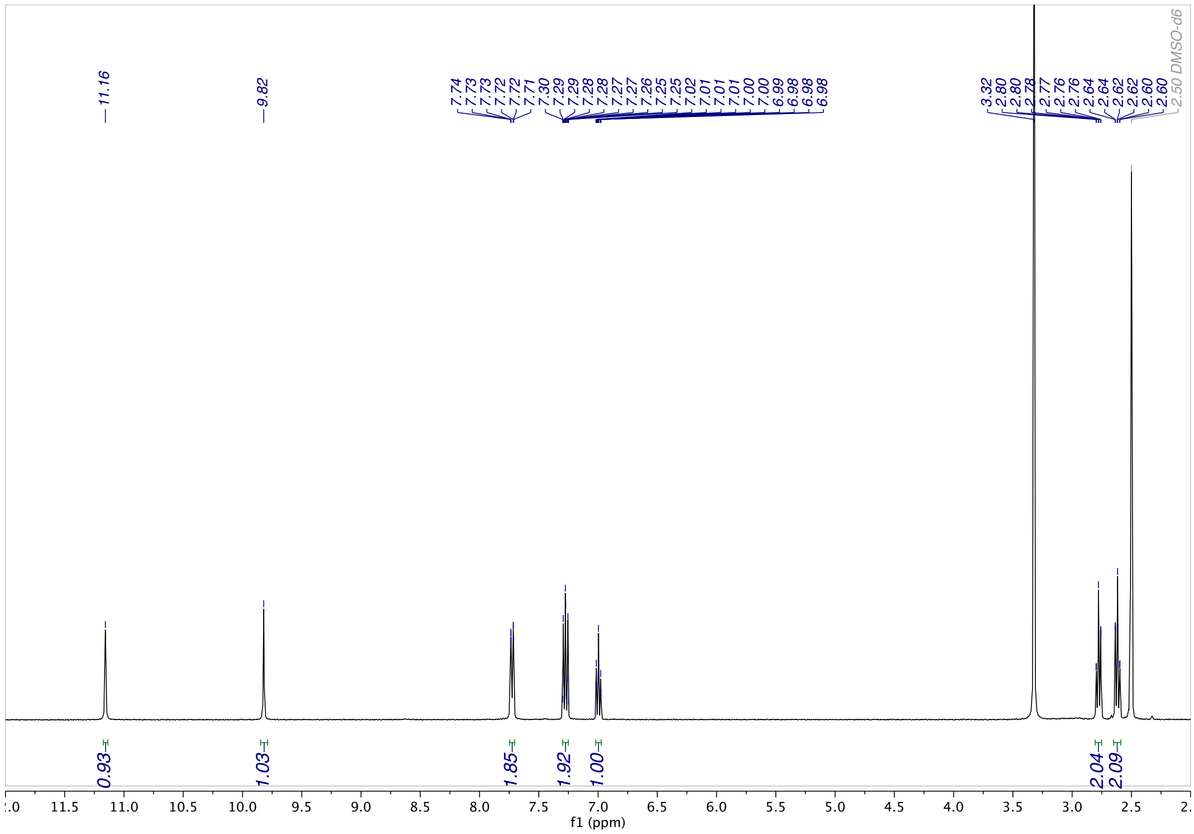

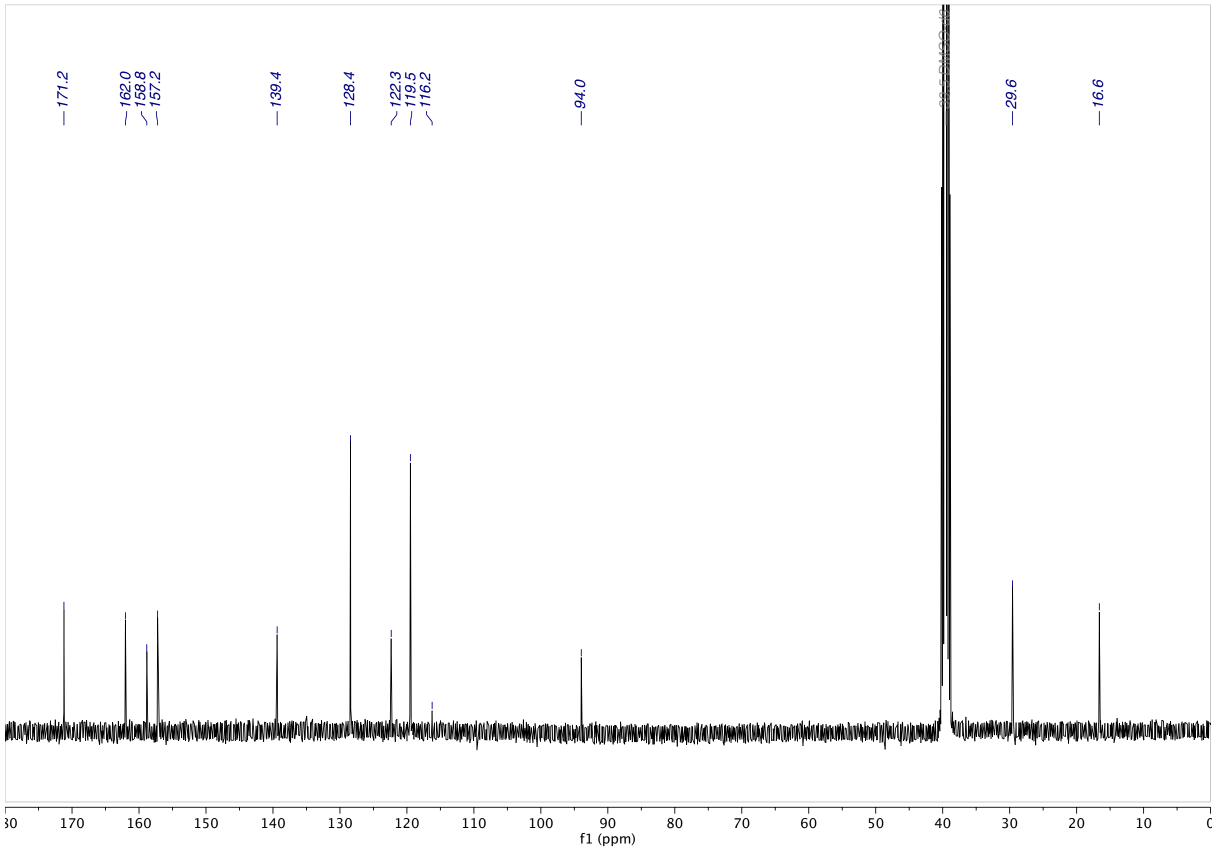

Figure S5. 4-Iodo-2-(phenylamino)-5,6-dihydropyrido[2,3-*d*]pyrimidin-7(8*H*)-one (17)

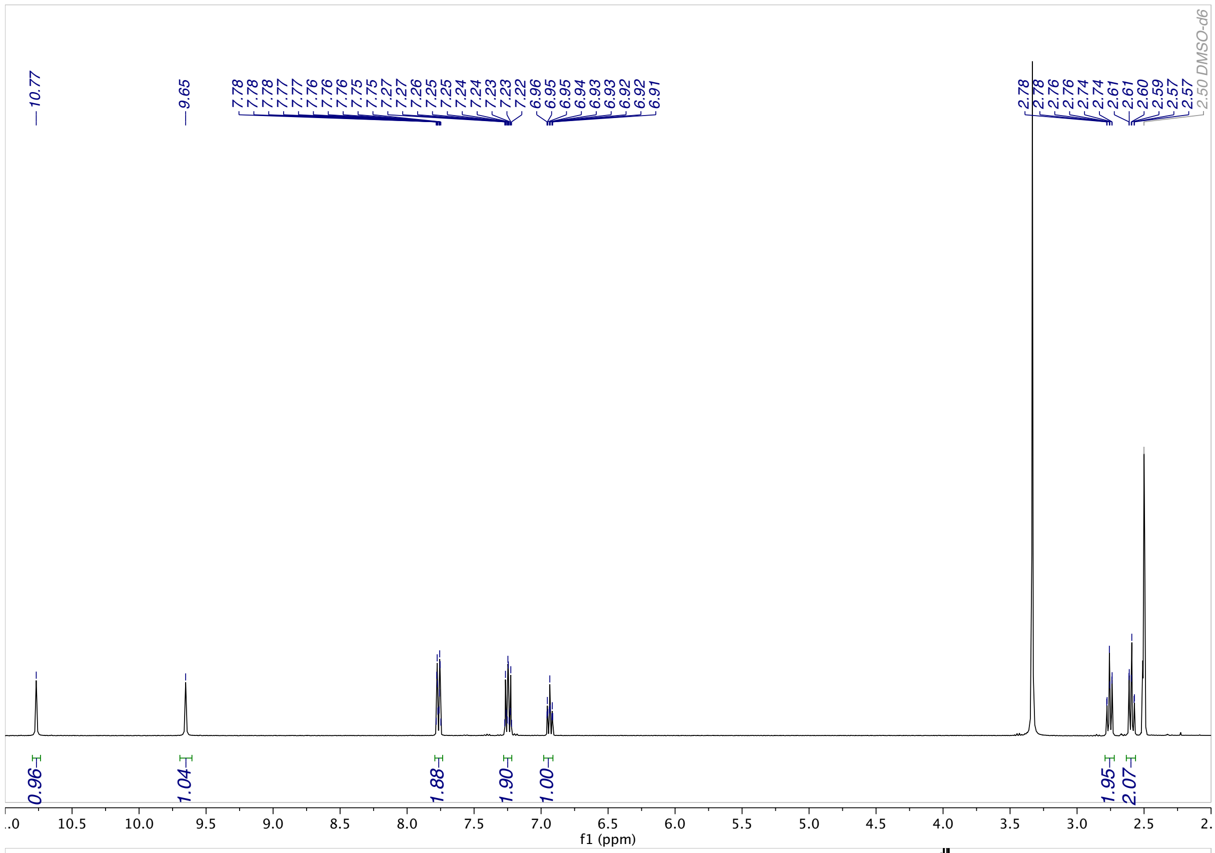

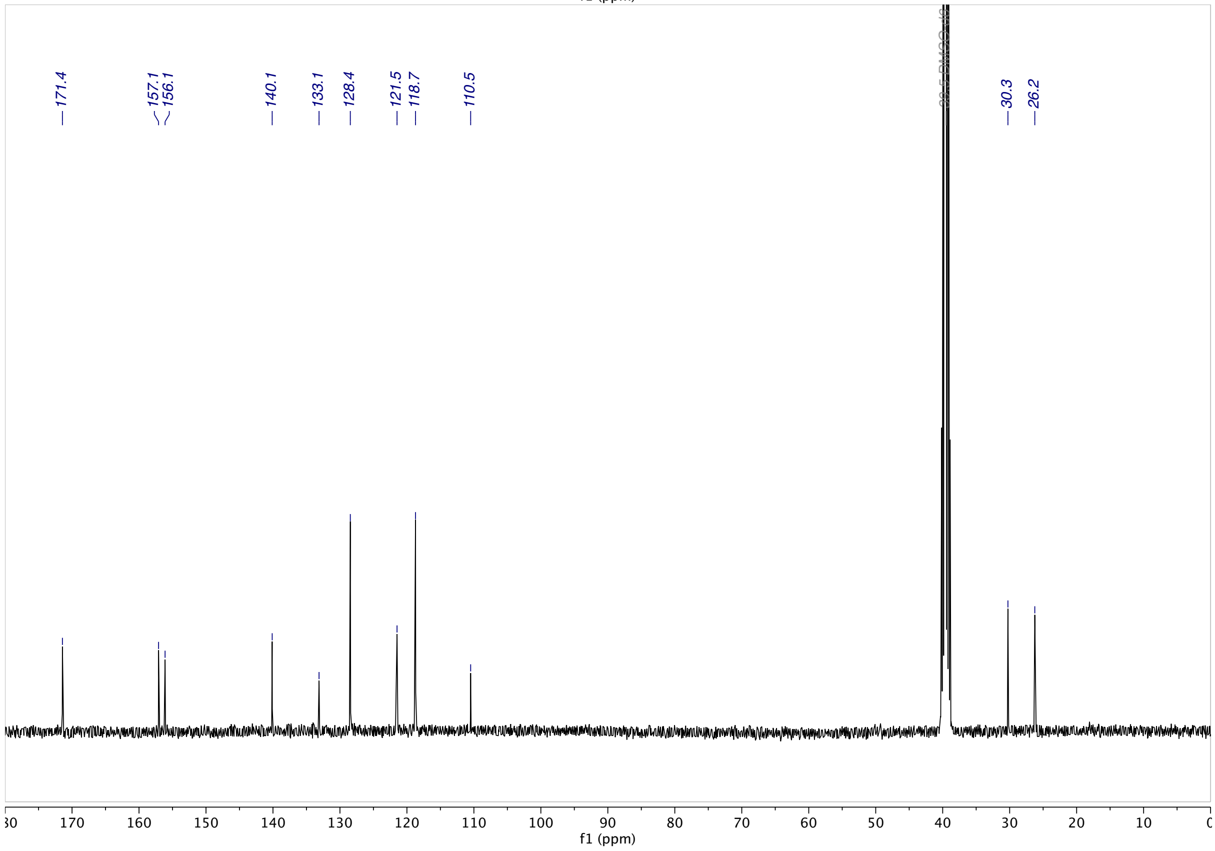

Figure S6. 4-((3-Morpholinopropyl)amino)-2-(phenylamino)-5,6-dihydropyrido[2,3-*d*]pyrimidin-7(8*H*)-one (18a)

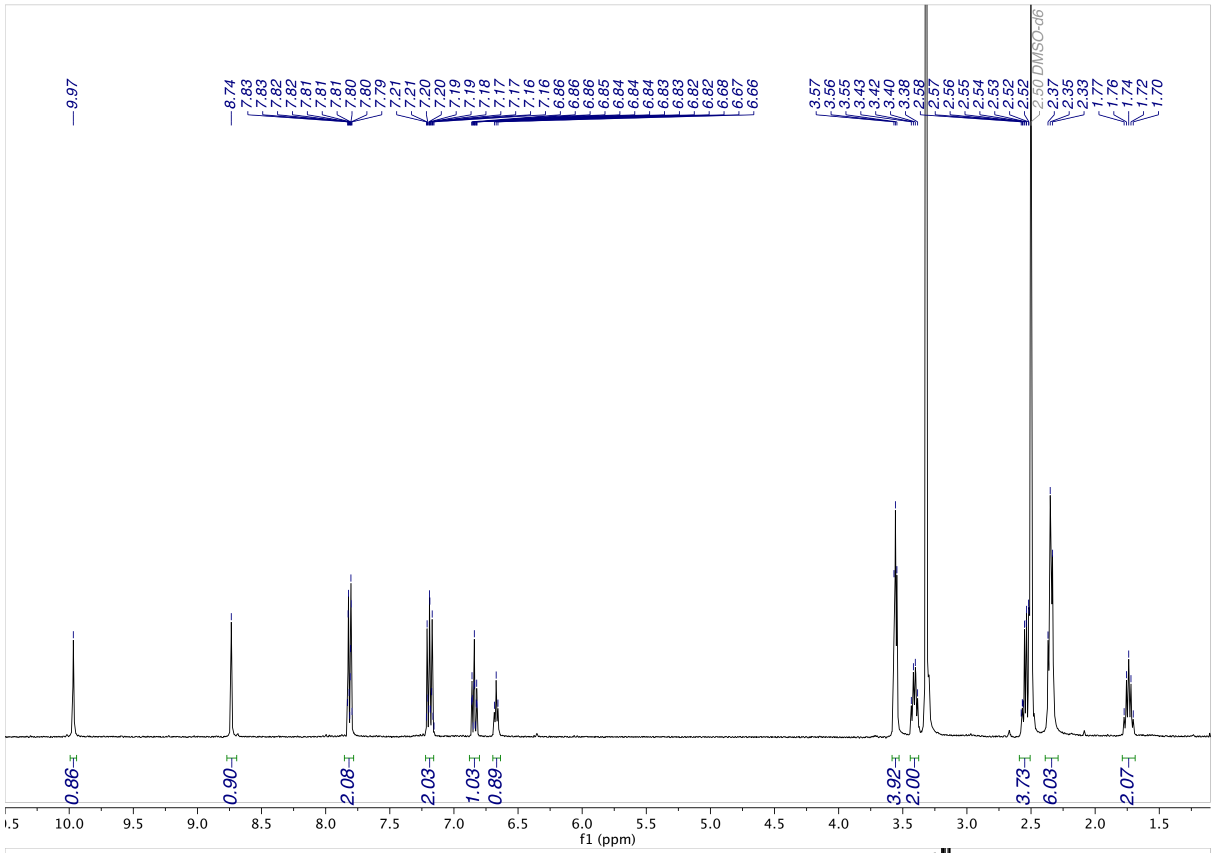

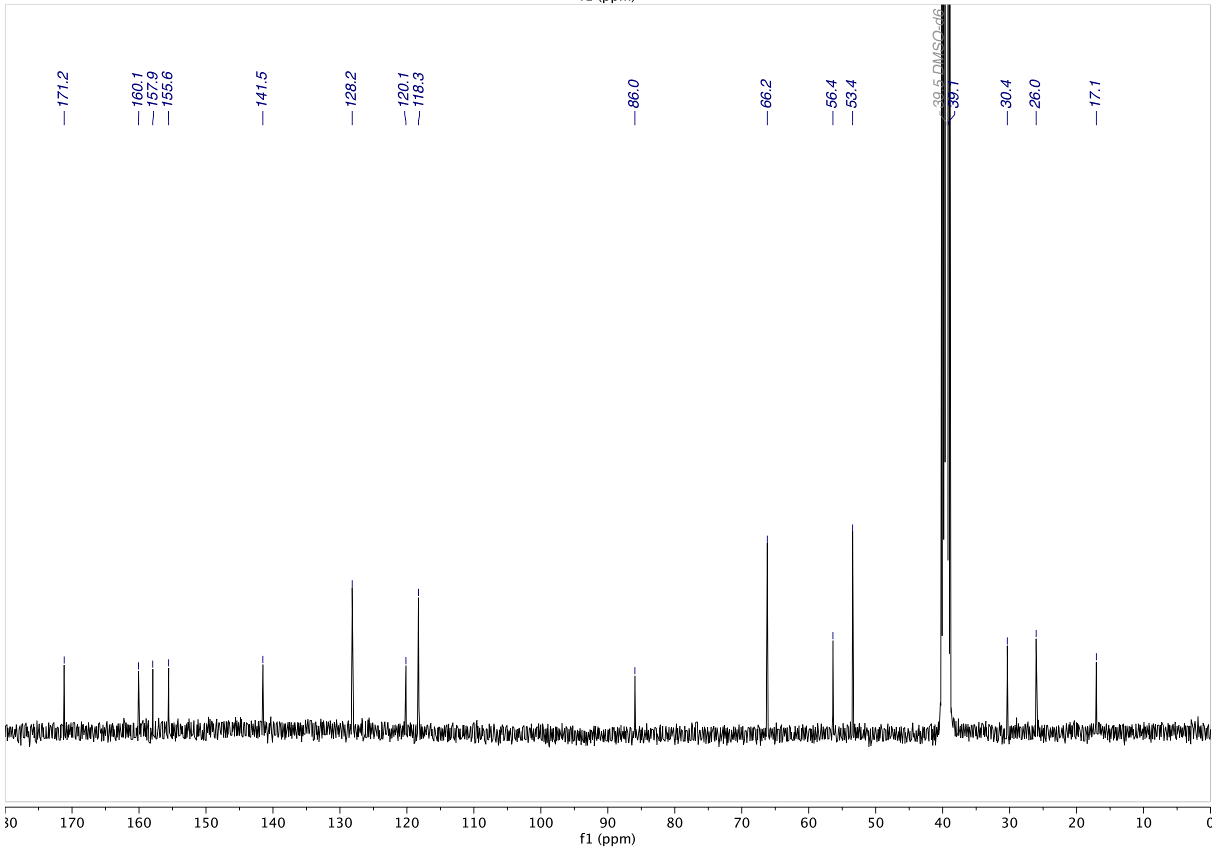

Figure S7. 4-(4-Ethylpiperazin-1-yl)-2-(phenylamino)-5,6-dihydropyrido[2,3-*d*]pyrimidin-7(8*H*)-one (18b)

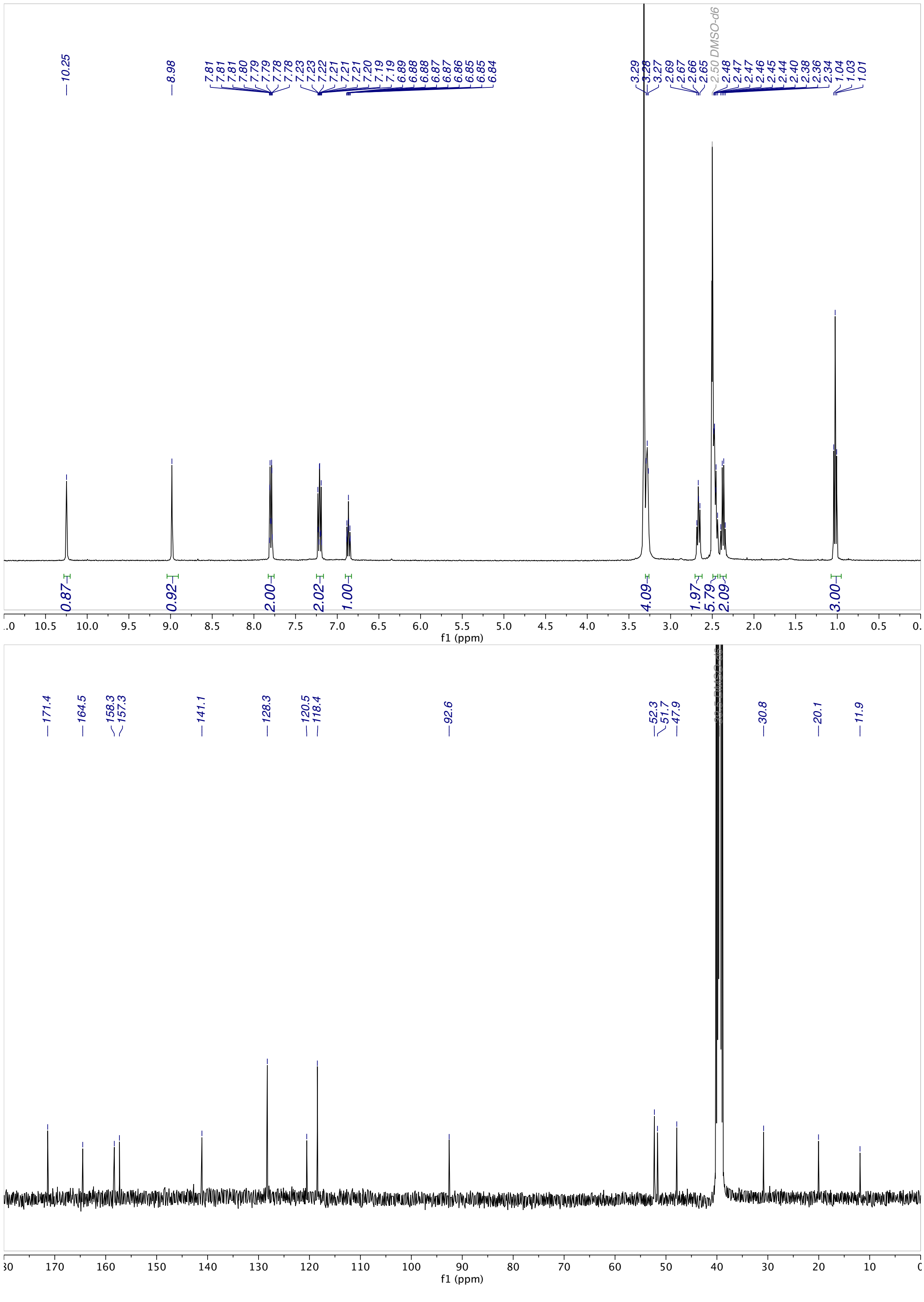

Figure S8. ***Tert*-butyl 4-(7-oxo-2-(phenylamino)-5,6,7,8-tetrahydropyrido[2,3-*d*]pyrimidin-4-yl)piperazine-1-carboxylate (18c)**

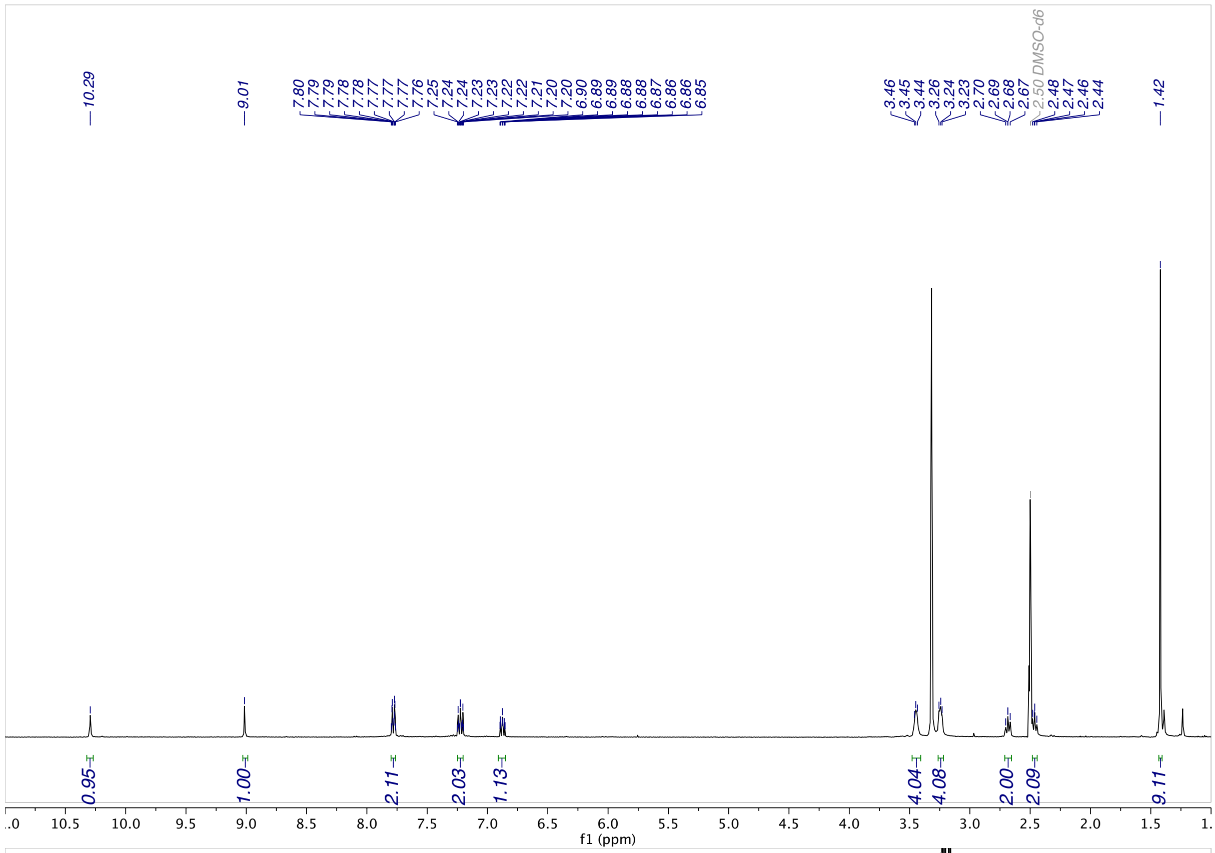

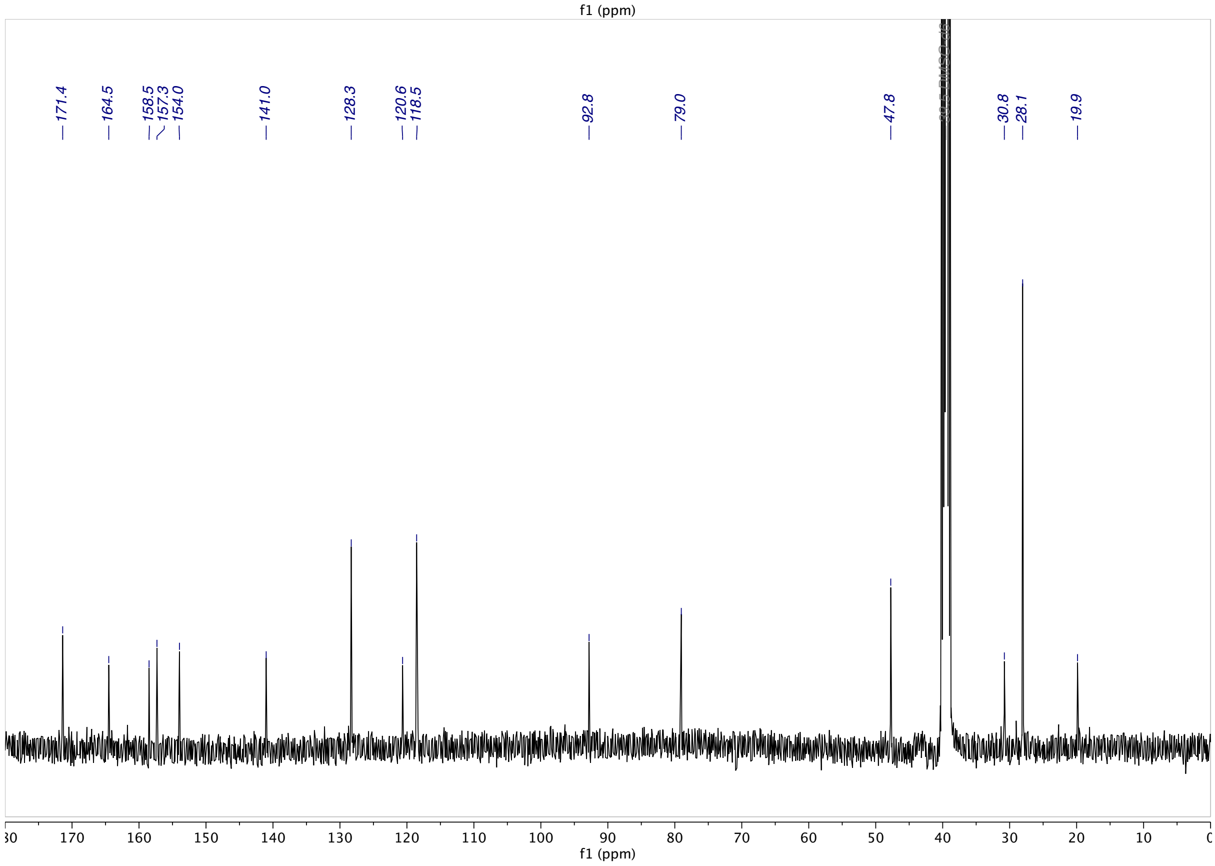

Figure S9. ***Tert*-butyl (1*S*,4*S*)-5-(7-oxo-2-phenylamino)-5,6,7,8-tetrahydropyrido[2,3-*d*]pyrimidin-4-yl)-2,5-diazabicyclo[2.2.1]heptane-2-carboxylate (18d)**

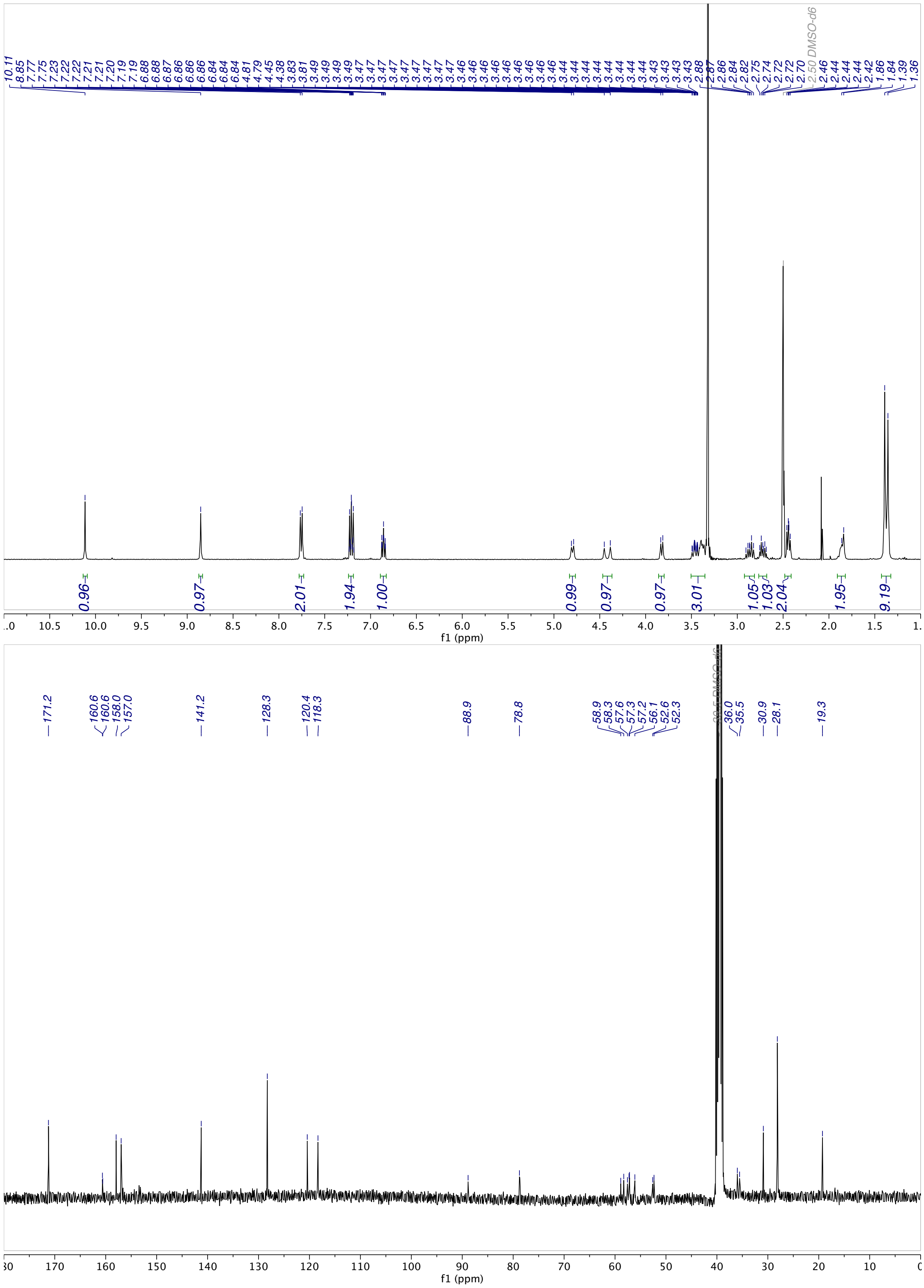

Figure S10. ***Tert*-butyl 7-(7-oxo-2-phenylamino)-5,6,7,8-tetrahydropyrido[2,3-*d*]pyrimidin-4-yl)-2,7-diazaspiro[4.4]nonane-2-carboxylate (18e)**

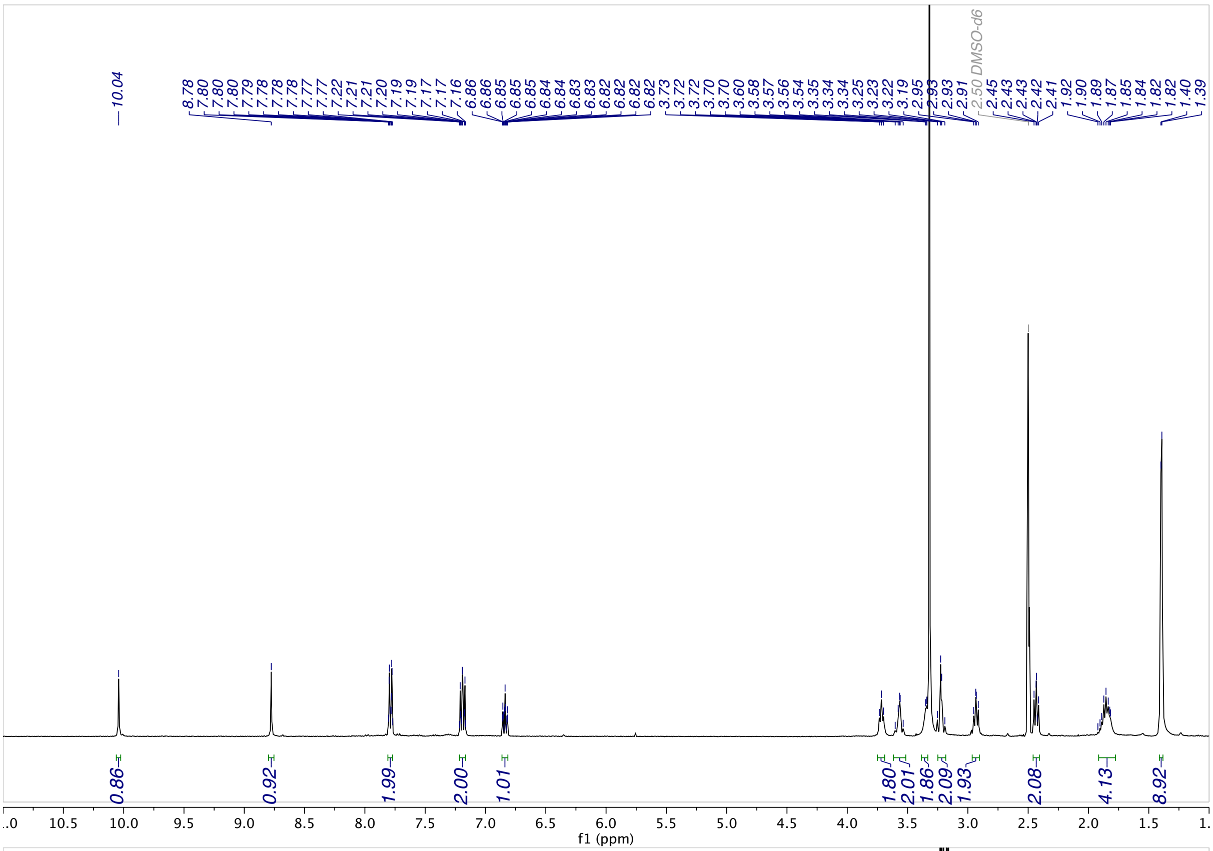

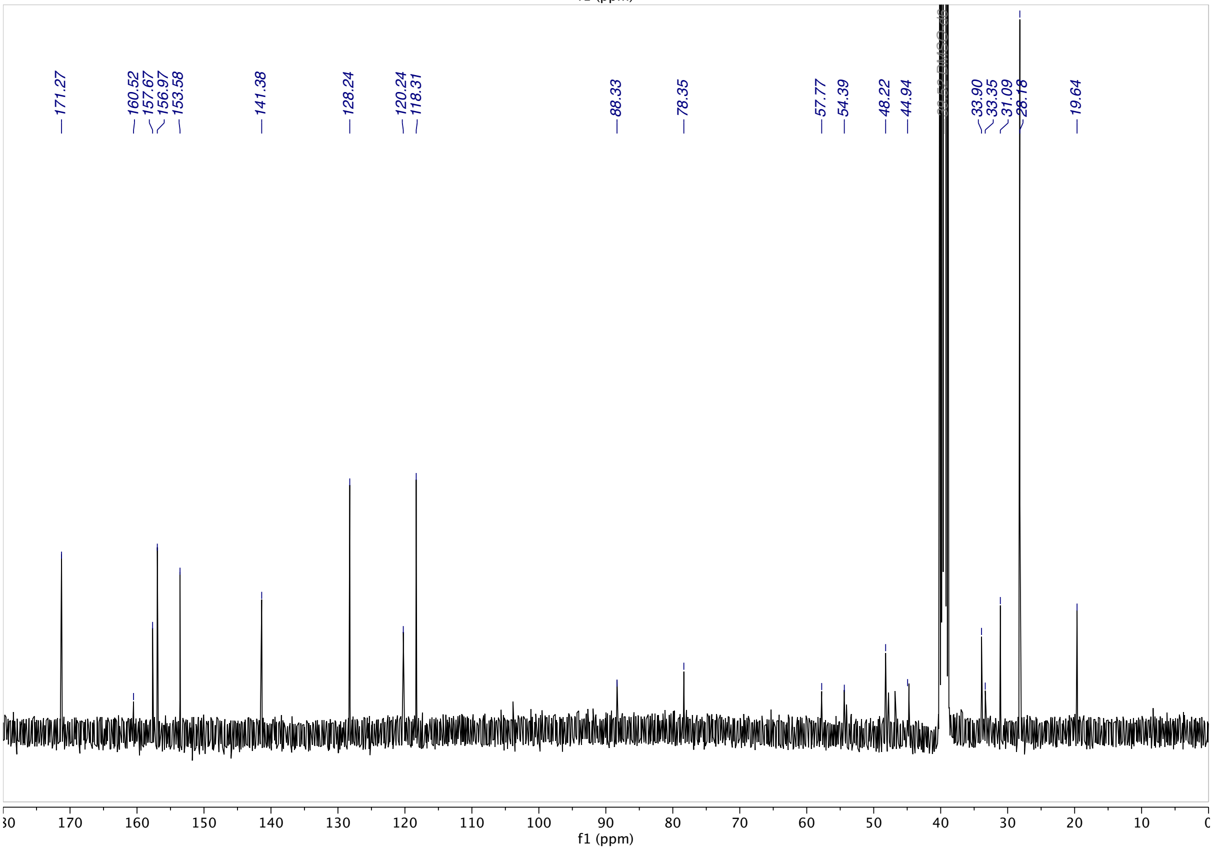

Figure S11. ***Tert*-butyl 7-(7-oxo-2-phenylamino)-5,6,7,8-tetrahydropyrido[2,3-*d*]pyrimidin-4-yl)-2,7-diazaspiro[3.5]nonane-2-carboxylate (18f)**

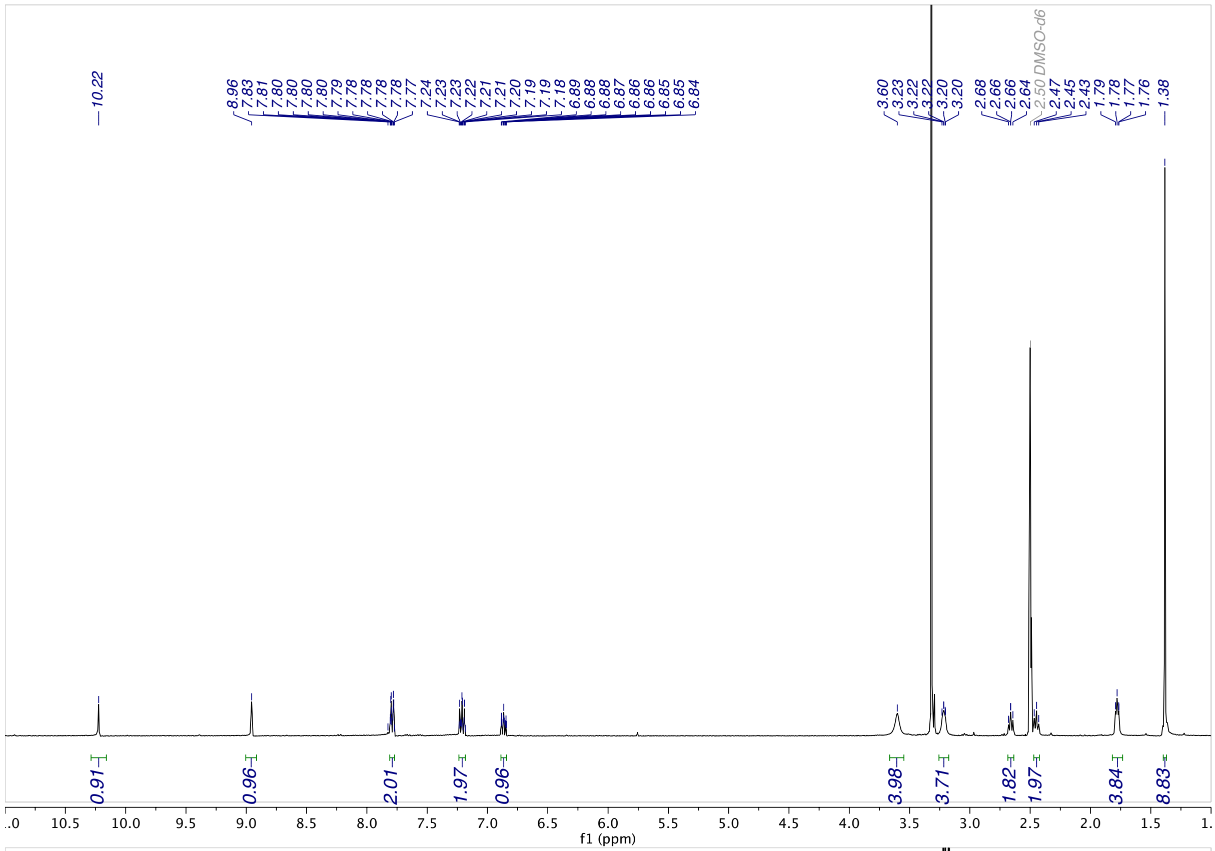

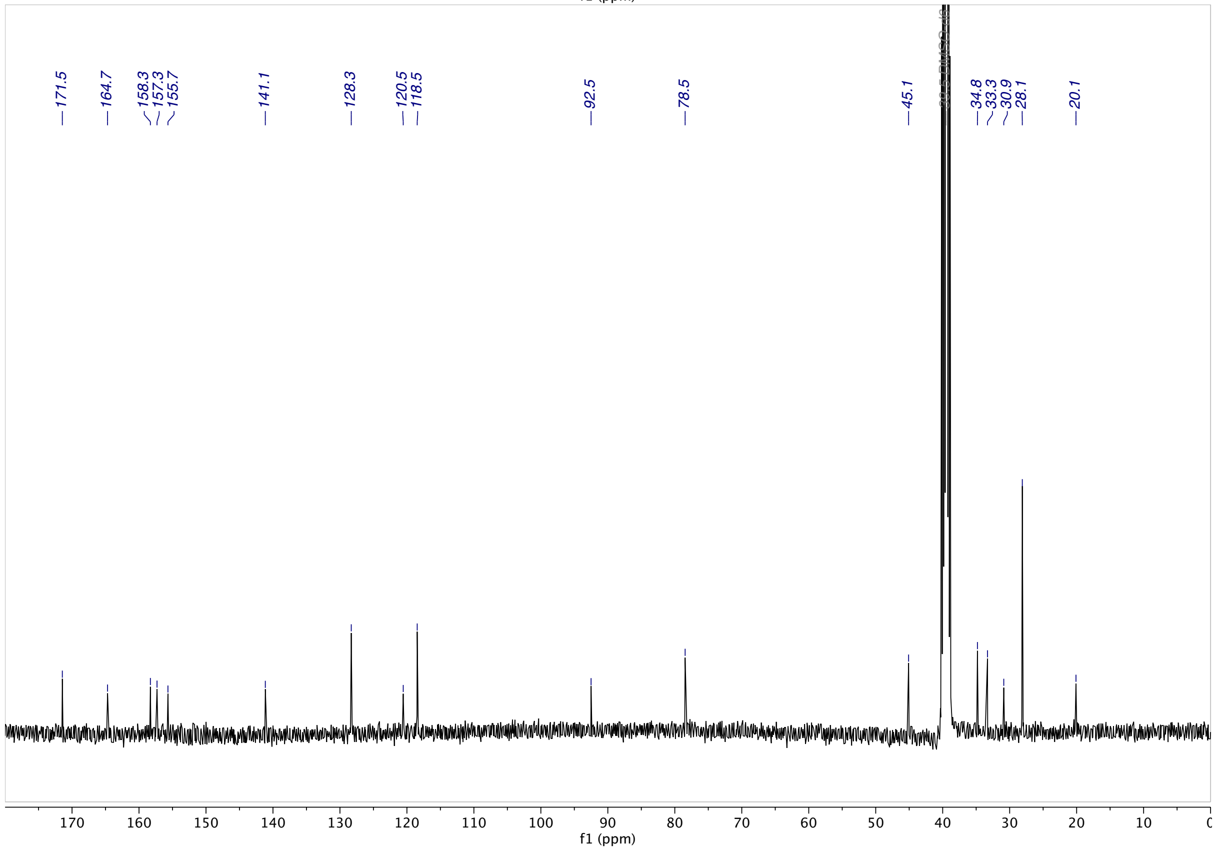

Figure S12. ***Tert*-butyl (3a*R*,6a*S*)-5-(7-oxo-2-(phenylamino)-5,6,7,8-tetrahydropyrido[2,3-*d*]pyrimidin-4-yl)hexahydropyrrolo[3,4-*c*]pyrrole-2(1*H*)-carboxylate (18g)**

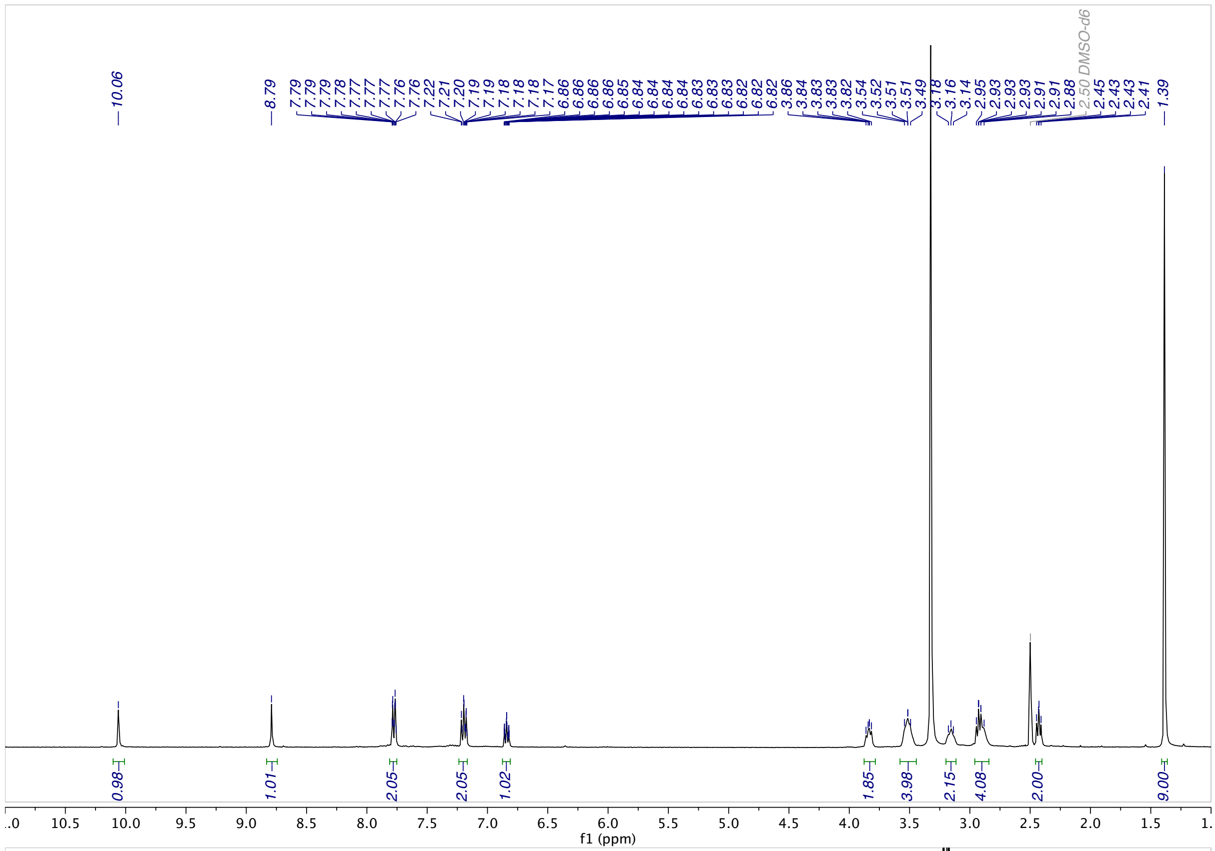

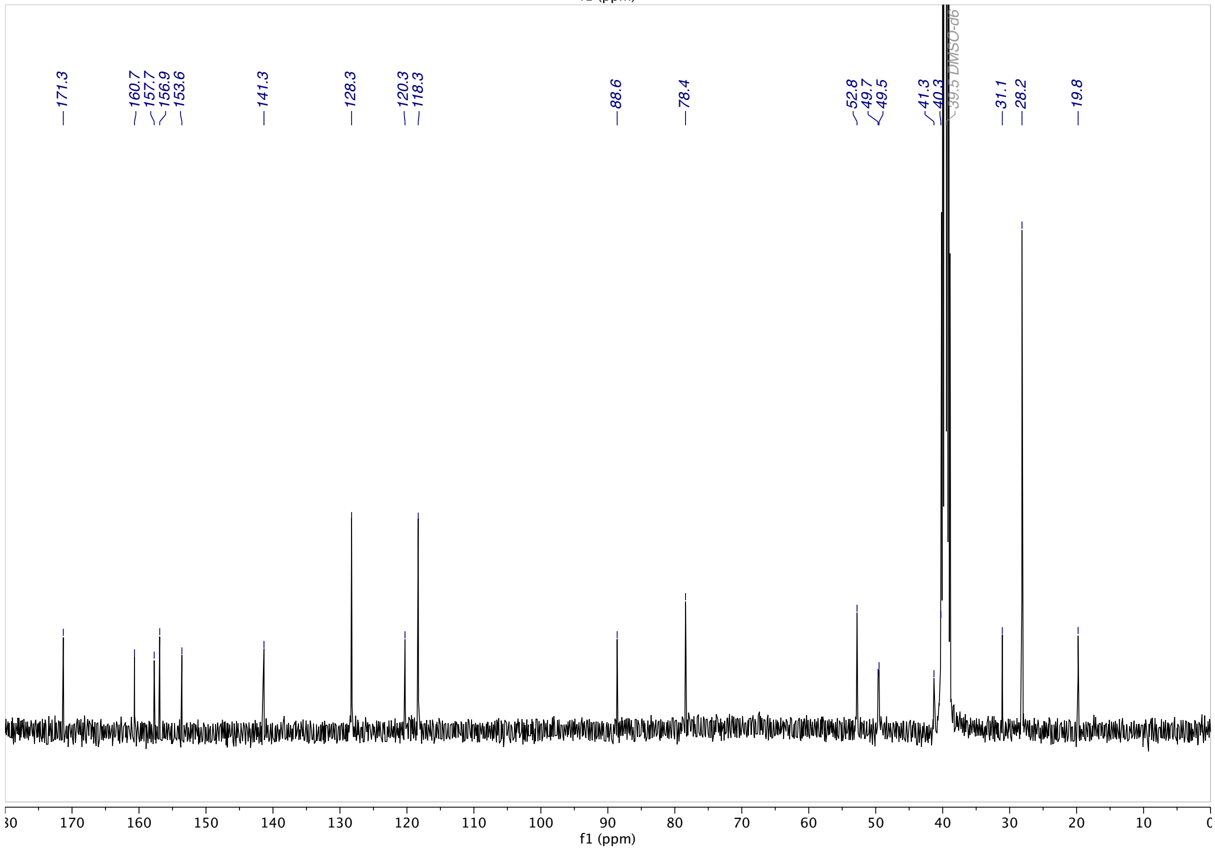

Figure S13. ***Tert*-butyl 3-(((7-oxo-2-(phenylamino)-5,6,7,8-tetrahydropyrido[2,3-*d*]pyrimidin-4-yl)amino)methyl)azetidine-1-carboxylate (18h)**

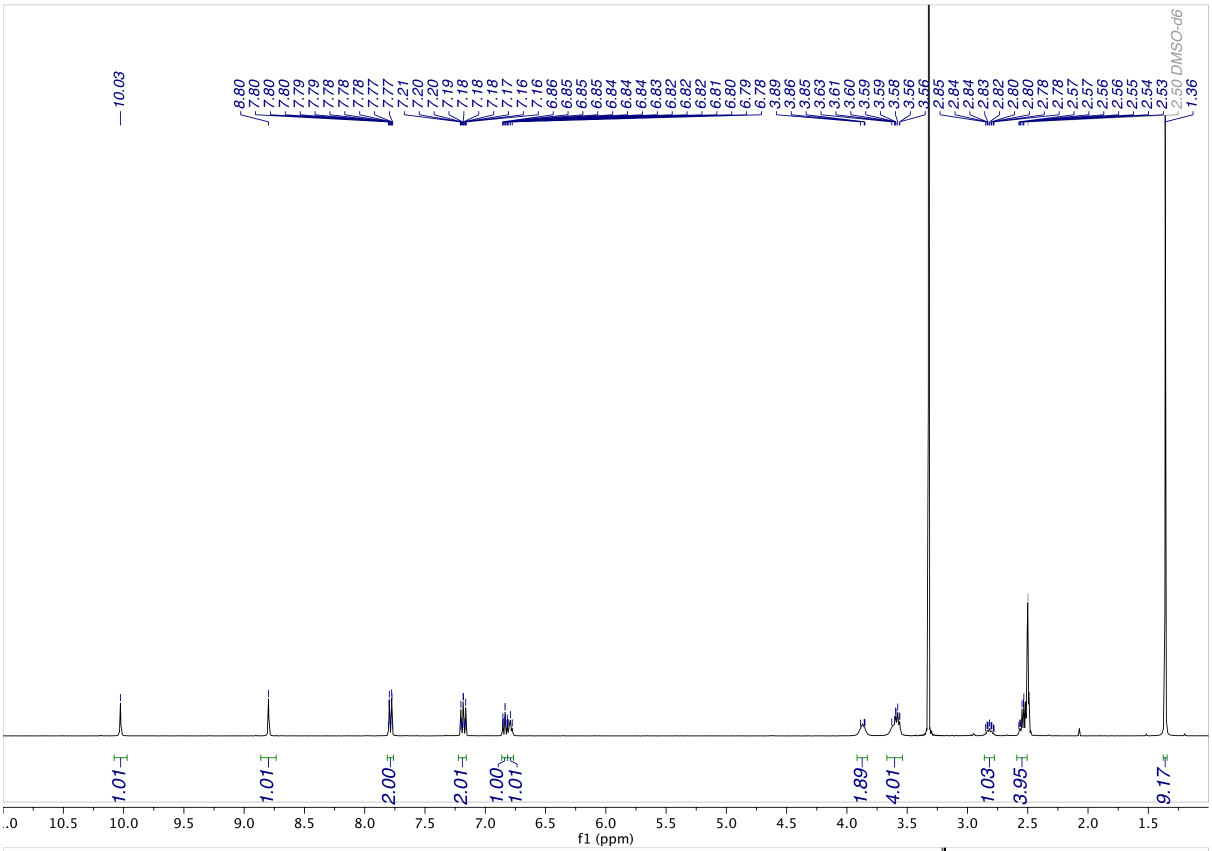

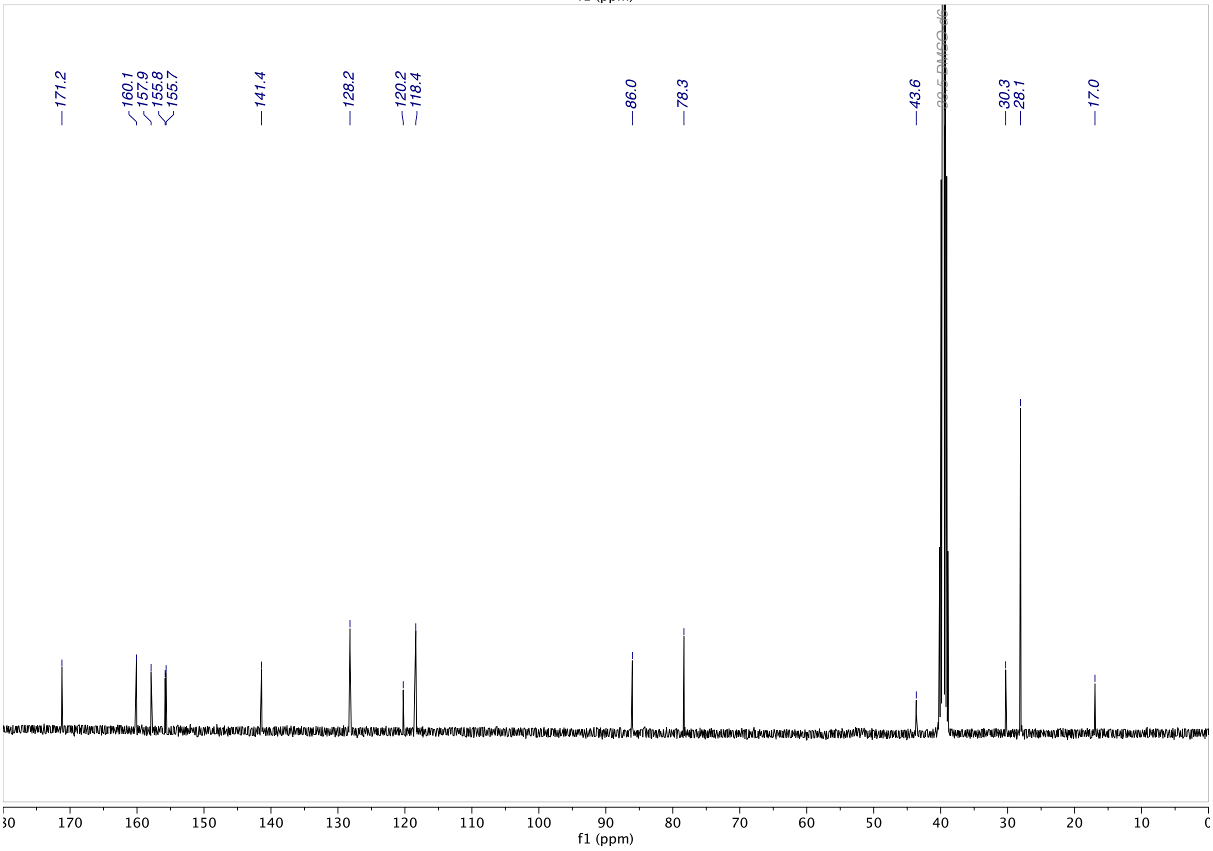

Figure S14. 2-(Phenylamino)-4-((3,4,5-trimethoxyphenyl)amino)-5,6-dihydropyrido[2,3-*d*]pyrimidin-7(8*H*)-one (19a)

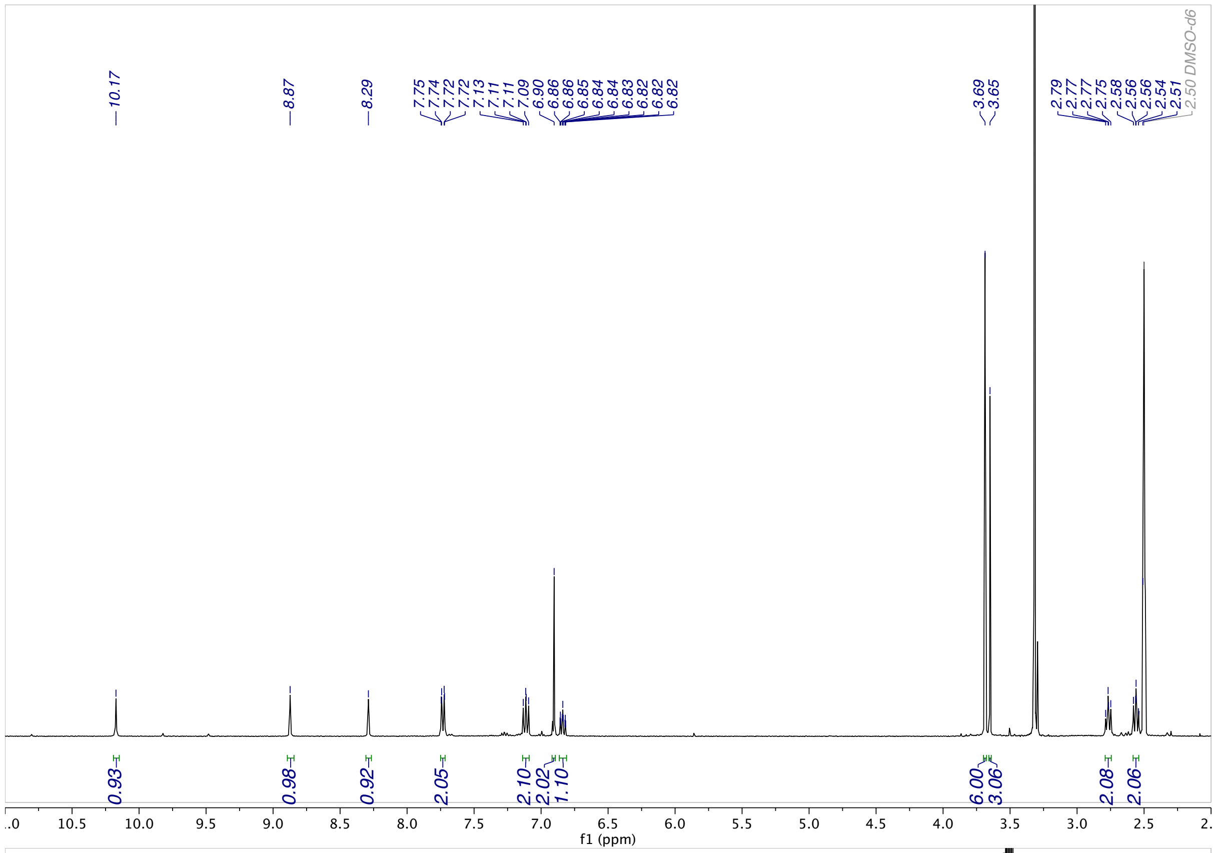

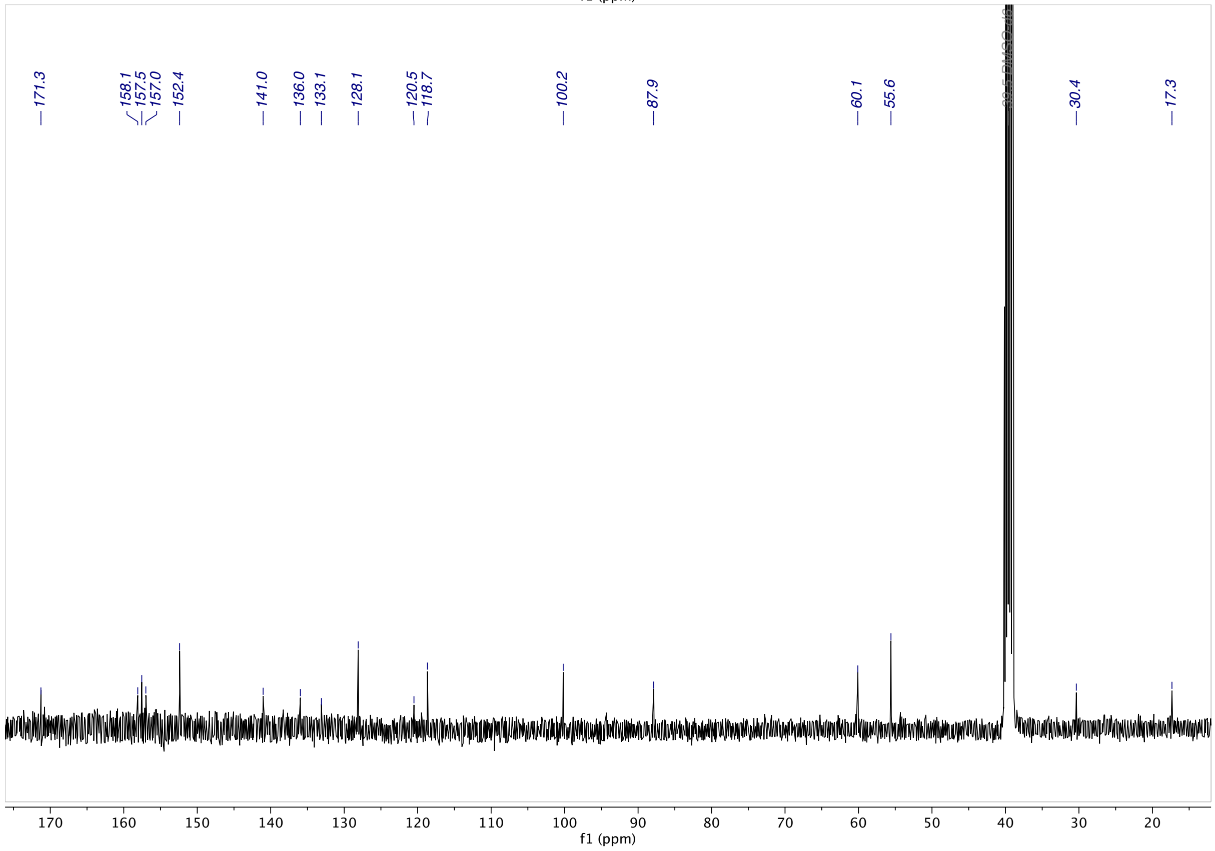

Figure S15. 4-((4-Morpholinophenyl)amino)-2-(phenylamino)-5,6-dihydropyrido[2,3-*d*]pyrimidin-7(8*H*)-one (19b)

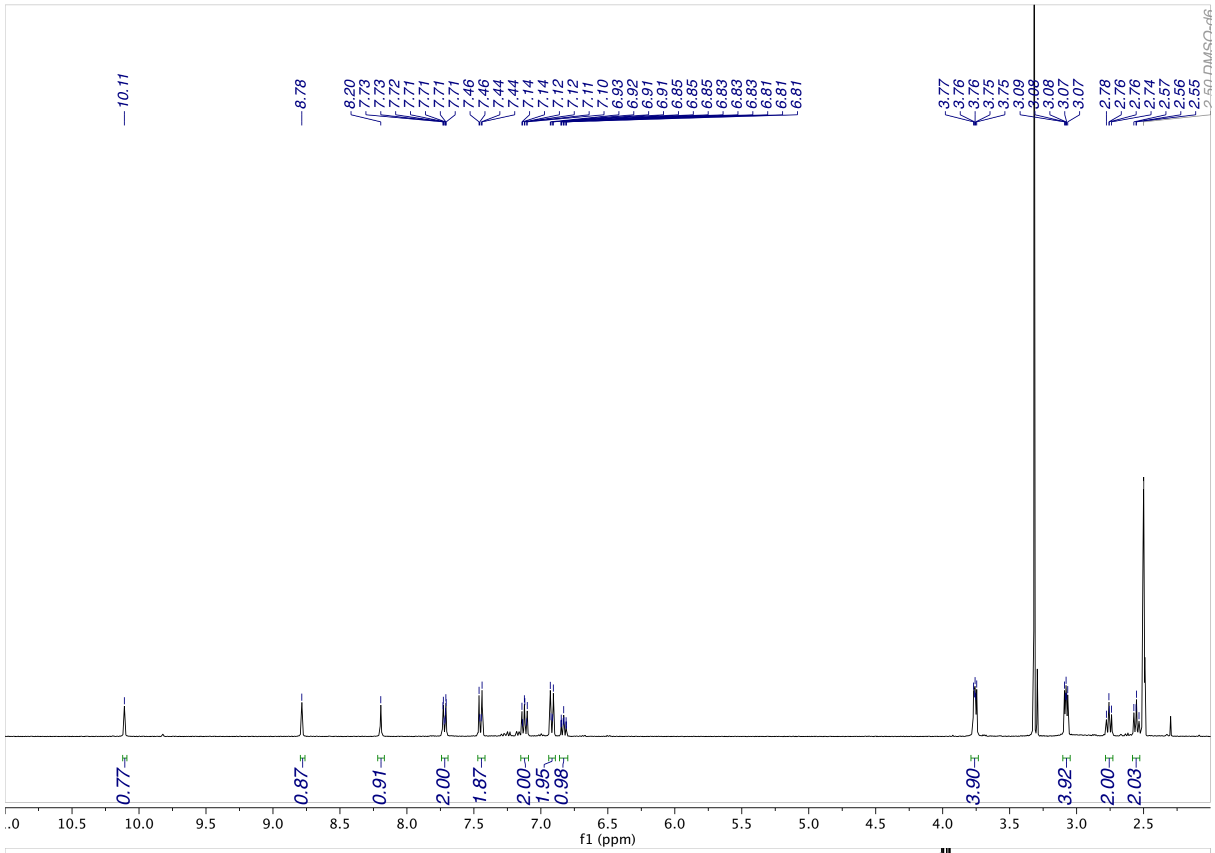

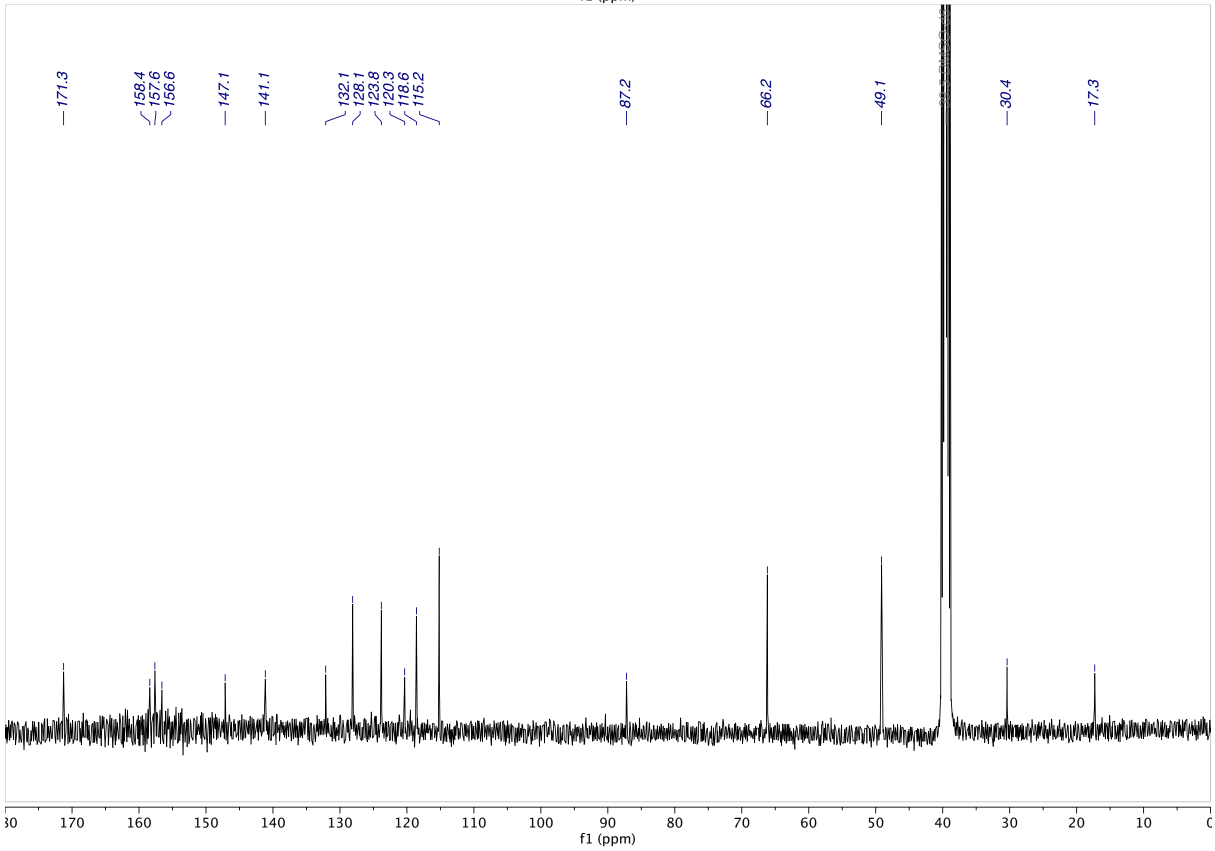

Figure S16. 4-((7-Oxo-2-(phenylamino)-5,6,7,8-tetrahydropyrido[2,3-*d*]pyrimidin-4-yl)amino)benzonitrile (19c)

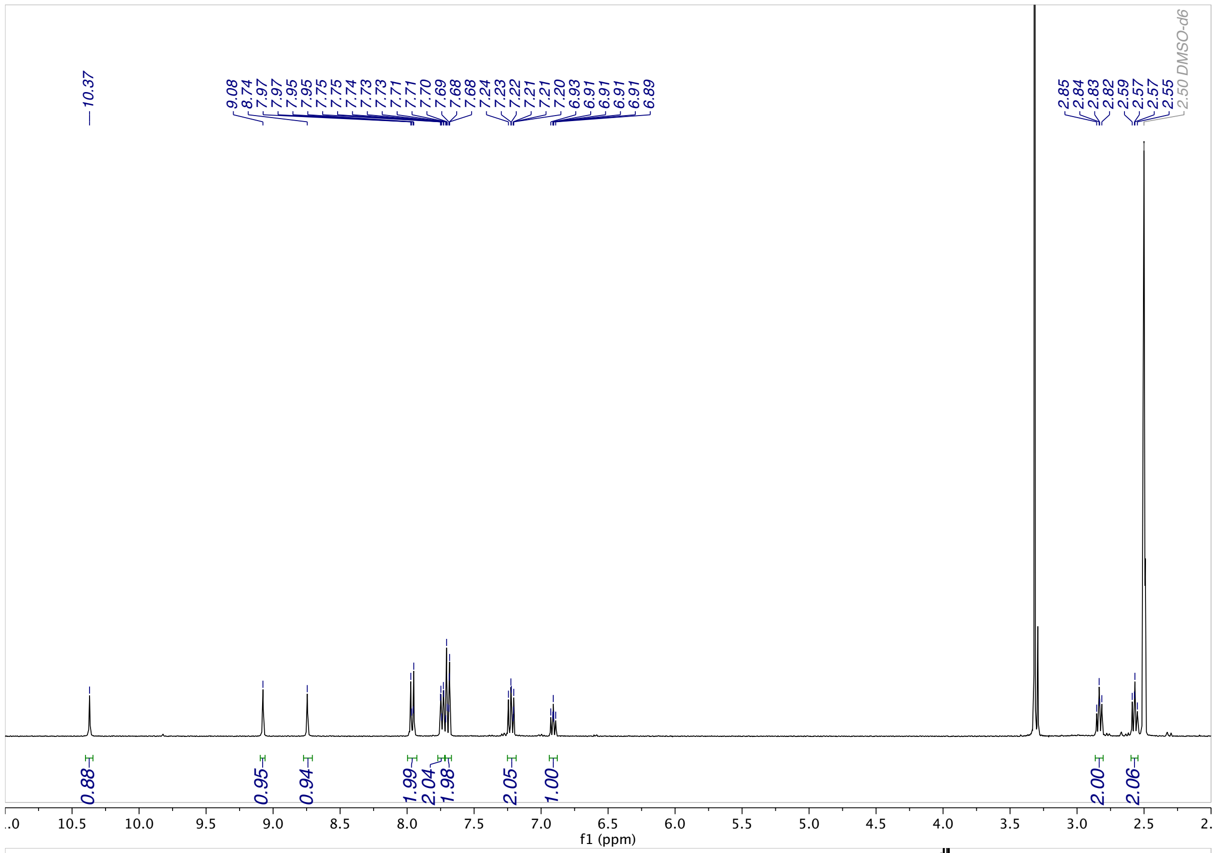

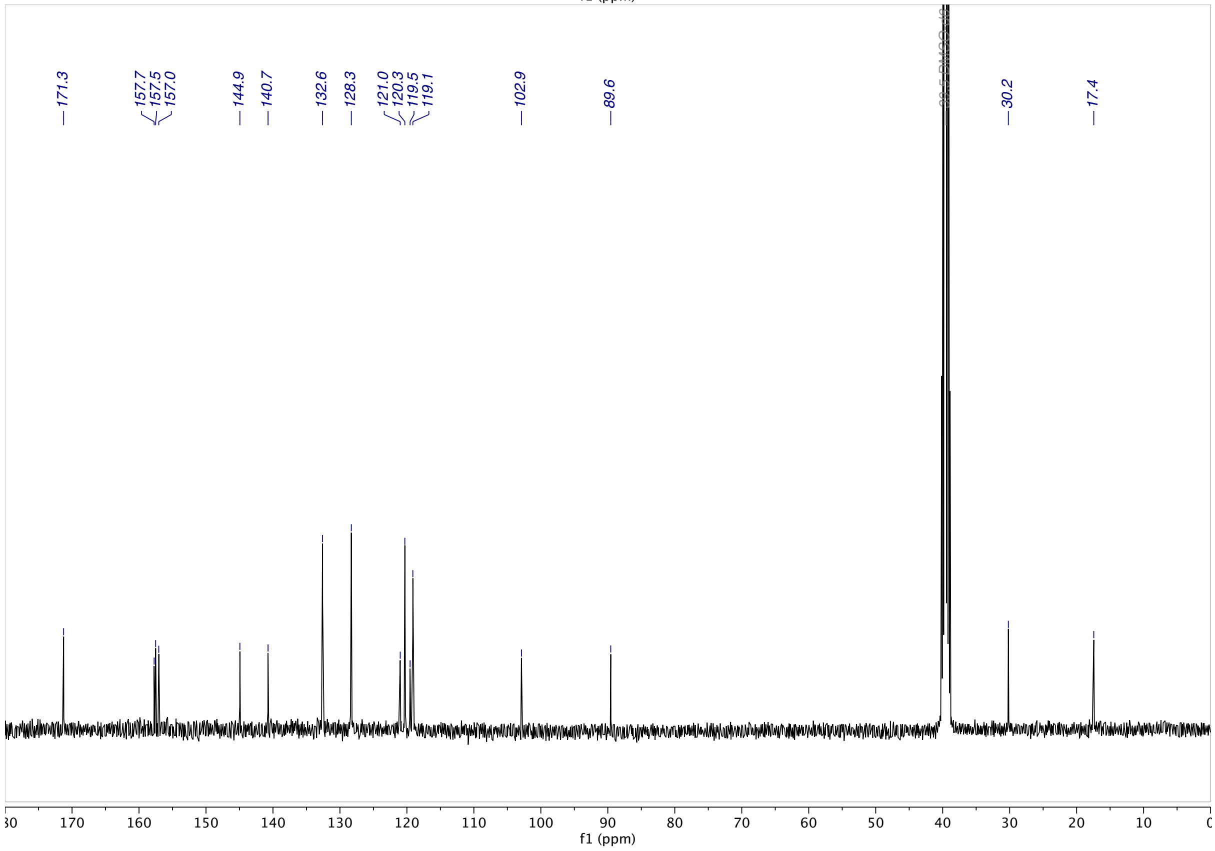

Figure S17. 4-((4-(4-Methylpiperazin-1-yl)phenyl)amino)-2-(phenylamino)-5,6-dihydropyrido[2,3-*d*]pyrimidin-7(8*H*)-one (19d)

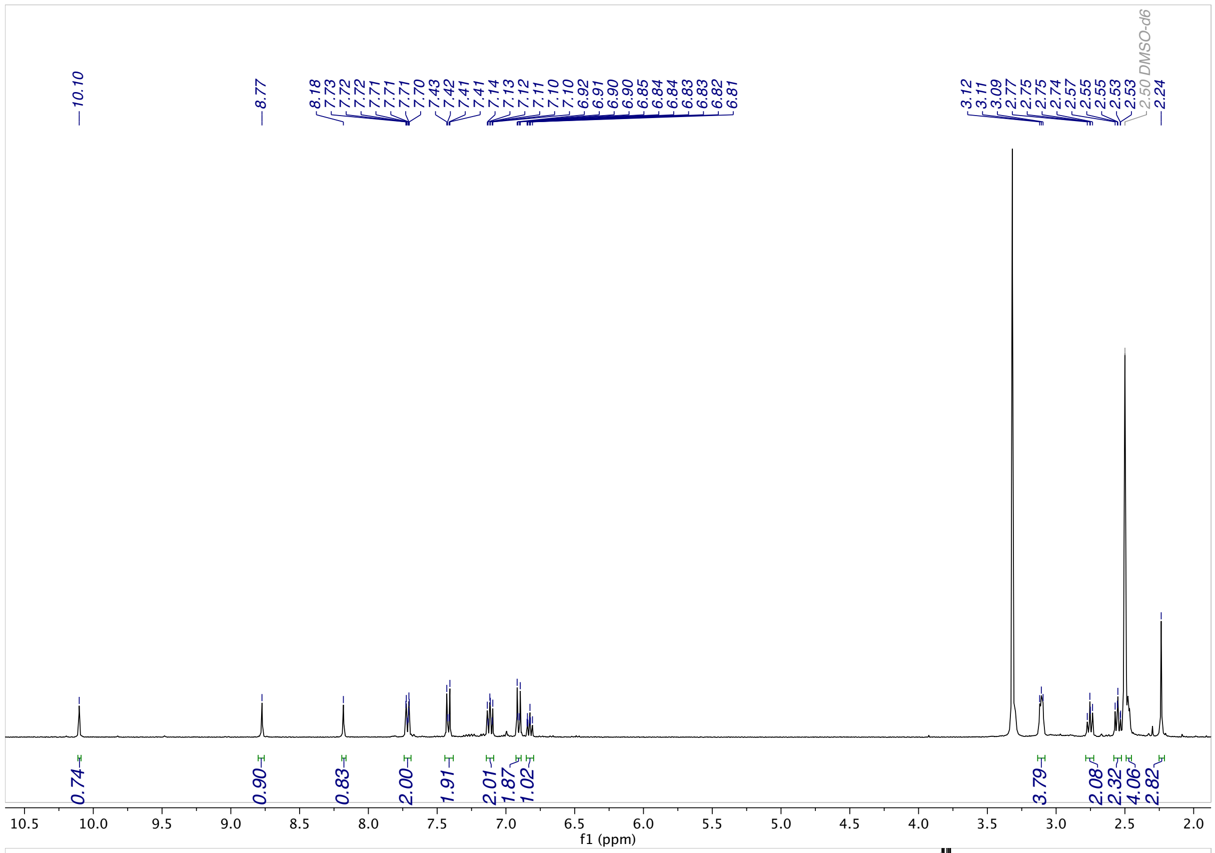

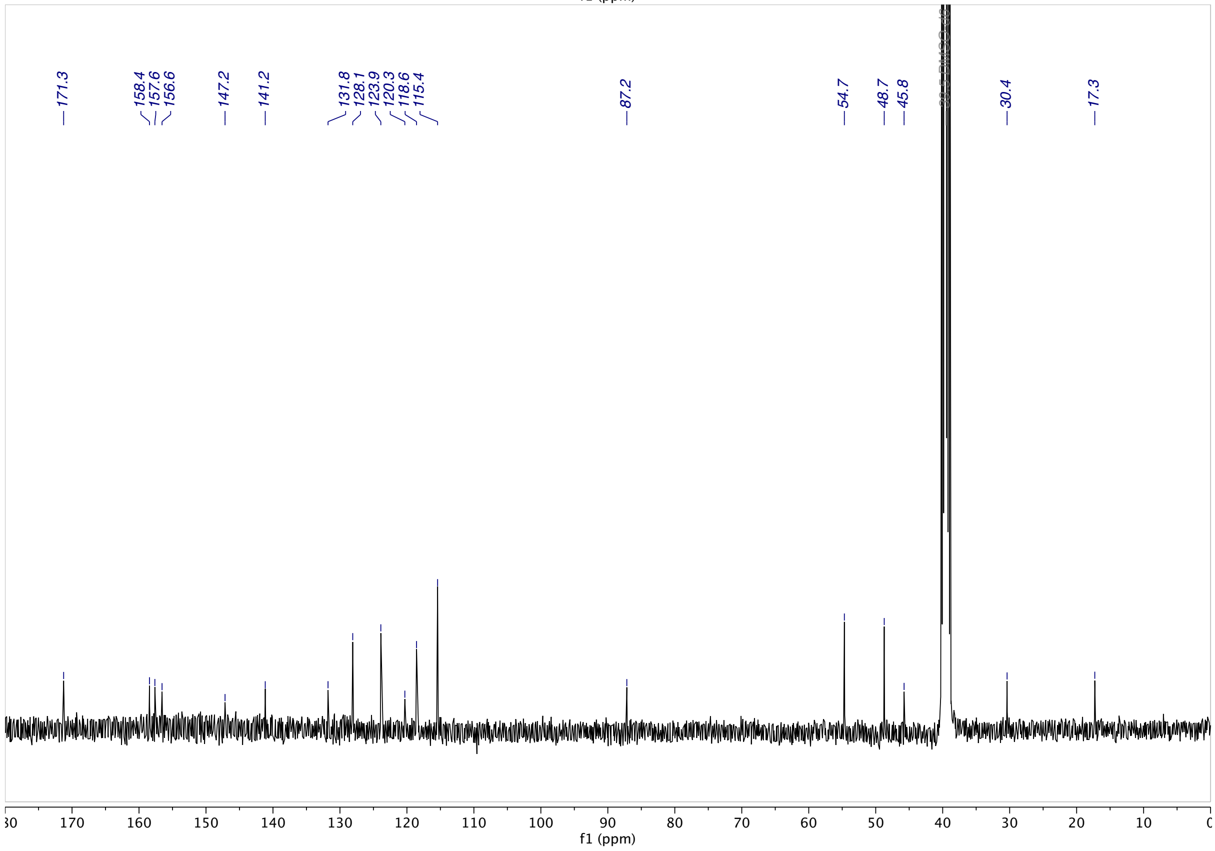

Figure S18. 4-((4-(Dimethylamino)phenyl)amino)-2-(phenylamino)-5,6-dihydropyrido[2,3-*d*]pyrimidin-7(8*H*)-one (19e)

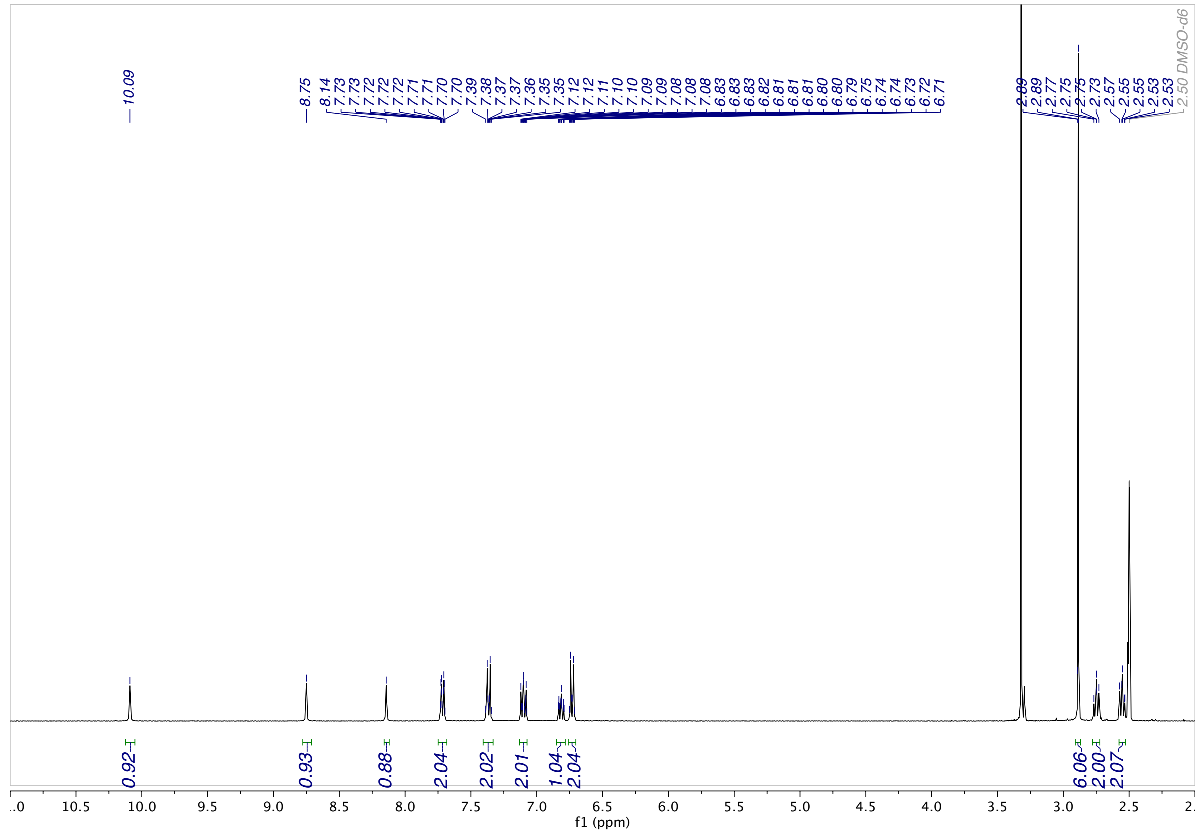

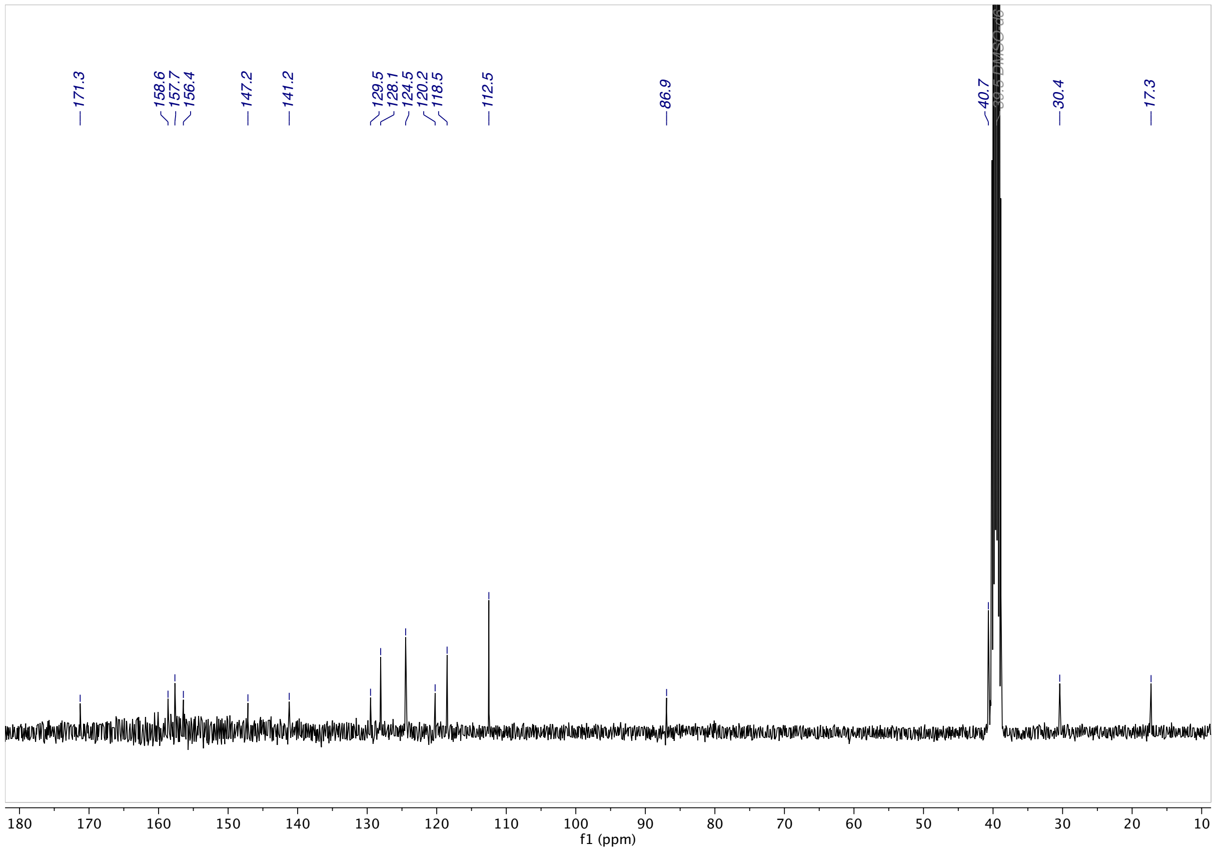

Figure S19. 4-((4-Nitrophenyl)amino)-2-(phenylamino)-5,6-dihydropyrido[2,3-*d*]pyrimidin-7(8*H*)-one (19f)

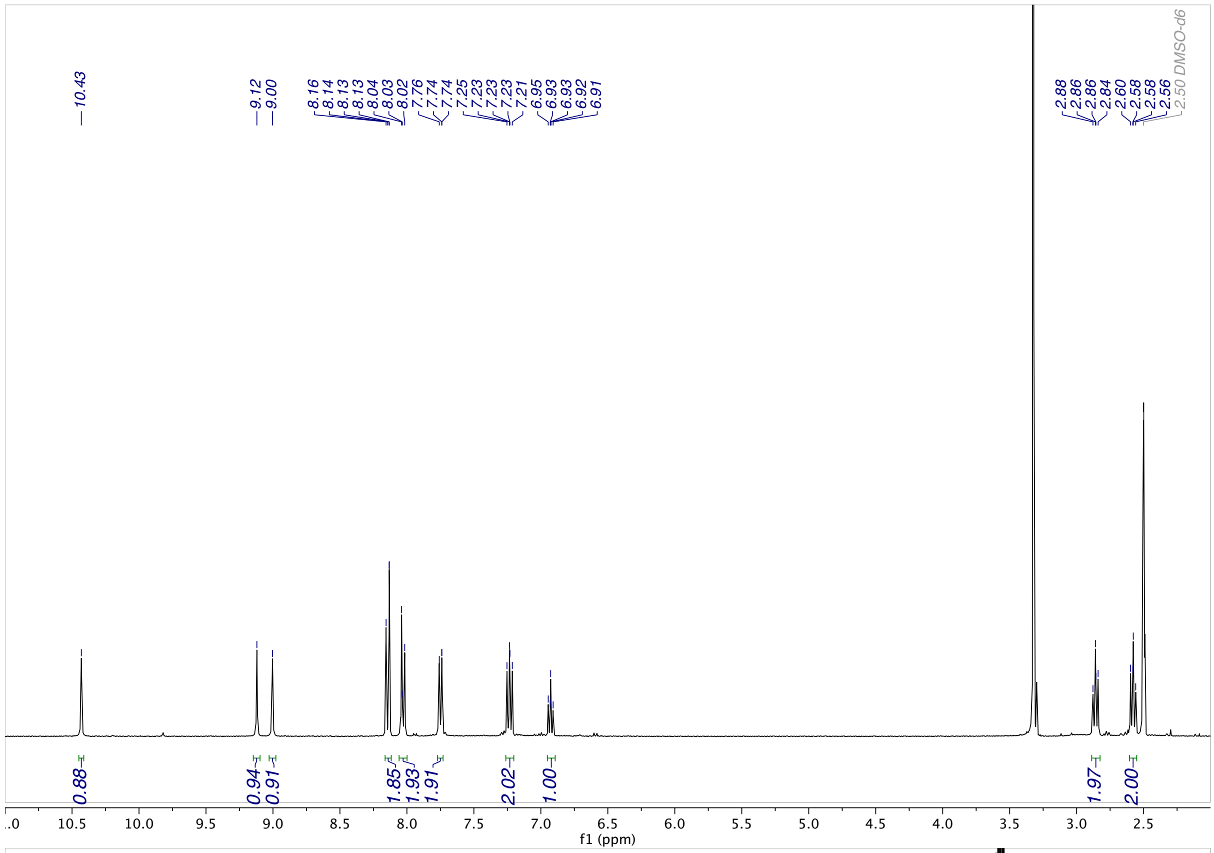

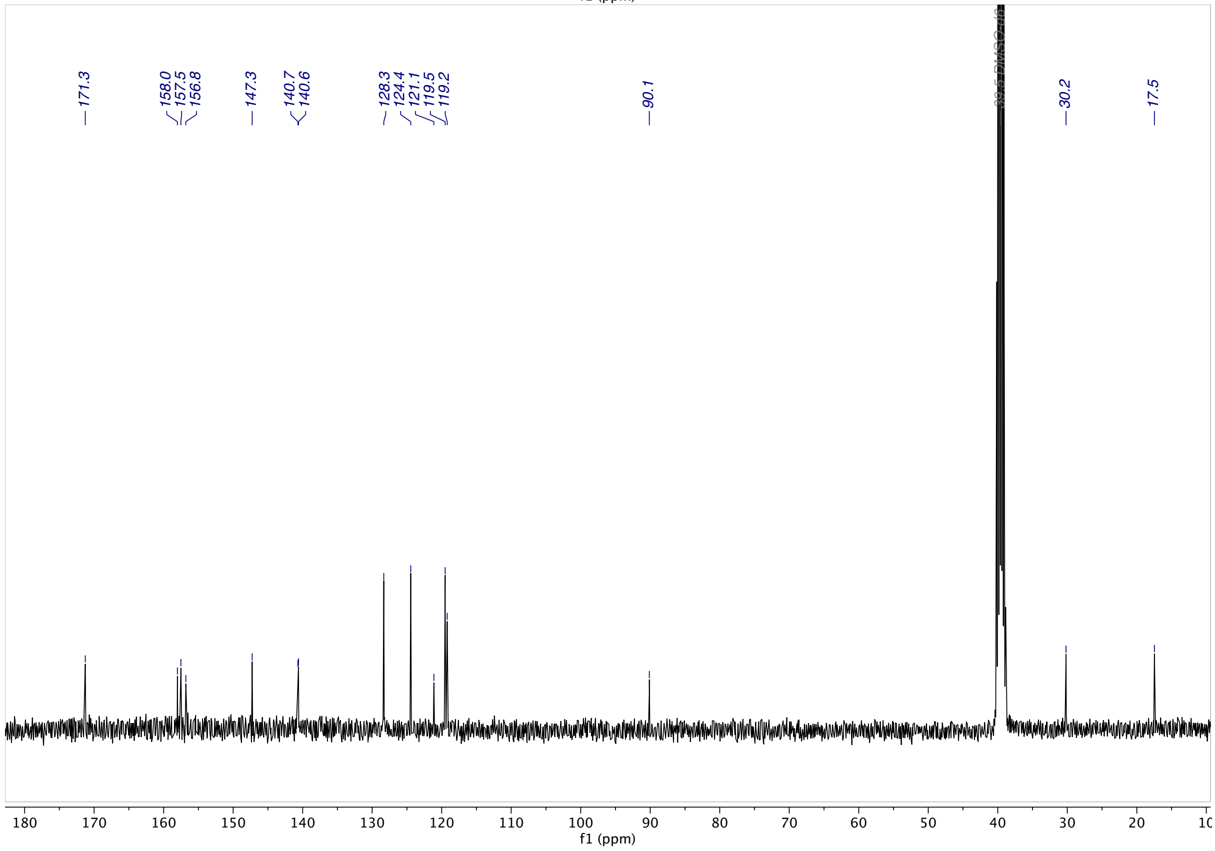

Figure S20. Methyl 3-((7-oxo-2-(phenylamino)-5,6,7,8-tetrahydropyrido[2,3-*d*]pyrimidin-4-yl)amino)thiophene-2-carboxylate (19g)

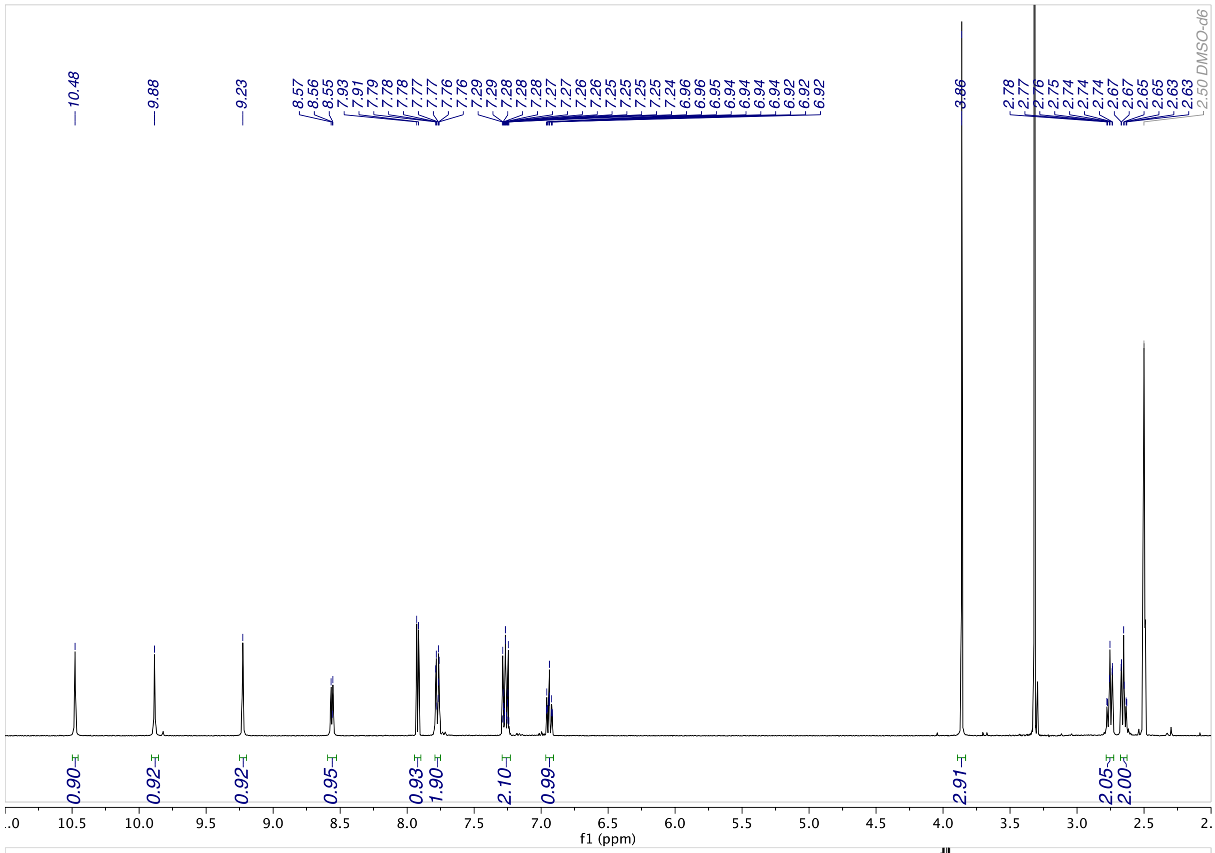

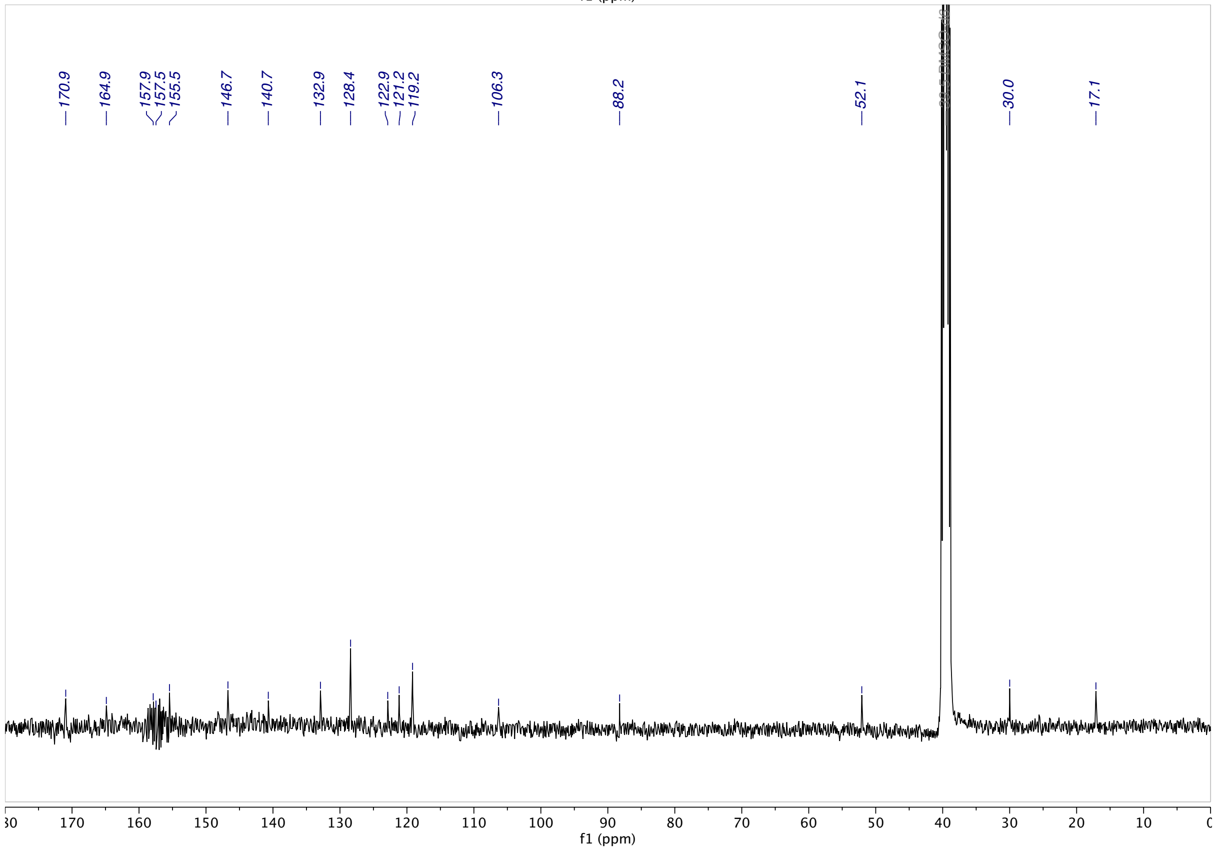

Figure S21. 2-(Phenylamino)-4-((3-(trifluoromethyl) phenyl)amino)-5,6-dihydropyrido[2,3-*d*]pyrimidin-7(8*H*)-one (19h)

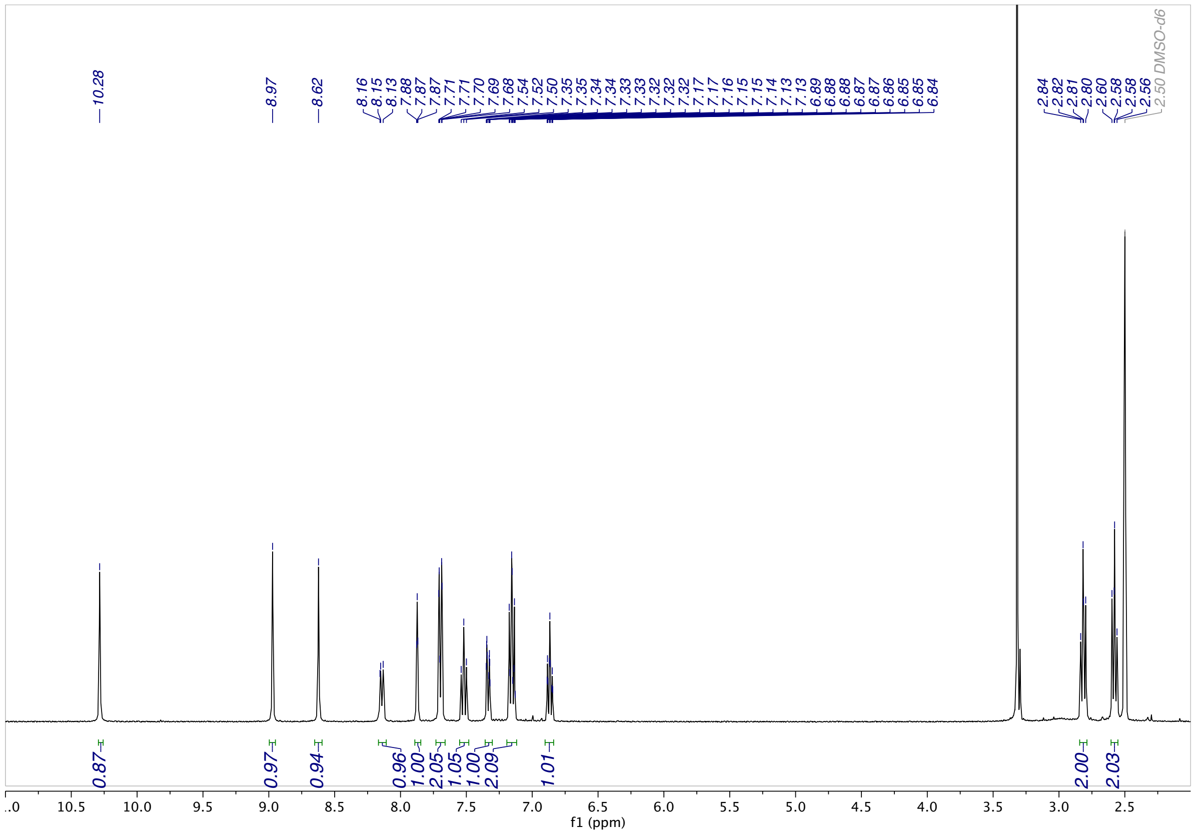

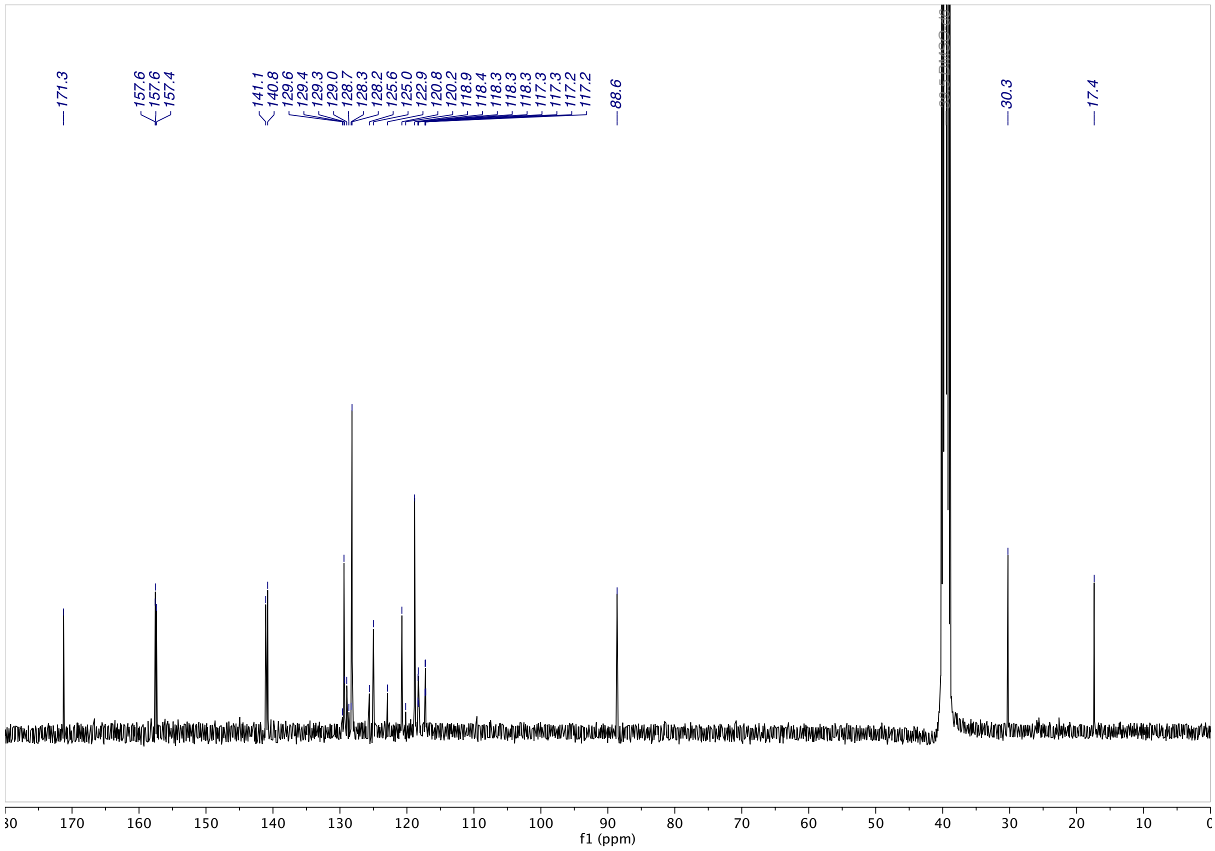

Figure S22. ***Tert*-butyl (4-((7-oxo-2-(phenylamino)-5,6,7,8-tetrahydropyrido[2,3-*d*]pyrimidin-4-yl)amino)benzyl)carbamate (19i)**

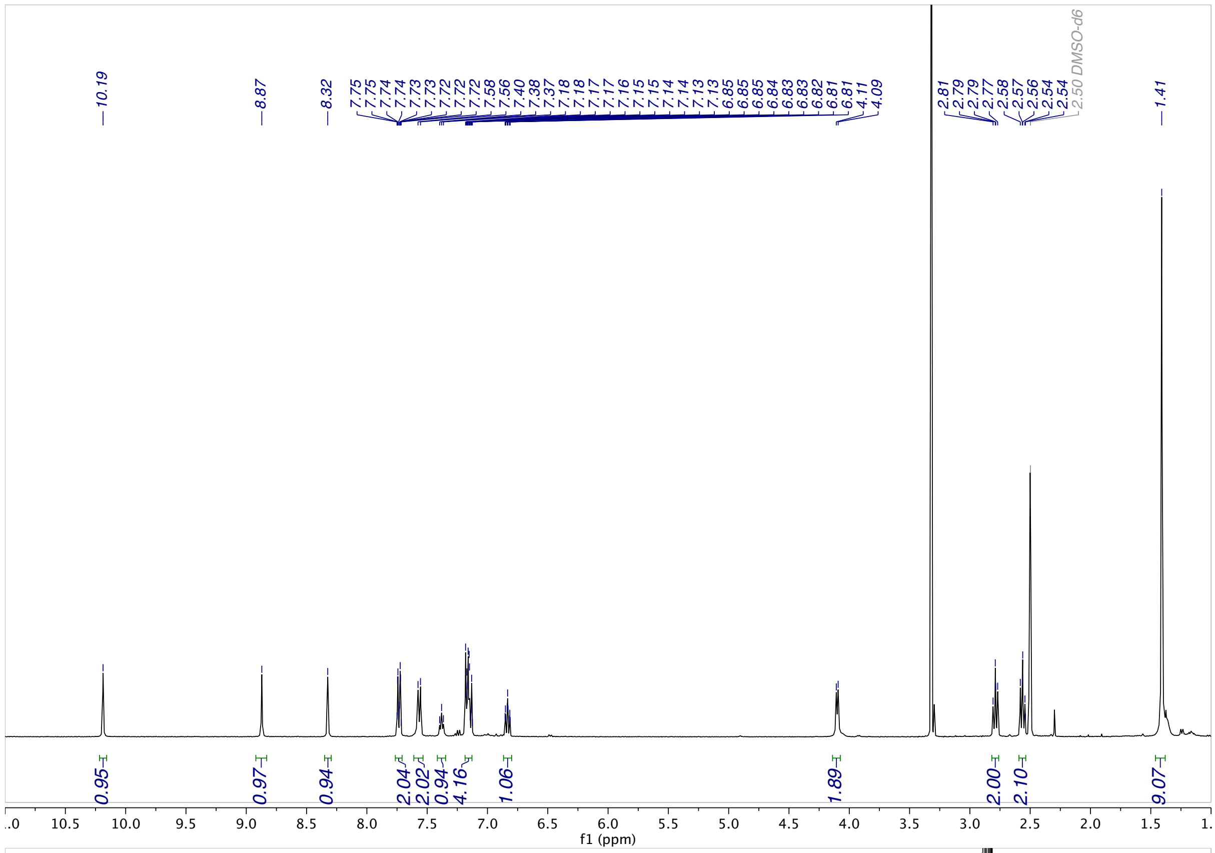

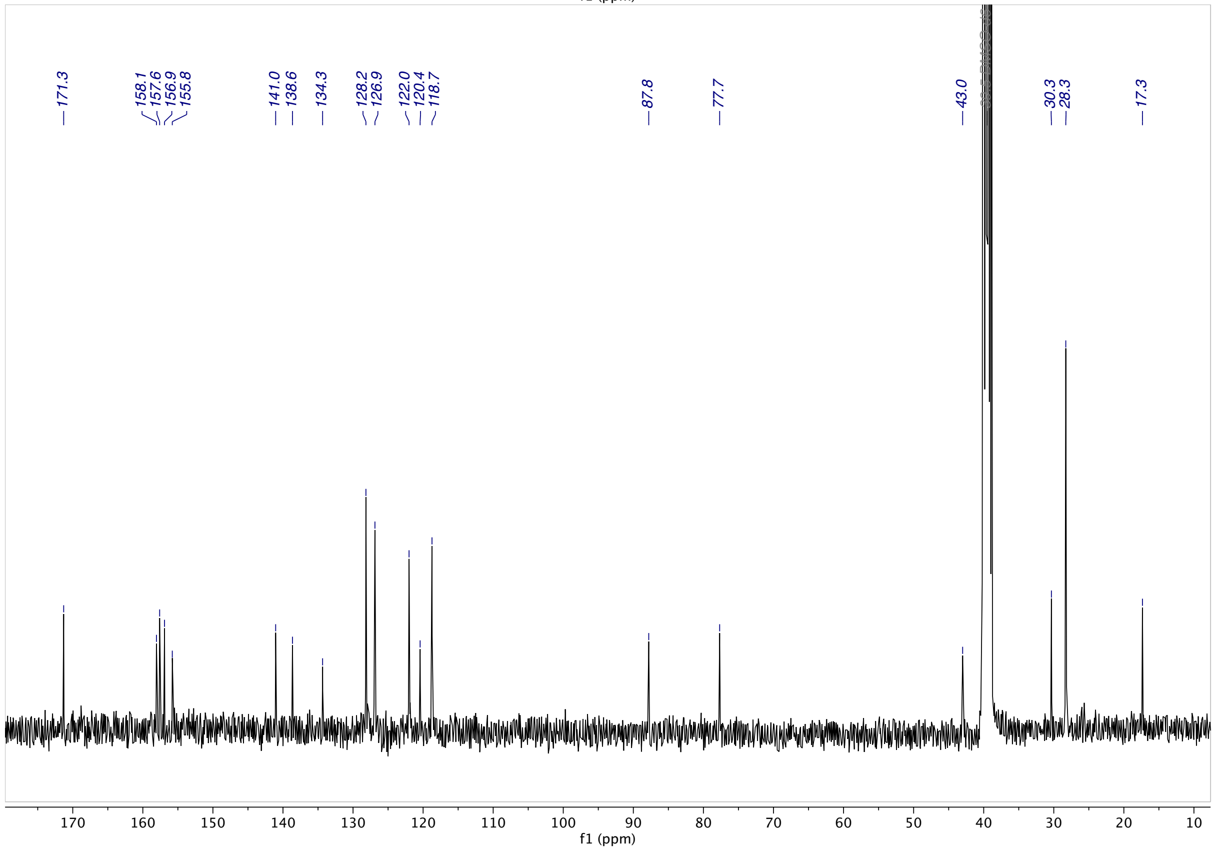

Figure S23. 4-((3-Nitrophenyl)amino)-2-(phenylamino)-5,6-dihydropyrido[2,3-*d*]pyrimidin-7(8*H*)-one (19j)

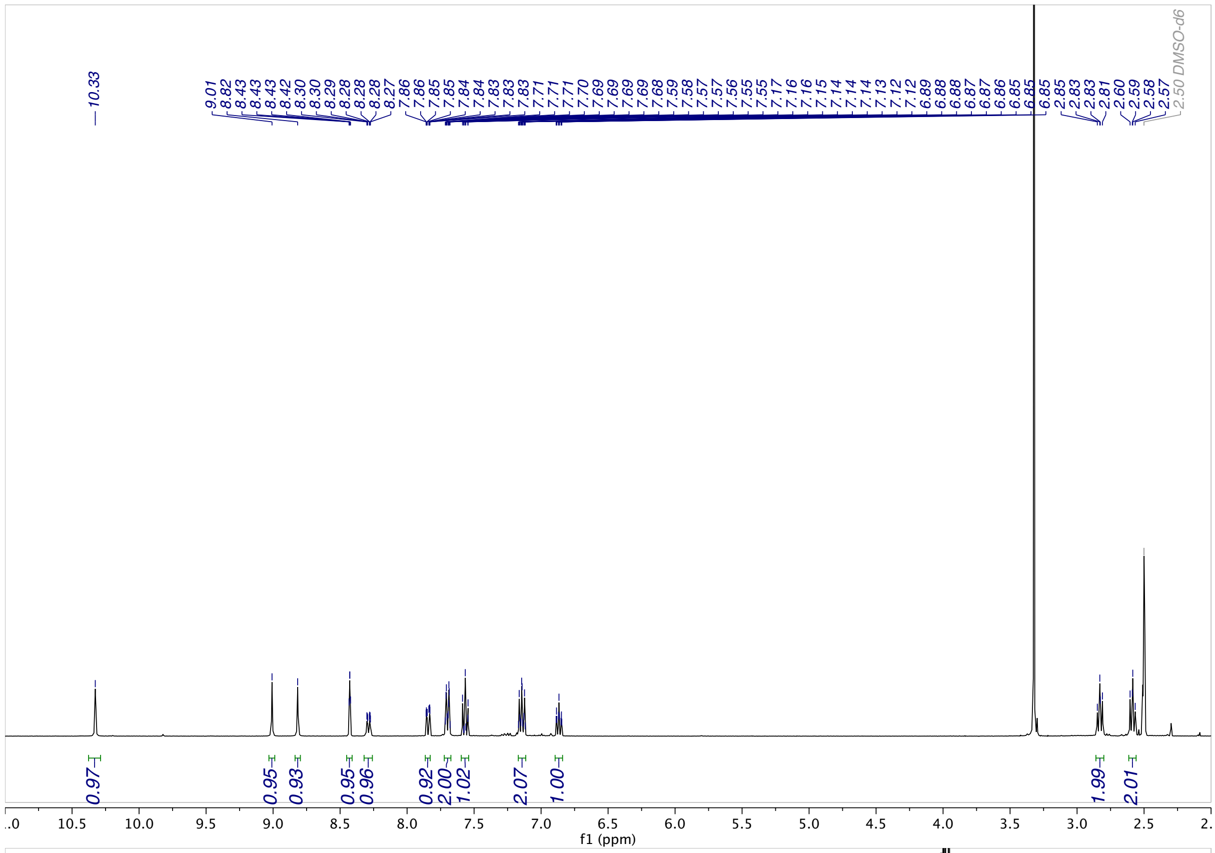

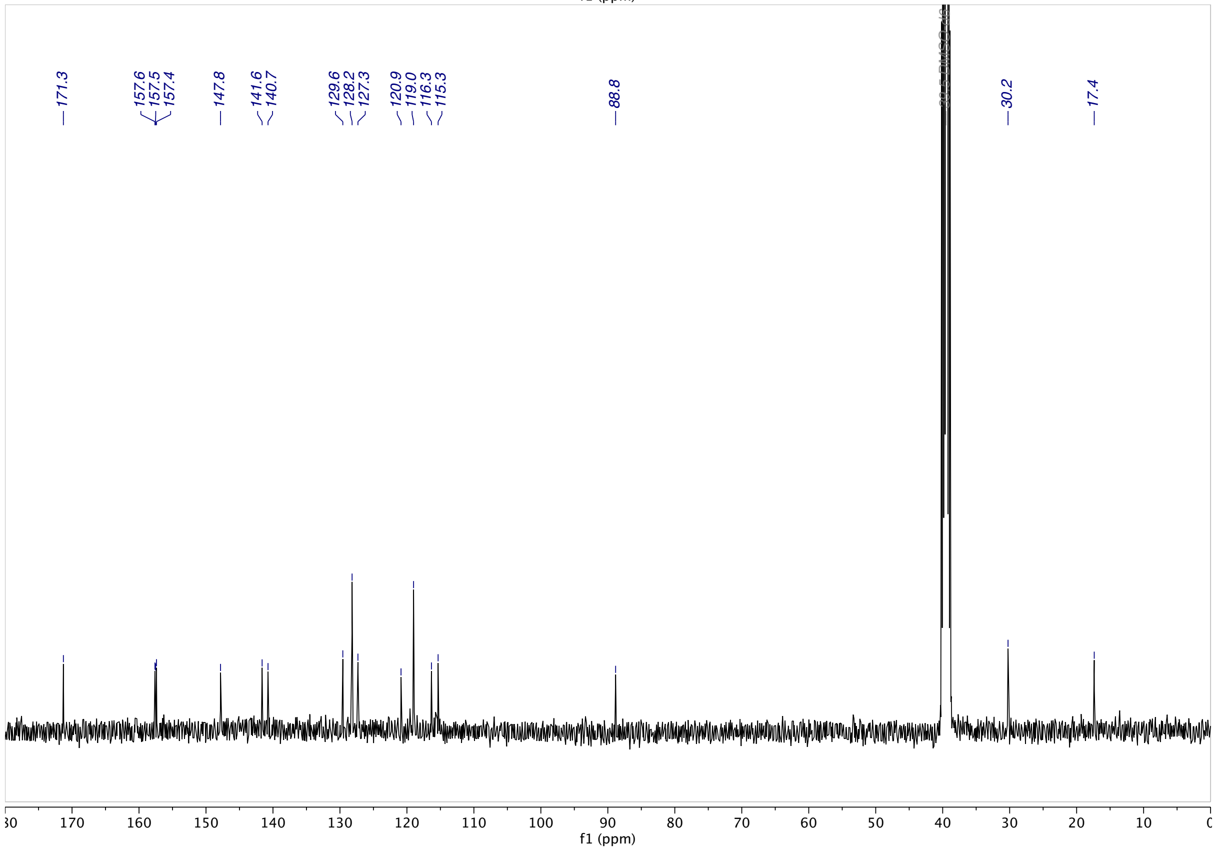

Figure S24. 4-((3-Aminophenyl)amino)-2-(phenylamino)-5,6-dihydropyrido[2,3-*d*]pyrimidin-7(8*H*)-one (19k)

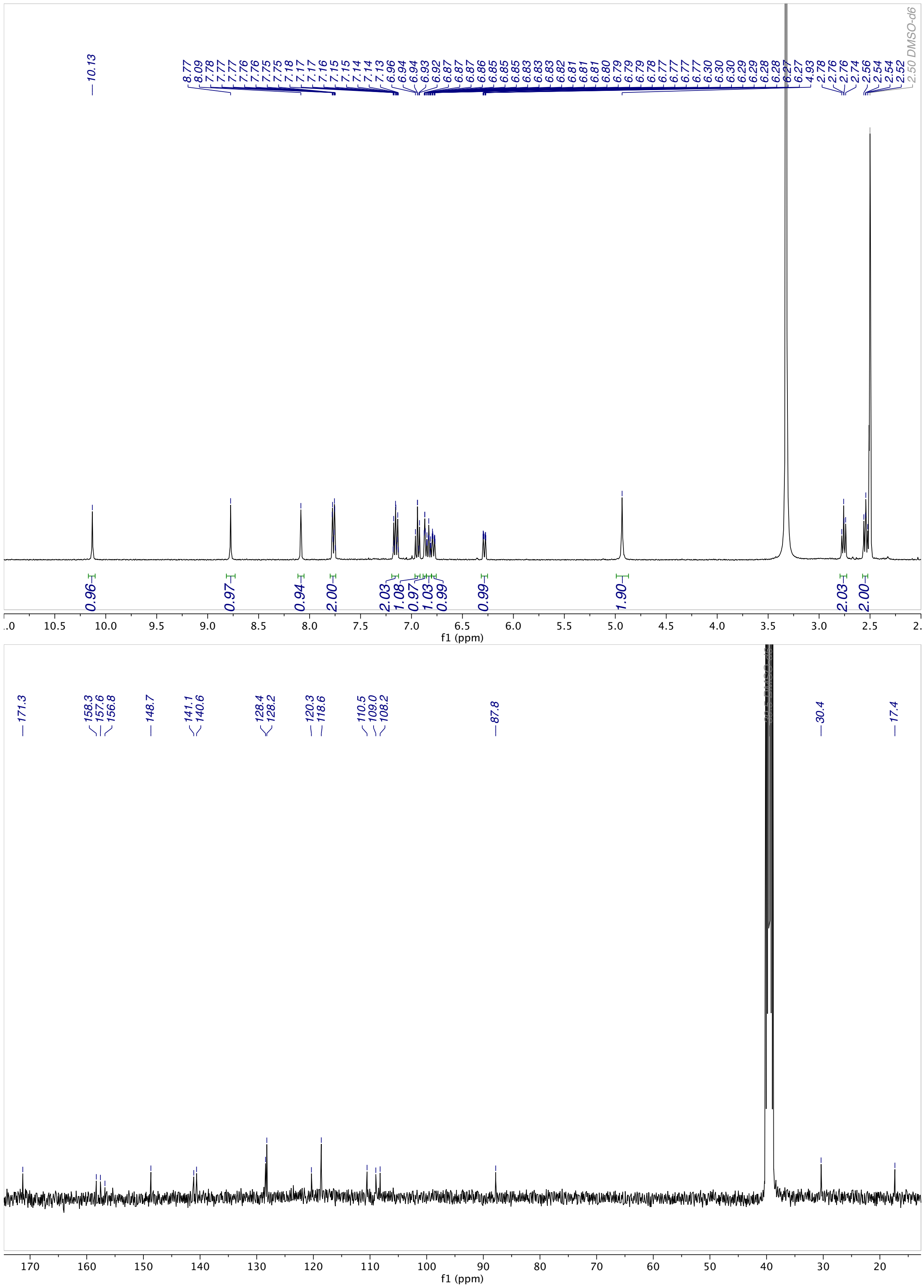

Figure S25. 4-Phenoxy-2-(phenylamino)-5,6-dihydropyrido[2,3-*d*]pyrimidin-7(8*H*)-one (20a)

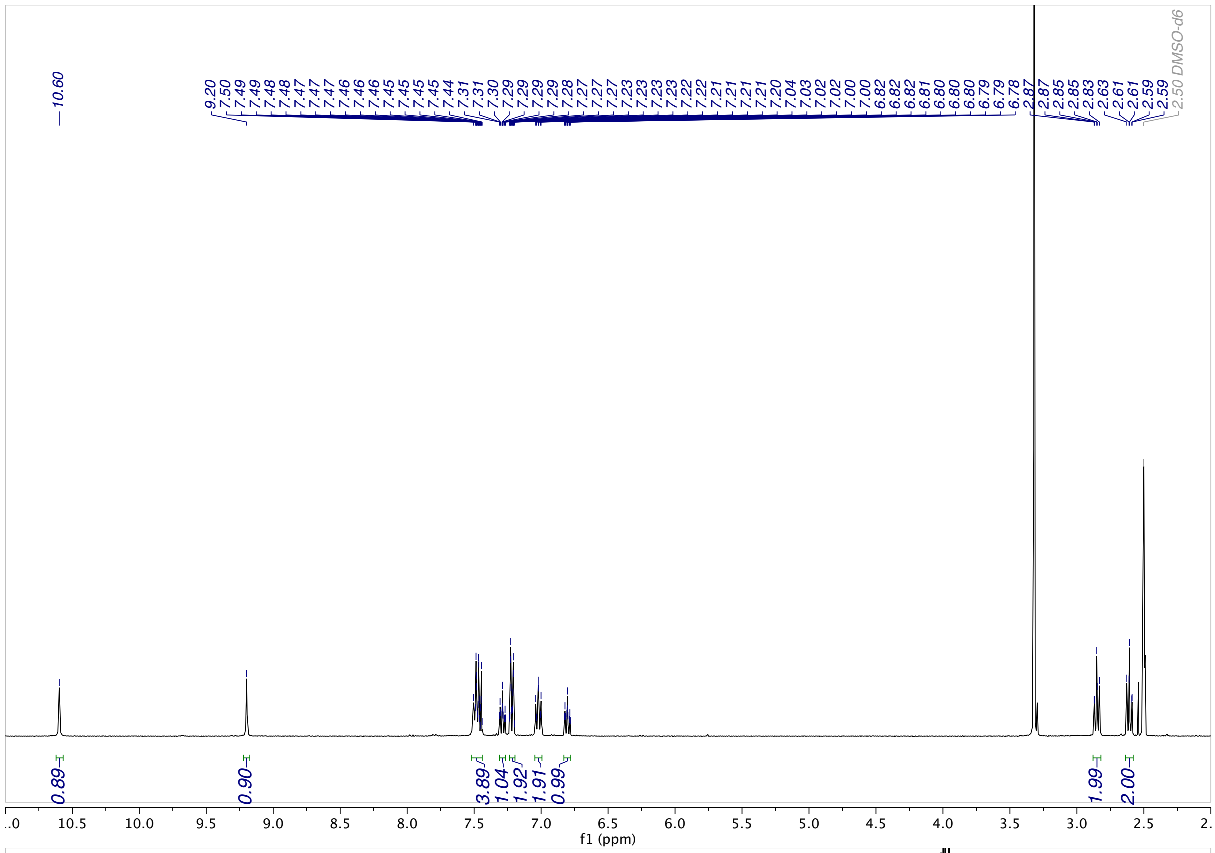

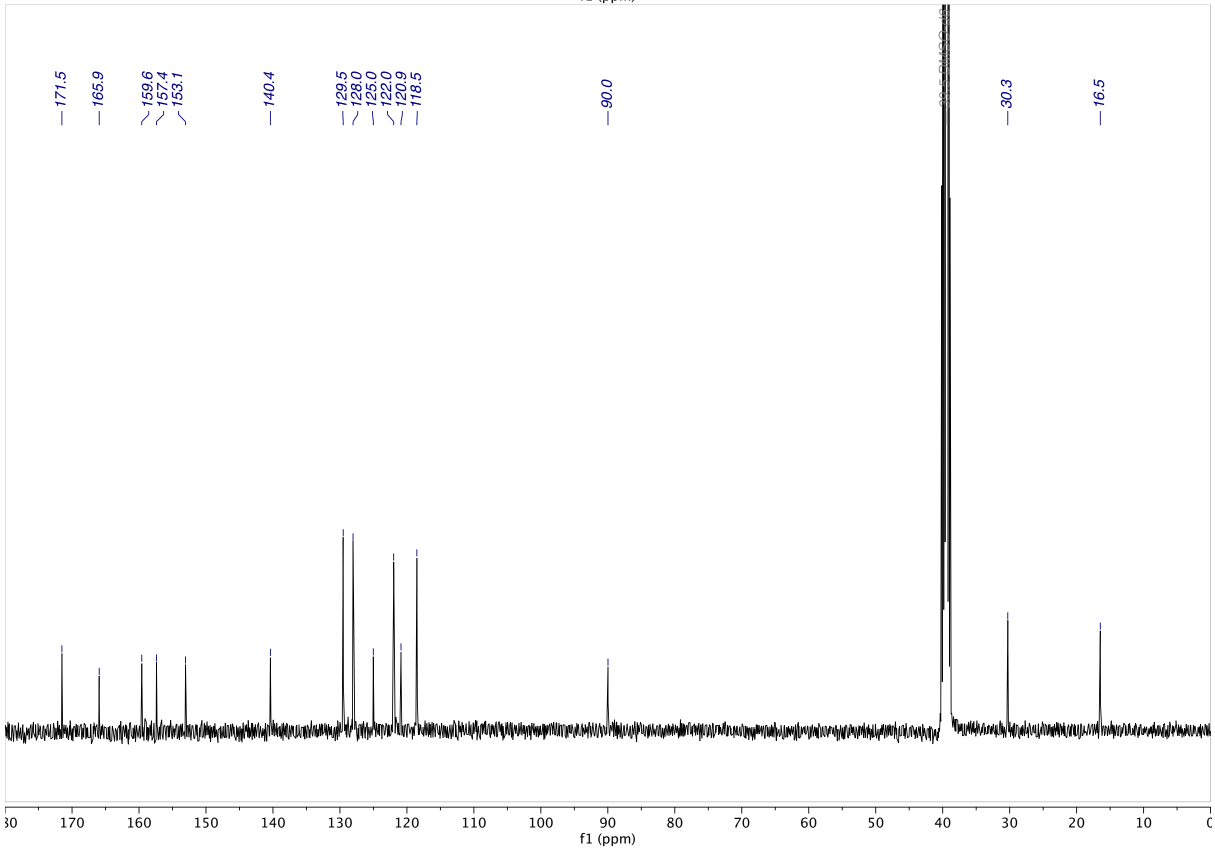

Figure S26. 2-(Phenylamino)-4-(59yridine-4-yloxy)-5,6-dihydropyrido[2,3-*d*]pyrimidin-7(8*H*)-one (20b)

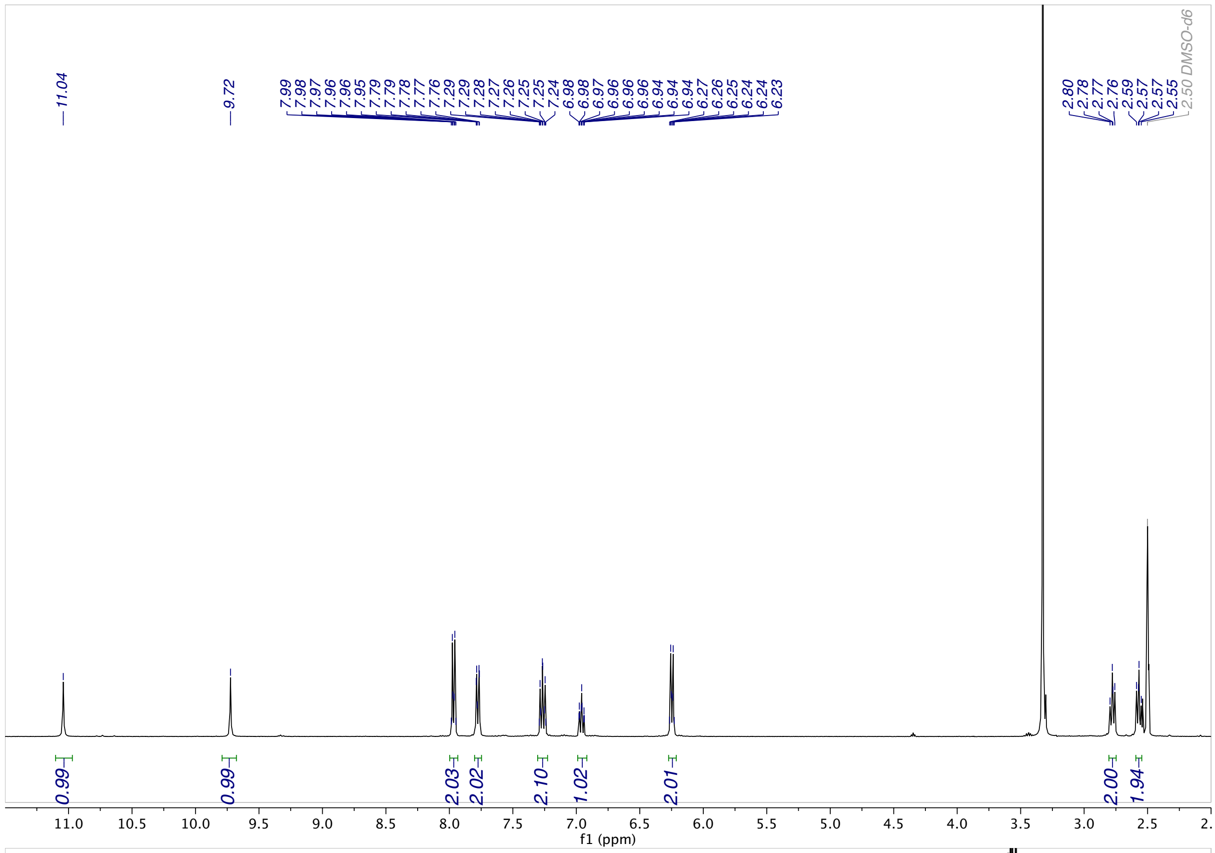

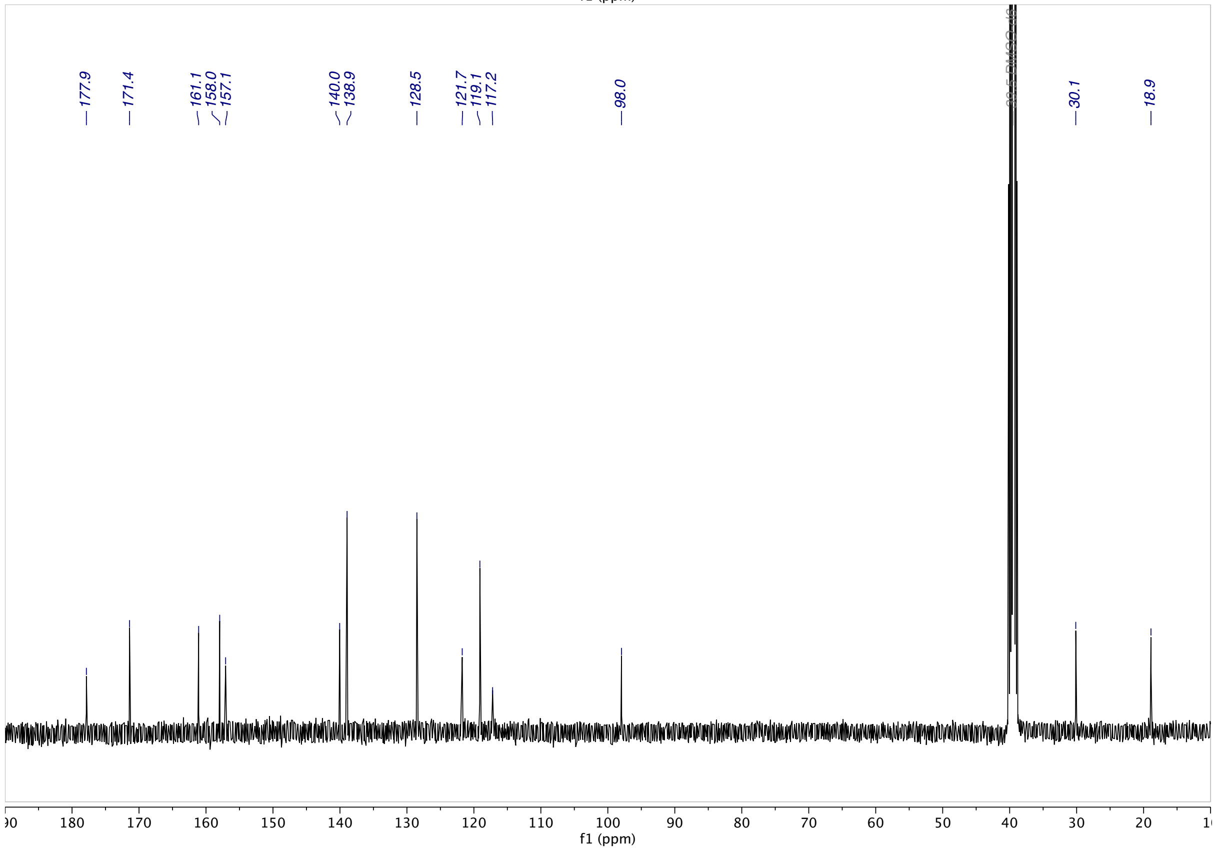

Figure S27. **4-((7-Oxo-2-(phenylamino)-5,6,7,8-tetrahydropyrido[2,3-*d*]pyrimidin-4-yl)oxy)benzonitrile (20c)**

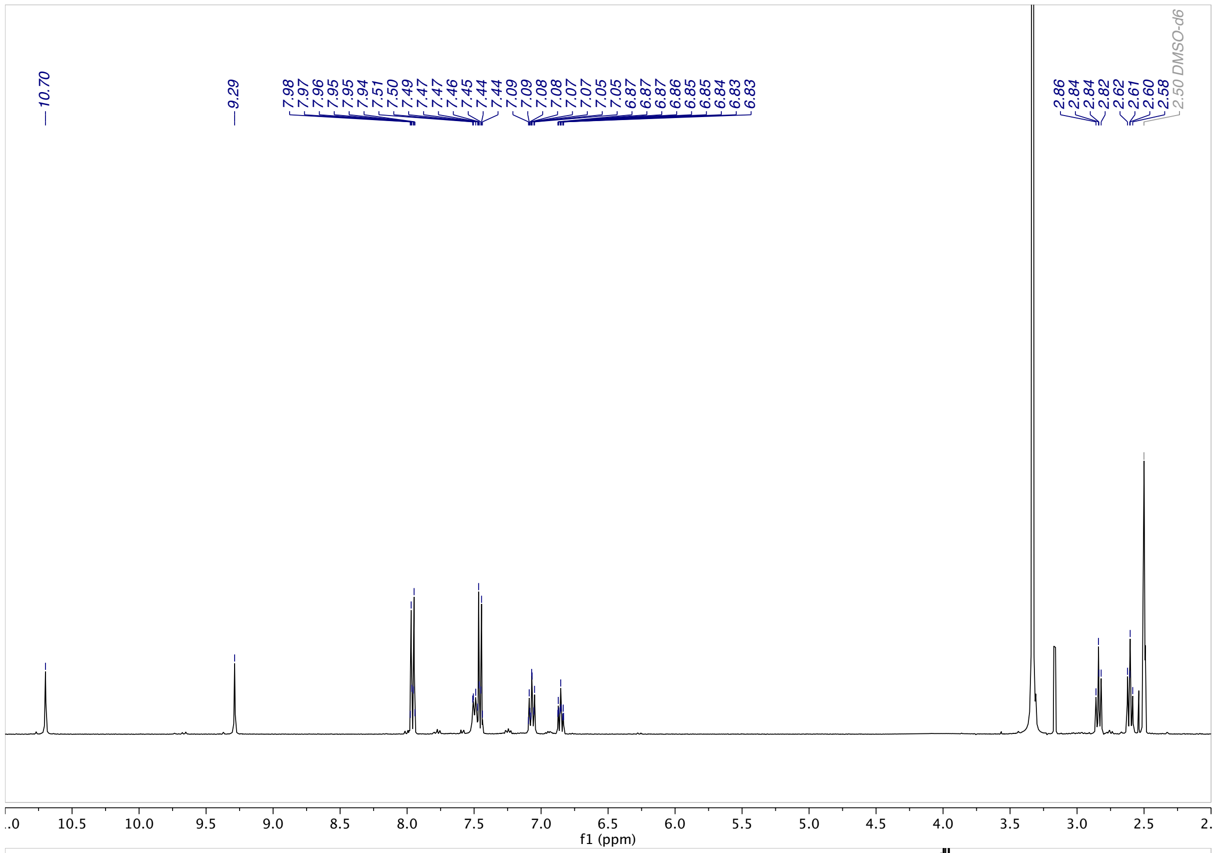

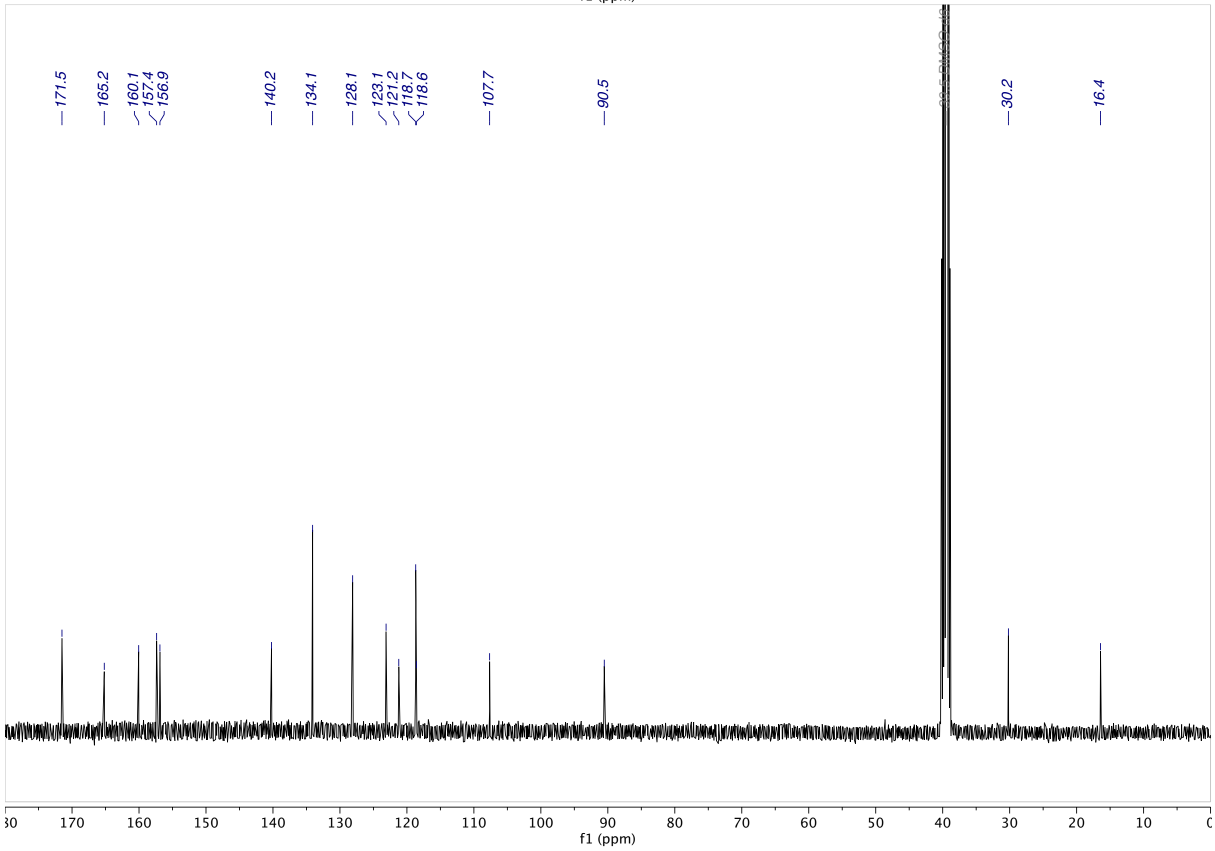

Figure S28. 4-(2-Fluorophenoxy)-2-(phenylamino)-5,6-dihydropyrido[2,3-*d*]pyrimidin-7(8*H*)-one (20d)

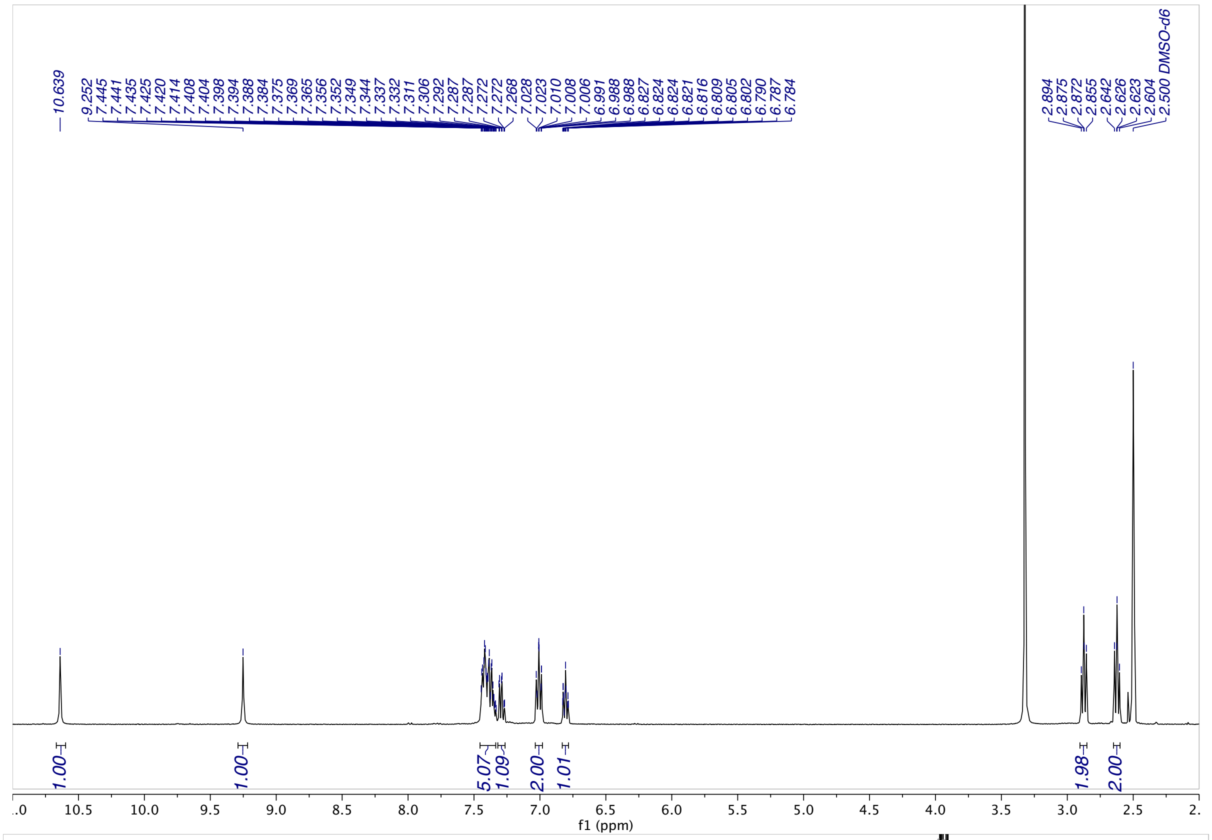

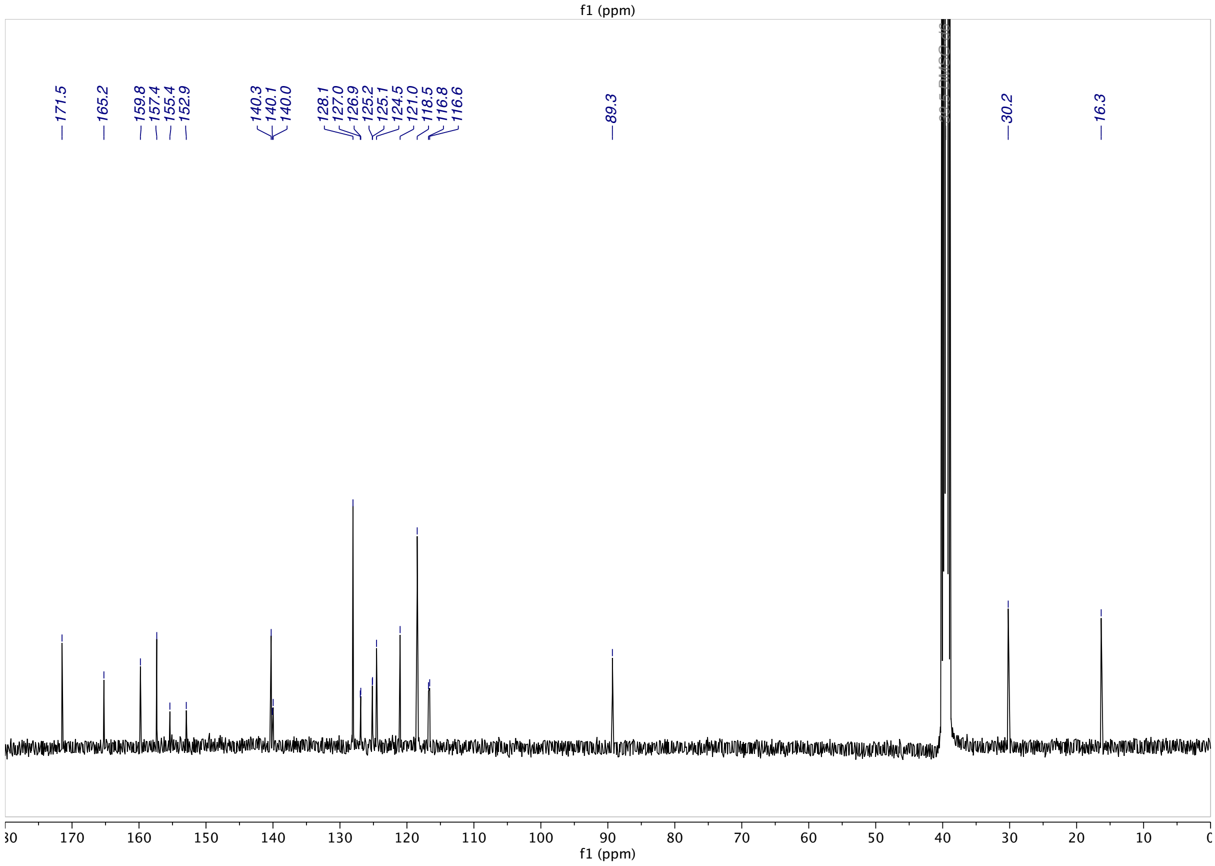

Figure S29. 4-(3,5-Dimethoxyphenoxy)-2-(phenylamino)-5,6-dihydropyrido[2,3-*d*]pyrimidin-7(8*H*)-one (20e)

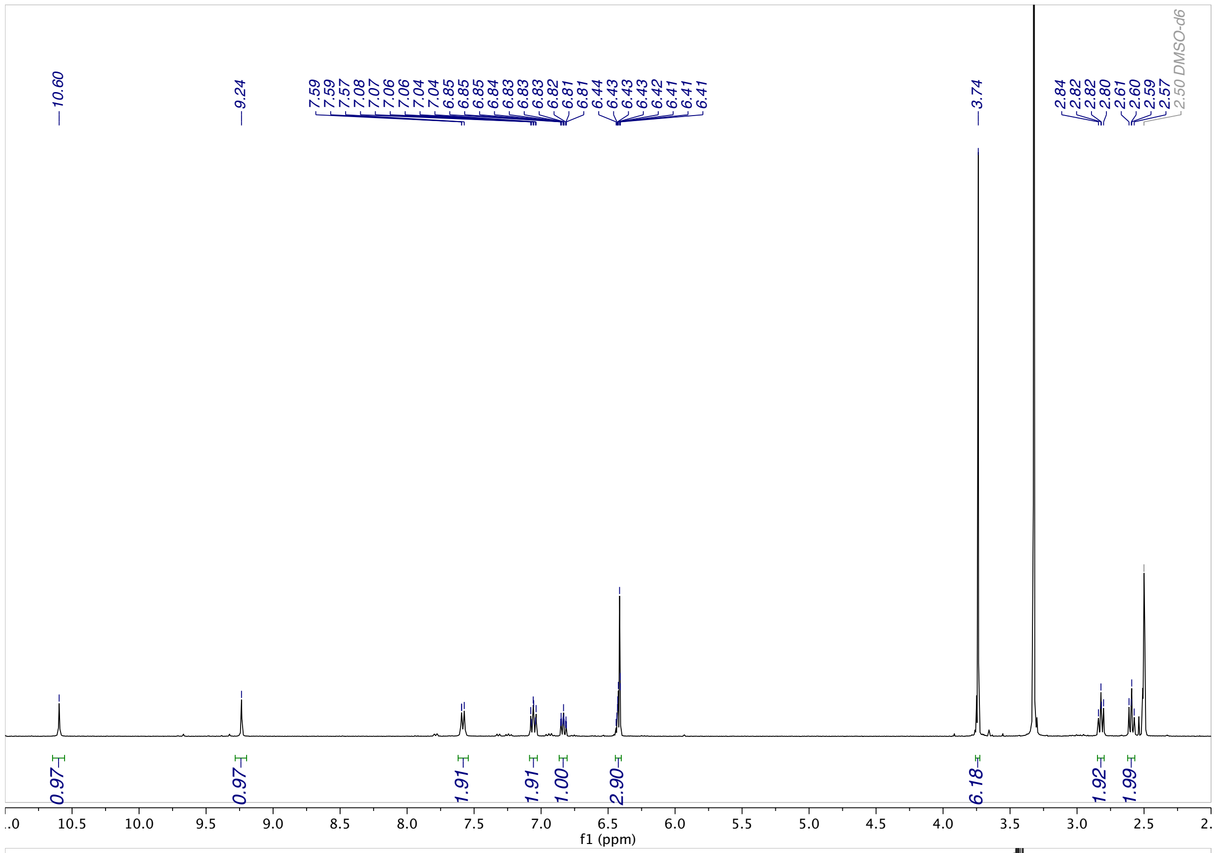

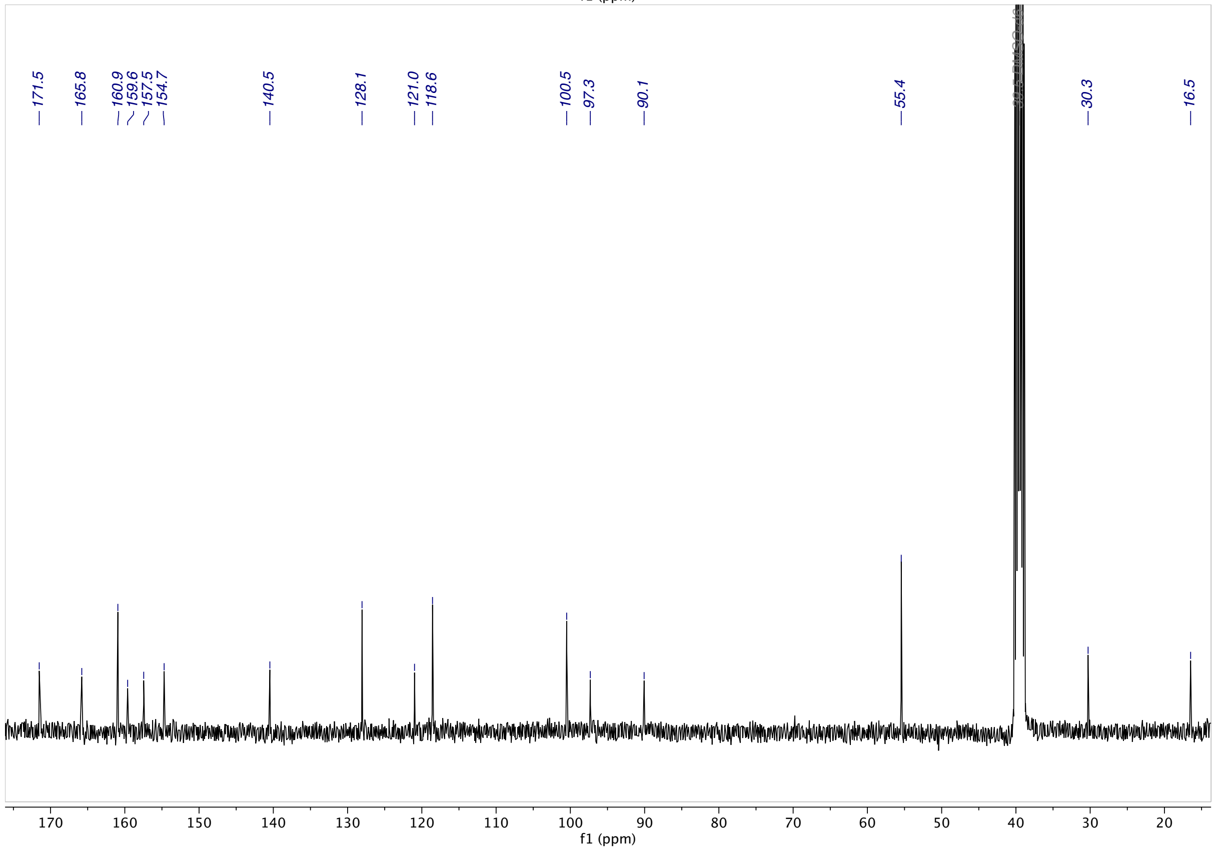

Figure S30. 2-(Phenylamino)-4-(3-(trifluoromethyl)phenoxy)-5,6-dihydropyrido[2,3-*d*]pyrimidin-7(8*H*)-one (20f)

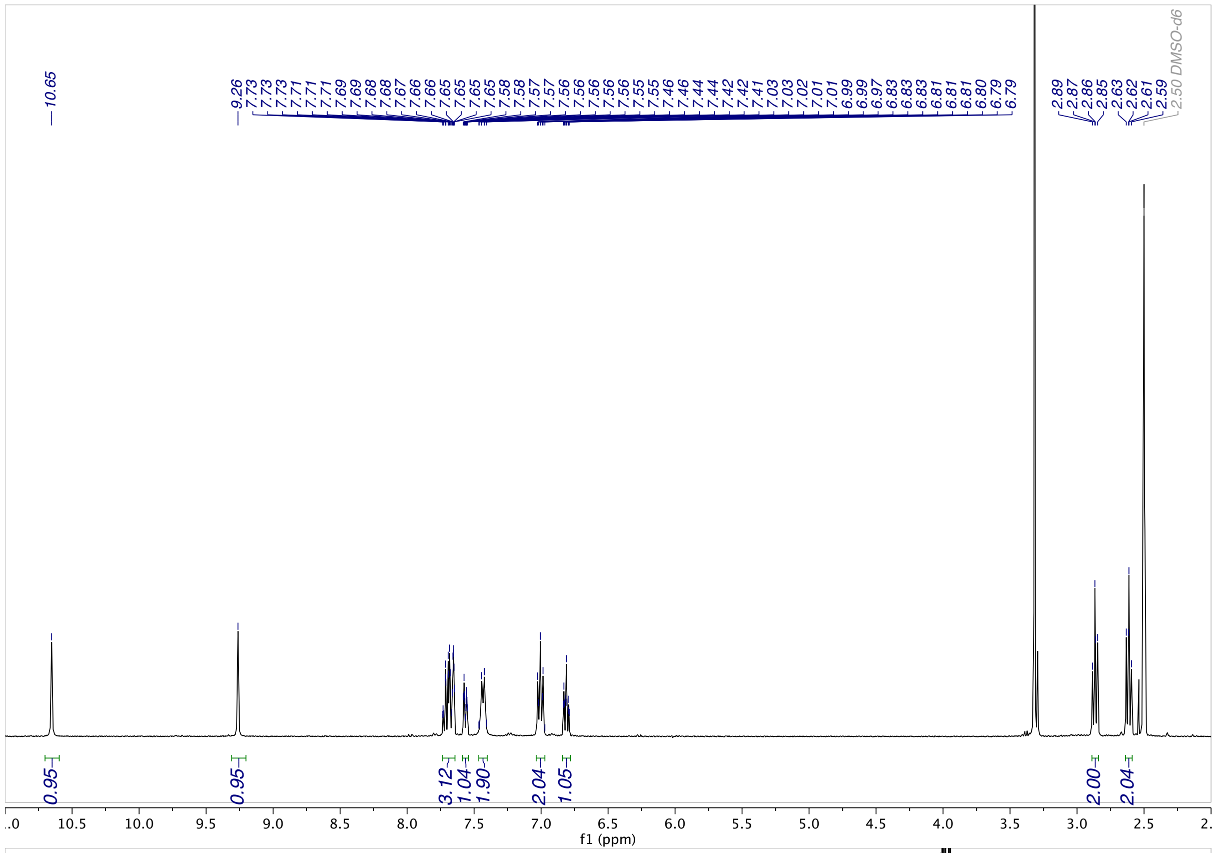

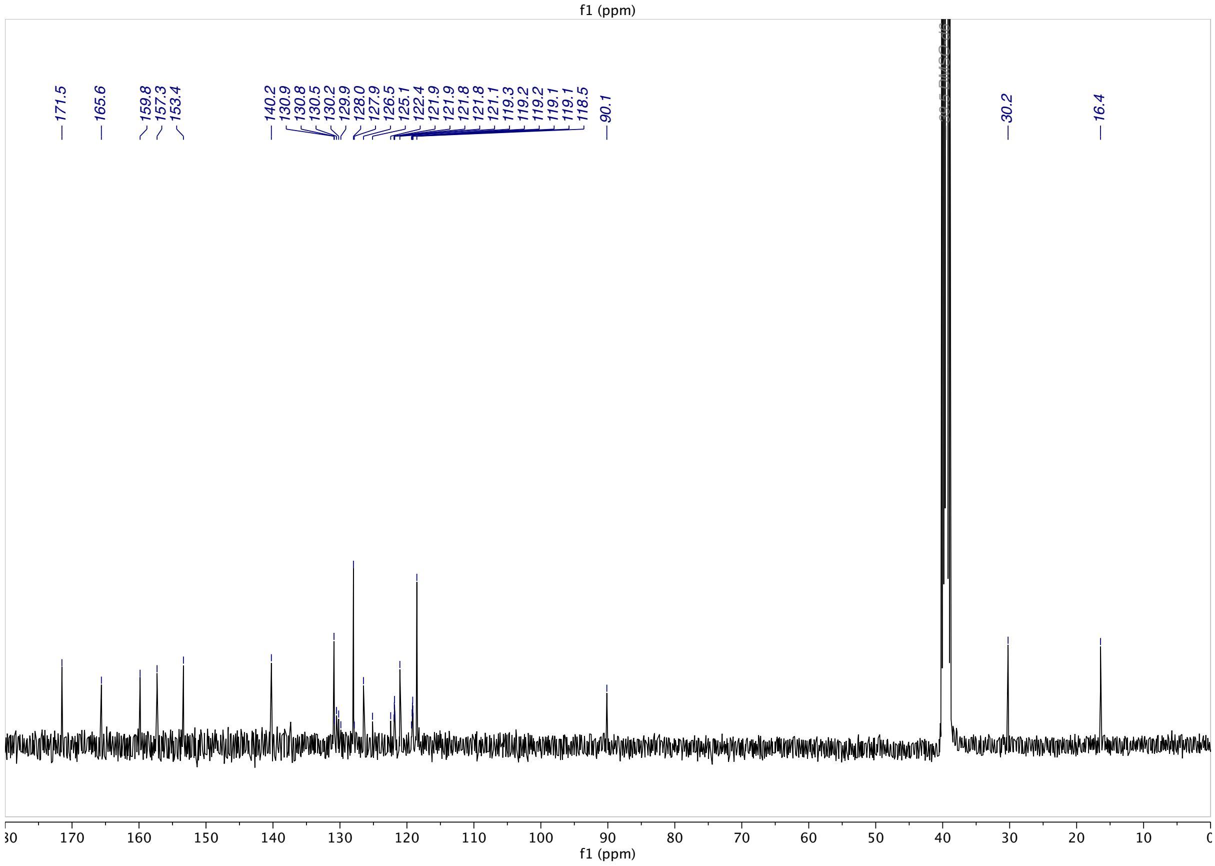

Figure S31. 4-(4-Bromo-2-fluorophenoxy)-2-(phenylamino)-5,6-dihydropyrido[2,3-*d*]pyrimidin-7(8*H*)-one (20g)

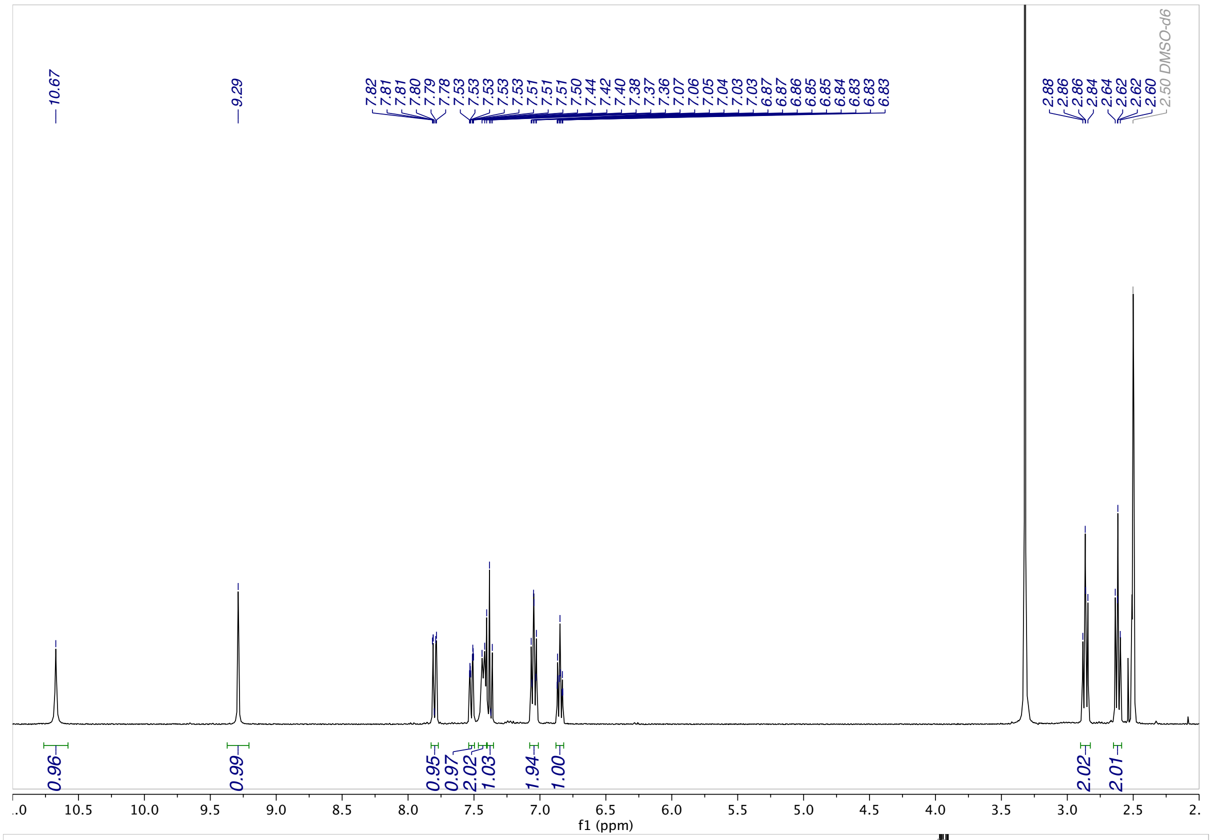

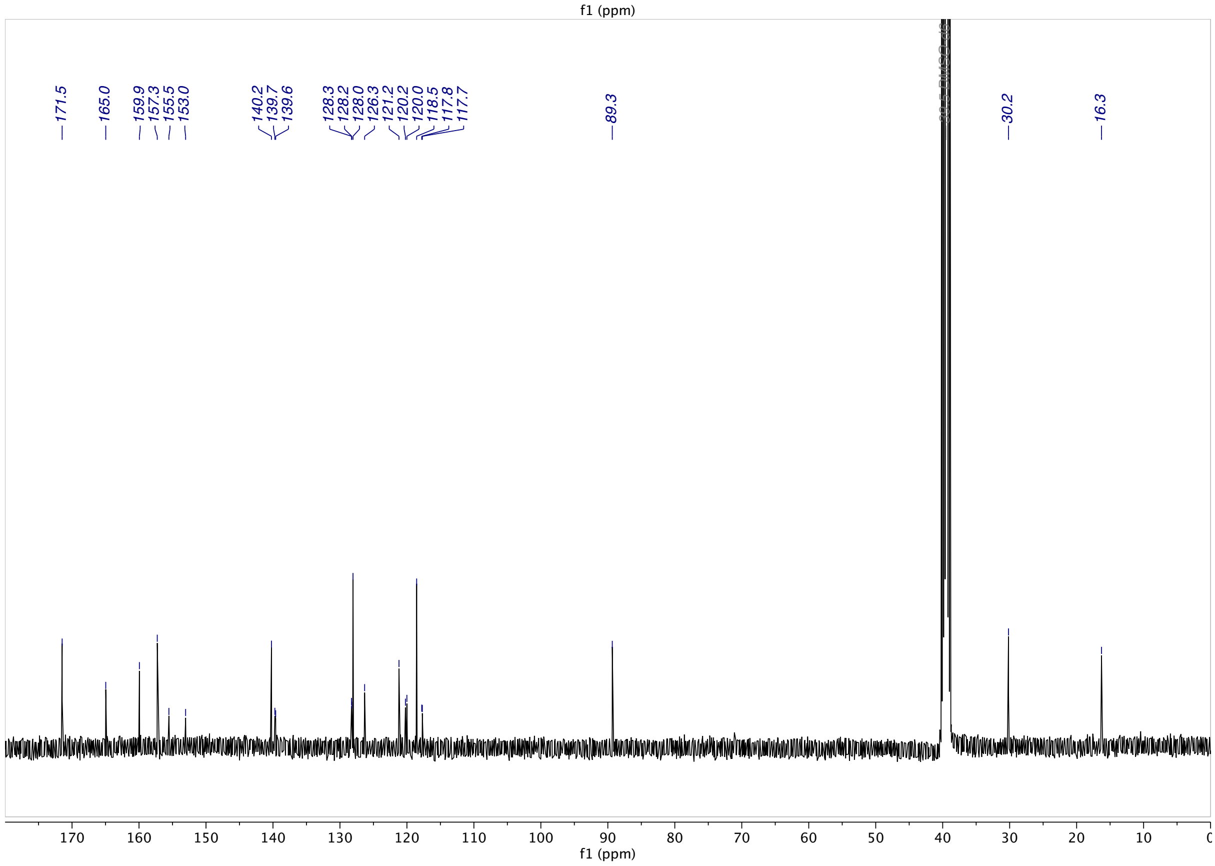

Figure S32. 4-(3-Nitrophenoxy)-2-(phenylamino)-5,6-dihydropyrido[2,3-*d*]pyrimidin-7(8*H*)-one (20h)

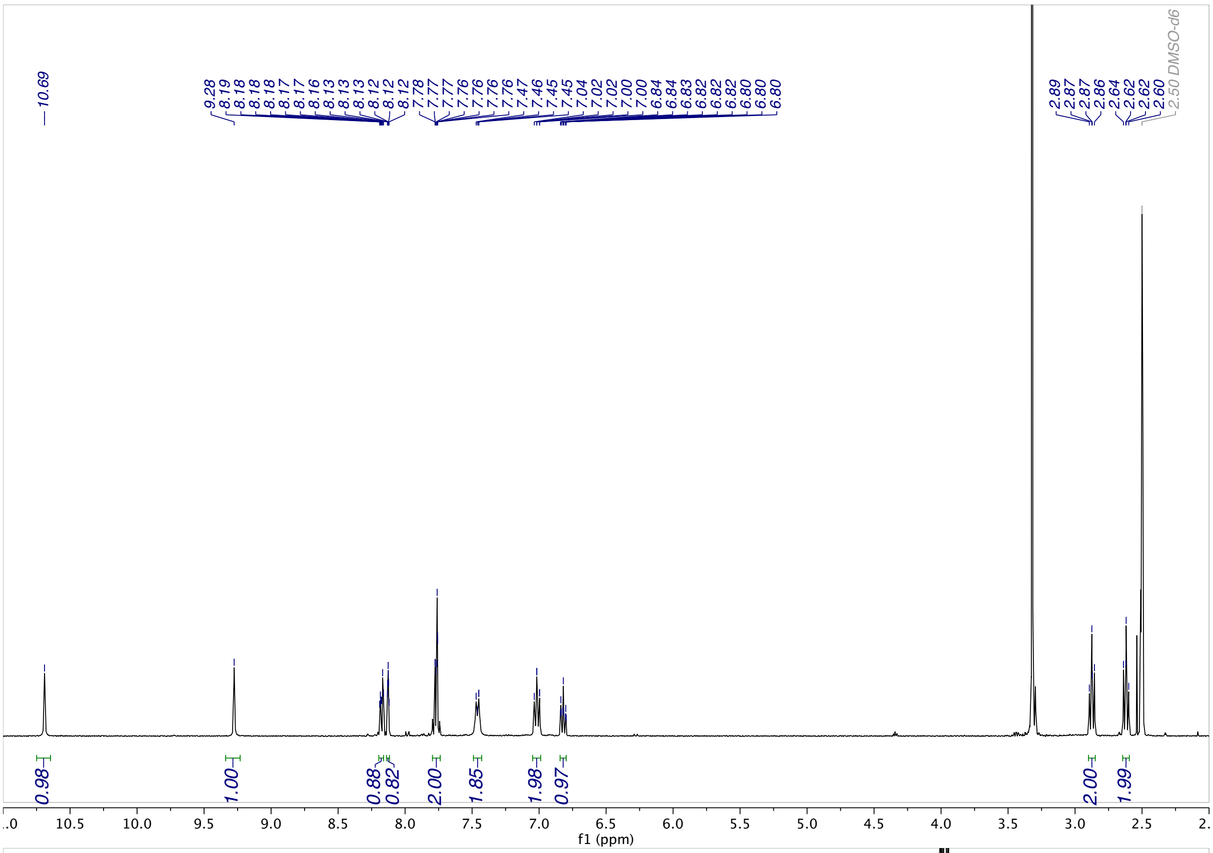

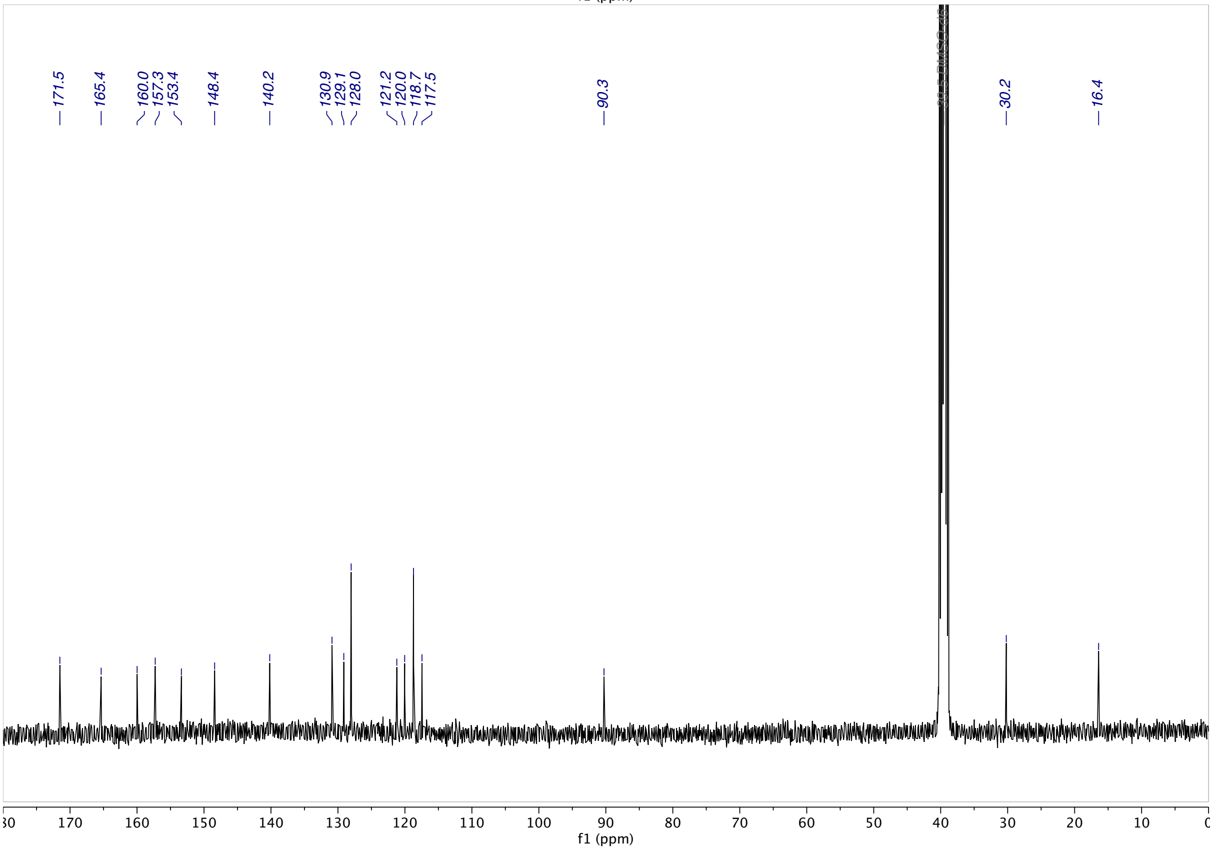

Figure S33. 4-(3-Aminophenoxy)-2-(phenylamino)-5,6-dihydropyrido[2,3-*d*]pyrimidin-7(8*H*)-one (20i)

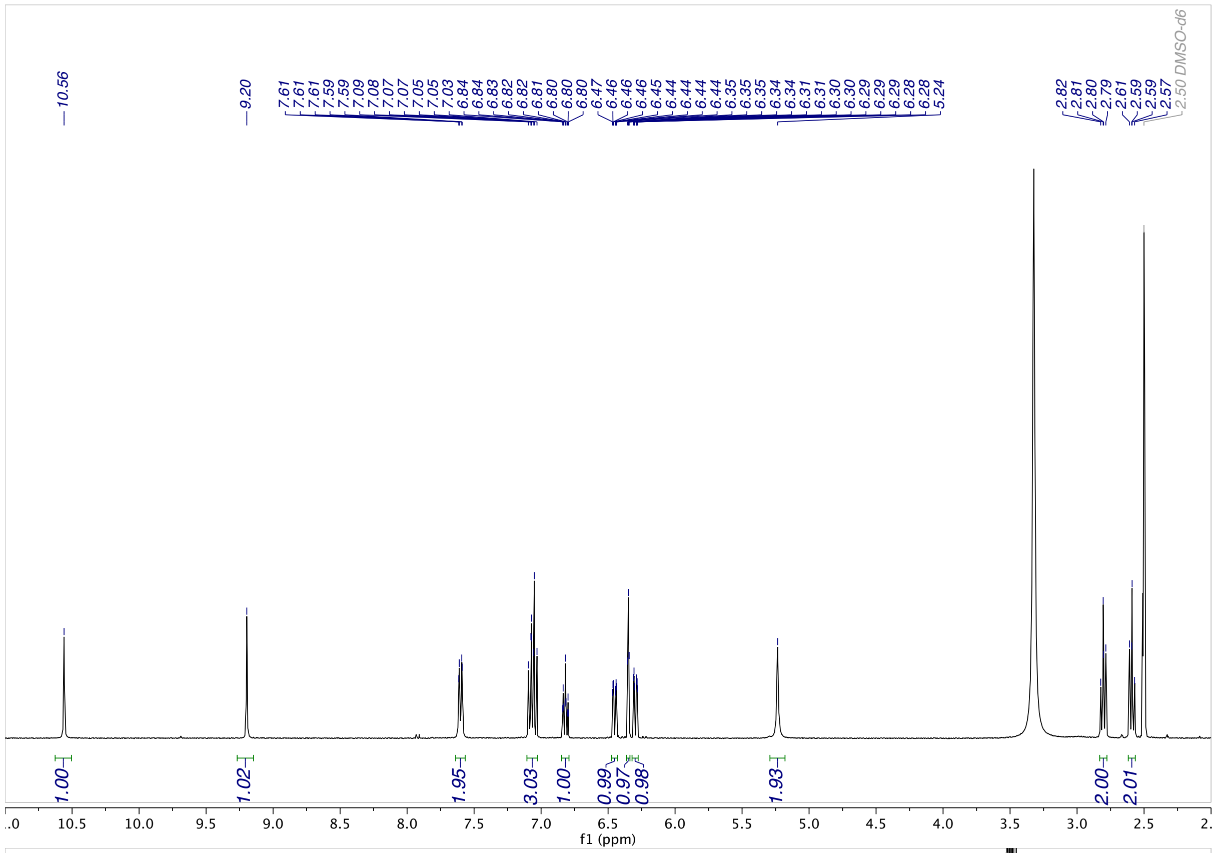

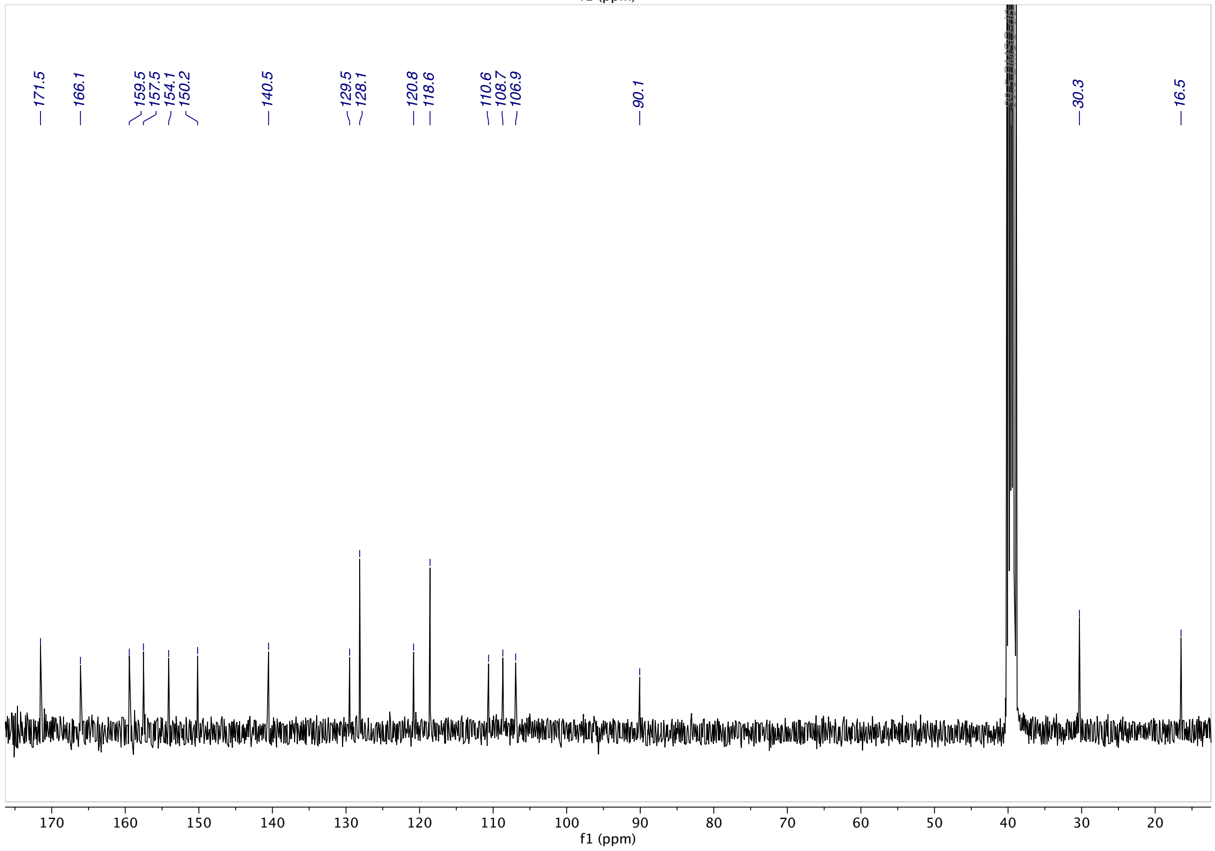

Figure S34. ***N*-(3-((7-oxo-2-(phenylamino)-5,6,7,8-tetrahydropyrido[2,3-*d*]pyrimidin-4-yl)oxy)phenyl)acrylamide (20j)**

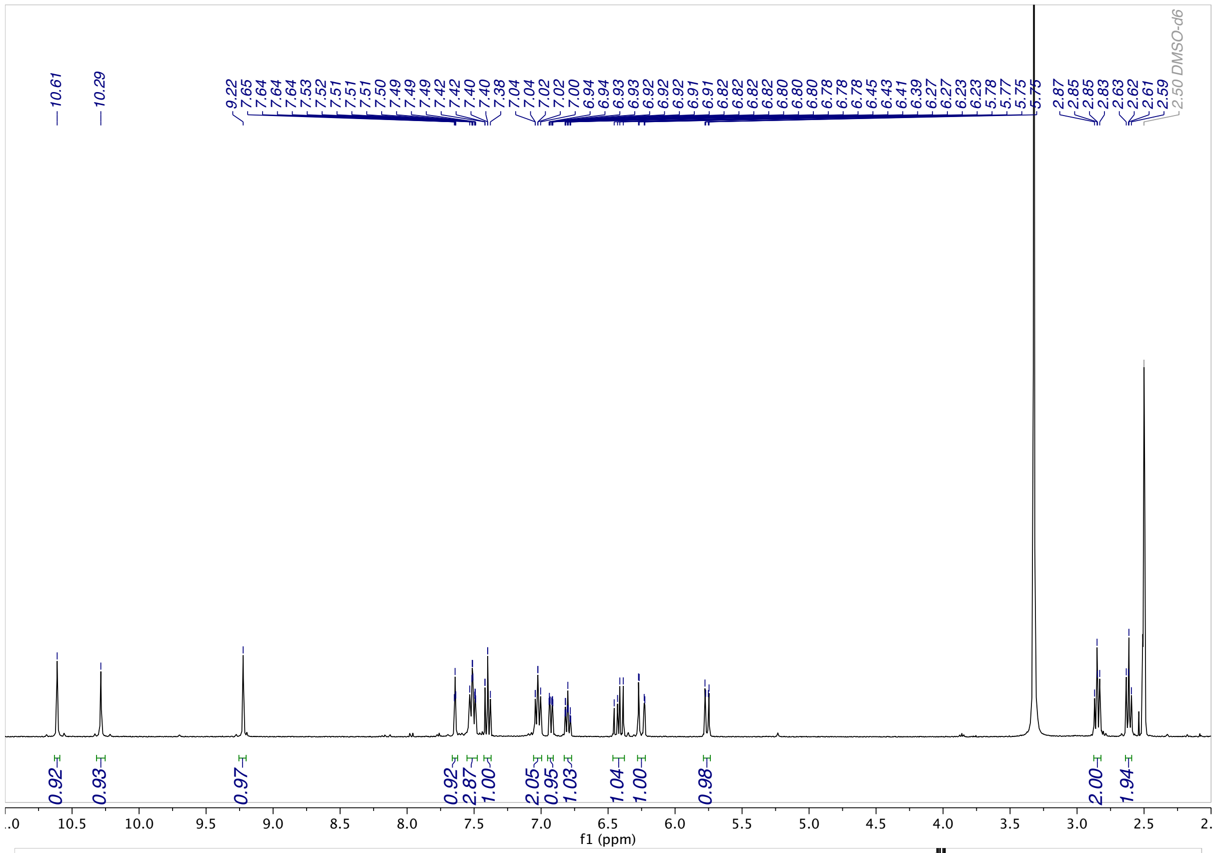

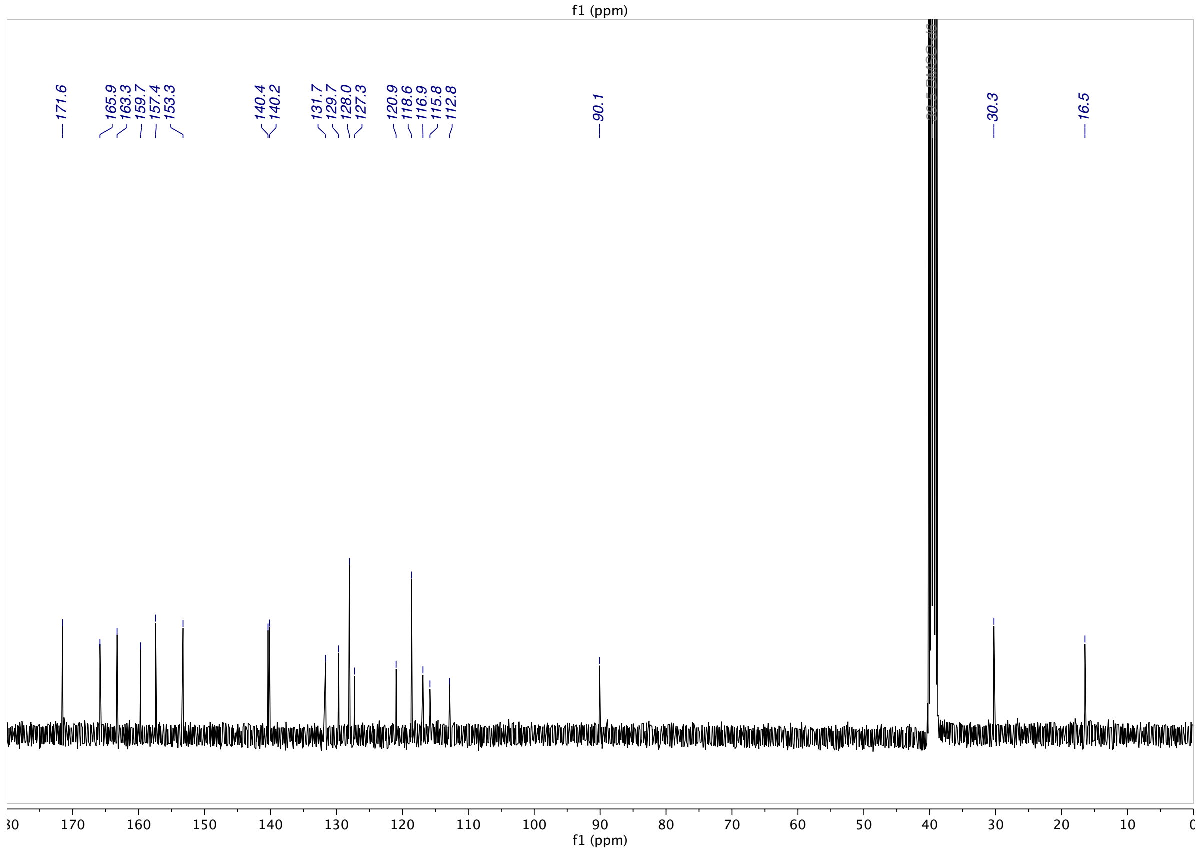

Figure S35. 4-((4-Nitrophenyl)thio)-2-(phenylamino)-5,6-dihydropyrido[2,3-*d*]pyrimidin-7(8*H*)-one (21a)

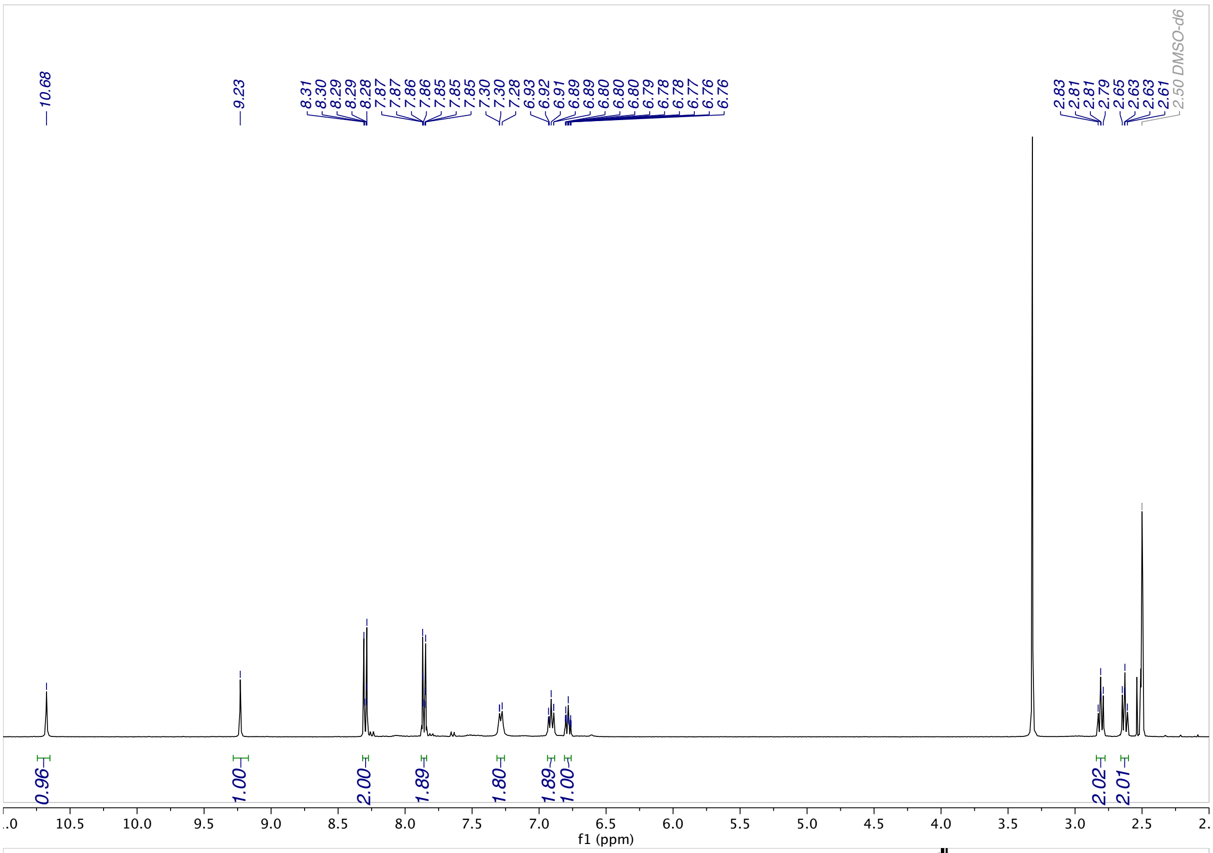

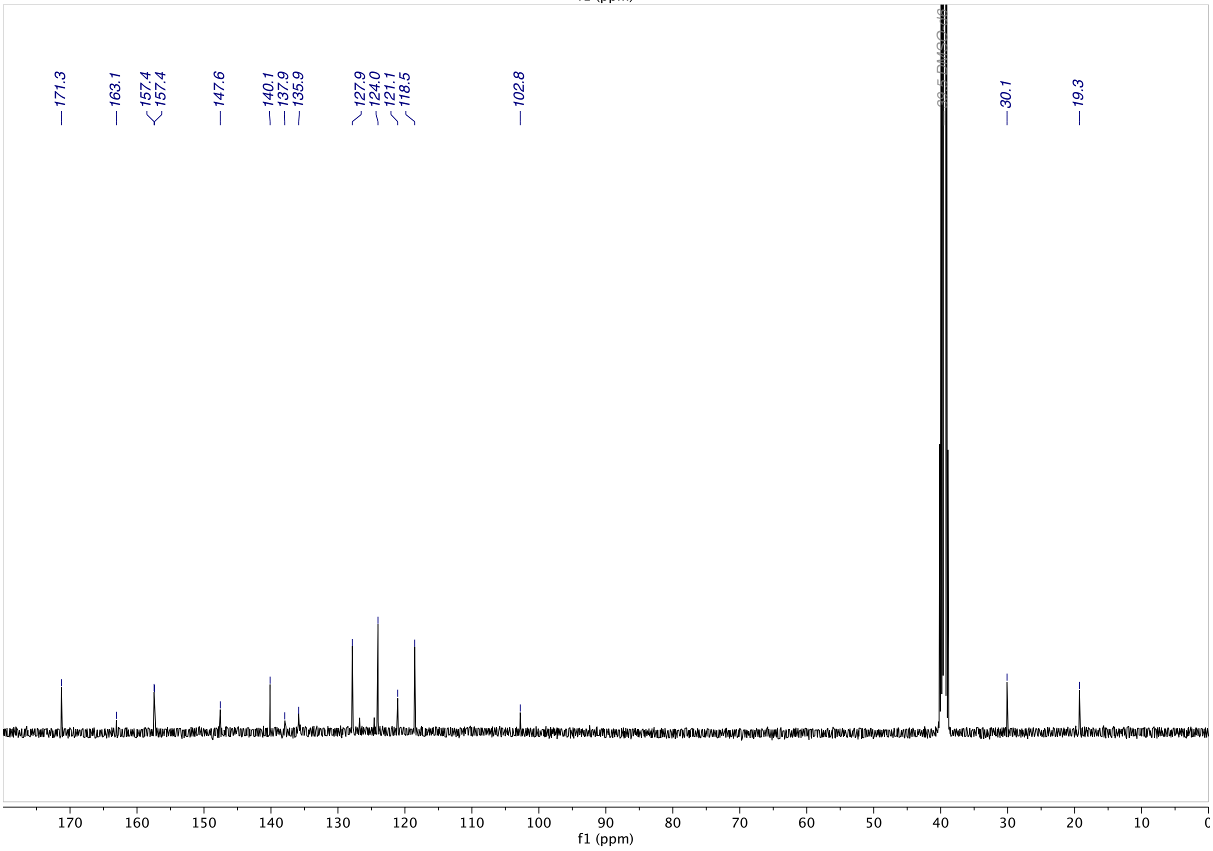

Figure S36. 4-((4-Methoxyphenyl)thio)-2-(phenylamino)-5,6-dihydropyrido[2,3-*d*]pyrimidin-7(8*H*)-one (21b)

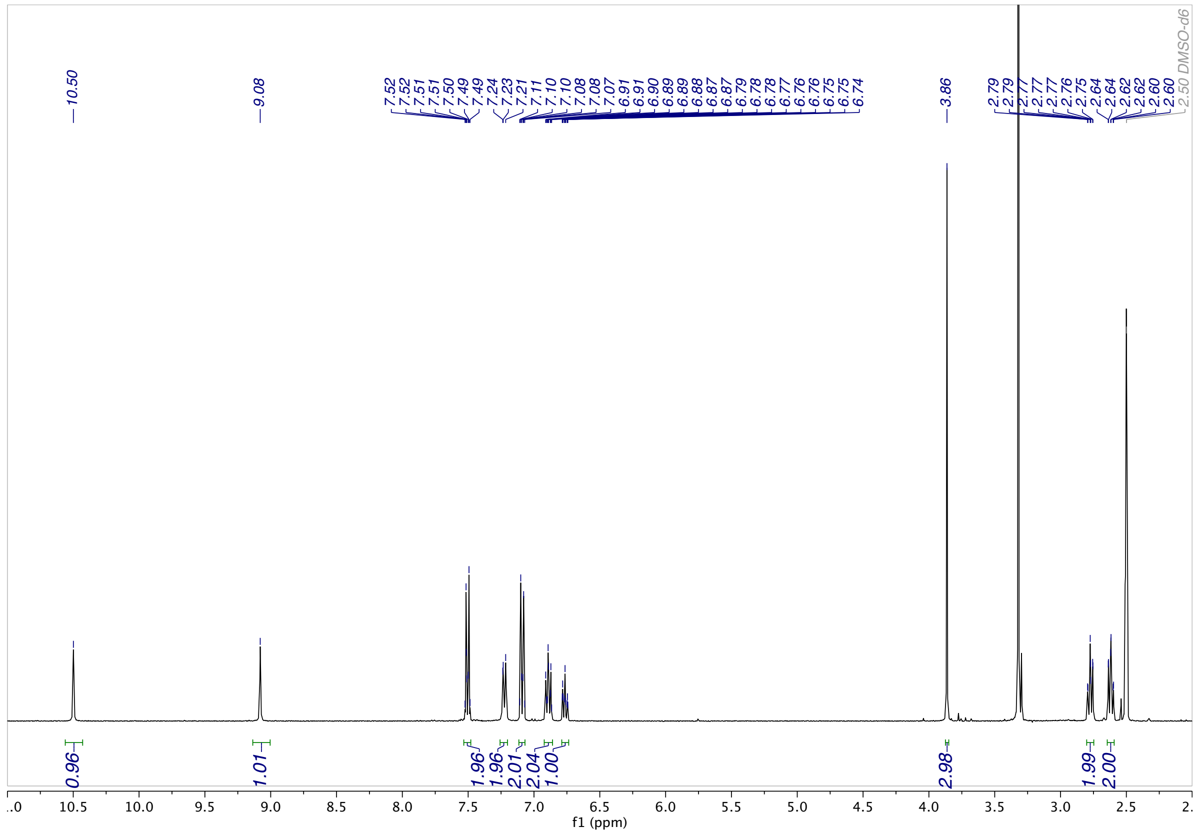

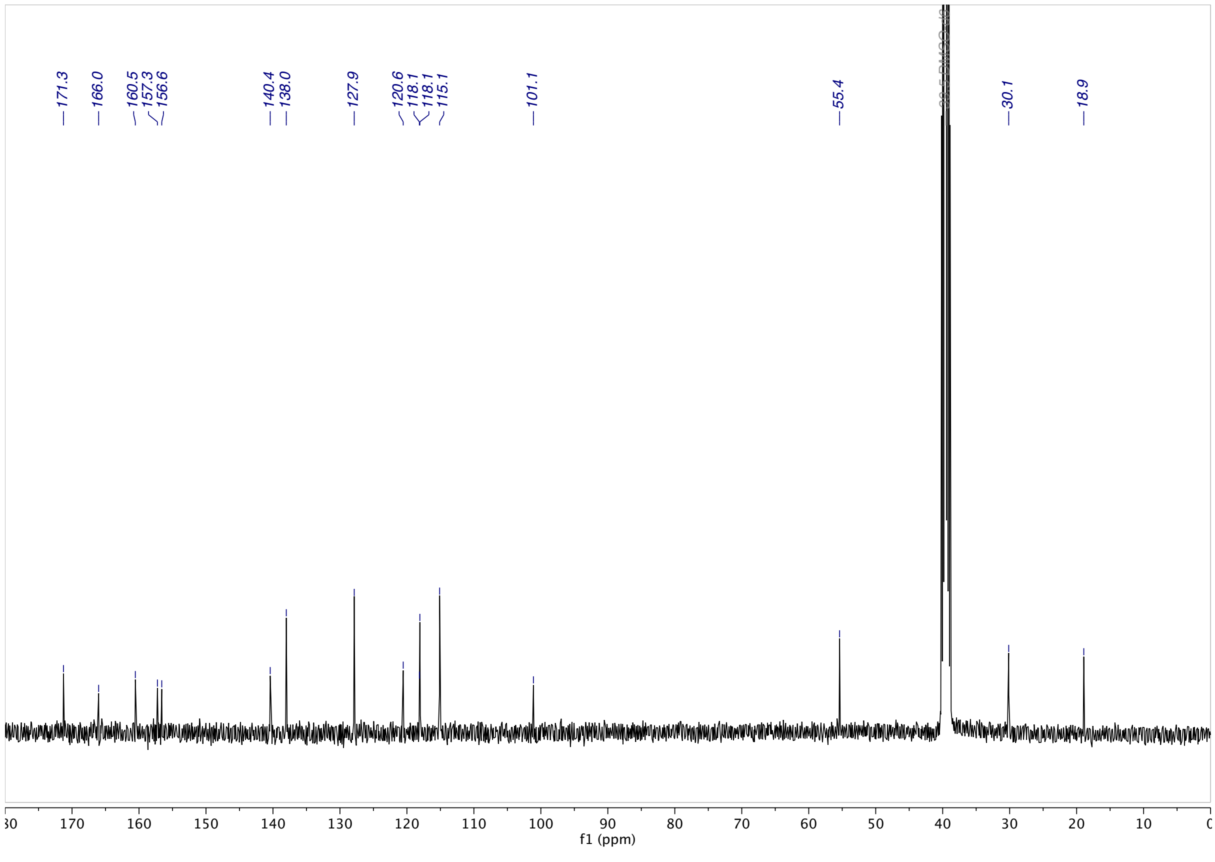

Figure S37. 4-Phenyl-2-(phenylamino)-5,6-dihydropyrido[2,3-*d*]pyrimidin-7(8*H*)-one (22a)

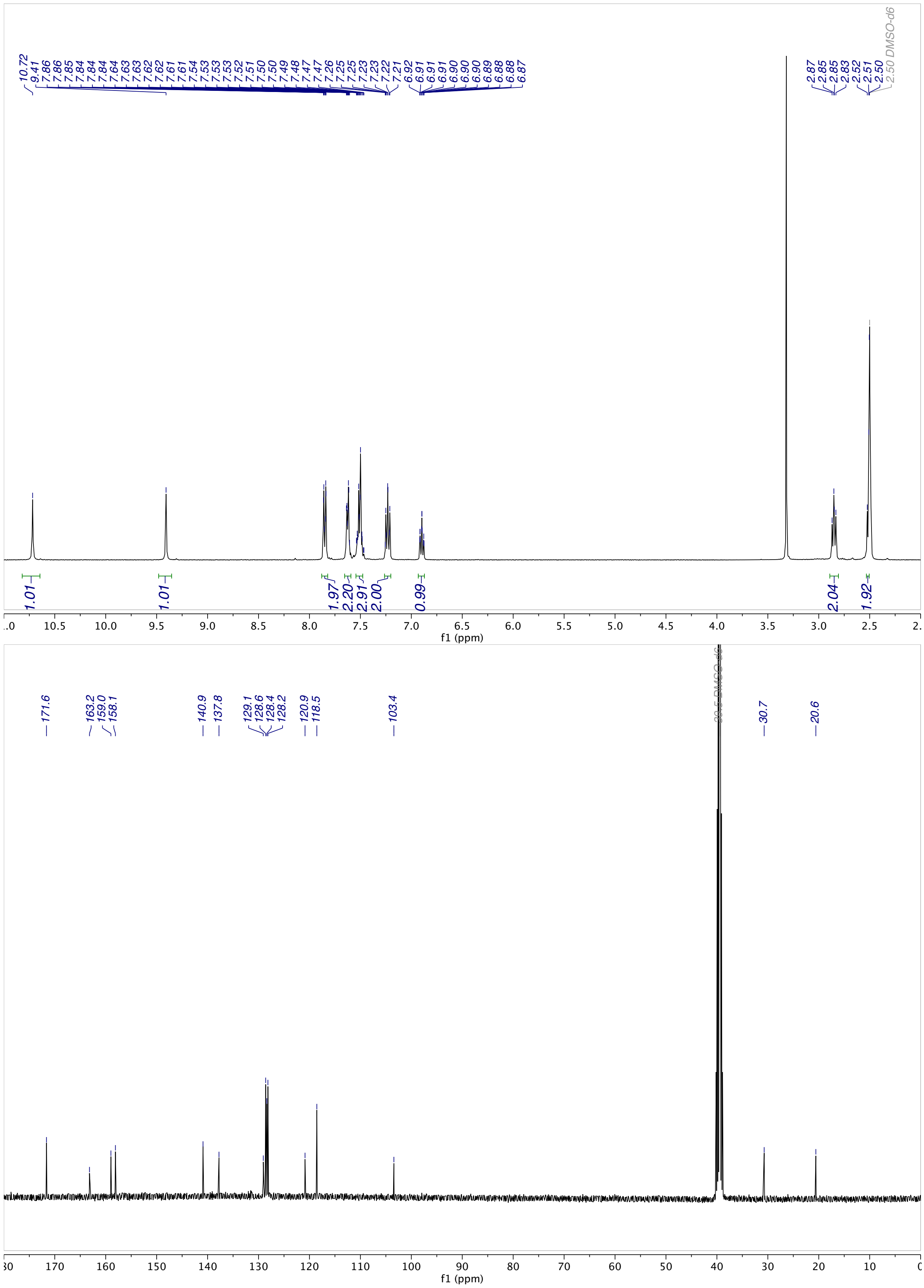

Figure S38. 2-(Phenylamino)-4-(p-tolyl)-5,6-dihydropyrido[2,3-*d*]pyrimidin-7(8*H*)-one (22b)

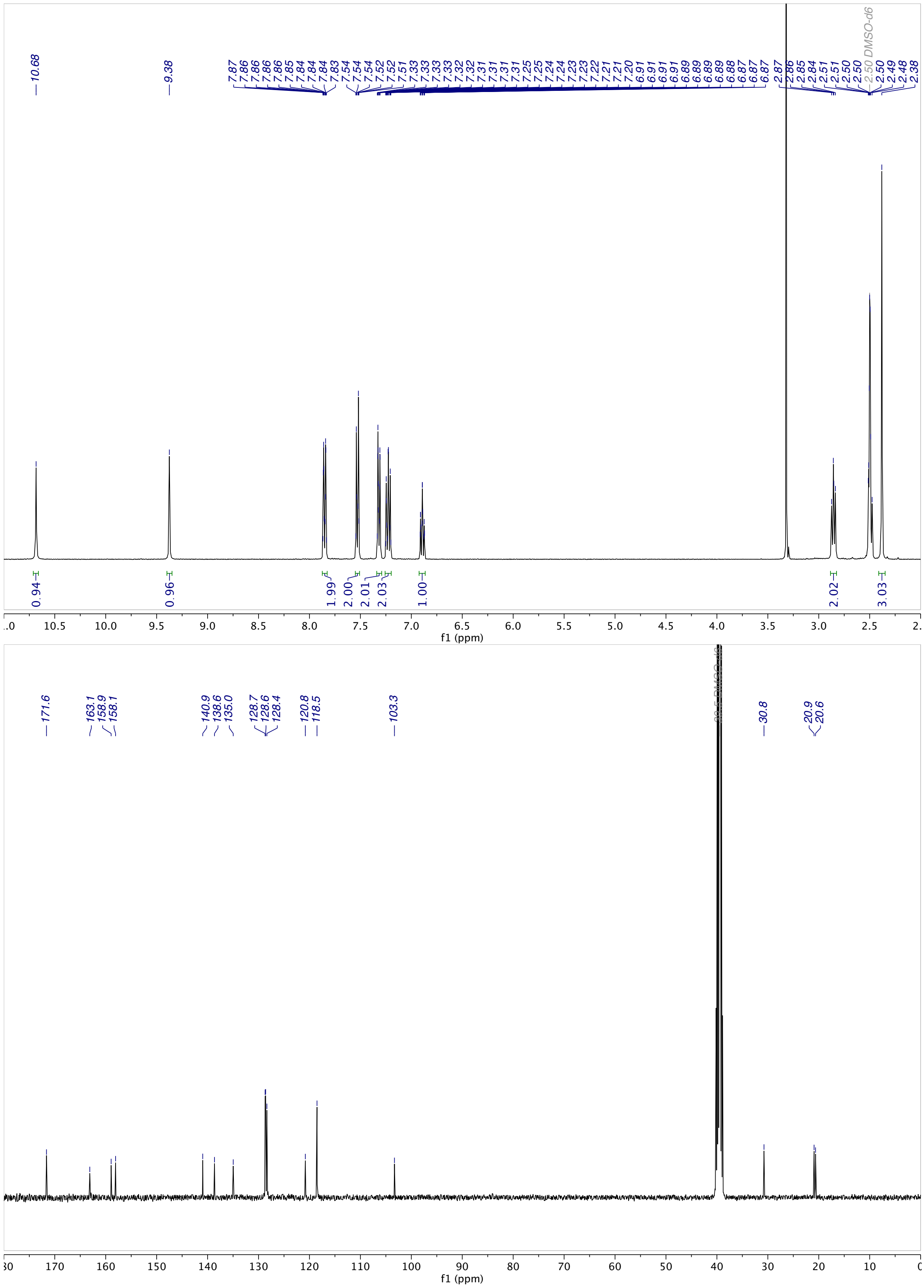

Figure S39. 4-(4-Methoxyphenyl)-2-(phenylamino)-5,6-dihydropyrido[2,3-*d*]pyrimidin-7(8*H*)-one (22c)

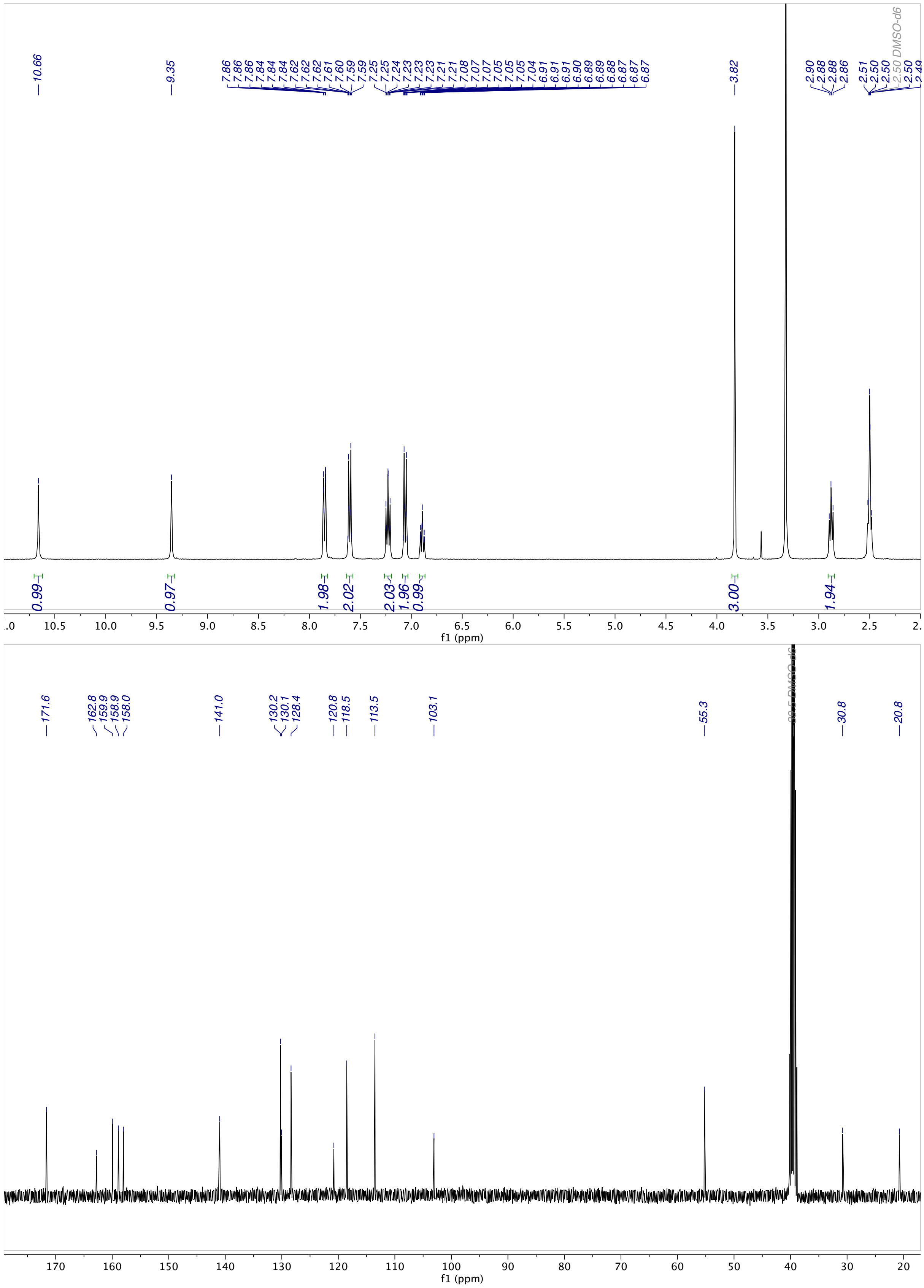

Figure S40. 2-(Phenylamino)-4-(3,4,5-trimethoxyphenyl)-5,6-dihydropyrido[2,3-*d*]pyrimidin-7(8*H*)-one (22d)

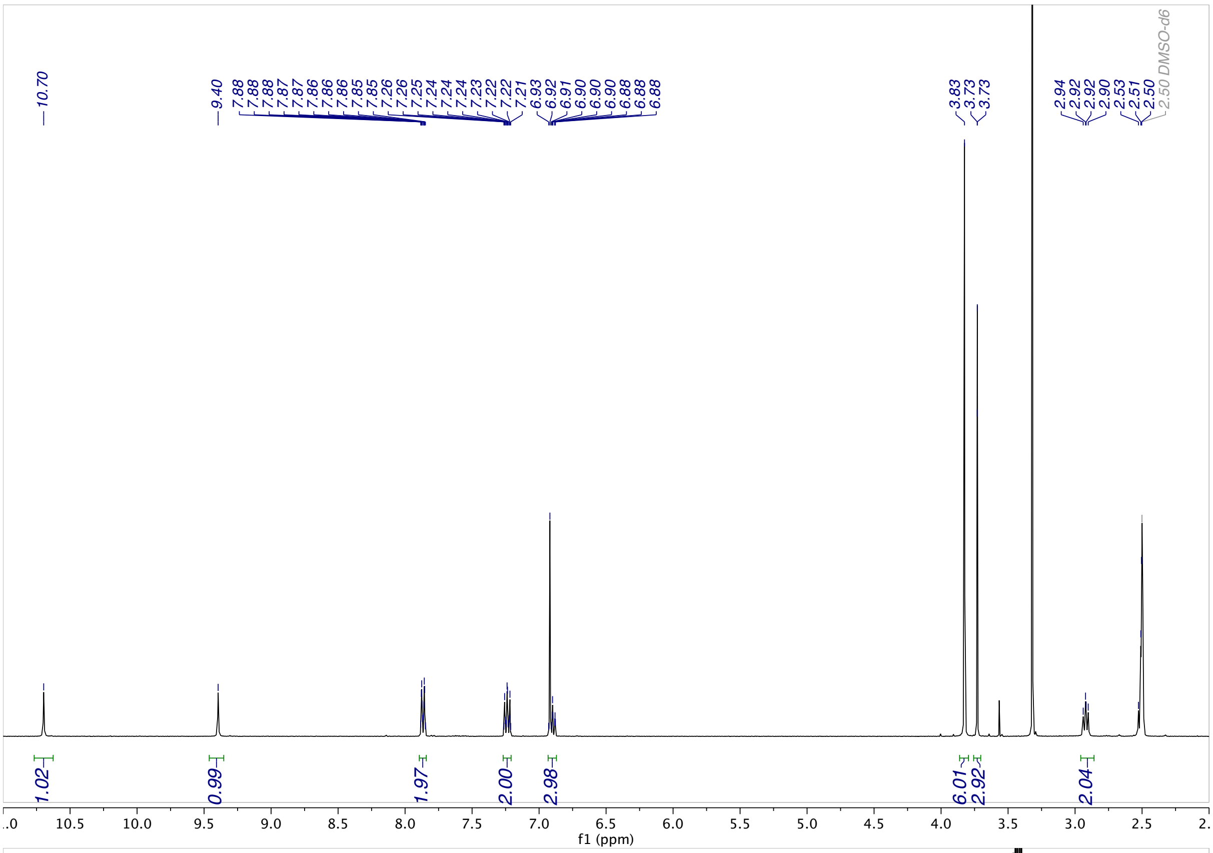

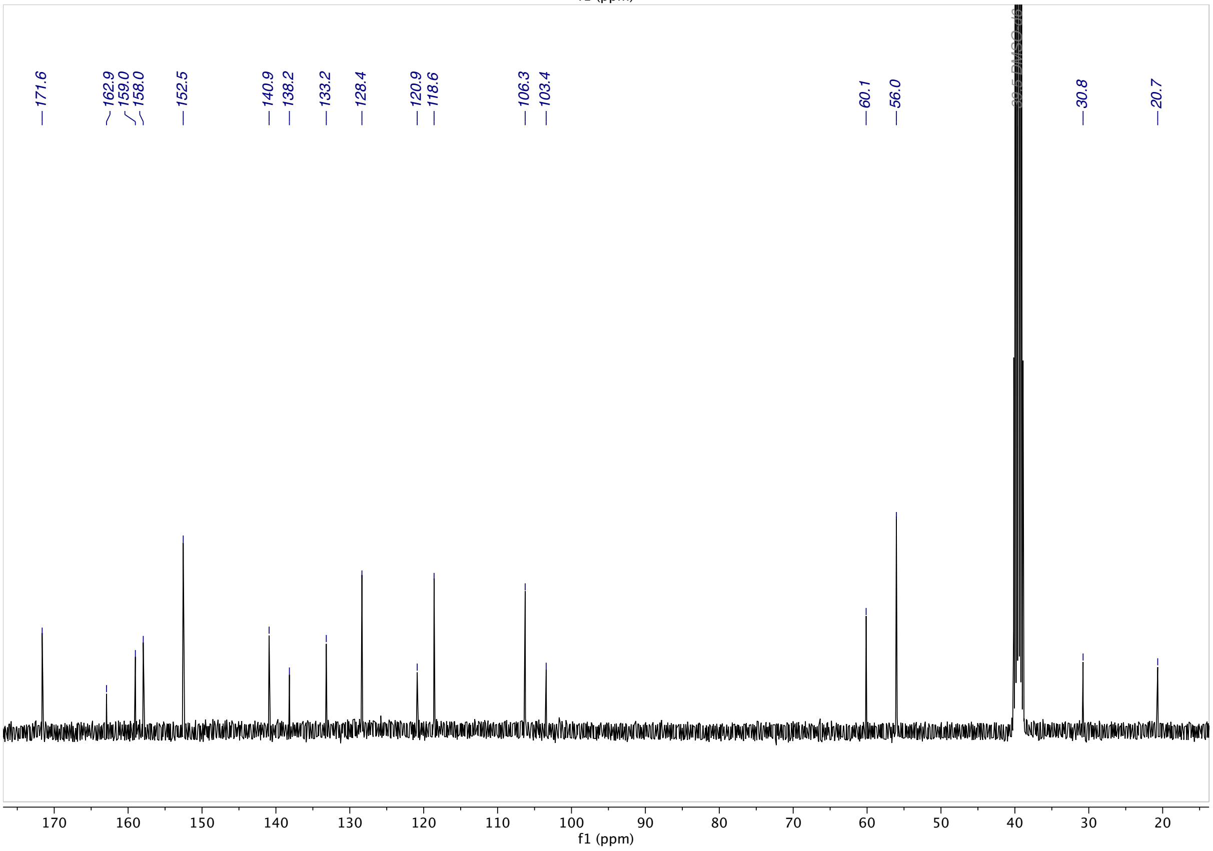

Figure S41. 4-(Naphthalen-2-yl)-2-(phenylamino)-5,6-dihydropyrido[2,3-*d*]pyrimidin-7(8*H*)-one (22e)

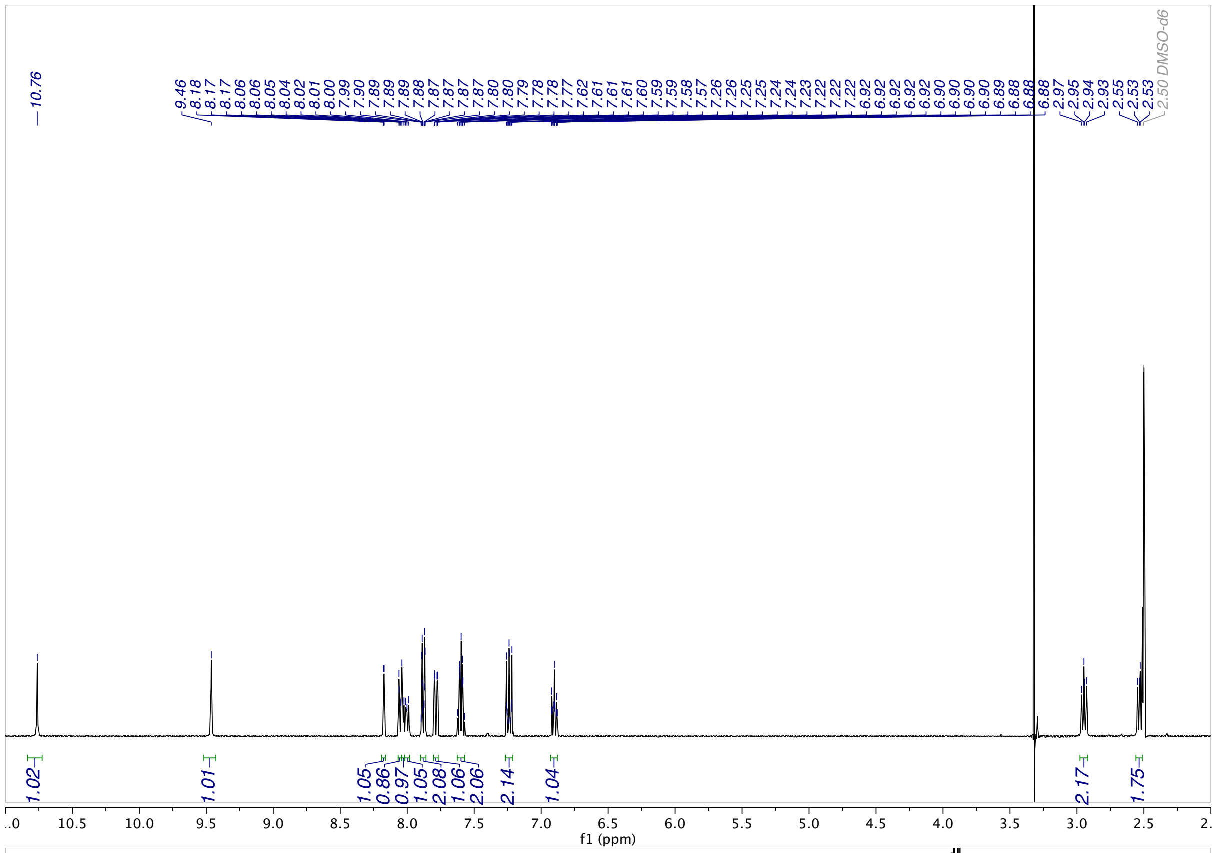

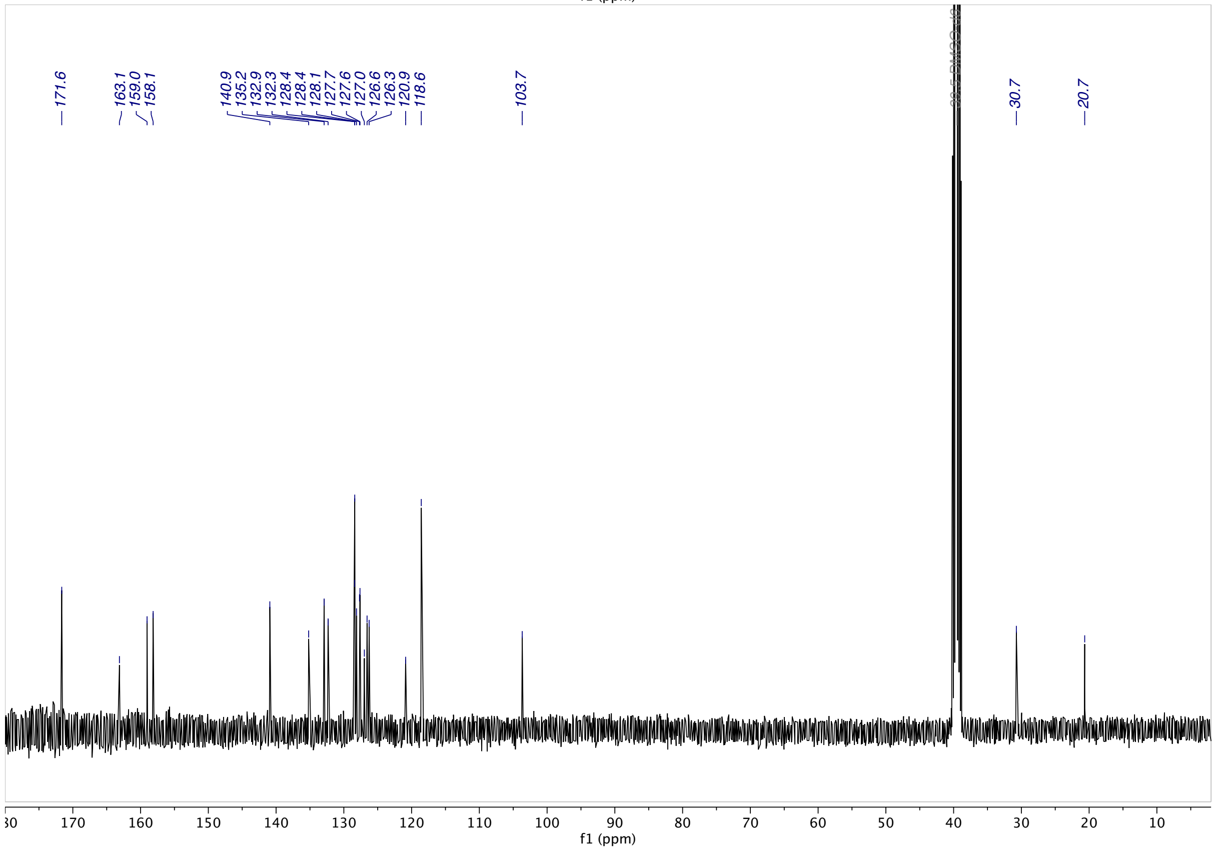

Figure S42. 4-(4-(Dimethylamino)phenyl)-2-(phenylamino)-5,6-dihydropyrido[2,3-*d*]pyrimidin-7(8*H*)-one (22f)

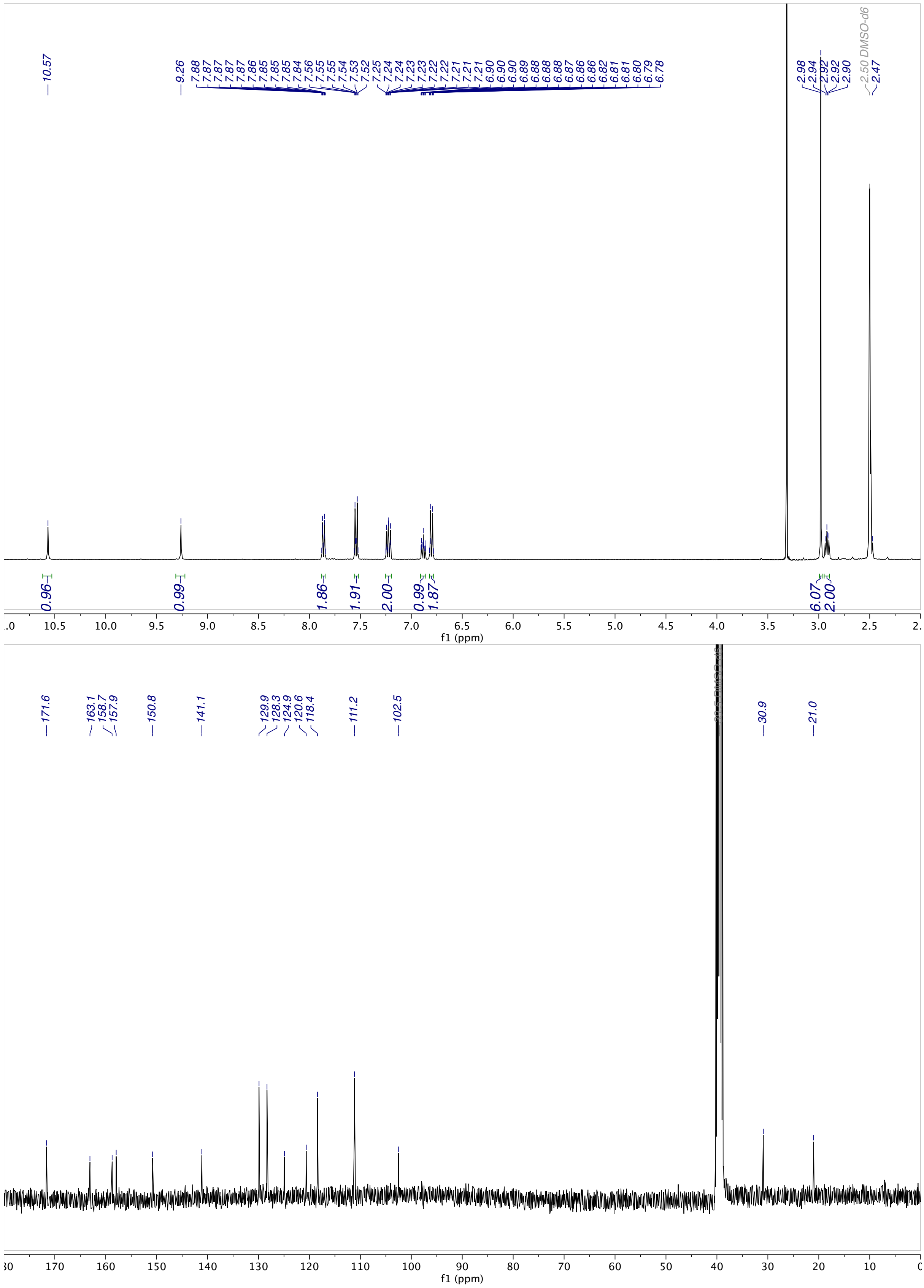

Figure S43. 4-(4-Fluorophenyl)-2-(phenylamino)-5,6-dihydropyrido[2,3-*d*]pyrimidin-7(8*H*)-one (22g)

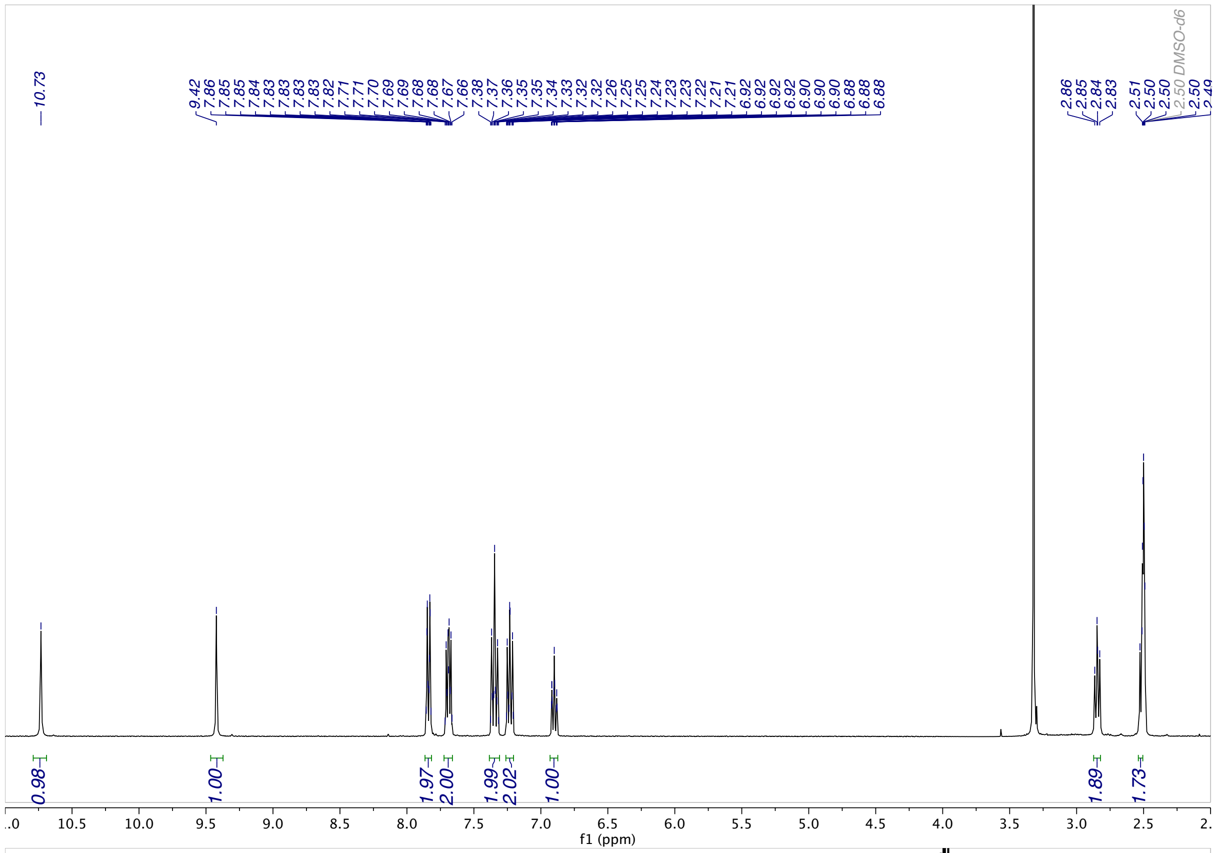

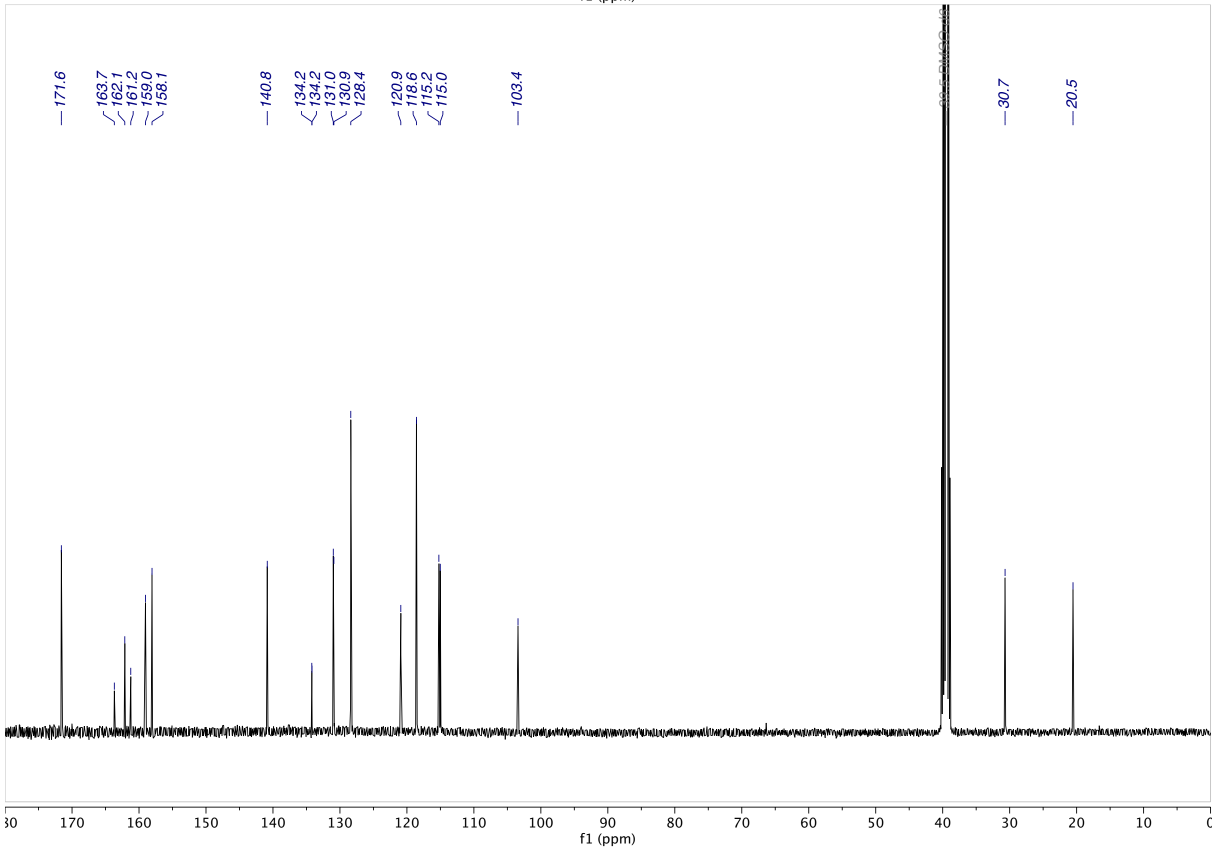

Figure S44. 4-(4-Nitrophenyl)-2-(phenylamino)-5,6-dihydropyrido[2,3-*d*]pyrimidin-7(8*H*)-one (22h)

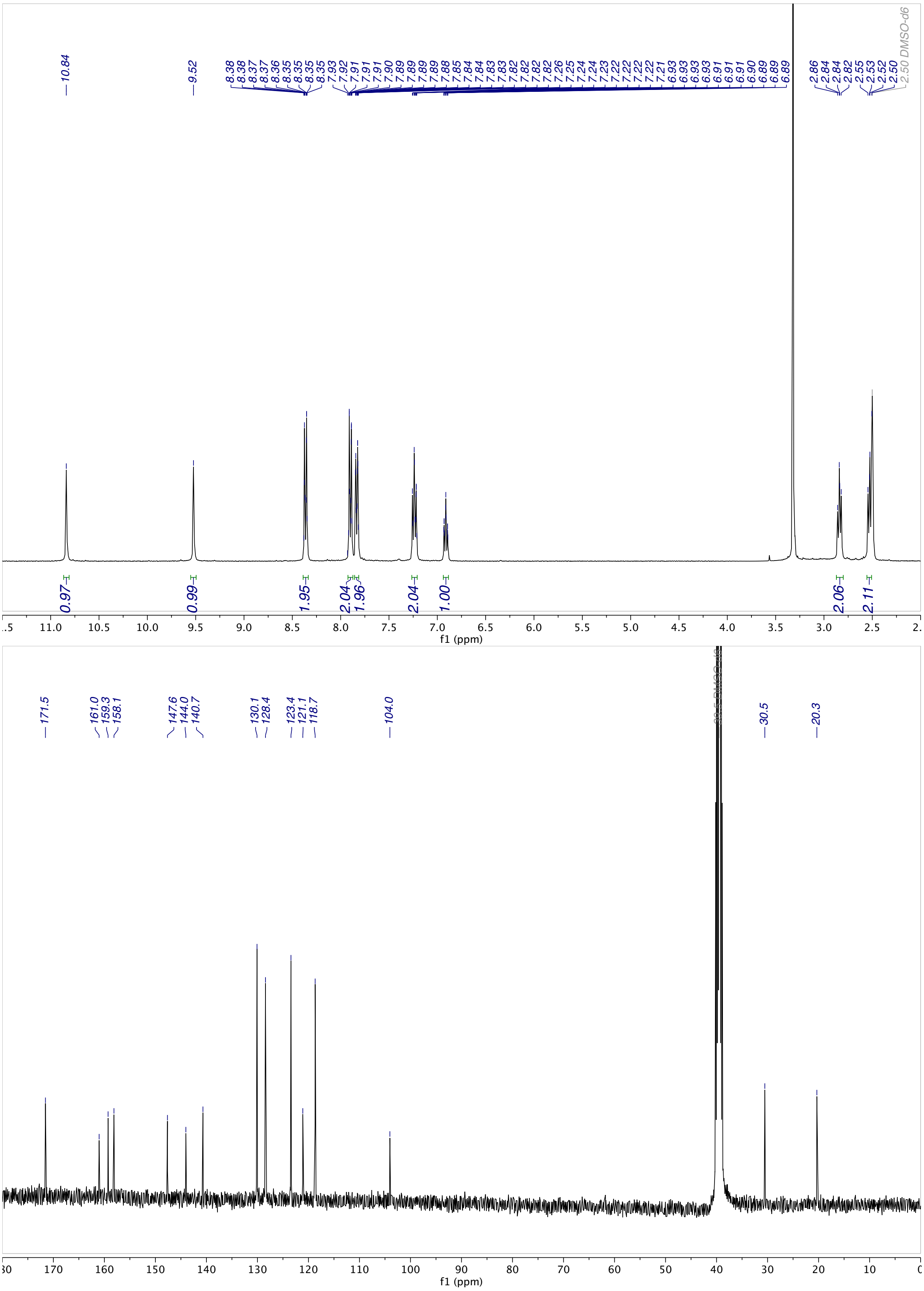

Figure S45. 4-(7-Oxo-2-(phenylamino)-5,6,7,8-tetrahydropyrido[2,3-*d*]pyrimidin-4-yl)benzonitrile (**22i**)

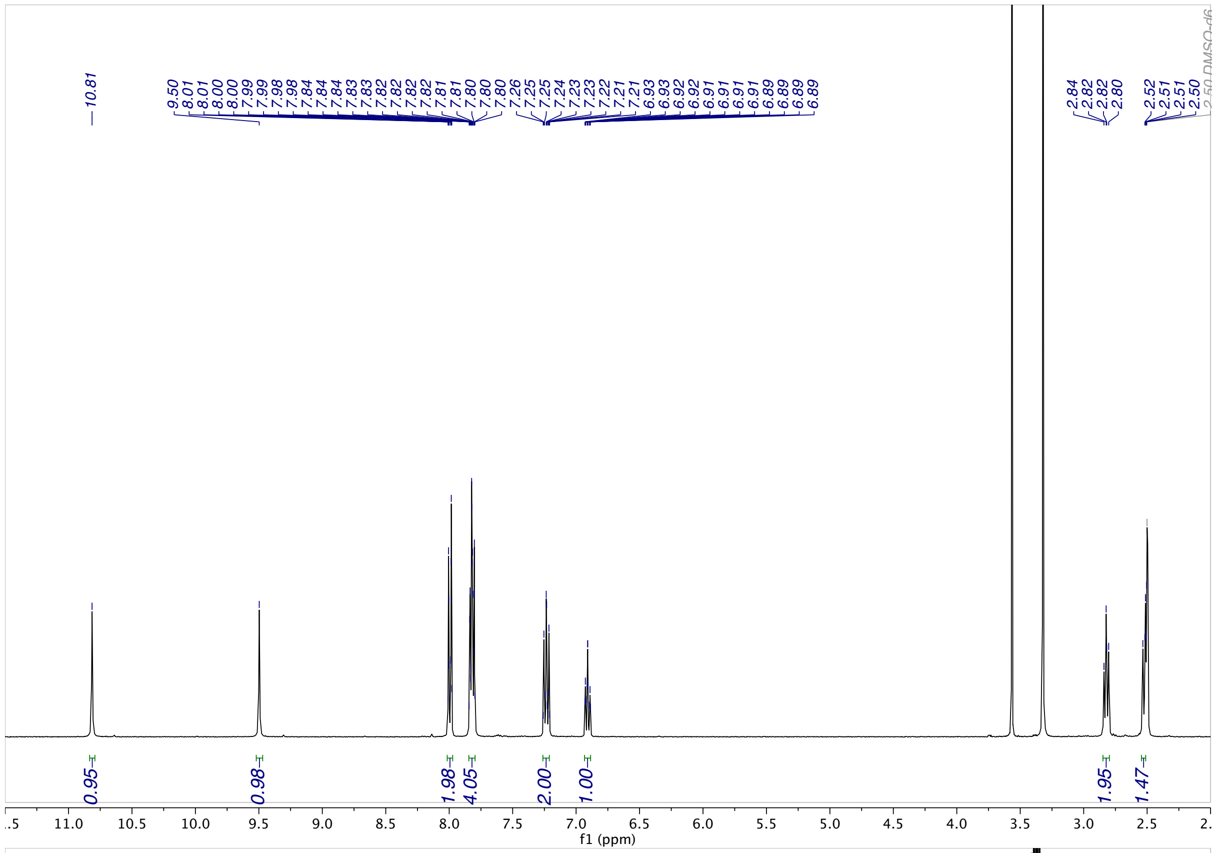

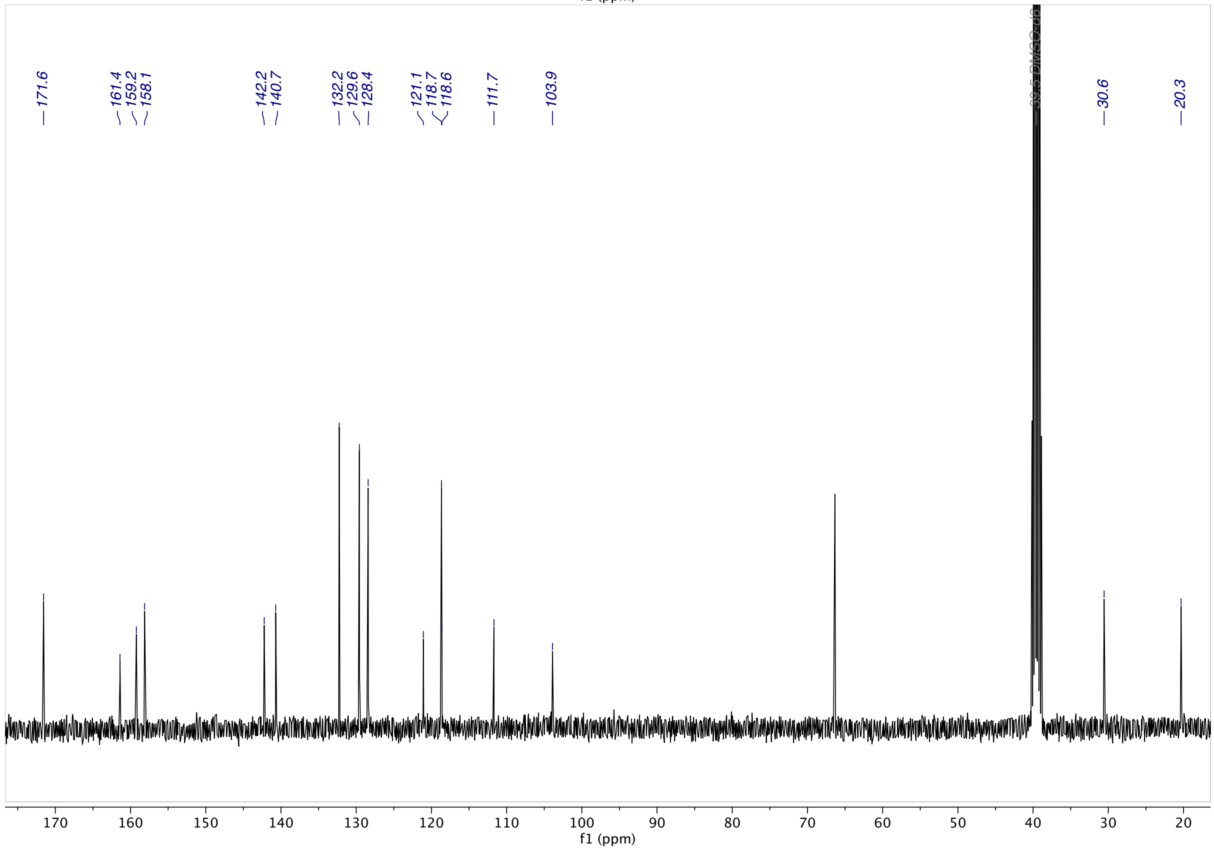

Figure S46. 2-(Phenylamino)-4-(thiophen-2-yl)-5,6-dihydropyrido[2,3-*d*]pyrimidin-7(8*H*)-one (22j)

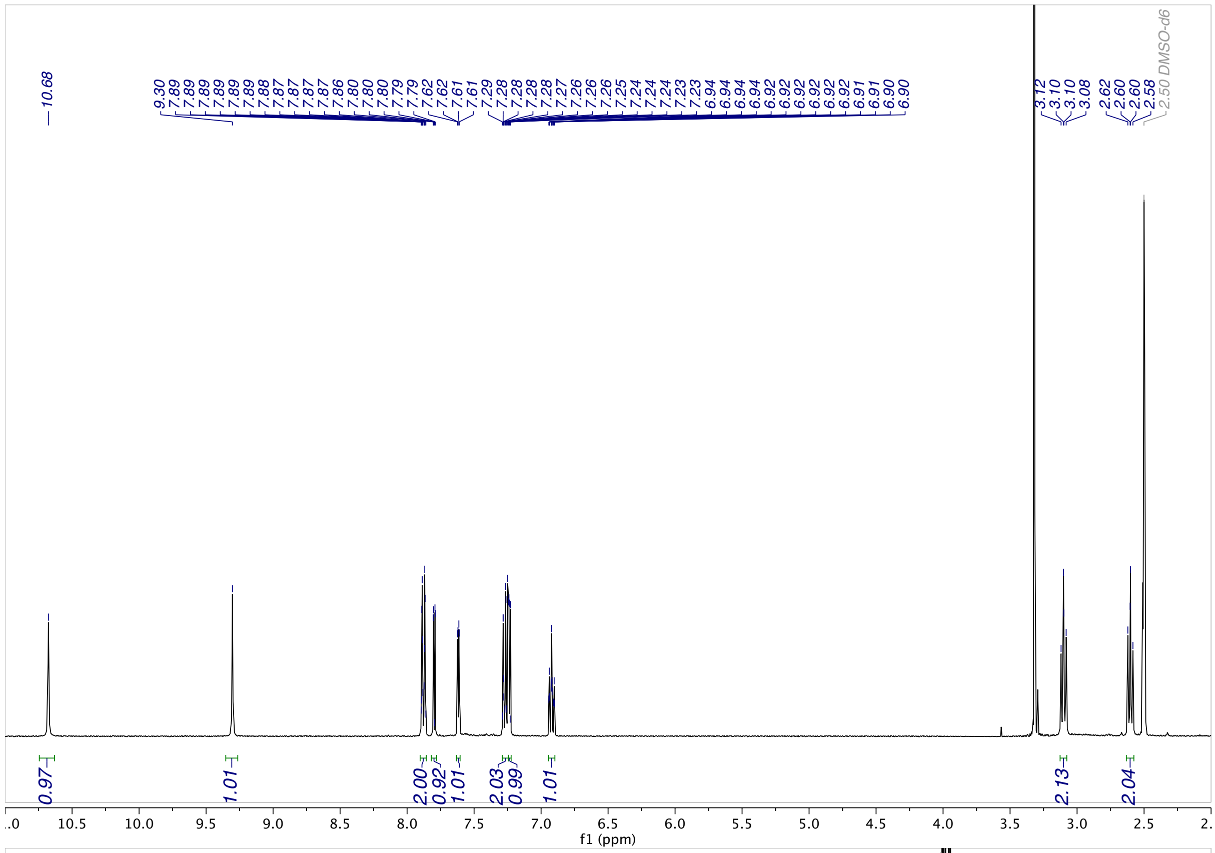

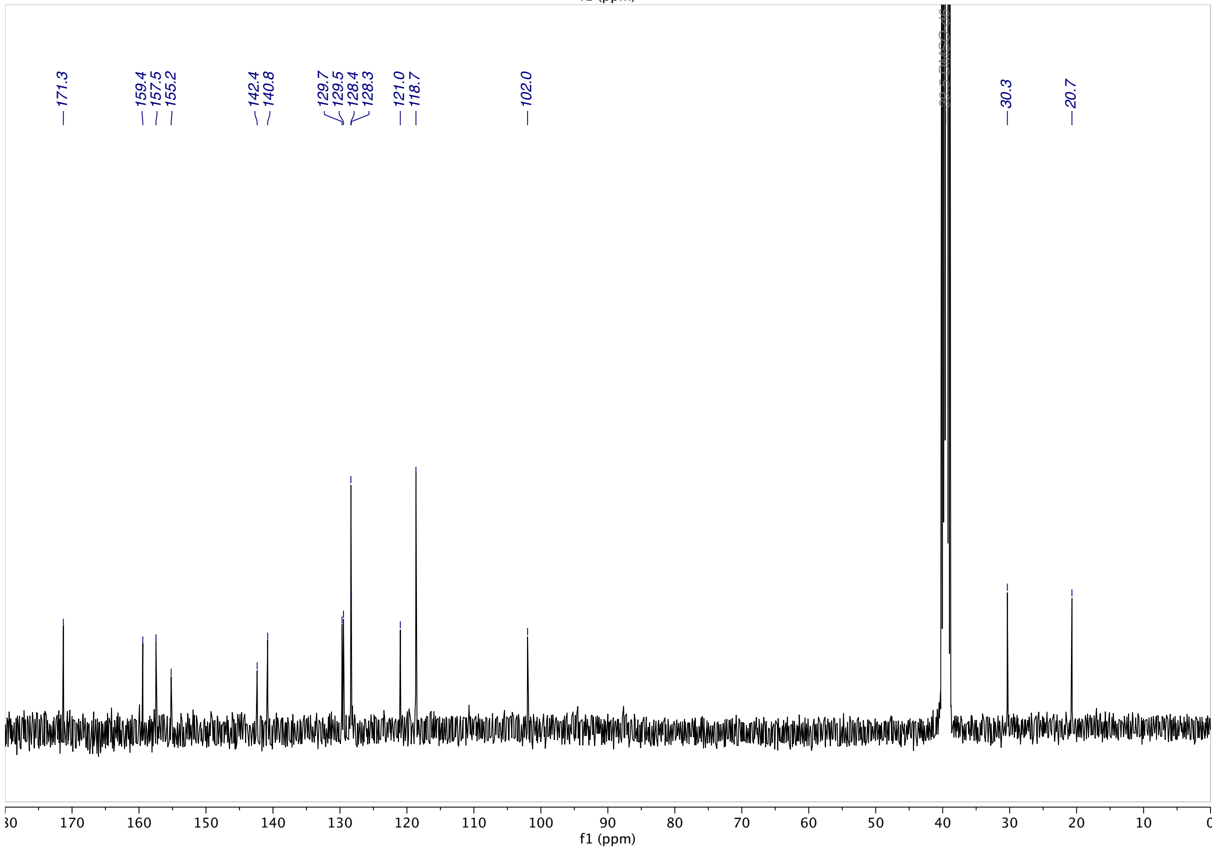

Figure S47. 4-(3-(Dimethylamino)prop-1-yn-1-yl)-2-(phenylamino)-5,6-dihydropyrido[2,3-*d*]pyrimidin-7(8*H*)-one (23a)

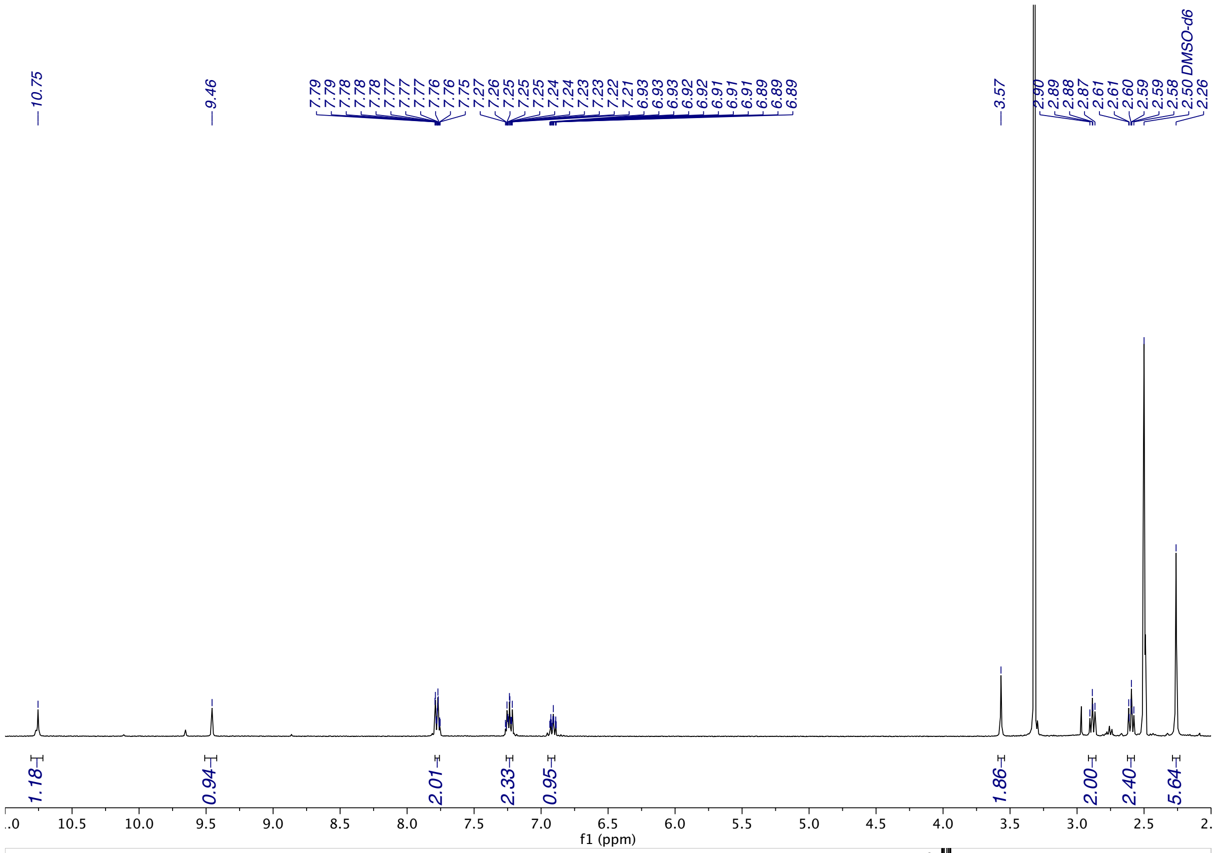

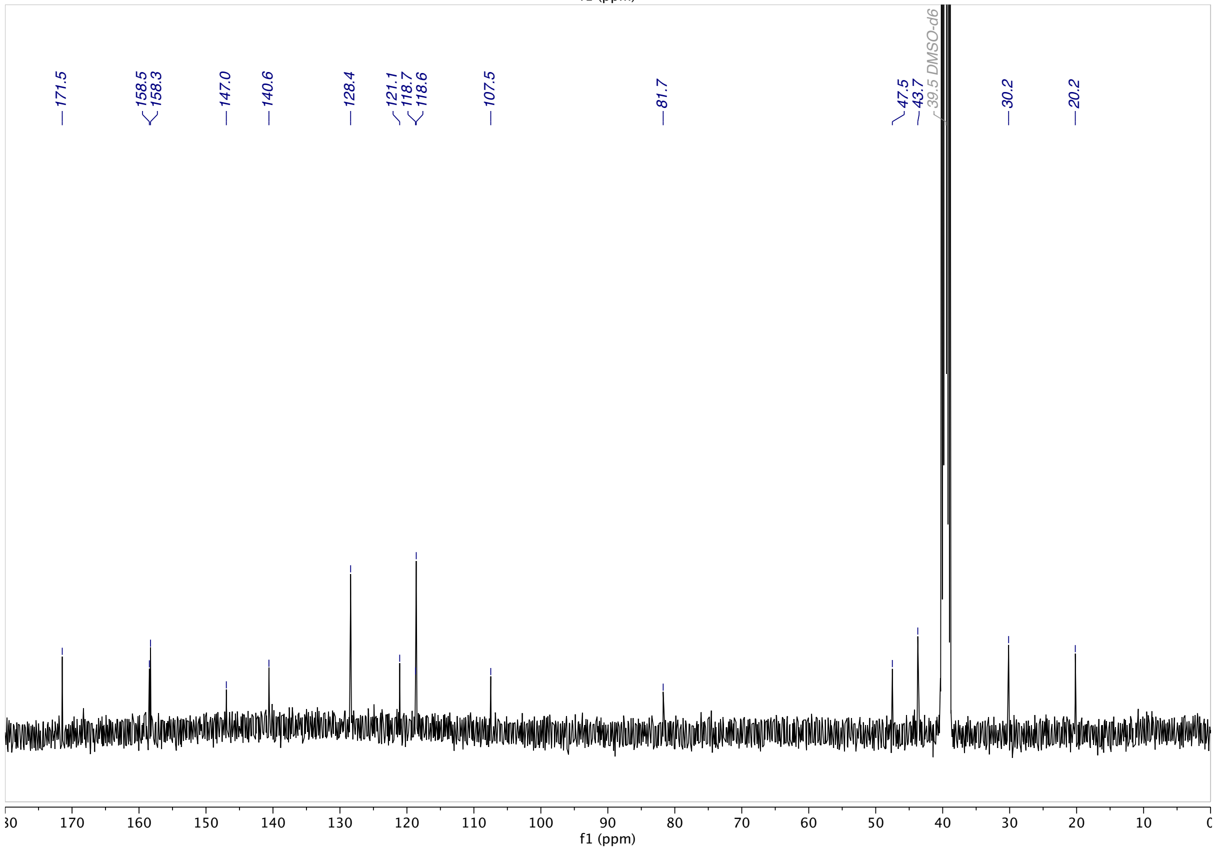

Figure S48. 2-(Phenylamino)-4-((trimethylsilyl)ethynyl)-5,6-dihydropyrido[2,3-*d*]pyrimidin-7(8*H*)-one (23b)

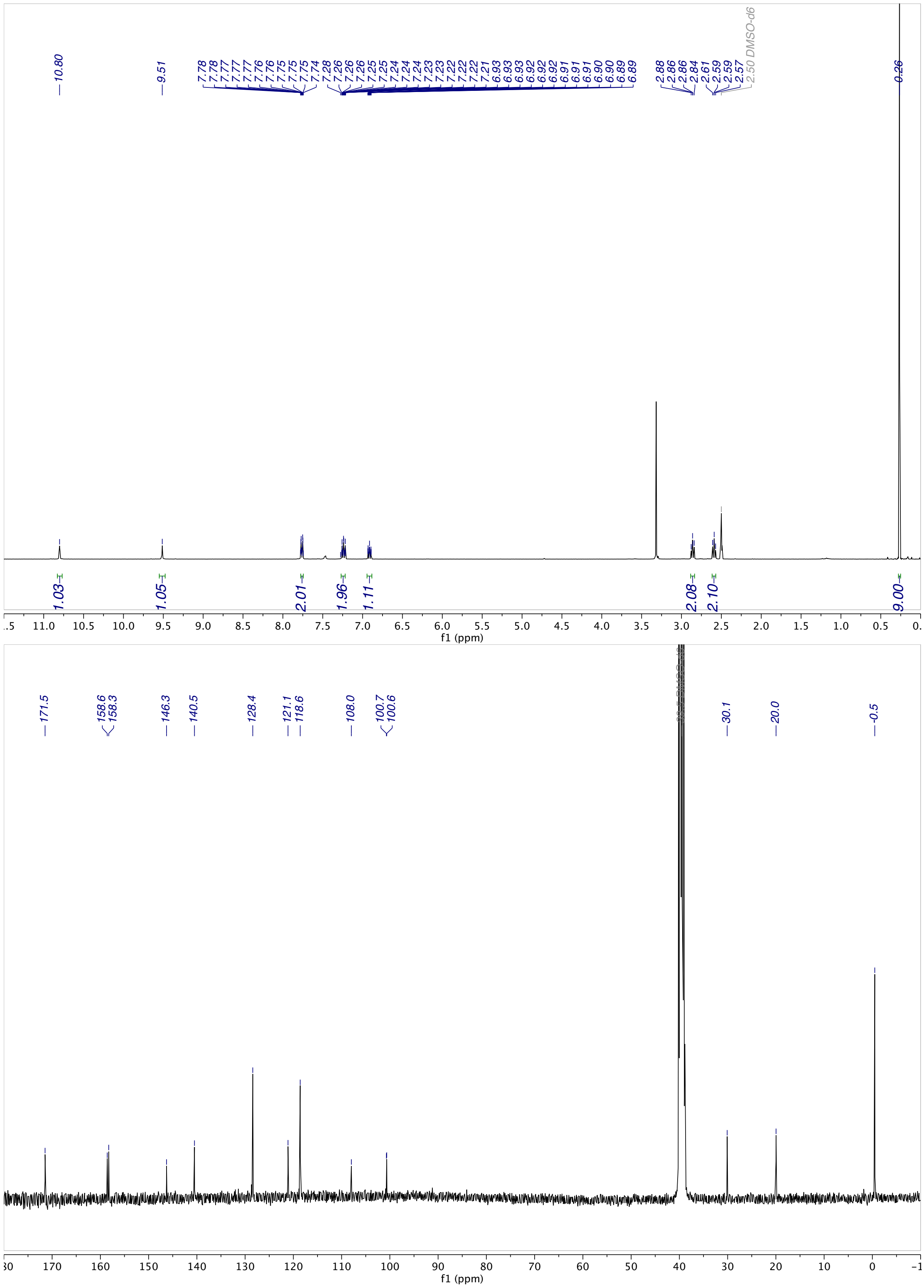

Figure S49. 4-Ethynyl-2-(phenylamino)-5,6-dihydropyrido[2,3-*d*]pyrimidin-7(8*H*)-one (24)

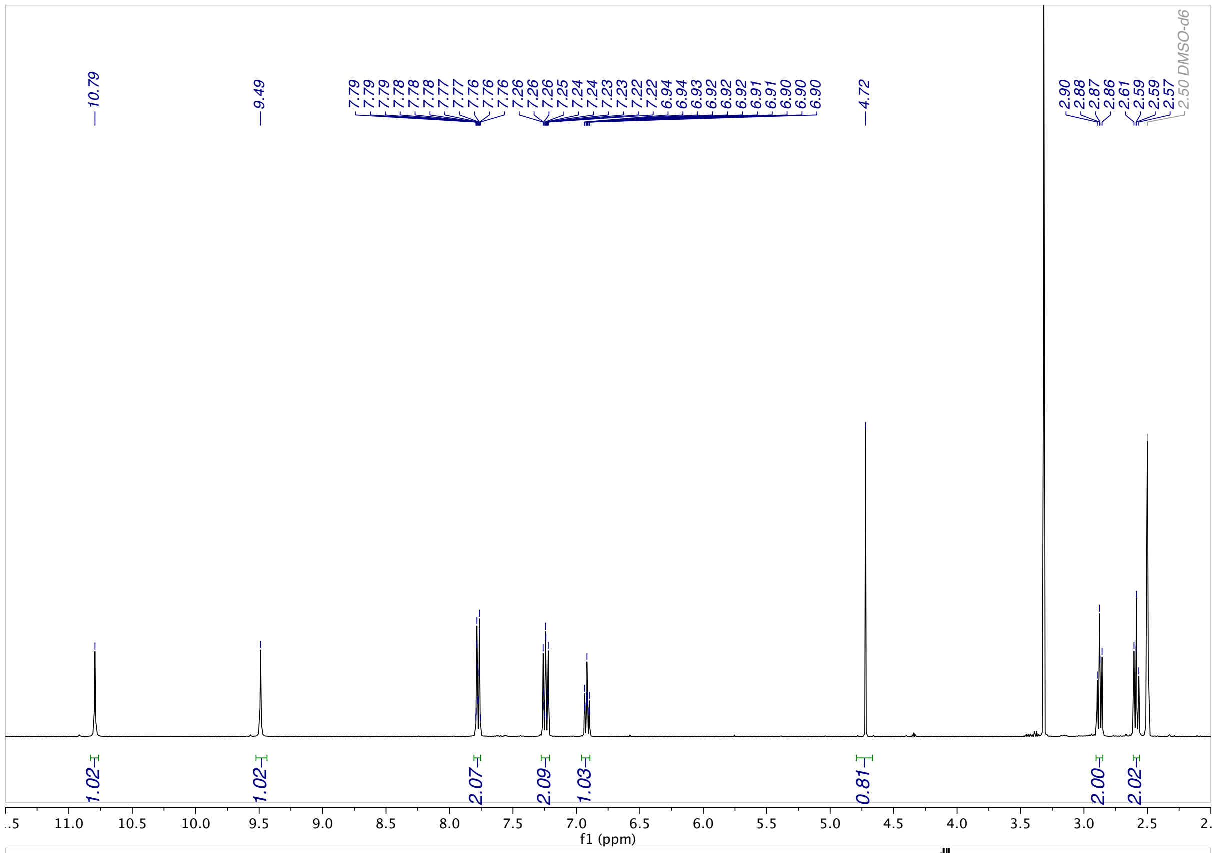

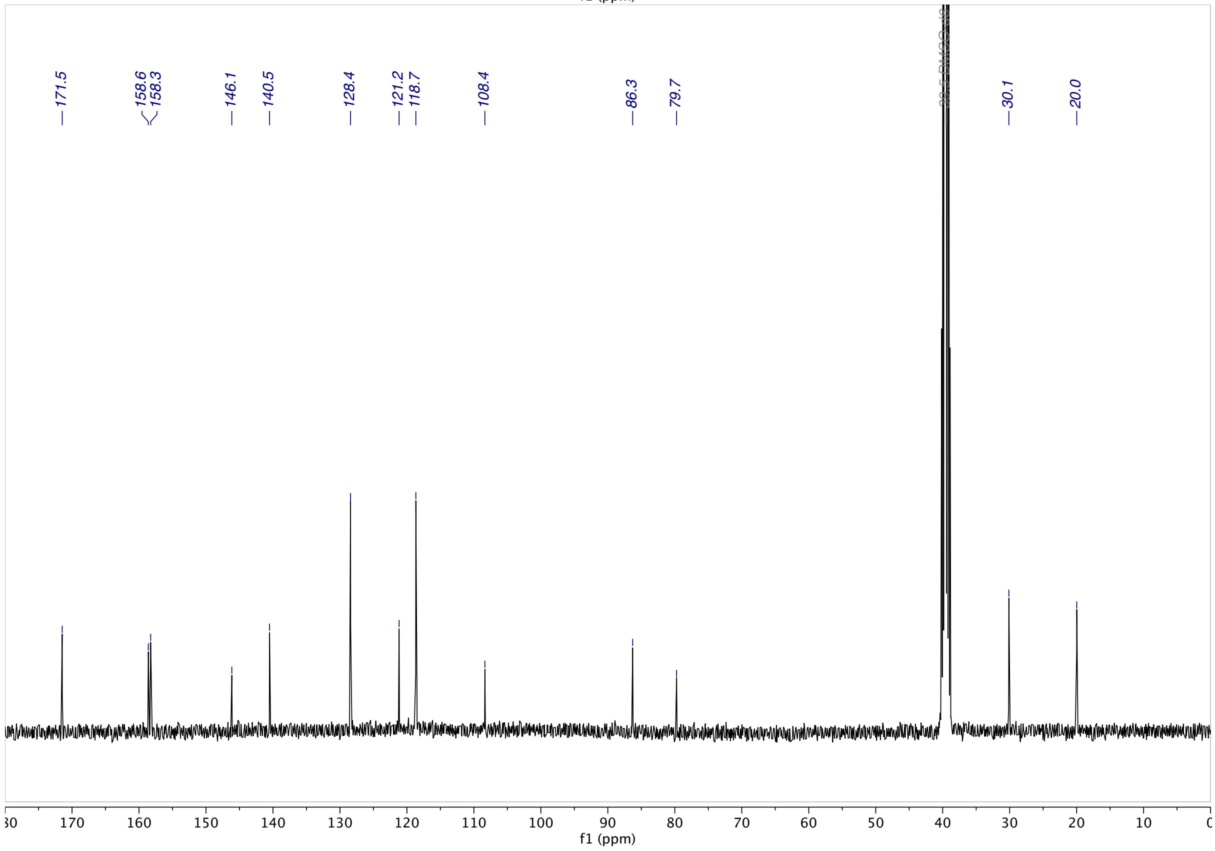

Figure S50. 2-(Phenylamino)-4-(phenylethynyl)-5,6-dihydropyrido[2,3-*d*]pyrimidin-7(8*H*)-one (25a)

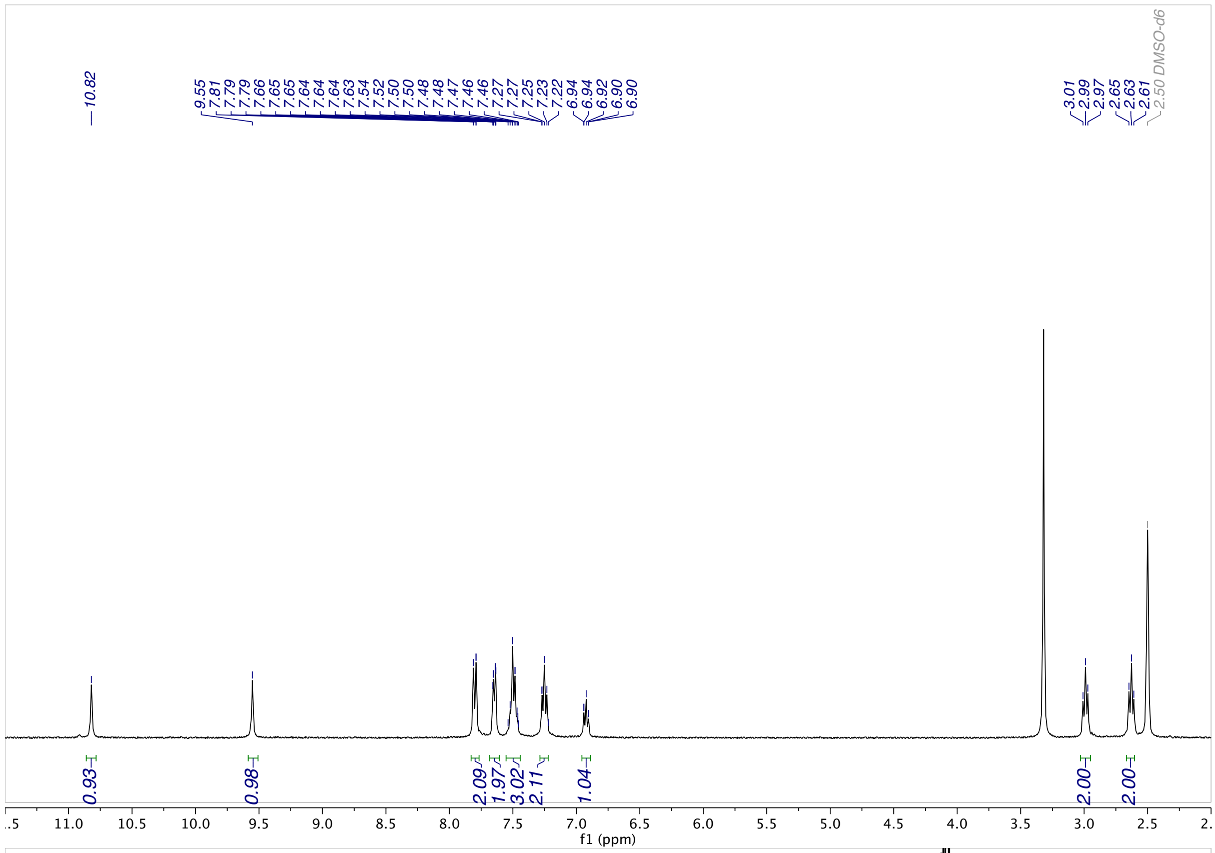

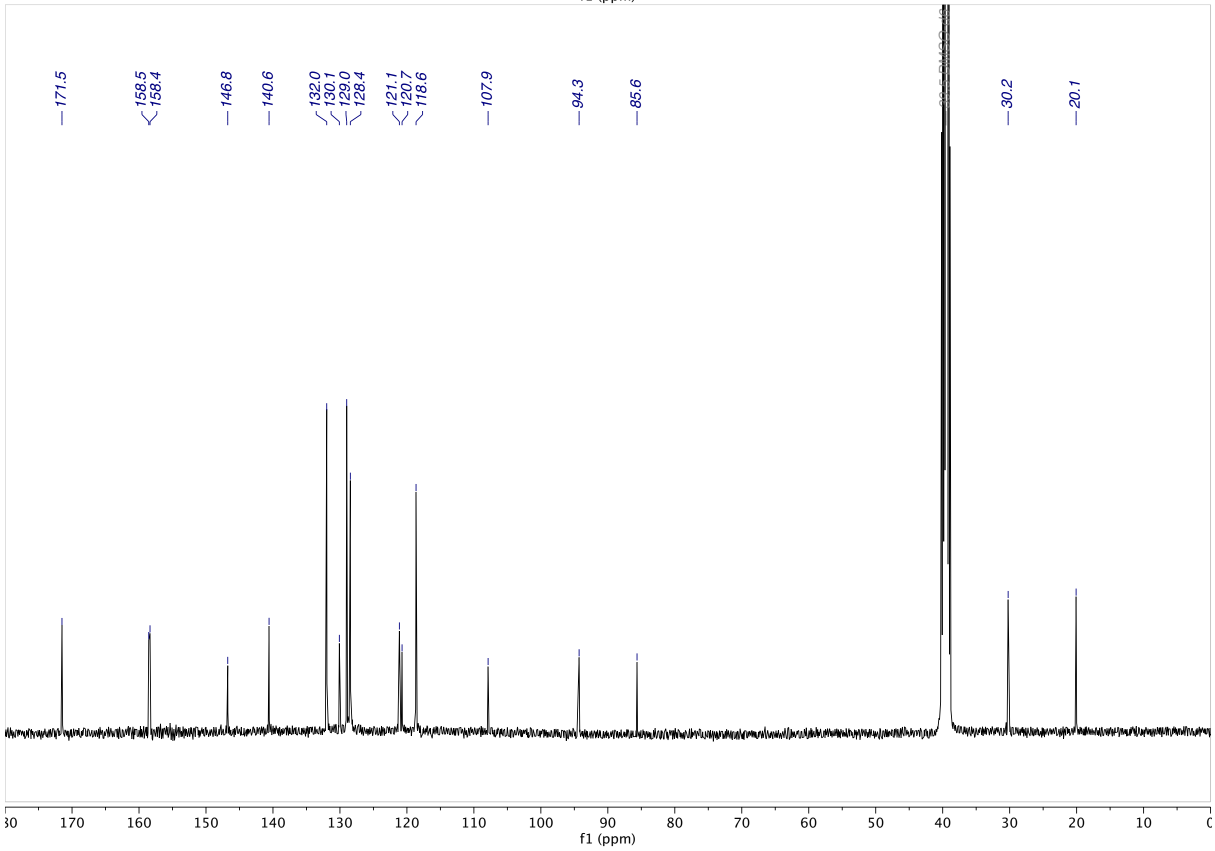

Figure S51. 4-(Naphthalen-1-ylethynyl)-2-(phenylamino)-5,6-dihydropyrido[2,3-*d*]pyrimidin-7(8*H*)-one (25b)

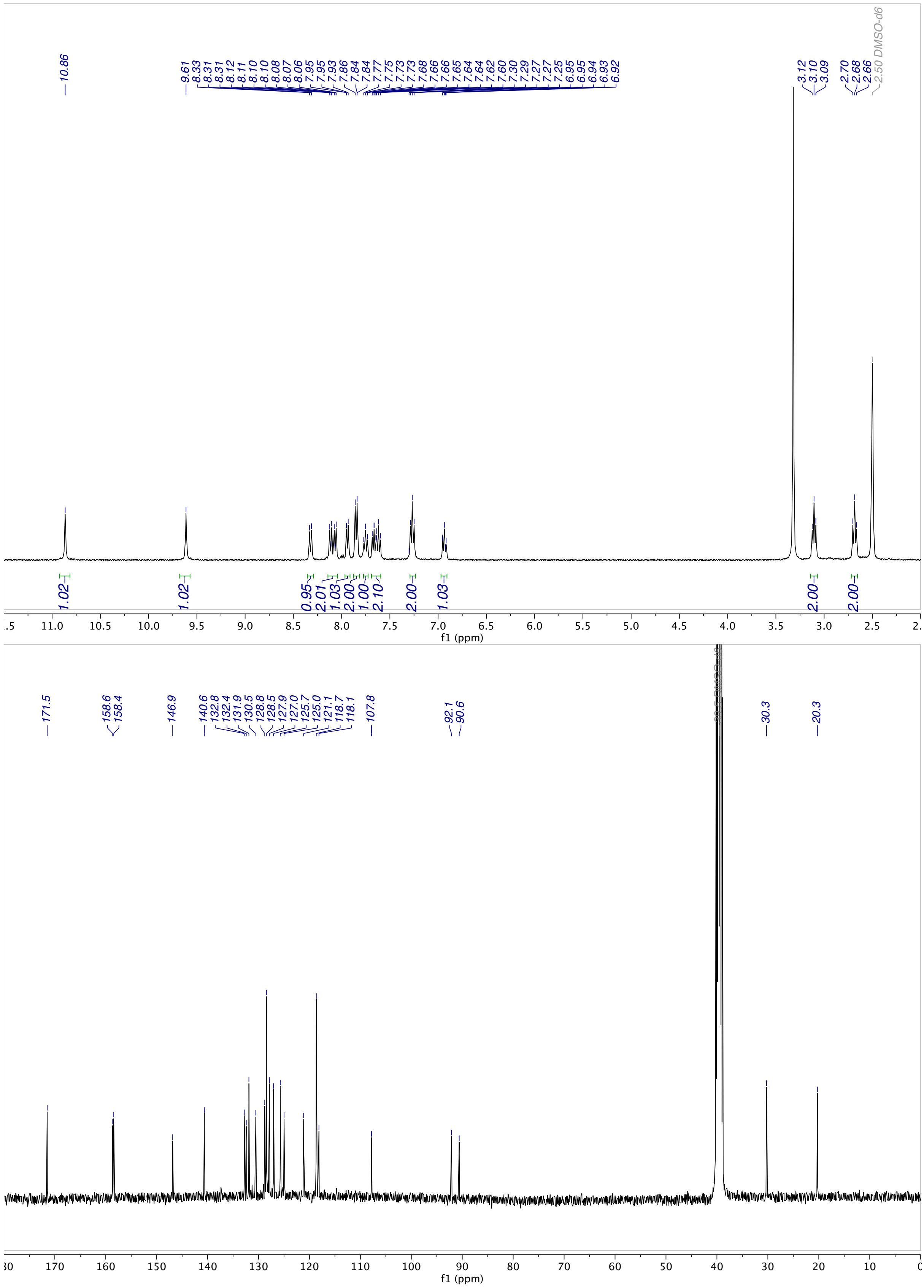

Figure S52. 4-([1,1'-Biphenyl]-4-ylethynyl)-2-(phenylamino)-5,6-dihydropyrido[2,3-*d*]pyrimidin-7(8*H*)-one (25c)

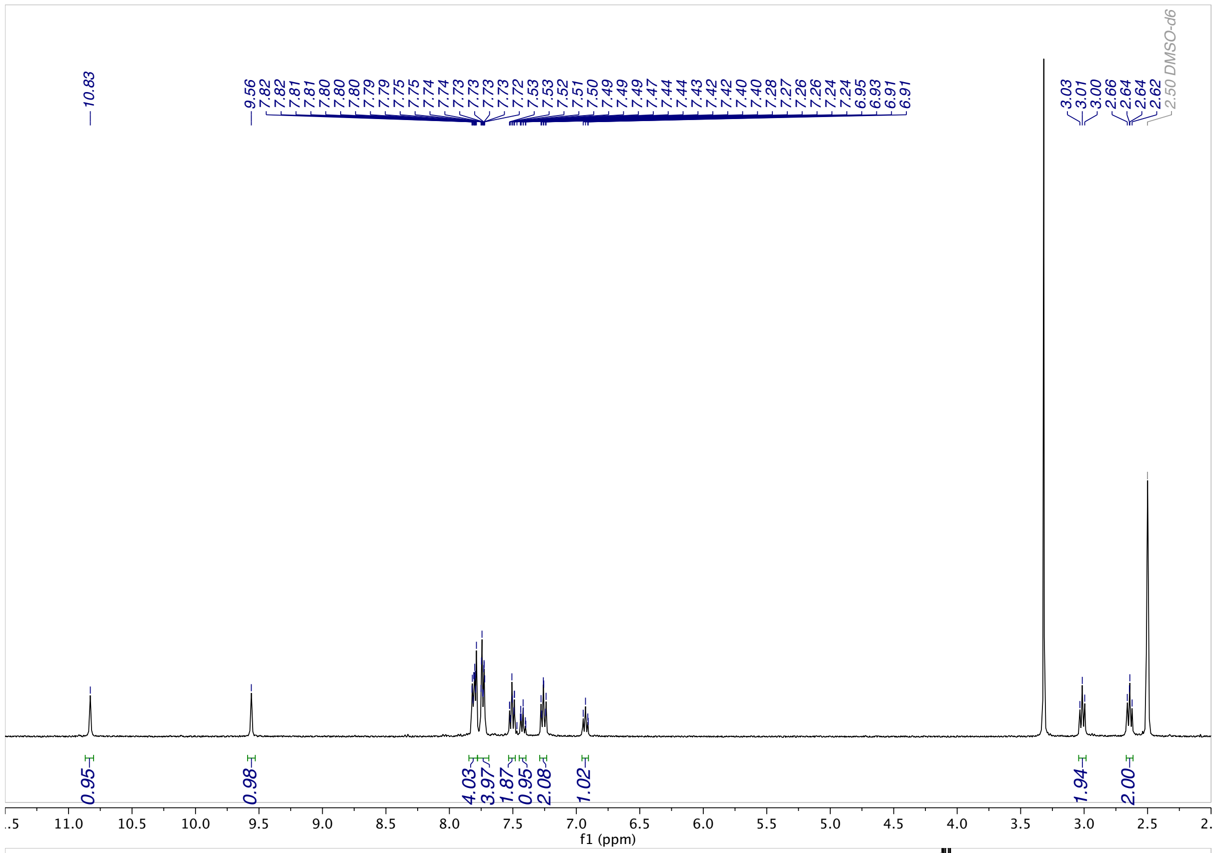

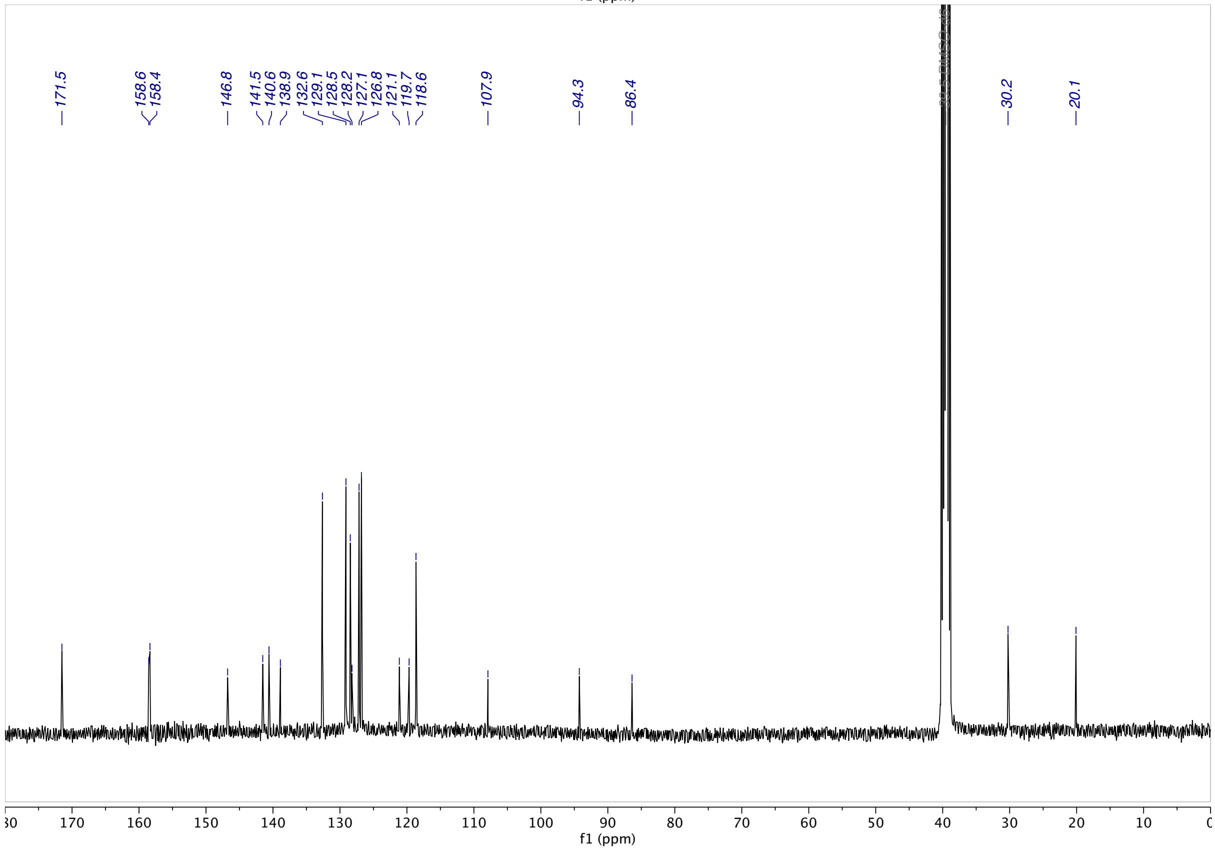

Figure S53. 2-(Phenylamino)-4-(pyridin-3-ylethynyl)-5,6-dihydropyrido[2,3-*d*]pyrimidin-7(8*H*)-one (25d)

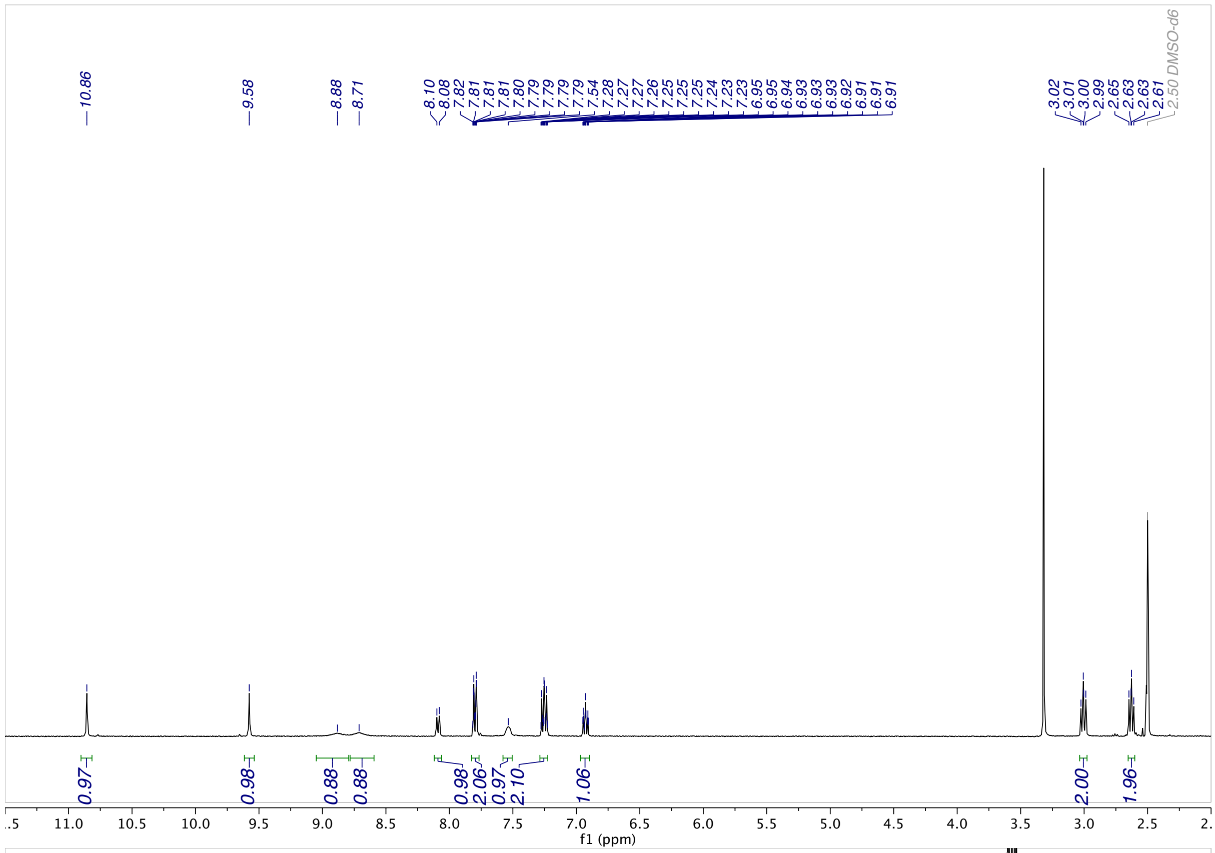

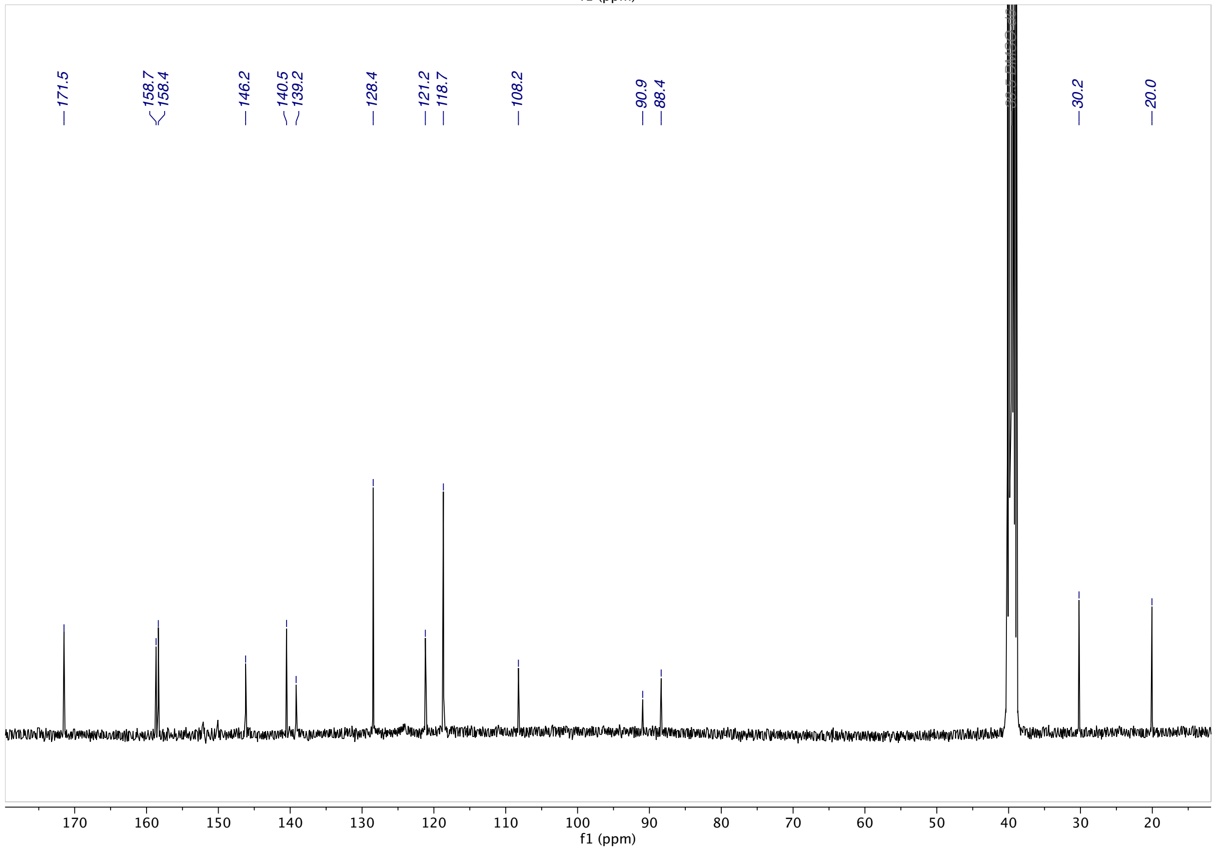

Figure S54. 4-((2,4-Dimethoxyphenyl)ethynyl)-2-(phenylamino)-5,6-dihydropyrido[2,3-*d*]pyrimidin-7(8*H*)-one (25e)

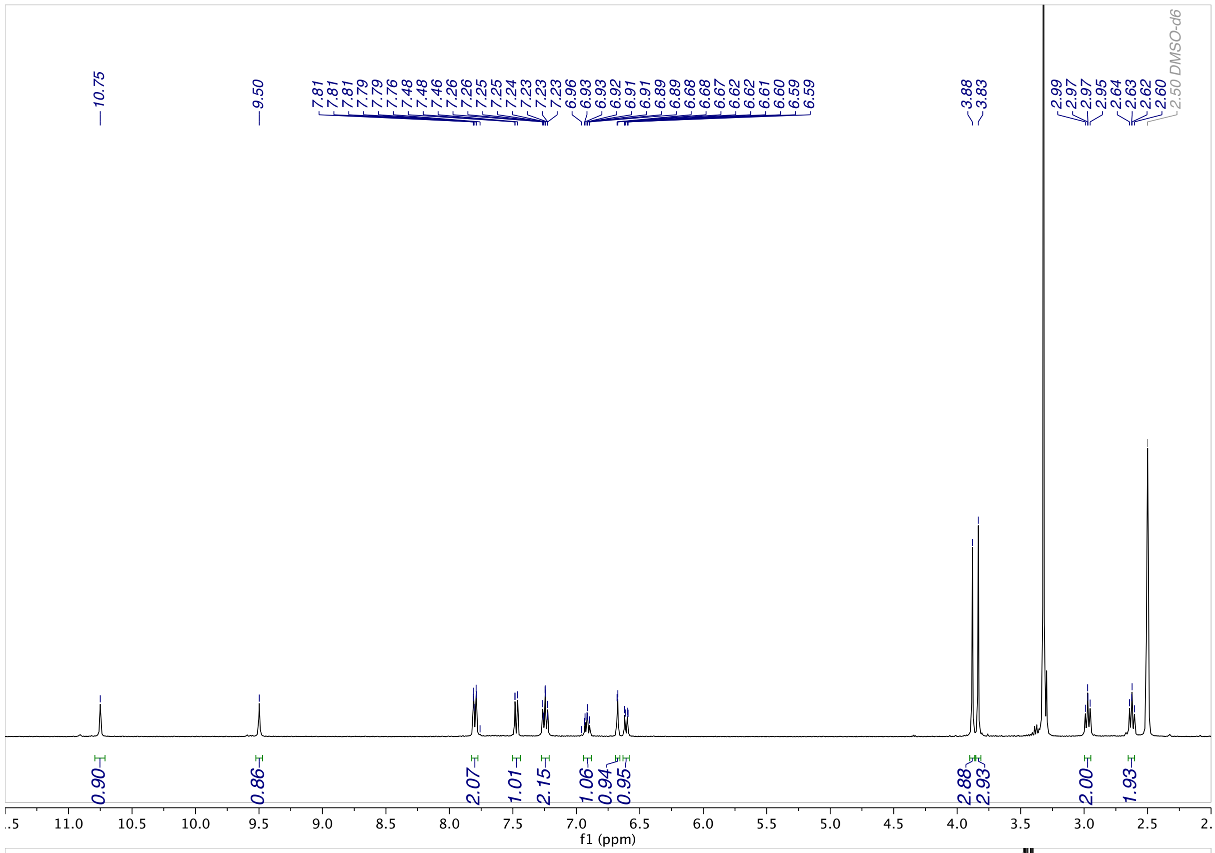

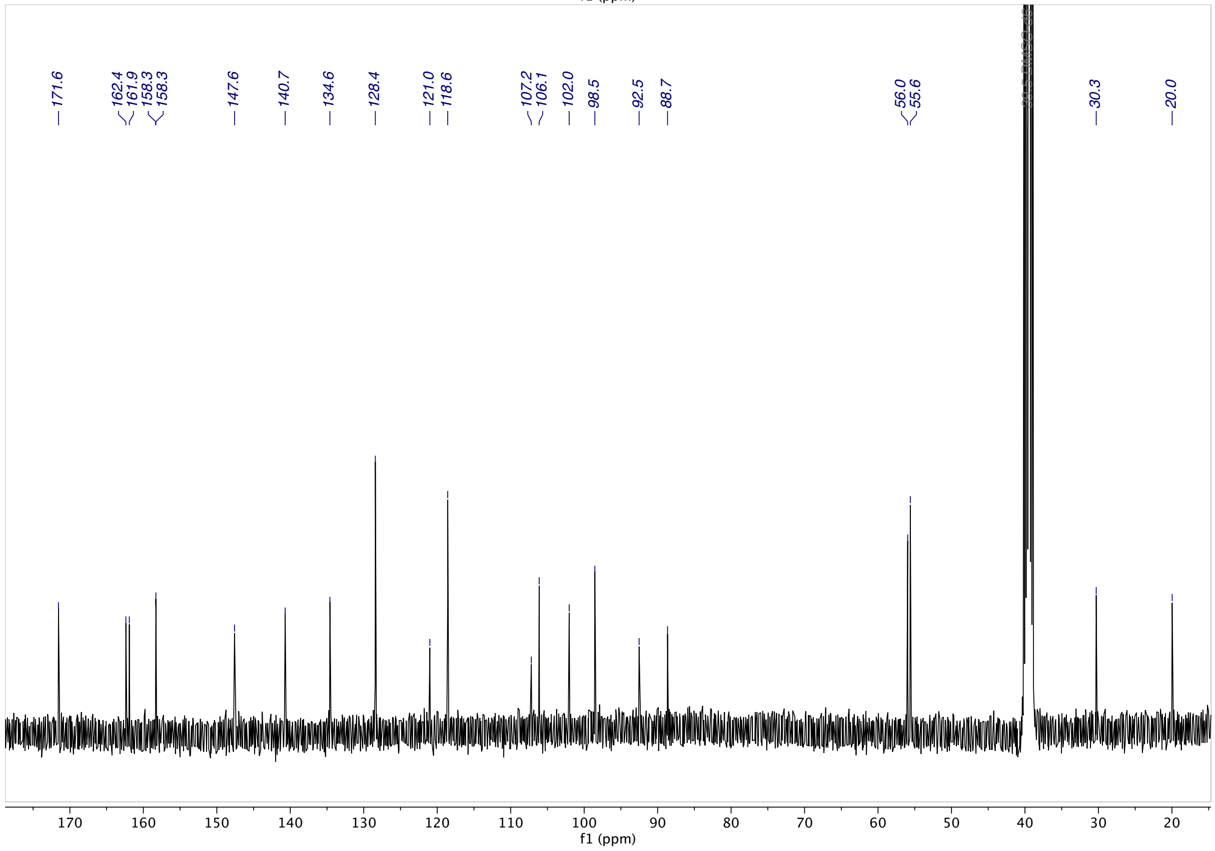

Figure S55. 4-((3-Aminophenyl)ethynyl)-2-(phenylamino)-5,6-dihydropyrido[2,3-*d*]pyrimidin-7(8*H*)-one (25f)

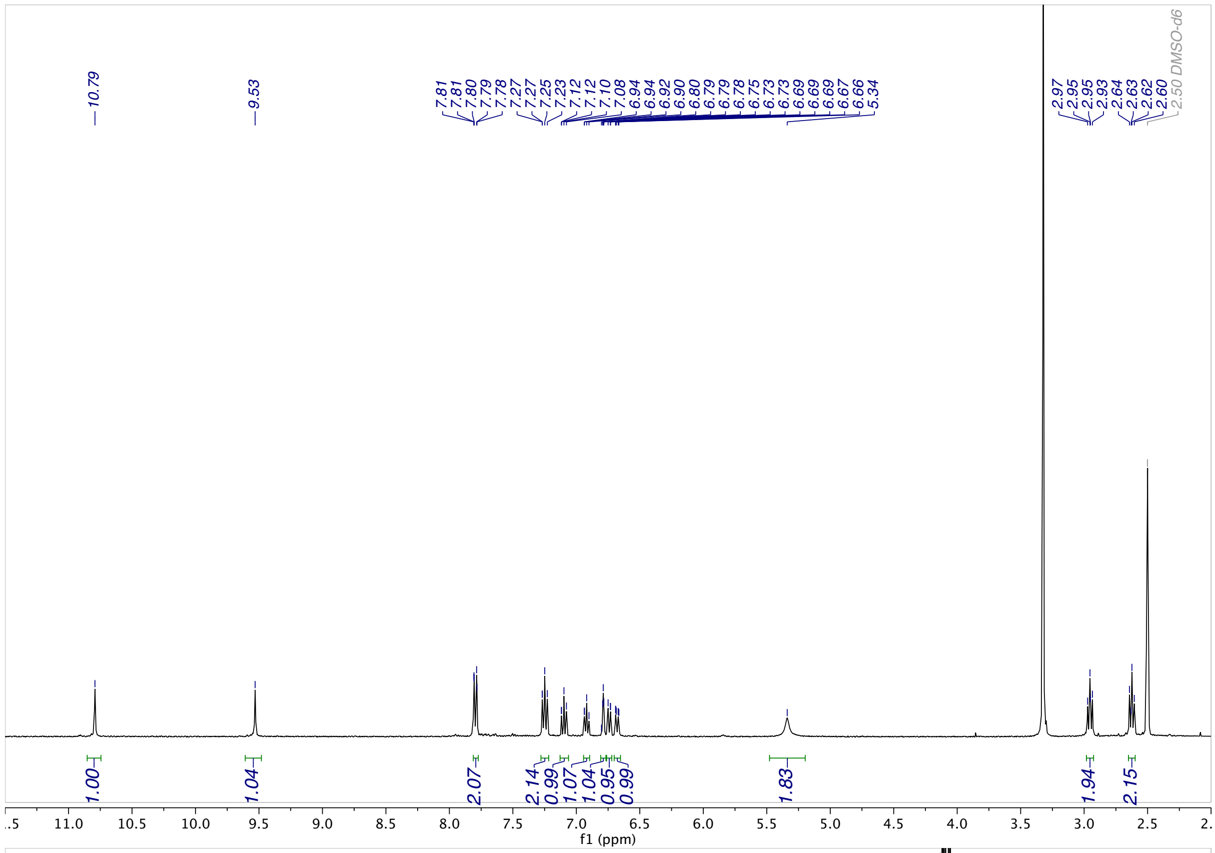

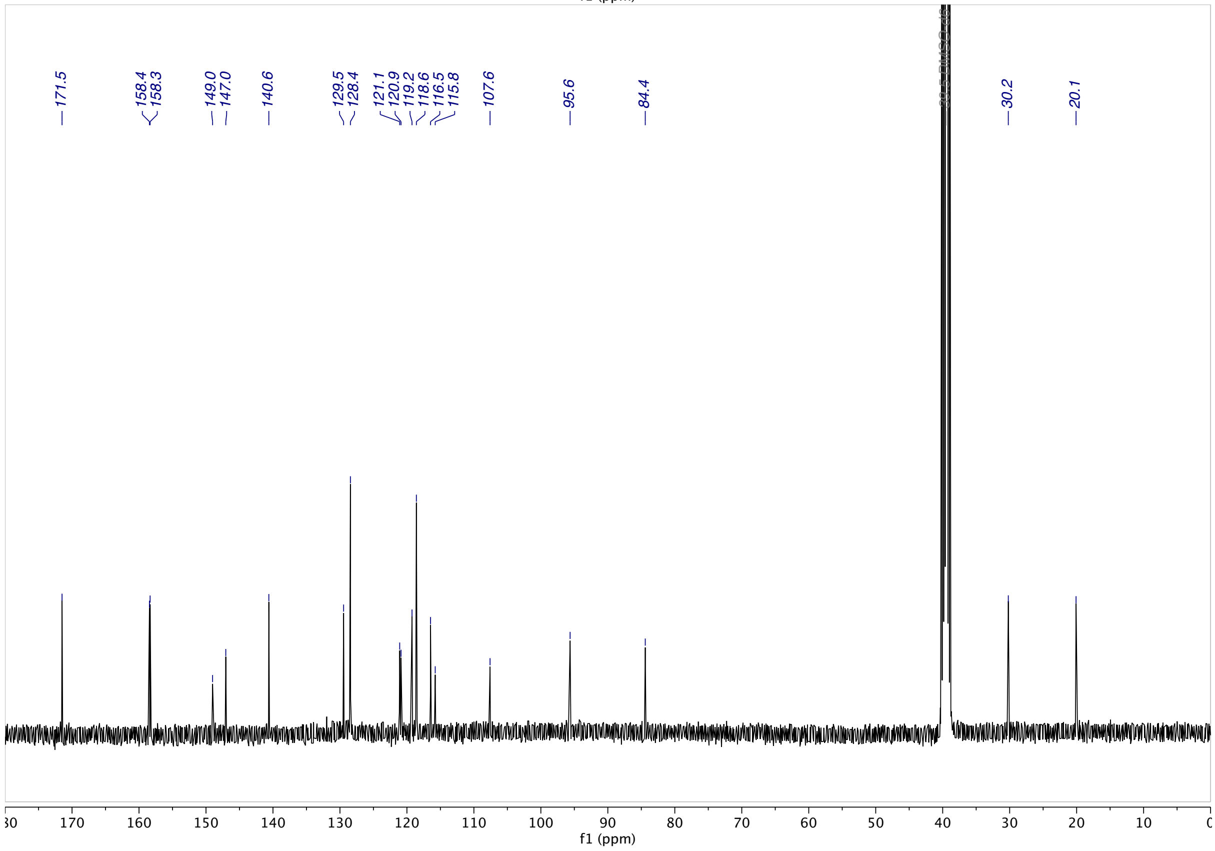

Figure S56. 4-((4-Nitrophenyl)ethynyl)-2-(phenylamino)-5,6-dihydropyrido[2,3-*d*]pyrimidin-7(8*H*)-one (25g)

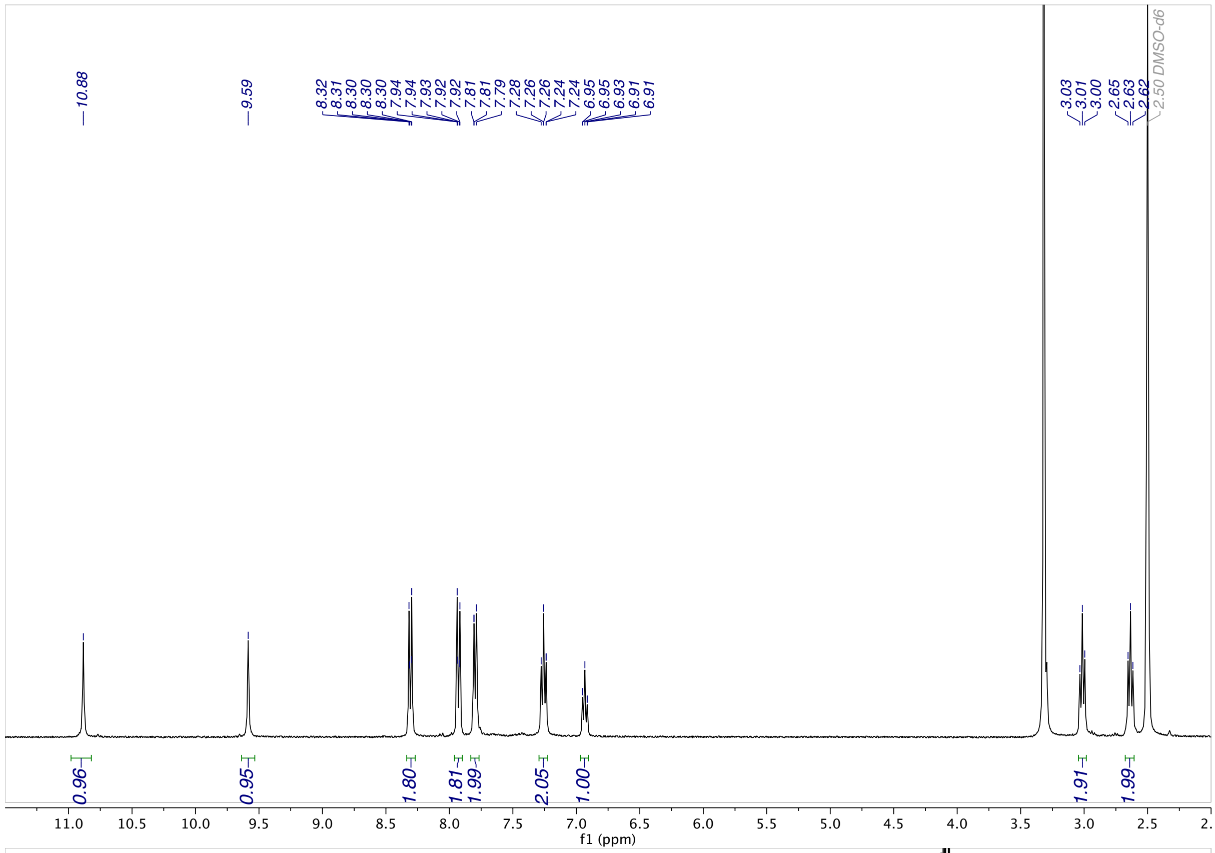

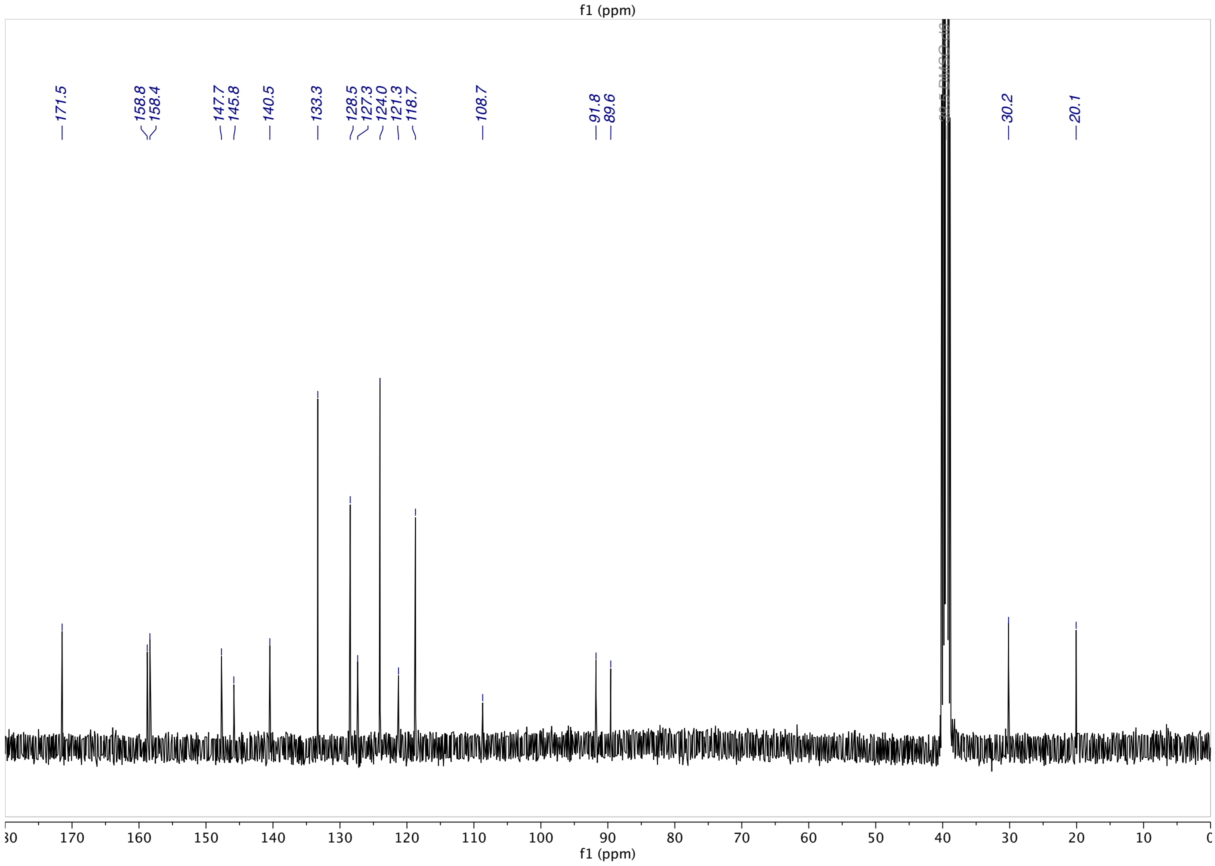

Figure S57. 4-((4-Fluorophenyl)ethynyl)-2-(phenylamino)-5,6-dihydropyrido[2,3-*d*]pyrimidin-7(8*H*)-one (25h)

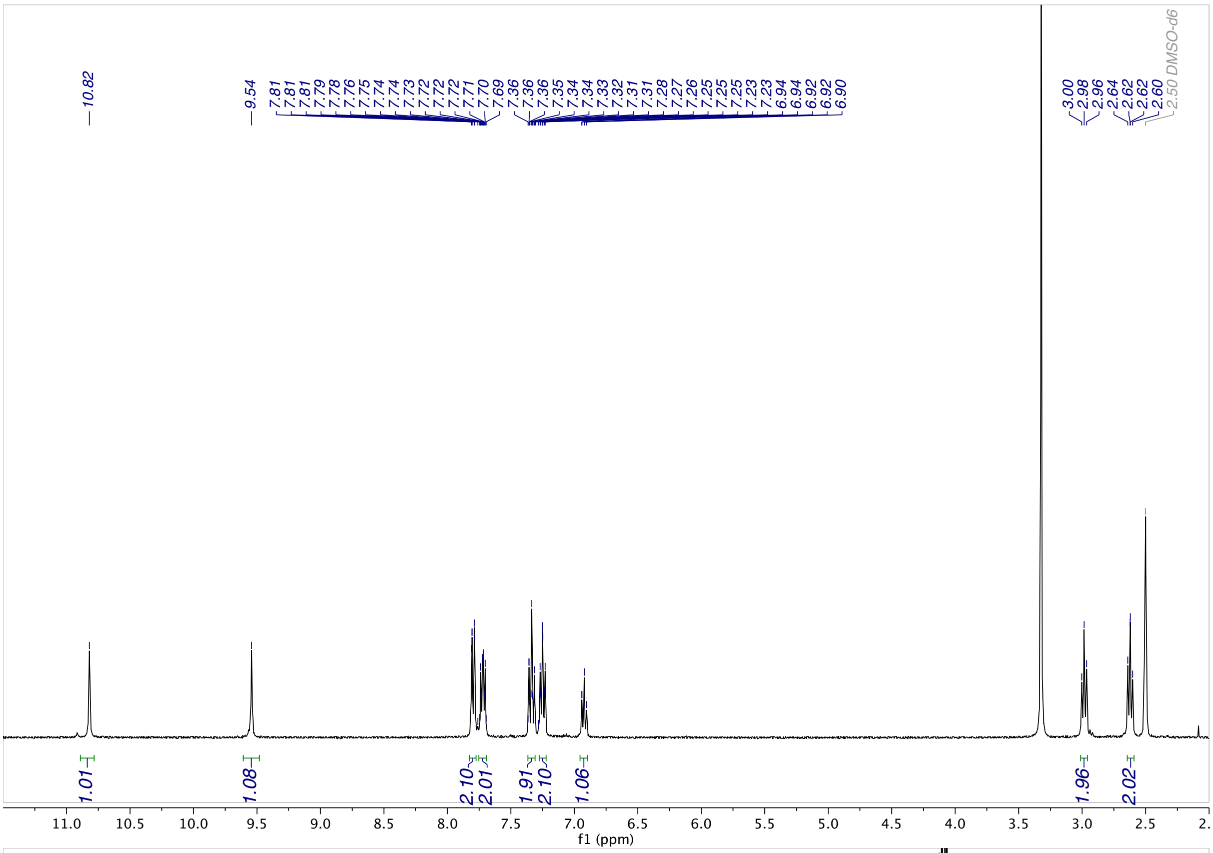

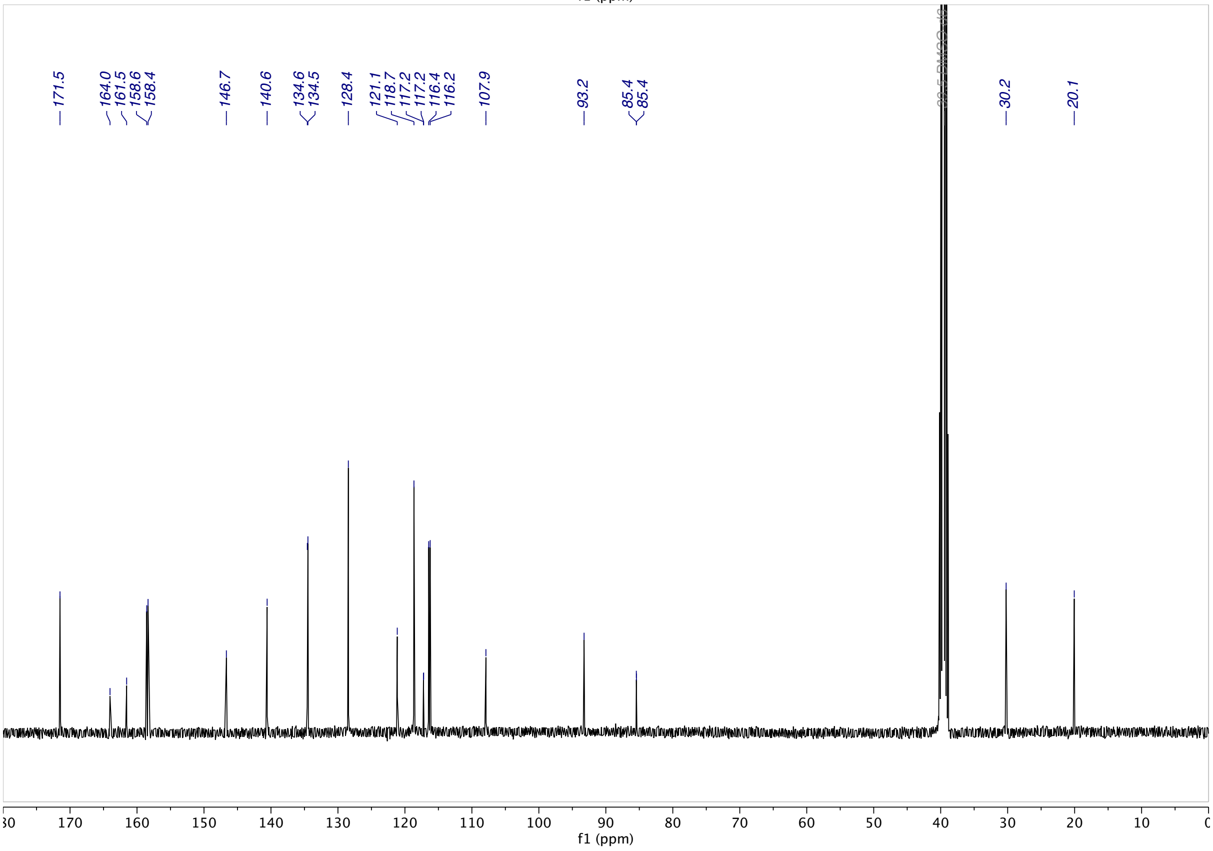

Molecular Modelling

3.1. General procedure

The binding mechanism of compounds under study were predicted by molecular docking. The molecular structure of Tyrosine Kinase Domain of ZAP-70, complexed with staurosporine, was retrieved from Protein Data Bank (PDBID:1U59). The system was prepared using the QuickPrep tool in MOE software.

Molecular docking was performed generating 200 poses using the Triangle Matcher as placement method (with London dG scoring function). Poses were therefore refined including the protein sidechains flexibility using an induced fit and changing the scoring function to GBVI/WSA dG, which estimates the free energy of binding of the ligand from a given pose.

3.2. Software

ZAP-70 modelling and molecular docking was conducted by Molecular Operating Environment (MOE), 2020.09; Chemical Computing Group ULC, 1010 Sherbooke St. West, Suite #910, Montreal, QC, Canada, H3A 2R7, 2021.
